# Proteome Analysis of Renoprotection Mediated by a Novel Cyclic Helix B Peptide in Acute Kidney Injury

**DOI:** 10.1038/srep18045

**Published:** 2015-12-10

**Authors:** Cheng Yang, Junjun Liu, Long Li, Meiyu Hu, Yaqiu Long, Xiaohui Liu, Tongyu Zhu, Xiao Huang, Shouliang Zhao, Shangfeng Liu, Ruiming Rong

**Affiliations:** 1 Department of Urology, Zhongshan Hospital, Fudan University, Shanghai, 200032, China; 2 Shanghai Key Laboratory of Organ Transplantation, Shanghai, 200032, China; 3 Department of Stomatology, Huashan Hospital, Fudan University, Shanghai, 200040, China; 4 Biomedical Research Center, Zhongshan Hospital, Fudan University, Shanghai, 200032, China; 5 CAS Key Laboratory of Receptor Research, Shanghai Institute of Material Medical, Chinese Academy of Sciences, Shanghai, 200032, China; 6 Department of Chemistry /Institutes of Biomedical Science, Fudan University, Shanghai, 200433, China; 7 Translational Center for Stem Cell Research, Tongji Hospital, Tongji University School of Medicine, Shanghai, 200065, China; 8 Department of Transfusion, Zhongshan Hospital, Fudan University, Shanghai, 200032, China; 9 Department of Plastic Surgery, Zhongshan Hospital, Fudan University, Shanghai, 200032, China

## Abstract

We developed a novel, erythropoietin-derived, non-erythropoiesis, cyclic helix B peptide (CHBP) that displays potent renoprotection against acute kidney injury (AKI). To determine the mechanism of CHBP-mediated protection, we investigated the proteomic profile of mice treated with CHBP in a kidney ischemia-reperfusion (IR) injury model. The isobaric tags for relative and absolute quantitation (iTRAQ)-labeled samples were analyzed using a QSTAR XL LC/MS system. In total, 38 differentially expressed proteins (DEPs) were shared by all experimental groups, while 3 DEPs were detected specifically in the IR + CHBP group. Eight significant pathways were identified, and oxidative phosphorylation was shown to be the most important pathway in CHBP-mediated renoprotection. The significant DEPs in the oxidative phosphorylation pathway elicited by CHBP are NADH-ubiquinone oxidoreductase Fe-S protein 6 (NDUFS6), alpha-aminoadipic semialdehyde synthase (AASS) and ATP-binding cassette sub-family D member 3 (ABCD3). The DEPs mentioned above were verified by RT-qPCR and immunostaining in mouse kidneys. We tested 6 DEPs in human biopsy samples from kidney transplant recipients. The trend of differential expression was consistent with that in the murine model. In conclusion, this study helps to elucidate the pharmacological mechanisms of CHBP before clinical translation.

Acute kidney injury (AKI) is a common problem in hospitalized patients, affecting >5% of all inpatients^1^ and 40% or more ICU patients. AKI significantly increases the risk of chronic renal disease and death, presenting a major burden to patients and healthcare systems^2^,^3^. However, no drug that prevents an increase in serum creatinine has been registered by the US Food and Drug Administration (FDA) until now.

Potential strategies for AKI therapy include drugs, cell therapy and gene intervention etc^4^. For instance, the α-melanocyte–stimulating hormone (α-MSH) has anti-inflammatory and anti-apoptotic activities, and has been proved to be able to ameliorate kidney ischemia reperfusion injury. AP214, as an α-MSH analogue, significantly reduced the composite endpoint consisting of death, need for renal replacement therapy, or a 25% reduction in renal function during a 90-day postoperative period in a clinical study^4^. Besides drugs, Xing *et al*. reported that bone marrow mesenchymal stem cells (MSCs) contributed to kidney repair after ischemia reperfusion injury in terms of enhancing peritubular capillaries and tubular epithelial cells repair and decreasing inflammation and apoptosis^5^. 15NP knocks down the *p53* gene, and is the first small interfering RNA (siRNA) to be systemically administered in humans. Quark company has recently completed a multicenter, randomized, double-blind, dose-escalation phase I trial of 15NP^6^. However, no drug is effective and applicable in clinic yet. Current AKI treatment is limited to support therapy and waiting. Therefore, there is an urgent need for a global health strategy to develop better drugs to reduce the enormous growing burden of AKI and its consequences.

Erythropoietin (EPO) is a hematopoietic hormone produced mainly by adult kidneys and has been routinely used in clinic for nearly 20 y to treat anemia. Apart from its erythropoietic effects, EPO also exhibits powerful tissue-protective effects against kidney IR injury^7^. However, four recently published clinical trials using high-dose EPO treatment following renal transplantation did not reveal any protective effect for short-term renal function, and in contrast reported an increased risk of thrombosis^8^. The application of EPO is restricted by its limited dosage which significantly breaks the balance between the benefit and risk. With the development of biochemistry and biotechnology, therapeutic peptides have become popular and are increasingly effective. Recently, our team and other researchers found that a novel linear peptide helix B surface peptide (HBSP) derived from EPO displays satisfactory renoprotective function by inhibiting inflammation and apoptosis in AKI models^9^,^10^,^11^. However, the 2-minute plasma half-life of HBSP restricts its application *in vivo*^12^. Therefore, our laboratory designed and synthesized a conformationally constrained cyclized helix B peptide (CHBP) with more than 30-folds and a 2.5-fold-longer half-life in human plasma and hepatocytes respectively *in vitro*, and a remarkably slow metabolism *in vivo* for the first time. CHBP also exerts potent renoprotective activity and significantly decreases local and systemic inflammation and apoptosis in the kidney^13^.

In the present study, to systemically and comprehensively demonstrate the mechanism of CHBP-mediated protection against AKI, we investigated the proteomic profile of mice treated with CHBP in a kidney ischemia-reperfusion (IR) injury model.

## Results

### CHBP improved renal function and histological structure, and decreased mitochondrial oxidative stress

**Figure 1:**
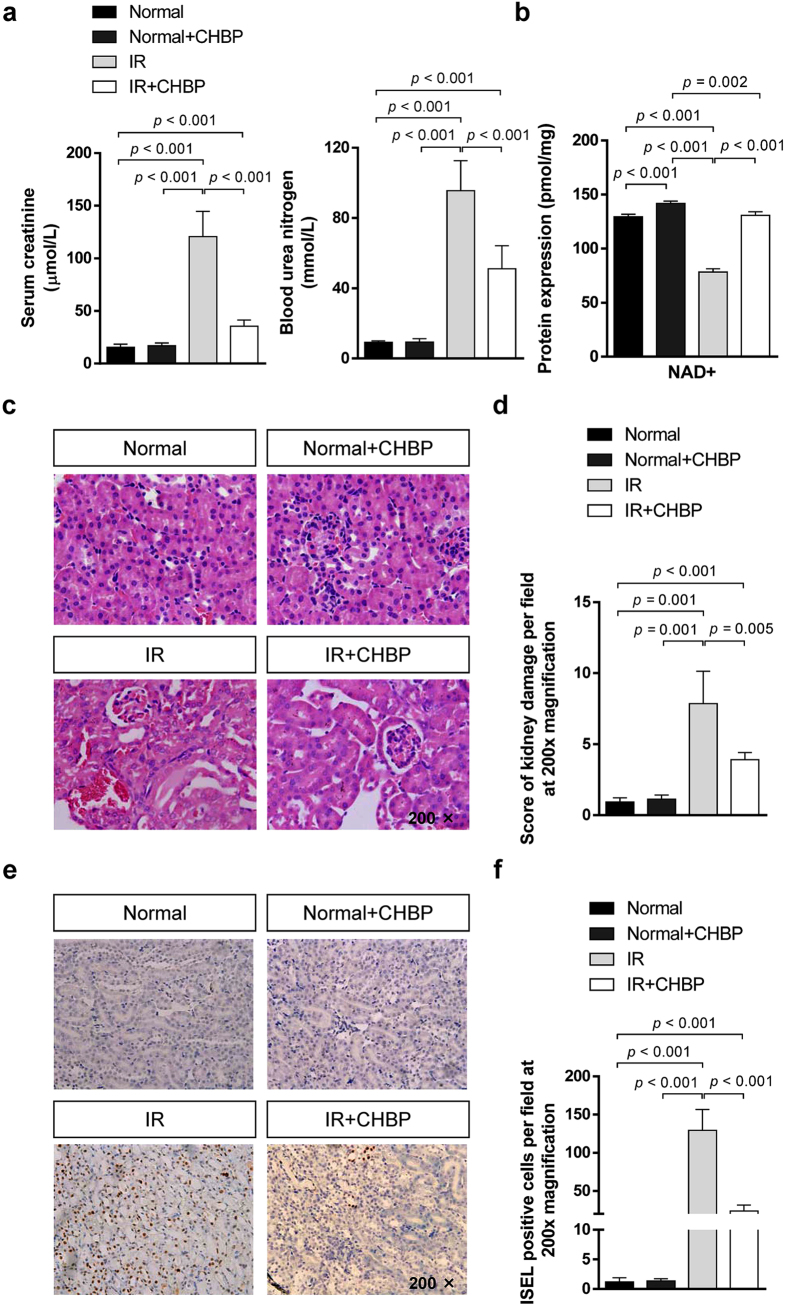
Assessment of renal function, NAD+ level, histological injury and apoptosis. In a kidney ischemia and 48 h reperfusion murine model, significantly lower levels of BUN and Scr were observed in the IR + CHBP group compared with the IR group (**a**). The level of NAD+ was significantly decreased in the IR group, but restored in the IR + CHBP group (**b**). Renal histologic damage was assessed in H&E stained sections (**c**). Mild tubular vacuolation and hemorrhage were observed in the IR + CHBP kidneys. In contrast, more severe tubular vacuolation, cell detachment (and luminal cell debris), protein casts and hemorrhage were observed in the IR kidneys. Semi-quantitative analysis revealed that CHBP significantly ameliorated IR-induced renal histological injury (**d**). The apoptotic cells were detected by ISEL assay (**e**). In the CHBP-treated group, ISEL + cells were dramatically reduced in the kidney post-reperfusion. In addition, CHBP did not influence the apoptosis without IR injury (**f**). Scr: serum creatinine; BUN: blood urea nitrogen; ISEL: *in situ* end labeling.

To evaluate the renoprotective ability of CHBP, we analyzed the levels of blood urea nitrogen (BUN) and serum creatinine (Scr), two indicators of renal function. In this kidney ischemia and 48 h reperfusion murine model, CHBP treatment improved the renal function in terms of much lower levels of BUN and Scr compared with those in the IR group (Fig. 1a). We further examined the level of mitochondrial oxidative stress in the kidney with and without CHBP treatment. After IR, the NAD+ level was significantly decreased compared to the normal group. However, CHBP recovered the NAD+ levels after IR injury. In addition, the level of NAD+ in the kidney was not influenced by CHBP treatment without IR injury (Fig. 1b). Renal histological assessment in each group was performed by H&E staining (Fig. 1c). Semi-quantitative analysis using a histological scoring system revealed that the tissue structure in the CHBP-treated group was well protected, with mild interstitial edema, fewer protein casts in tubular lumens, and less cellular infiltration, free from tubular epithelial vacuolation or detachment frequently found in the CHBP-untreated group (Fig. 1d).

### CHBP ameliorated apoptosis

Considering that apoptosis is a main process during kidney IR injury, we detected apoptotic cells using *in situ* end labeling (ISEL) assay (Fig. 1e). In the CHBP-treated group, ISEL+ cells were dramatically reduced in the kidney post-reperfusion. In addition, CHBP did not influence the apoptosis without IR injury (Fig. 1f).

### Identification of differentially expressed proteins

Isobaric tags for relative and absolute quantitation (iTRAQ)-labeled samples were analyzed using a QSTAR XL LC/MS system to obtain better coverage of the tissue proteome. The protein signature was searched against the UniProt database. In total, 1102 proteins were identified, 139 of which displayed fold changes >1.5 (*p* < 0.05) in all experimental groups compared to the normal group (Table S1); these proteins were identified as differentially expressed proteins (DEPs). The distributions of the 139 DEPs and their overlapping expression in different groups were illustrated by Venn diagram analysis as shown in Figure S1. As indicated in Figure S1, 38 DEPs were shared by all experimental groups, 8 DEPs were only detected in the normal + CHBP kidney group, and 14 DEPs were shared by the normal + CHBP and IR + CHBP groups, indicating that these 14 proteins can be affected by CHBP and are modeled independently (in both healthy and injury conditions). In contrast, 3 DEPs were detected specifically in the IR + CHBP group, suggesting a disease-specific correlation involved in the mechanism mediated by CHBP during kidney IR injury.

### Gene Network Modules Identified by WGCNA

Thus far the analyses focused on individual gene changes between experimental and normal groups, but did not reveal the crucial shift of gene networks. To understand the co-expression relationships between the 139 DEPs at a systems level, we performed weighted gene co-expression network analysis (WGCNA)^14^,^15^.

**Figure 2:**
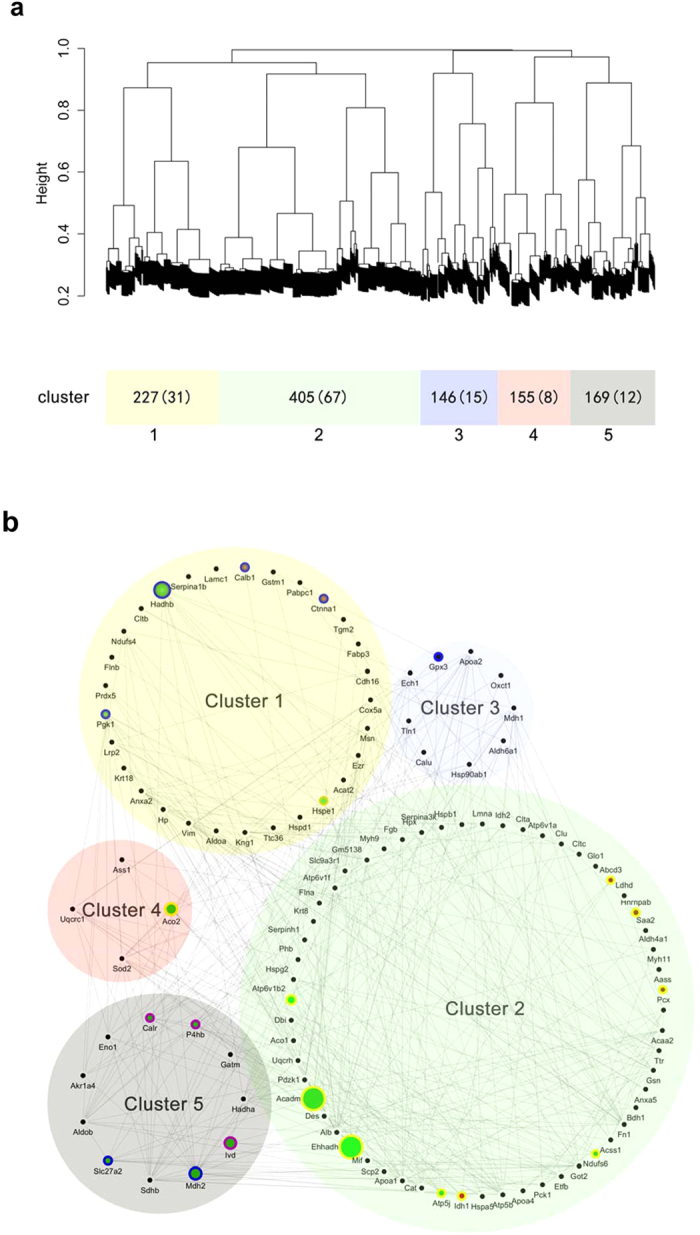
Gene network modules enriched in all experimental groups. Hierarchical cluster tree indicating co-expression modules in all experimental groups identified using WGCNA. Modules correspond to branches and are labelled by colour as indicated by the first colour band underneath the tree (**a**). Gene interaction network in co-expression modules. Size of the dots represents hubness. Spot color: red, upregulated; green, downregulated. Spot border color: purple, proteins differentially expressed in only the IR + CHBP group; yellow, proteins differentially expressed in both the IR + CHBP and normal + CHBP groups; blue, proteins differentially expressed in only the normal + CHBP group; black, protein interaction partners extracted from the STRING database (combined information with co-expression, direct interaction). Edge width: interaction confident score (**b**).

**Figure 3:**
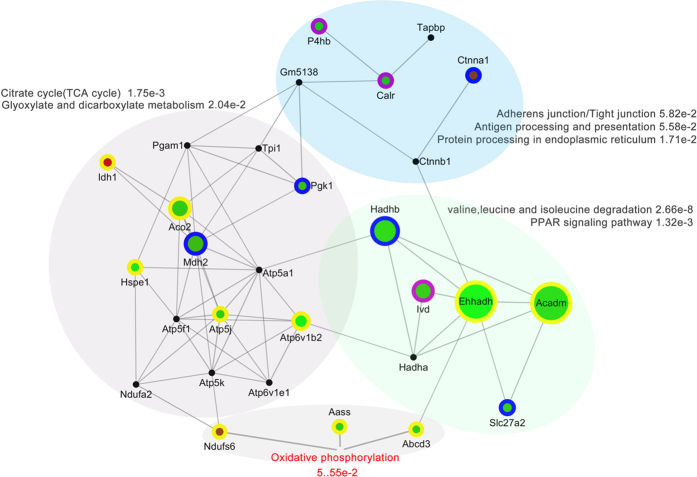
Different functional modules and significant pathways involved in CHBP-mediated IR kidney protection. Genes: spots, with the sizes of the spots indicating the importance of the genes. Spot color: red, upregulated; green, downregulated. Spot border color: purple, proteins differentially expressed in only the IR + CHBP group; yellow, proteins differentially expressed in both the IR + CHBP and normal + CHBP groups; blue, proteins differentially expressed in only the normal + CHBP group; black, protein interaction partners extracted from the STRING database (combined information with co-expression, direct interaction). Edge width: interaction confident score.

This unsupervised and unbiased analysis identified multiple gene-network modules corresponding to clusters of correlated genes, five modules were identified: yellow, green, blue, red, and gray (Fig. 2a). Gene network analysis of the modules revealed a hierarchical organization of highly connected genes in each module, through which key hub genes in the network can be identified (Fig. 2b). Using the WGCNA measure of intramodular gene connectivity, we identified 29 hub genes across all modules. To list, NADH-ubiquinone oxidoreductase Fe-S protein 6 (NDUFS6), alpha-aminoadipic semialdehyde synthase (AASS) and ATP-binding cassette sub-family D member 3 (ABCD3) are significant hub genes. Others including isocitrate dehydrogenase 1 (IDH1), aconitate hydratase (ACO2) and malate dehydrogenase (MDH2) *et al*., which are centrally located in their respective modules and may thus be critical components within the network (Fig. 3). The discovery of these modules would certainly help with better understanding of the coordinately expressed genes in all experimental groups.

### Functional analysis

Gene Ontology term enrichment analysis revealed the involvement of the modules in oxidative phosphorylation and in other diverse signaling and physiological pathways (Fig. 3). Molecular function analysis indicated that these modules are related to a number of molecular activities such as protein binding activity, oxygen transport and other binding activities. According to the cellular component analysis, most of the genes belong to mitochondrion, extracellular region, cytoplasm and other cell projections and organelles. The biological process analysis indicated that the majority of these genes play roles in transport, signal transduction, and ATP hydrolysis-coupled proton transport (Table S1).

These DEPs were also systematically analyzed using the STRING database, Kyoto Encyclopedia of Genes and Genomes pathway and Cytoscape software for the identification of various associated networks. These analyses indicated the association of these DEPs with 8 significant pathways including oxidative phosphorylation signaling; citrate cycle signaling; adherens junction/tight junction signaling; glyoxylate and dicarboxylate metabolism signaling; antigen processing and presentation signaling; protein processing in endoplasmic reticulum signaling; alanine, aspartate and glutamate metabolism signaling; and PPAR signaling pathways (Fig. 3). Moreover, oxidative phosphorylation is the most important pathway in CHBP-mediated renoprotection because the components of this activity are specifically expressed in the IR + CHBP kidney group.

### Verification of the DEPs in mice and human kidneys

**Figure 4:**
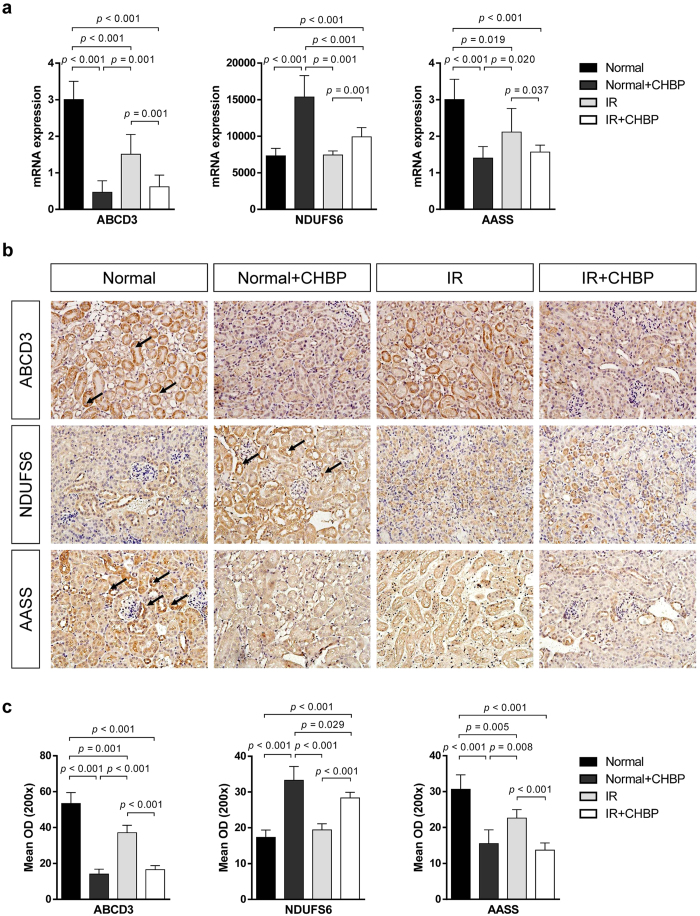
Verification of NDUFS6, ABCD3 and AASS expression in mouse kidneys. The levels of NDUFS6, ABCD3 and AASS mRNA expression were examined by RT-qPCR (**a**). The levels of NDUFS6, ABCD3 and AASS protein expression were detected in mouse kidneys by immunostaining (**b**).

**Figure 5:**
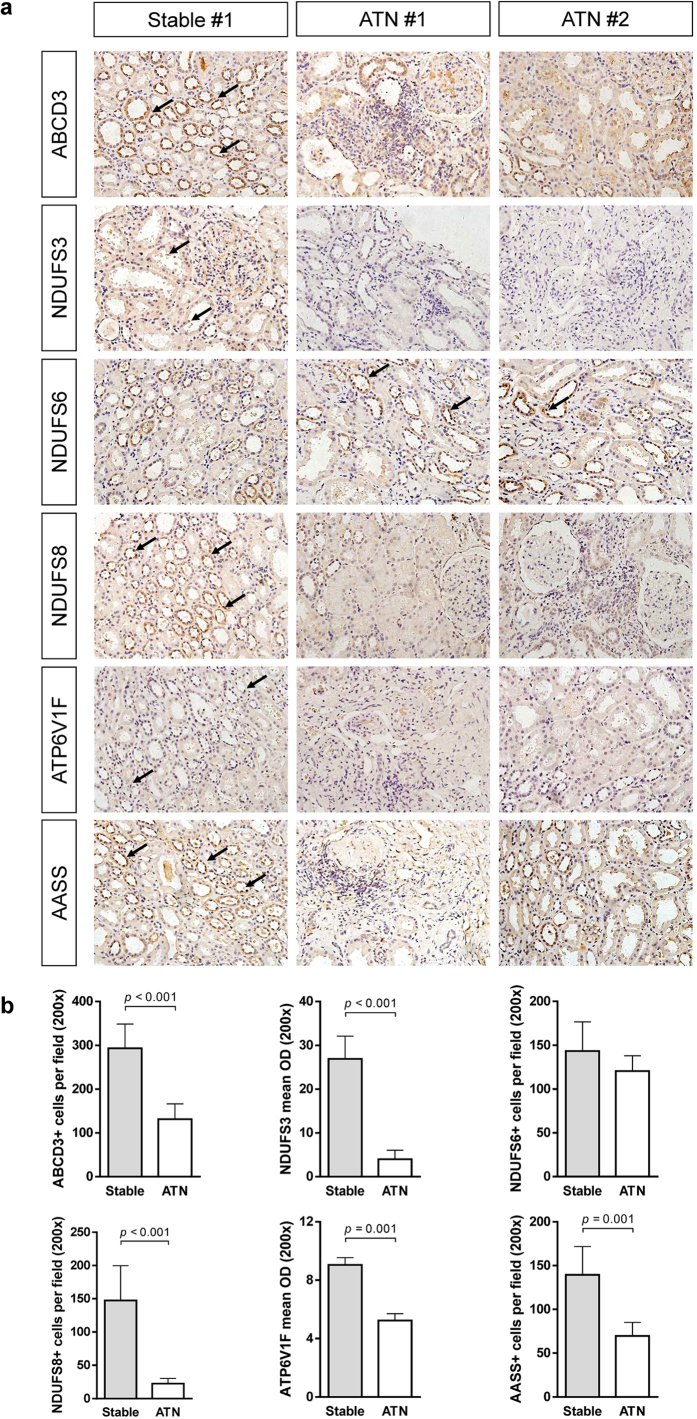
Verification of ABCD3, NDUFS3, NDUFS6, NDUFS8, ATP6V1F and AASS expression in human transplant kidneys. The expression levels of these 6 proteins, which displayed significant changes in their proteomic profiles, were detected in human kidney samples from biopsies of kidney transplant recipients (**a**). Semi-quantity analysis indicated significant decrease in the expression levels of all except NDUFS6 in the ATN group (**b**). ATN: acute tubular necrosis (ATN); stable: biopsy-proven stable.

The important proteins significantly altered by CHBP treatment were also evaluated and confirmed at the mRNA level (Fig. 4a). Moreover, we also examined NDUFS6, ABCD3 and AASS protein expression in mouse kidney using immunostaining (Fig. 4b). These proteins are primarily in the tubular area, and semi-quantitative analysis confirmed the proteomic profiling results (Fig. 4c). Finally, we tested these 6 DEPs in human kidney samples from biopsies of kidney transplant recipients (Fig. 5a). The acute tubular necrosis (ATN) transplant kidney was similar to the IR kidney in the murine model because the major reason for ATN in clinical transplantation is ascribed to IR injury without rejection injury^16^. Due to the impossibility of performing a renal biopsy on a healthy volunteer, we compared the expression of these 6 significant proteins between the ATN group and biopsy-proven stable (stable) group. The trend of differential expression is consistent with that in the murine model (Fig. 5b).

### Verification in the IR *in vitro* model

**Figure 6:**
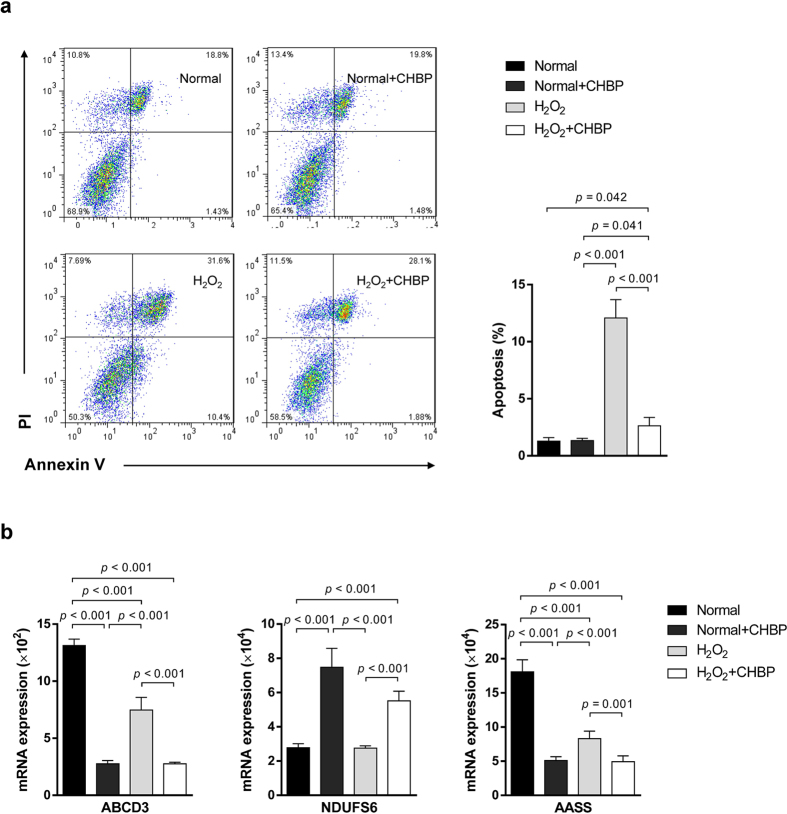
Verification in the IR *in vitro* model. CHBP significantly inhibited apoptosis of TECs compared with that in the H_2_O_2_ group (**a**). The change patterns of ABCD3, NDUFS6 and AASS were consistent with those in the mice and human kidneys (**b**).

To further confirm the proteomic result, we used an IR *in vitro* model in the condition of H_2_O_2_-induced oxidative stress injury. CHBP significantly inhibited the apoptosis of TECs compared with that in the H_2_O_2_ group (Fig. 6a). Besides, the change patterns of ABCD3, NDUFS6 and AASS were consistent with those in the mice and human kidneys (Fig. 6b).

## Discussion

These results suggested that the CHBP-mediated renoprotection against IR injury is primarily dependent on energy metabolism and oxidative stress. Hypoxic event is an initiation of AKI, which induces tubular cell apoptosis^17^. For instance, hypoxia inducible factor 1-alpha (HIF-1 alpha) is induced during reperfusion after renal ischemia and is critical for proximal tubule cell survival^18^. Interestingly, in our previous study, we found that CHBP-mediated renoprotection is involved in energy metabolism^13^. This renoprotective activity increased the expression of AMP-dependent protein kinase (AMPK) α, which acts to restore ATP levels back to homeostasis. We also found that CHBP regulates the AMP-activated protein kinase (AMPK)/Akt/mTOR axis, which also functions in protein synthesis and cell proliferation and survival.

The renoprotection pathways mediated by CHBP shown in the present study supports our previous study. IDH1 catalyzes the oxidative decarboxylation of isocitrate, producing alpha-ketoglutarate and CO_2_. ACO2 catalyzes the stereo-specific isomerization of citrate to isocitrate. MDH2 is an enzyme that reversibly catalyzes the oxidation of malate to oxaloacetate using the reduction of NAD^+^ to NADH. This reaction is part of many metabolic pathways, including the citric acid cycle. Phosphoglycerate kinase 1 (PGK1) is a major enzyme used in glycolysis, in the first ATP-generating step of the glycolytic pathway. The PPAR-γ pathway is being explored for their antioxidant and anti-inflammatory activities against IR injury of various organs including kidney^19^. Accumulating experimental data have revealed that natural and synthetic PPAR-γ ligands exert beneficial effects against IR injury. Activation of PPAR-γ using a high-affinity ligand nitroalkene derivatives of oleic acid significantly inhibited mitochondria-dependent apoptotic cascade in human renal tubular epithelial cells (TECs)^20^. Protein disulfide-isomerase (P4HB), catenin alpha-1 (CTNNA1) and calreticulin (CALR) were significant DEPs involved in adherens junction/tight junction signaling, antigen processing and presentation signaling and protein processing in endoplasmic reticulum signaling. Calreticulin was been demonstrated to play a key role in the adaptation and survival of thick ascending limb of Henle’s loop cells under hyperosmotic NaCl stress conditions due to its calcium binding and storage capacity^21^. It is given that AKI results in incomplete tubular repair. Renal IR injury is a well-established cause of renal fibrosis, which is a main cause of end-stage renal disease^22^,^23^. Interestingly, in a rat renal fibrosis model induced by unilateral ureteric obstruction, CALR was consistently up-regulated from the earliest stages of the process of renal fibrosis, even before the accumulation of extracellular matrix (ECM) in TECs^24^. In a subsequent study, the pro-fibrosis effect of CALR in kidneys is proved in an *in vivo* model, and knockdown of CALR expression reduced the development of tubulointerstitial fibrosis^25^. In the present study, the reduction of CALR by CHBP treatment might be an explanation for CHBP-mediated anti-fibrosis in IR-induced tubulointerstitial fibrosis in our previous study^13^.

Oxidative phosphorylation is associated with mitochondrial stress. NAD+ and NADH play crucial roles in a variety of biological processes including energy metabolism, mitochondrial functions, and gene expression^26^. Studies have indicated that NAD+ administration can profoundly decrease oxidative cell death in ischemic organ injury^27^,^28^. Here we found that the level of NAD+ in kidneys was significantly increased by CHBP, suggesting that CHBP ameliorates the oxidative phosphorylation in the presence of IR injury. The significant DEPs in the oxidative phosphorylation pathway elicited by CHBP are NDUFS6, AASS and ABCD3.

NDUFS6 is a nuclear gene that belongs to mitochondrial respiratory chain complex I, the deficiency of which is the most common energy generation disorder. NDUFS6 mutations are a cause of lethal neonatal mitochondrial complex I deficiency^29^. A recent study demonstrated that complex I deficiency due to inhibited NDUFS6 expression is an independent cause of renal impairment^30^. Although NDUFS6 is an indispensable gene responsible for energy generation in mitochondria, interestingly, CHBP significantly decreased NDUFS6 expression in the kidney. Given that CHBP dramatically improved renal function and ameliorated tissue injury in a mouse IR model at 48 h post-reperfusion^13^, this activity could be explained by a feedback regulatory mechanism. In our previous study regarding the mechanism through which HBSP protects the kidneys against 30 min ischemia followed by 48 h reperfusion injury, we found that the expression of heterodimer receptor βcR/EPOR, which is the receptor of HBSP, was upregulated by IR injury but downregulated by HBSP. IR injury increased the expression of this endogenous protective heterodimer receptor as a self-protection mechanism. In contrast, the renoprotective effect of HBSP treatment is sufficient and physiological upregulation is therefore unnecessary^10^. This negative feedback mechanism may also exist for CHBP-mediated renoprotection. Serum creatinine and blood urea nitrogen levels in the CHBP-treated group at 48 h post-reperfusion were even lower than at 6 h and 12 h^13^, while no fluctuation of renal function was observed during the entire IR process. This dramatic improvement in renal function indicates that CHBP provides powerful renoprotection that restored the endogenous cellular physiological protective response to baseline levels quickly. Therefore, NDUFS6 expression is significantly lower after CHBP treatment.

ABCD3 belongs to the ATP-binding cassette (ABC) transporter family, which is composed of 48 multispan membrane proteins and further divided into seven subfamilies (from ABCA to ABCG) according to their sequence homology^31^. ABC transporters promote or regulate the transport of specific substrates including amino acids, peptides, and proteins across various biological membranes^31^. ABCD3 is frequently used as a marker of peroxisomes^32^, which are important for cellular protection against inflammatory damage^33^. No current studies have examined the relation between ABCD3 and kidney injury, however there has been report of decrease of ABCD3 expression in IR hCMEC/D3 cells in a stroke disease model^31^. In another neuron injury model, a significant increase in the expression of the ABCD3 gene encoding peroxisomal membrane protein-70 was observed in drug-protective neurons^33^. These results suggested that ABCD3 is a novel target for kidney anti-IR injury therapy and that peroxisome activity upregulation may be a therapeutic mechanism of CHBP.

AASS is a bifunctional enzyme that catalyzes the first two steps in lysine degradation. In humans, defects in one or both of these activities result in familial hyperlysinemia^34^. Thus far, no research regarding the relation between AASS and kidney injury has been reported. Lysine metabolism participates in several pathophysiological processes in different organs. For instance, acetylation, which is a lysine modification, has been reported to have neuroprotective effects against ischemic retinal degeneration^35^. Xie *et al*. found that lysine methylation negatively regulates FOXO3-mediated transcription and oxidative stress-induced neuronal cell death^36^. To elucidate the mechanism of AASS-mediated renoprotection by CHBP therapy, additional studies are required, such as the localization of AASS in the kidney and its epigenetic modifications. The limitations also include the molecular mechanisms of NDUFS6 and ABCD3, both of which are involved in the CHBP-mediated renoprotection, further studies still needs to be done in the future.

Numerous studies performed in animals and humans tried to seek out reliable biomarkers for AKI diagnosis or alert using “-omic” technologies, such as genomics, transcriptomics and proteomics^37^. Neutrophil gelatinase-associated lipocalin (NGAL) and kidney injury molecule (KIM-1) are classical biomarkers of AKI^38^,^39^. In this study, we analyzed the KIM-1 and NGAL expression in the proteomics data. Compared to normal group, KIM-1 was increased in the IR kidney (3.87 fold change), while CHBP reduced its expression level (1.51 fold change). Unfortunately, we didn’t find the protein NGAL in our proteomics data. These result indicated that CHBP ameliorated TECs injury.

In conclusion, this study provides the first comprehensive proteomic analysis of CHBP-regulated kidney protein expression during IR injury. Oxidative stress is one of the major mechanism associated with CHBP renoprotection against IR injury. Besides, metabolisms including amino acids and glucoses are also involved in the effect of CHBP. These results not only help to elucidate the pharmacological mechanisms of CHBP before clinical translation but also deepen the understanding of molecular and signal pathways in kidney IR injury.

## Methods

### Kidney IR injury

Male BALB/c mice weighing 20–25 g were obtained from Shanghai Slac Lab Animal, Co., Ltd., and bred in an SPF grade experimental animal room. For the kidney IR model, the mice were anesthetized intraperitoneally with pentobarbital at 0.1 g/kg. The core body temperature of each mouse was maintained at 37 °C using a homeothermic pad during the entire procedure. The abdominal cavity was exposed by a midline incision. Then, both kidneys were exposed, and the renal pedicles were carefully isolated. Bilateral renal occlusion was performed for 30 min using non-traumatic vascular clamps. Occlusion was confirmed by observing the blanching of the entire kidney surface. After removing the clips, the kidneys were observed for an additional 5 min to ensure the occurrence of color change, which indicated blood reperfusion. Subsequently, 1 mL of 37 °C saline solution was injected into the abdomen. The incision was sutured in two layers. All animal experiments were performed according to the guidelines of the Care and Use of Laboratory Animals of the Laboratory Animal Ethical Commission of Fudan University with good animal surgical research practices, and was approved by the the Animal Ethical Committee of Zhongshan Hospital, Fudan University.

To evaluate the CHBP-induced changes in the proteomic profiles, the mice were randomly divided into four groups (n = 5): (1) the normal group: normal mice without any treatment; (2) the normal + CHBP group: normal mice with one dose of 8 nmol/kg CHBP but without IR injury; (3) the IR group: IR injury with phosphate-buffered solution (PBS) injected i.p.; and (4) the IR + CHBP group: IR injury with one dose of 8 nmol/kg CHBP injected i.p. at the onset of reperfusion. All animals were ethically euthanized at 48 h post-reperfusion.

### Renal function and NAD+ measurement

Whole blood was drawn from the heart and centrifuged at 4 °C, 2500 rpm, for 25 min to obtain serum samples. The level of serum creatinine and blood urine nitrogen was measured by the Hitachi 7060 automatic biochemistry analyzer (Hitachi, Tokyo, Japan). Nicotinamide adenine dinucleotide (NAD+) protein level in the kidney was quantified using a commercially available kit (BioVision, Milpitas, CA, USA) according to the manufacturer’s instructions.

### Histological assessment

Hematoxylin and eosin (H&E) staining was performed to assess histological injury. The tissue sections were blind-labeled and reviewed by two renal pathologists. Renal damage was graded by the percentage of tubular injury, and the following histological scoring system was used to estimate the damage: 0 (<1%), 1 (1% to 10%), 2 (11% to 20%), 3 (21% to 40%), 4 (41% to 60%), 5 (61% to 75%), and 6 (>75%). The scores represent the severity of tubular injury (including proximal tubule brush border loss, cell swelling or vacuolization, and cell necrosis). Scores in the range from 1 to 2 represent mild injury, whereas scores in the ranges from 3 to 4 and 5 to 6 represent moderate and severe injuries, respectively.

### ISEL apoptotic cells

ISEL apoptotic cells were detected using a TUNEL Apoptosis Detection Kit (Millipore, MA, USA). Paraffin sections of 4 μm were digested by 40 μg/mL of proteinase K (EMD Chemicals, NJ, USA) for 15 min at 37 °C, incubated with TdT and digoxigenin-dUTP at 37 °C for 60 min, and transferred to a wash/stop buffer for 30 min. After adding anti-digoxigenin-peroxidase complex for 30 min, the tissue sections immersed in buffer were developed by 3′-amino-9-ethylcarbazole (AEC, DAKO, Carpinteria, USA) substrate (dark red color). Apoptotic cells were examined at 200× magnification in 20 fields for semi-quantitation.

### Protein extraction

The collected kidney samples were washed three times with PBS and then homogenized by crushing in liquid nitrogen with the aid of a mortar and pestle. Once tissues were ground to fine powder, 20% w/v lysis buffer (7M urea, 2 M thiourea 0.1% Phenylmethanesulfonyl fluoride, and 65 mM Dithiothreitol) was added and resuspend in ice for 30 min. Tissue lysate was transferred to sterile microcentrifuge tubes and centrifuged at 12000 g at 4 °C for 15 min. Clear supernatant was collected in separate microcentrifuge tubes.

### Protein digestion and iTRAQ labeling

High abundance proteins were depleted using ProteoMiner^TM^ protein enrichment kits (Bio-Rad), and protein samples were quantified following the Bradford method^40^. In total, 100 μg of protein from each group was reduced, alkylated, and trypsinized following the manufacturer’s instructions. Then, the digested samples were labeled with 4-plex iTRAQ reagents (Applied Biosystems, Inc., Foster City, CA). The sample labeling strategy for differential quantitative proteomic analysis was Normal-119, Normal + CHBP-113, IR-121 and IR + CHBP-114. The labeled digests were then mixed and dried using a rotary vacuum concentrator (Christ RVC 2-25; Osterode am Harz, Germany). The analytic processes were repeated twice, including protein depletion and digestion, iTRAQ labeling, SCX fractionation, and LC-MS/MS analysis.

## 2D LC-MS/MS analysis

The combined peptide mixture were fractionated by strong cation exchange (SCX) chromatography on a 20 AD high-performance liquid chromatography (HPLC) system (Shimadzu, Kyoto, Japan) using a polysulfoethyl column (2.1 × 100 mm, 5 μm, 300 Å; The Nest Group, Southborough, MA). Peptides were eluted with a linear gradient 0–45% buffer B (350 mM KCl, 10 mM KH_2_PO_4_ in 25% ACN, pH 2.6) in buffer A (10 mM KH_2_PO_4_ in 25% ACN, pH2.6) at a flow rate of 200 μL/min for 60 min at a flow rate of 200 μL/min. The absorbance at 214 and 280 nm was monitored and a total of 20 SCX fractions were collected along the gradient. These fractions were dried down by the rotary vacuum concentrator, dissolved in buffer A (5% acetonitrile, 0.1% FA) and analyzed on a QSTAR XL system (Applied Biosystems) interfaced with a 20AD HPLC system (Shimadzu). Peptides were separated on a Zorbax 300SB-C_18_ column (0.1 × 15 mm, 5 μm, 300 Å; Microm, Auburn, CA). The HPLC gradient was 5–35% buffer B (95% acetonitrile, 0.1% FA) in buffer A (5% acetonitrile, 0.1% FA) at a flow rate of 0.3 μL/min for 70 min. Survey scans were acquired from *m*/*z* 400–1800 with up to four precursors selected for MS/MS from *m*/*z* 100–2000.

### Data analysis

The MS/MS spectra were extracted and searched against the International Protein Index (IPI) database (version 3.45, Mouse) using ProteinPilot software (version 3.0, Applied Biosystems). Search parameters were set as follows: (1) sample type:iTRAQ 4-plex (Peptide labeled); (2) cysteine alkylation: MMTS; (3) digestion: trypsin; (4) instrument: QSTAR ESI; (5) special factors: none; (6) species: mouse sapiens; (7) ID Focus: biological modifications; (8) database: UniProtKB/Swiss-Prot FASTA (166705 mouse sequences); and (9) search effort: Thorough ID. MS tolerance was set to 100ppm and MS/MS tolerance was set to 0.6 Da.

The software reports two types of scores for each protein: unused ProtScore and total ProtScore. The unused ProtScore is a measurement of all the peptide evidence for a protein that is not better explained by a higher ranking protein. Using the following criteria to consider a protein for further statistical analysis: unused ProtScore >1.3 with at least one peptide with 95% confidence per repetition. The candidate proteins were examined in the Protein ID of the Protein Pilot software. Protein expression ratios were computed on basis of the peak area ratios of the peptides accounting for the same protein. The bias correction algorithm was applied to correct for unequal mixing during the combination of the different labeled samples, based on the assumption that most proteins do not change in expression. All quant ratios (both the average ratio for proteins and the individual peptide ratios) were corrected for the bias. The true value for the average ratio was expressed as an error factor (EF = 10^(95% confidence interval)^) and calculated as reported^41^. Protein quantification required an EF of less than 2 and a *P*-value < 0.05; only fold-changes >1.5 or <0.66 were considered significant. The peptide and proteins were exported, and saved as excel files.

### Weighted gene co-expression network analysis

A signed weighted correlation network was constructed using all genes that were expressed at an FPKM value of 0.1 or higher in at least one of the experimental groups. Soft power parameter was estimated and used to construct adjacency matrix for selected genes using the topological overlap measure, and the dynamic hybrid tree cut algorithm was used to detect clusters. The node centrality, defined as the sum of within-module connectivity measures, was used to rank genes for hubness within the entire module. To visualize the constructed networks by hard thresholding of edge distances, the closest 150 edges were represented using Cytoscape 3.0.0^42^.

### Gene ontology analysis

Functional annotation of the differentially expressed tissue proteins (*p* < 0.05 and | fold change | > 1.5) identified in different experimental groups in our quantitative proteomic analysis were performed with the Database for Annotation, Visualization, and Integrated Discovery (DAVID)^43^,^44^ and the Search Tool for the Retrieval of Interacting Genes/Proteins (STRING) database (version 9.1)^45^. Fisher exact test was performed for enrichment and Benjamin adjust was used to correct for multiple testing of the data.

### Real-time Quantitative PCR (RT-qPCR)

Total RNA was extracted from mouse kidneys using TRIzol reagent (Invitrogen, Carlsbad, USA) according to the manufacturer’s instructions. Total RNA (3 μg to 5 μg) was transcribed into complementary DNA using Superscript II reverse transcriptase (Invitrogen) and random primer oligonucleotides (Invitrogen). Gene-specific primers for mice were designed based on the sequences available from PubMed (Table S2 contains the list of primer sequences). RT-qPCR was performed using a MasterCycler RealPlex4 system (Eppendorf, Hamburg, Germany) in combination with Absolute QPCR SYBR Green premix (TaKaRa Bio, Inc., Tokyo, Japan). After a hot start (30 s at 95 °C), the amplification parameters were as follows: 5 s at 95 °C, 30 s at 55 °C, and 60 s at 72 °C for 45 cycles. The expression levels, which were normalized to that of GAPDH, were calculated using the 2^−ΔCt^ method.

### Immunohistochemistry

Immunohistochemical staining was performed on paraffin sections using a DAKO ChemMate EnVision Detection Kit (DAKO). Antigen retrieval was performed using 10 mM sodium citrate buffer (pH 6.0) in a steam bath maintained by high power microwave for 20 min. The sections were blocked and labeled with anti-ABCD3 (1:50 dilution, Abcam, Cambridge, MA, USA), anti-NDUFS3 (1:25 dilution, Abcam), anti-NDUFS6, anti-NDUFS8 (1:50 dilution, Abcam), anti-ATP6V1F (1:25 dilution, Santa Cruz Biotechnology, Dallas, TX, USA) and anti-AASS antibodies (1:50 dilution, Abcam) at 4 °C overnight. The antibody binding was revealed by diaminobenzidine (DAB).

### Tubular epithelial cells (TECs) stimulation and apoptosis detection

Mice renal proximal TECs were cultured and incubated with CHBP (1 μM) for 12 h, and stimulated with 50 μM H_2_O_2_ (Sinopharm Chemical Reagent Co., Ltd., Shanghai, China) for 45 min. PBS was used as a positive control, and normal TEC without any stimulation was used as a negative control. After washing and changing the medium, the TEC was cultured for 24 h with PBS or CHBP. Cell apoptosis was detected by an Annexin V-FITC Apoptosis Detection Kit (Merck, Darmstadt, German) as mentioned previously^13^. The experiment was repeated at least three times.

### Clinical Sample Collection

To verify the important protein markers identified from the mouse model, we collected kidney sample from patients. The AKI and stable samples were collected from 3 patients who received living-related or deceased kidney transplantation at Zhongshan Hospital, Fudan University. Two recipients with AKI received kidney graft biopsies due to increased serum creatinine, and the recipient with stable renal function received protocol biopsy. All the diagnoses were confirmed by pathological assessment. The demographic characteristics of patients were shown in Table 1. The tissue sample collection was approved by the Ethic Committee of Zhongshan Hospital, Fudan University, and the informed consent was obtained from all subjects. The tissue slides are from biopsy paraffin blocks. The methods were carried out in accordance with the approved guidelines and complied with the 1975 Declaration of Helsinki.

### Statistical Analysis

The data are presented as the mean ± standard deviation (SD). Statistical analysis (Statistical Package for the Social Sciences 18.0 software, SPSS Inc., Armonk, NY, USA) was performed using one-way ANOVA for more than two groups. The Scheffe test was used for post hoc analysis. Statistical significance was set at *p* < 0.05.

## Additional Information

**How to cite this article**: Yang, C. *et al*. Proteome Analysis of Renoprotection Mediated by a Novel Cyclic Helix B Peptide in Acute Kidney Injury. *Sci. Rep*. **5**, 18045; doi: 10.1038/srep18045 (2015).

## Supplementary Material

Supplementary Information

## Figures and Tables

**Table 1:** Demographic characteristics of patients

	Stable	ATN #1	ATN #2
Gender	Male	Male	Male
Age (y)	35	43	59
Serum creatinine (μmol/L)	88	139	176
eGFR (ml/min/1.73 m^2^)	90.9	51.7	36.7
Pre-transplant PRA (%)			
Class I	0	0	0
Class II	0	0	0
ISP			
CNI + MMF + Pred (Y/N)	Y	Y	Y
SRL + MMF + Pred (Y/N)	N	N	N

ATN: acute tubular necrosis; eGFR: estimated glomerular filtration rate; PRA: panel reactive antibody; ISP: immunosuppressive protocol; CNI: calcineurin inhibitor; MMF: mycophenolate mofetil; Pred: prednisone; SRL: sirolimus.
