# Supplementary material for: Proteome Analysis of Renoprotection Mediated by a Novel Cyclic Helix B Peptide in Acute Kidney Injury
Source: Sci Rep. 2015 Dec 10;5:18045. doi: 10.1038/srep18045 (PMC4674932; doi:10.1038/srep18045)
Supplement: Supplementary Information [file srep18045-s1.pdf]

Supplementary Materials

Proteome Analysis of Renoprotection Mediated by a Novel Cyclic Helix B Peptide in Acute Kidney Injury

Cheng Yang [1, 2, 9 *]; Junjun Liu [3 *]; Long Li [2 *]; Meiyu Hu [2,4], Yaqiu Long [5]; Xiaohui Liu [6]; Tongyu Zhu [1, 2]; Xiao Huang [7]; Shouliang Zhao [3]; Shangfeng Liu [3]; Ruiming Rong [1, 2, 8]

1. Department of Urology, Zhongshan Hospital, Fudan University, Shanghai, 200032, China

2. Shanghai Key Laboratory of Organ Transplantation, Shanghai, 200032, China

3. Department of Stomatology, Huashan Hospital, Fudan University, Shanghai, 200040, China.

4. Biomedical Research Center, Zhongshan Hospital, Fudan University, Shanghai, 200032, China.

5. CAS Key Laboratory of Receptor Research, Shanghai Institute of Material Medical, Chinese Academy of Sciences, Shanghai, 200032, China

6. Department of Chemistry /Institutes of Biomedical Science, Fudan University, Shanghai, 200433, China

7. Translational Center for Stem Cell Research, Tongji Hospital, Tongji University School of Medicine, Shanghai, 200065, China

8. Department of Transfusion, Zhongshan Hospital, Fudan University, Shanghai, 200032, China

9. Department of Plastic Surgery, Zhongshan Hospital, Fudan University, Shanghai, 200032, China

* These authors contributed equally to this article.

Figure S1

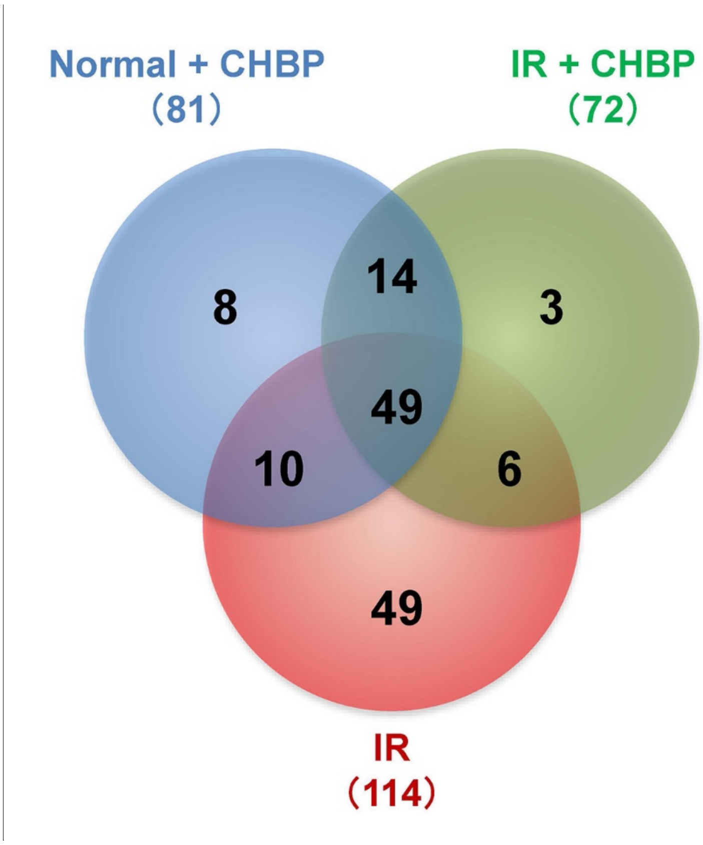

Venn diagrams and expression of 139 DEPs identified by iTRAQ in different experimental groups. The distributions of 139 DEPs and their overlapping expression in different groups are illustrated. Thirty-eight DEPs were shared by all experimental groups, 8 DEPs were only detected in the normal + CHBP kidney group, and 14 DEPs were shared by the normal + CHBP and IR + CHBP groups, indicating that these 14 proteins can be affected by CHBP and are modeled independently (in both healthy and injury conditions). In contrast, 3 DEPs were specifically detected in the IR + CHBP group, suggesting a disease-specific correlation involved in the mechanism mediated by CHBP during kidney IR injury. DEPs: differentially expressed proteins.

Figure S2

NDUSF6

Con...	Conf	Sequence
2.00	99	EVNENFAIDLIAQQPVNEV...
2.00	99	GVQVSPSGEK
2.00	99	IIACDGGGGALGHPK
2.00	99	ITHTGQVYDEK
2.00	99	QKEVNENFAIDLIAQQPVN...
2.00	99	TGTCGYCGLQFK
2.00	99	VYINLDK

Peptide: GVQVSPSGEK[IT8]

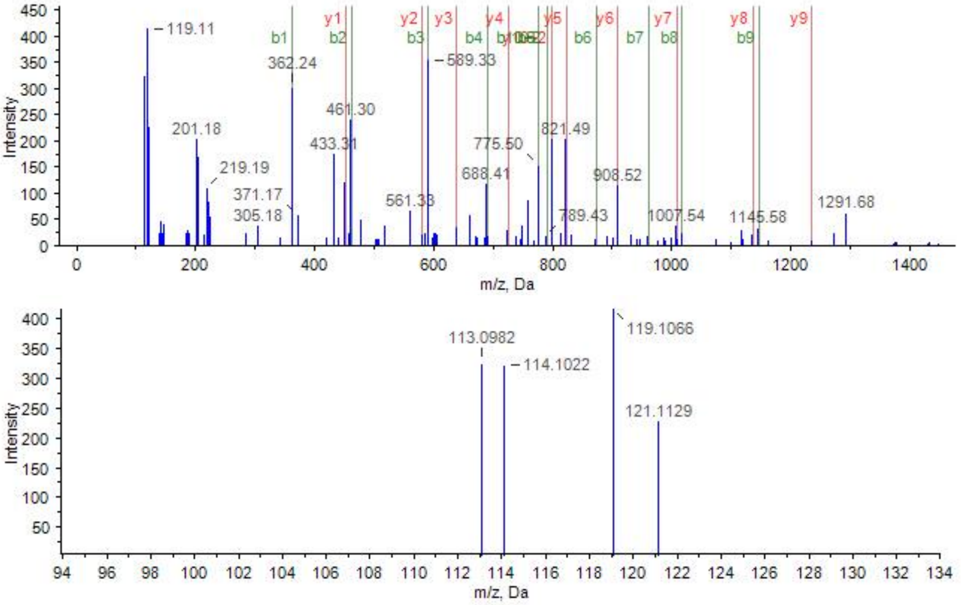

Peptide: IIAC[MSH]DGGGGALGHPK[IT8]

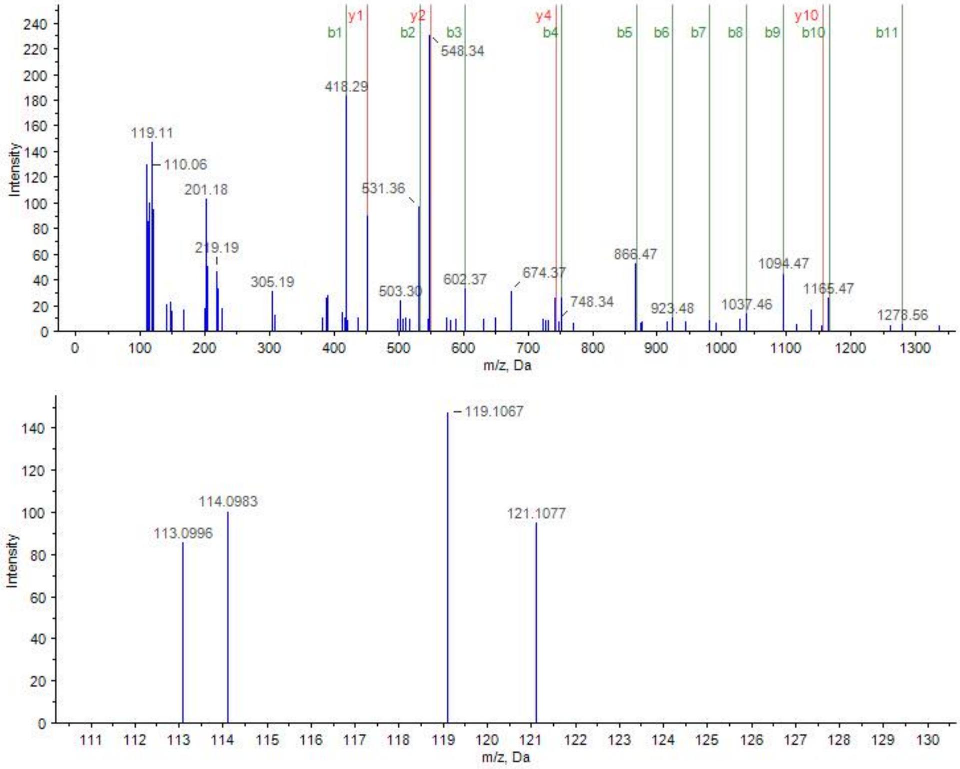

Abcd3

Con...	Conf	Sequence
2.00	99	LFYVPQRPYMTLGTLR
2.00	99	VLGELWPLFGGR

Peptide: LFYVPQRPYMTLGTLR

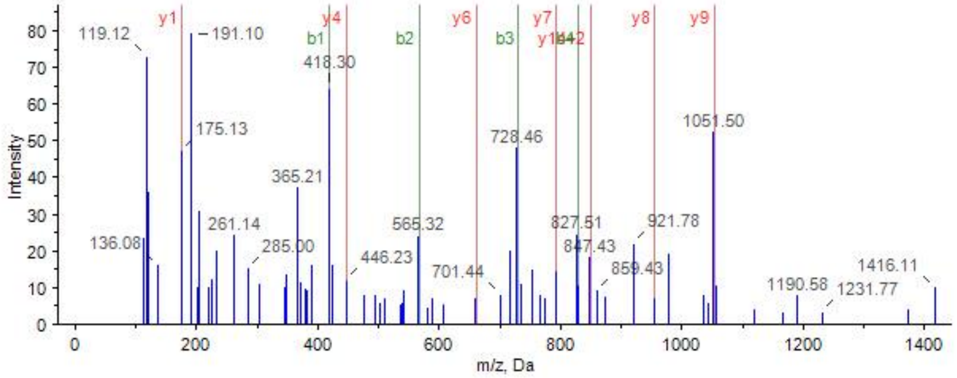

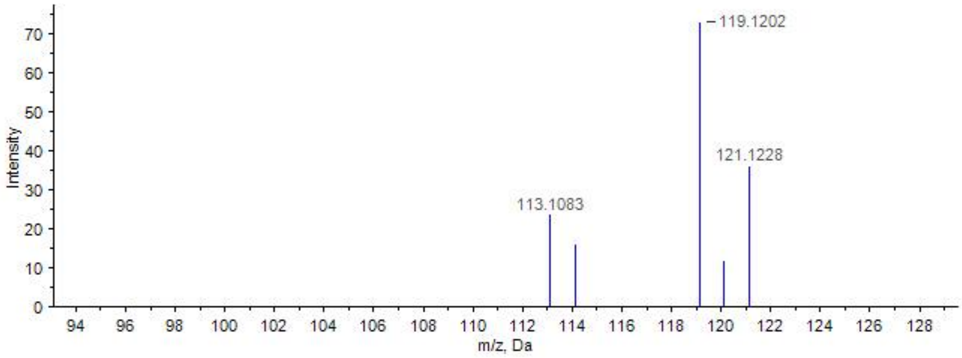

Peptide: VLGELWPLFGGR

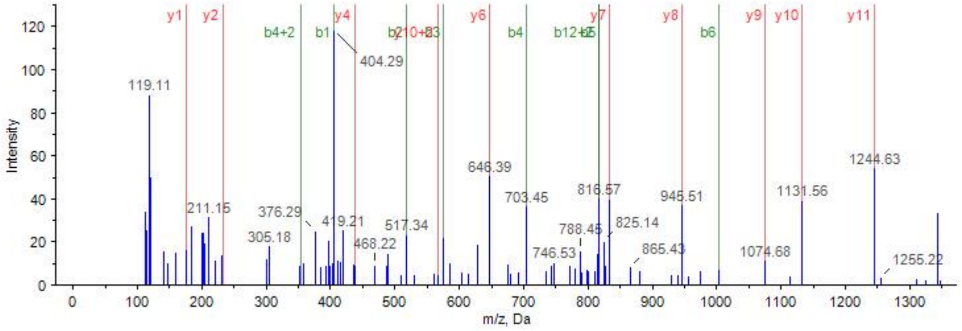

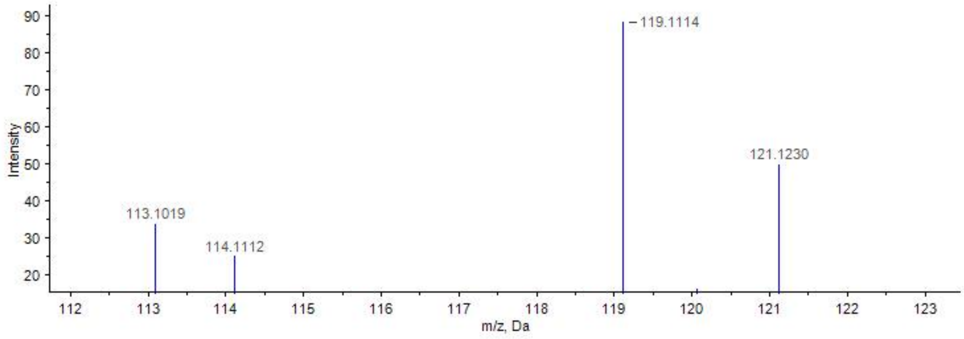

Aass

Con...	Conf	Sequence
2.00	99	EIYGPILER
2.00	99	GLMGPFTK
2.00	99	LGGDNTQLEAAEWLGLLGD...
2.00	99	VYGTVLSR

Peptide: EIYGPILER

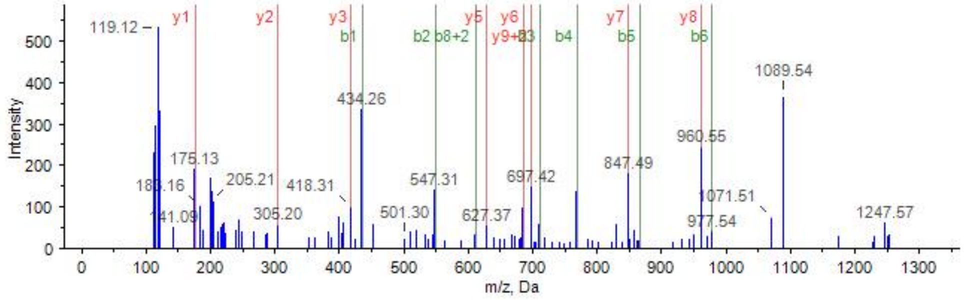

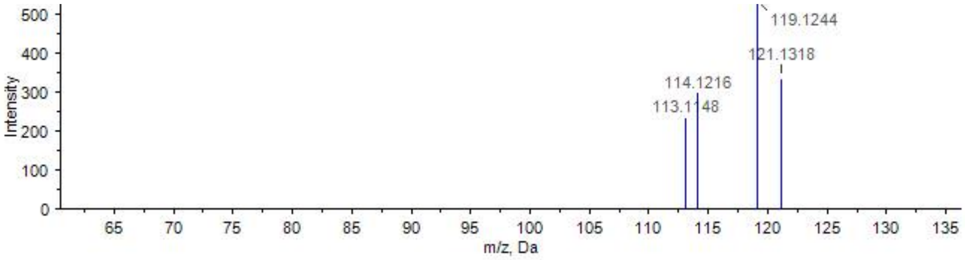

Peptide: GLMGPFTK

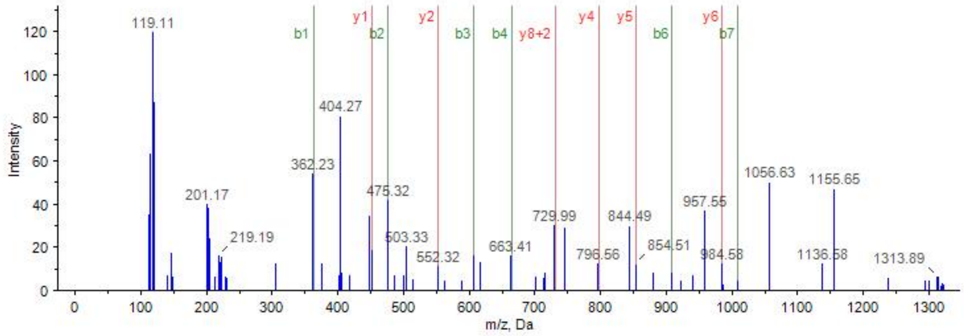

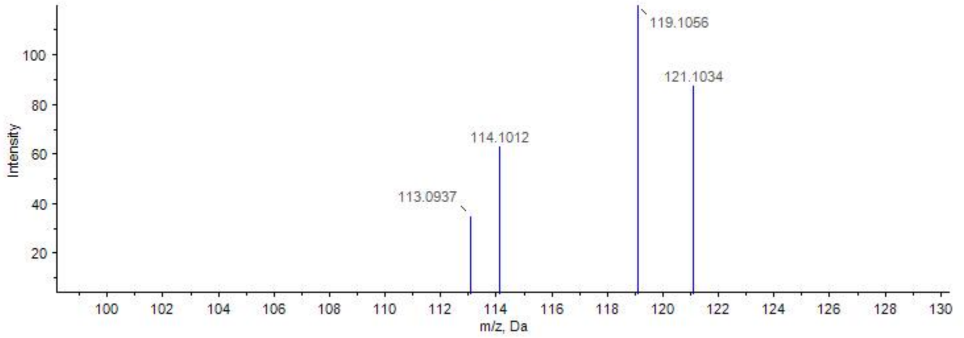

Acadm

Con...	Conf	Sequence
2.00	99	AFAGDIANQLATDAVQIFG...
2.00	99	ANWYFLLAR
2.00	99	EEIIPVAPEYDK
2.00	99	ENVLIGEGAGFK
2.00	99	GIAFEDVR
2.00	99	IAMGAFDR
2.00	99	IYQIYEGTAQIQR
2.00	99	TRPTVAAGAVGLAQR

Peptide: IPVAPEYDK[IT8]

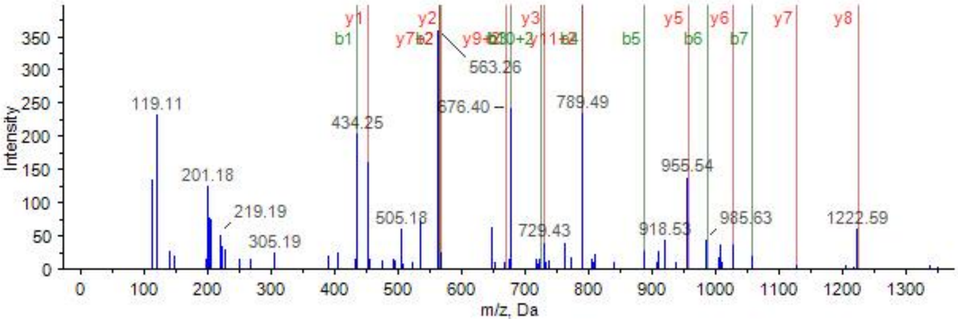

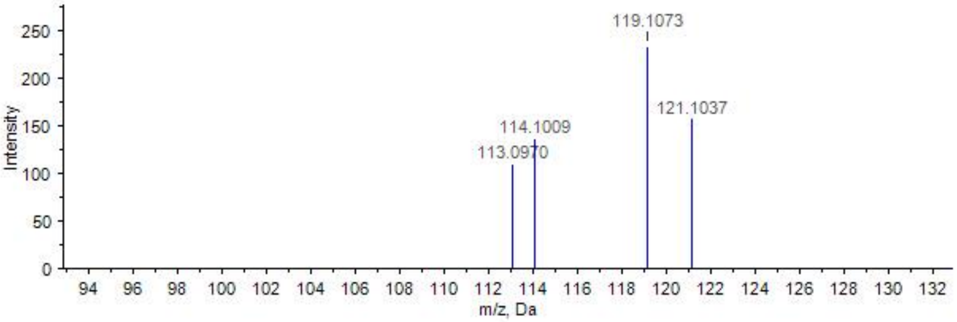

Ehhadh

Con...	Conf	Sequence
2.00	99	ELSSVDLVIEAVFEDMNLK
2.00	99	GIAISFAR
2.00	99	GTQLLPR
2.00	99	LGILDVVVK
2.00	99	PEVMLGILPGAR
2.00	99	QNPDIPQLEPSDYLR
2.00	99	SDPVEEAIK
2.00	99	TASAQPVSSVGVLGLGTMGR
2.00	99	VGFPEVMLGILPGAR
2.00	99	VVGVPVALDLITSGR
2.00	99	YSPIADMLCEAGR

Peptide: TASAQPVSSVGVLGLGTMGR

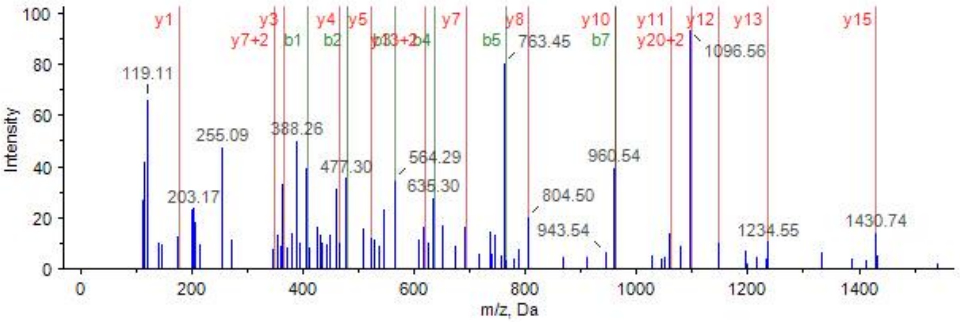

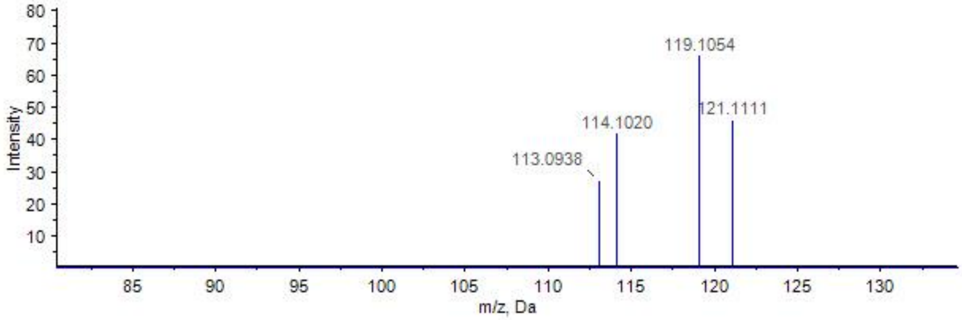

Slc27a2

Peptide: TFVPMTENIYNAIIDK[IT8]

Con...	Conf	Sequence
2.00	99	DAVSVFYVSR
2.00	99	GENVATTEVADIVGLVDFV...
2.00	99	TFVPMTENIYNAIIDK

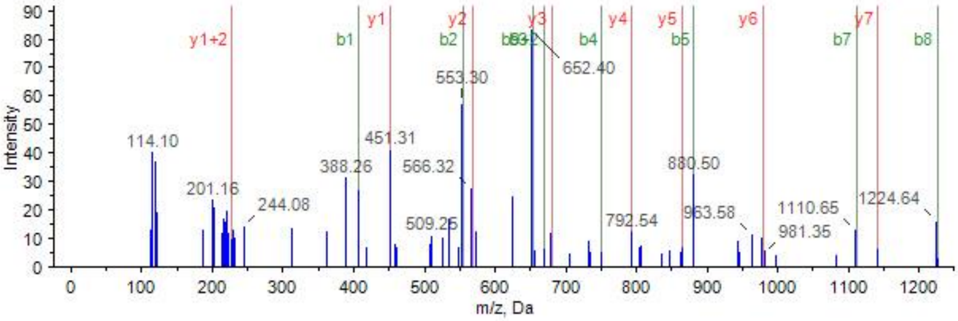

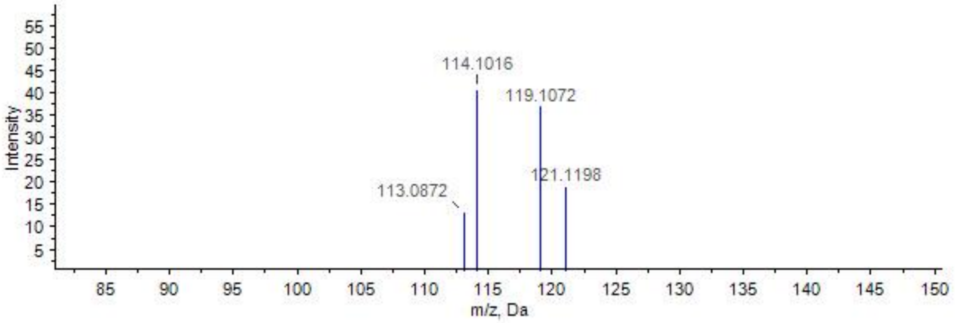

Ivd

Peptide: GITAFIVEK[IT8]

Con...	Conf	Sequence
2.00	99	FLQENLAPK
2.00	99	GITAFIVEK
2.00	99	GMPGFSTSK
2.00	99	IGQFQLMQGK
2.00	99	LISGEFIGALAMSEPNAGS...
2.00	99	TDLTAVPASR

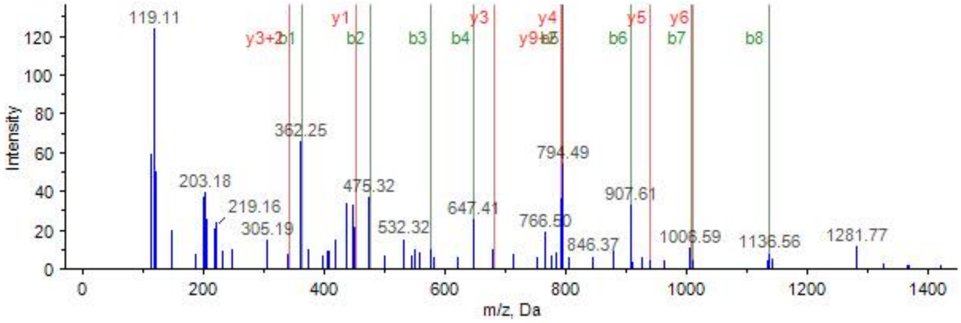

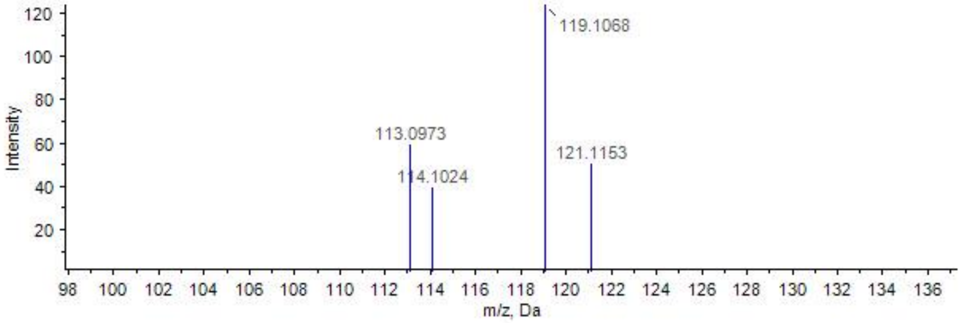

Hadhb

Con...	Conf	Sequence
2.00	99	AMDSDWFAQNYMGR
2.00	99	FNIWGGSLSLGHPFGATGCR
2.00	99	LAAAFAVSR
2.00	99	LNFLSPELPAVAEFSTNET...
2.00	99	MEQDEYALR
2.00	99	NIVVVEGVR

Peptide: AMDSDWFAQNYMGR

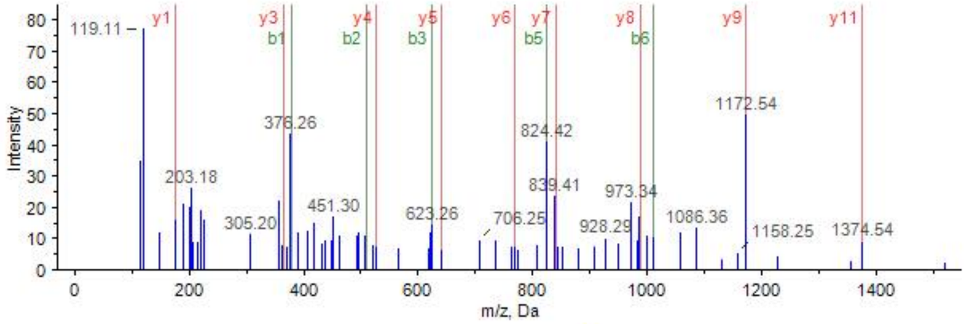

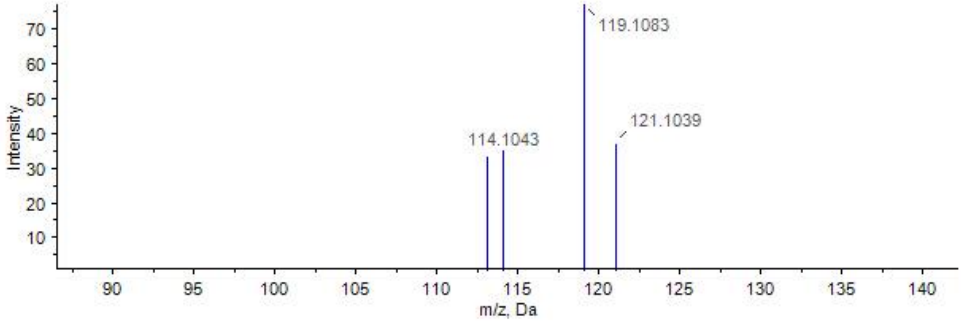

Atp6v1b2

Con...	Conf	Sequence
2.00	99	GFPGYMYTDLATIYER
2.00	99	GPVVLAEDFLDIMGQPINP...
2.00	99	IPQSTLSEFYPR
2.00	99	IYPEEMIQTGISAIDGMNS...
2.00	99	NGSITQIPILTMPNDDITH...
2.00	99	QIYPPINVLPSLSR
2.00	99	TPVSEDMLGR

Peptide: GFPGYMYTDLATIYER

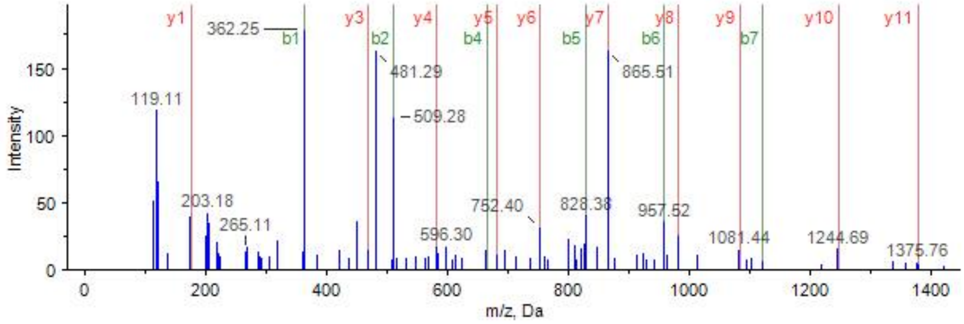

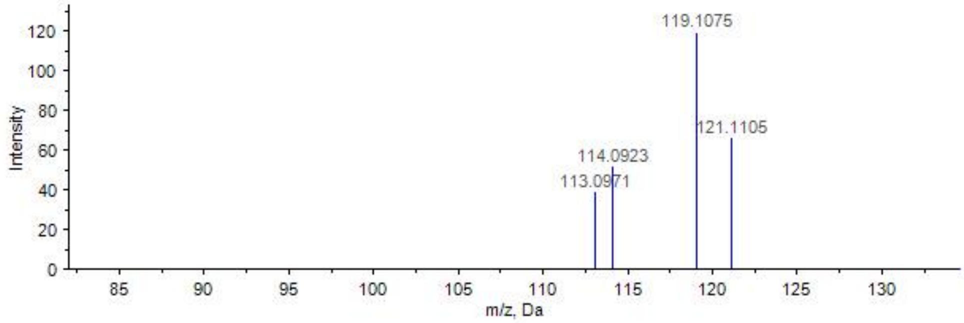

Atp5j

Peptide: FEVIDK[IT8]PQS

Con...	Conf	Sequence
2.00	99	FEVIDKPQS
2.00	99	GEMDTFPTFK
2.00	99	QASGGPVDIGPEYQQDLDR
2.00	99	RQASGGPVDIGPEYQQDLDR

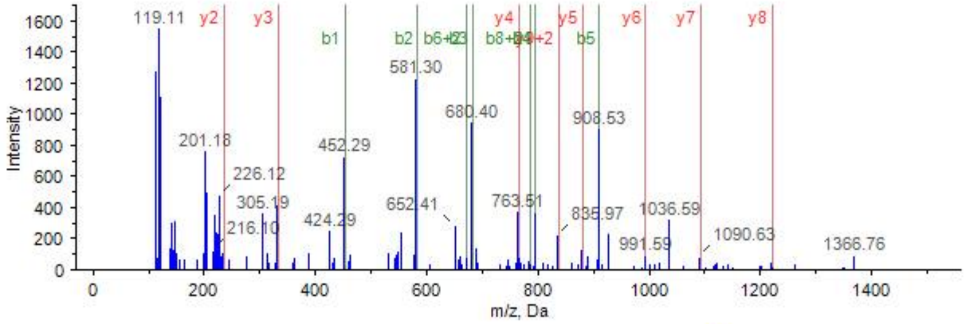

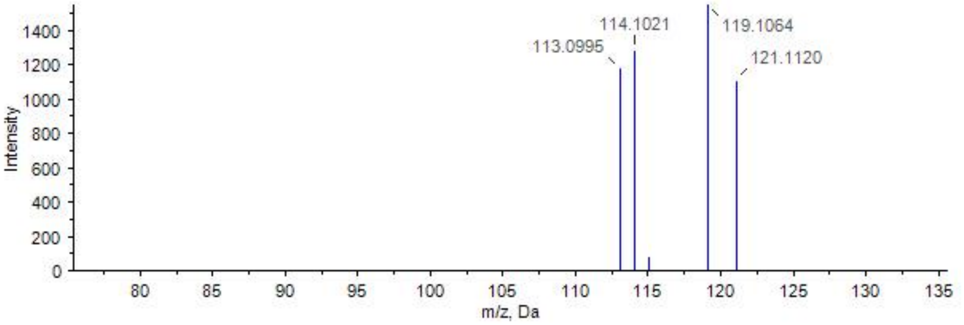

Hspe1

Peptide: GGIMLPEK[IT8]

Con...	Conf	Sequence
2.00	99	DSDILGK
2.00	99	GGIMLPEK
2.00	99	SAAETVTK
2.00	99	VLLPEYGGTK
2.00	99	VLQATVVAVGSGGK
2.00	99	VVLDDKDYFLFR

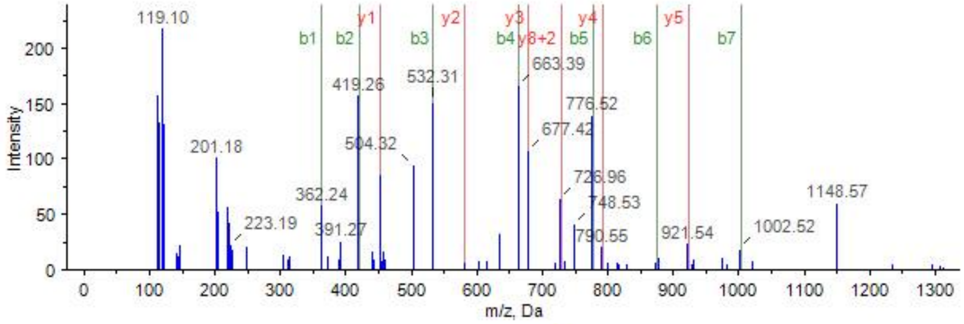

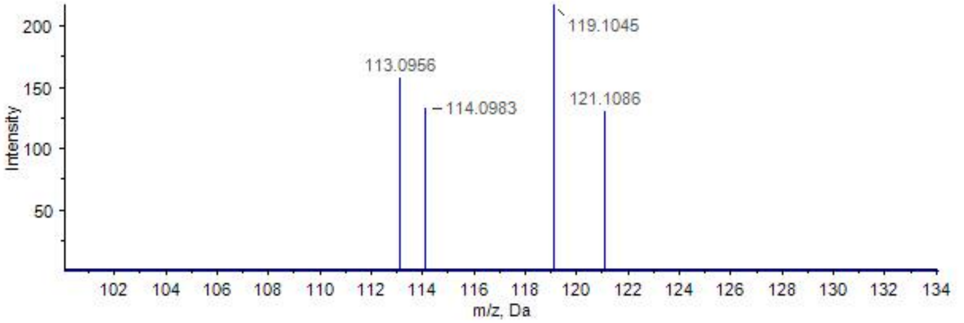

Mdh2

Con...	Conf	Sequence
2.00	99	AGAGSATLSMAYAGAR
2.00	99	AKVAVLGASGGIGQPLSLL...
2.00	99	GCDVVVIPAGVPR
2.00	99	IFGVTTLDIVR
2.00	99	IQEAGTEVVK
2.00	99	LTLYDIAHTPGVAADLSHI...
2.00	99	MIAEAIPELK
2.00	99	NLGIGK
2.00	99	TIIPLISQCTPK
2.00	99	VAVLGASGGIGQPLSL
2.00	99	VAVLGASGGIGQPLSLLL
2.00	99	VAVLGASGGIGQPLSLLLK
2.00	99	VAVLGASGGIGQPLSLLLK...
2.00	99	VAVLGASGGIGQPLSLLLK...
2.00	99	VAVLGASGGIGQPLSLLLK...
2.00	99	VDFPQDQLATLTGR
2.00	99	VNVPVIGGHAGK

Peptide: AGAGSATLSMAYAGAR

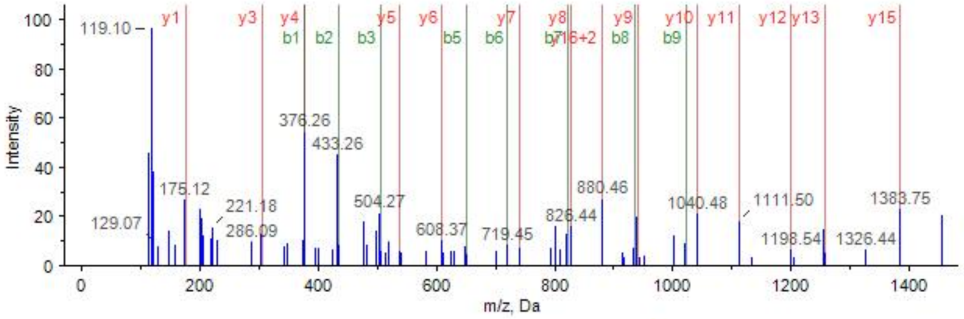

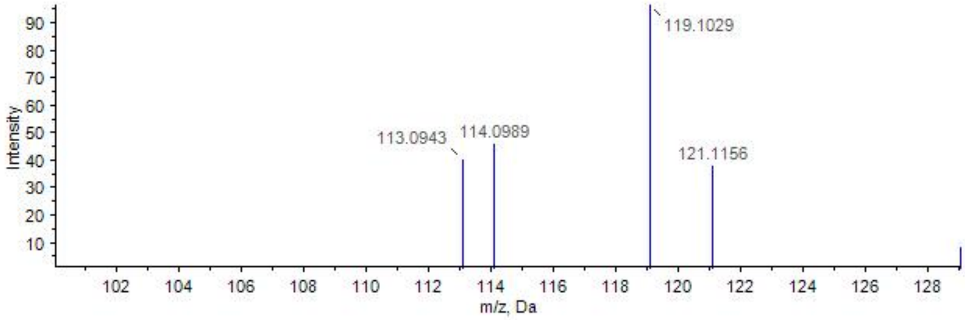

Pgk1

Con...	Conf	Sequence
2.00	99	AAVPSIK
2.00	99	AHSSMVGVNLPQK
2.00	99	ELNYFAK
2.00	99	LGDVYVNDAFGTAHR
2.00	99	QIVWNGPVGVFEWEAFAR
2.00	99	SLMDEVVK
2.00	99	VLNNMEIGTSLYDEEGAK

Peptide: QIVWN[Dea]GPVGVFEWEAFAR

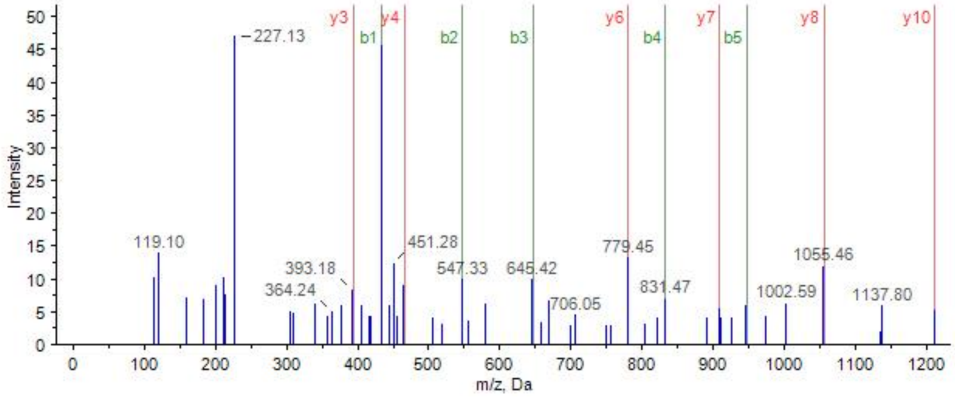

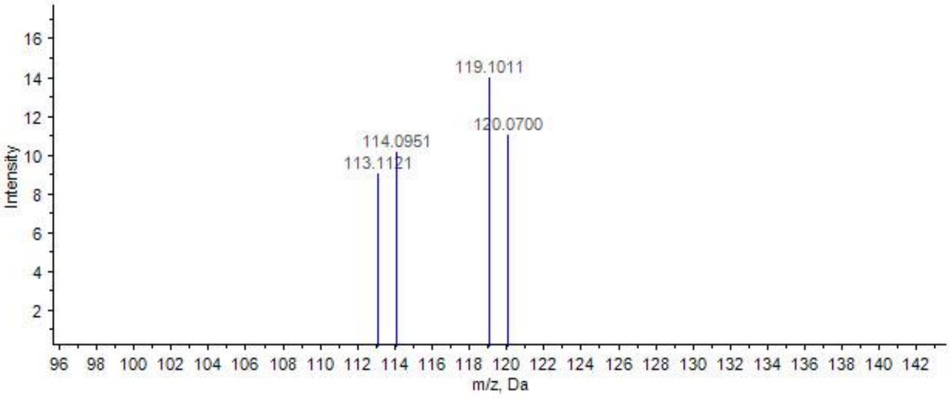

Aco2

Con...	Conf	Sequence
2.00	99	AGSALNR
2.00	99	DGYAQILR
2.00	99	DINQEVYNFLATAGAK
2.00	99	EGWPLDIR
2.00	99	EHAALEPR
2.00	99	FKLEAPDADELPR
2.00	99	FNPETDFLTGK
2.00	99	IVYGHLDDPANQEIER
2.00	99	LEAPDADELPR
2.00	99	NAVTQEFGPVPDTAR
2.00	99	NDANPETHAFVTSPEIVTA...
2.00	99	NTIVTSYNR
2.00	99	SQFTITPGSEQIR
2.00	99	TDIANLAEEFK
2.00	99	VAMQDATAQMAMLQFISSG...
2.00	99	VDVSPTSQR
2.00	99	WVVIGDENYGEGSSR

Peptide: FNPETDFLTGK[IT8]

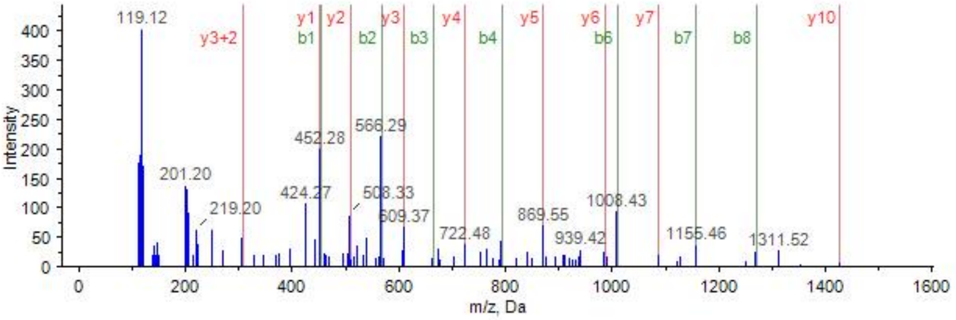

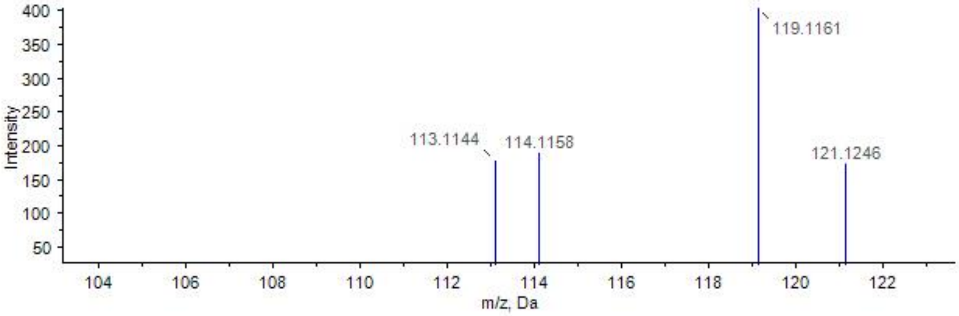

Idh1

Con...	Conf	Sequence
2.00	99	ATDFVVPGPGK
2.00	99	DATNDQVTK
2.00	99	GQETSTNPIASIFAWSR
2.00	99	GWPLYLSTK
2.00	99	LILPYVELDLHSYDLGIENR
2.00	99	LVTGWVKPIIIGR
2.00	99	NILGGTVFR
2.00	99	TVEAEAAHGTVTR

Peptide: ATDFVVPGPGK[IT8]

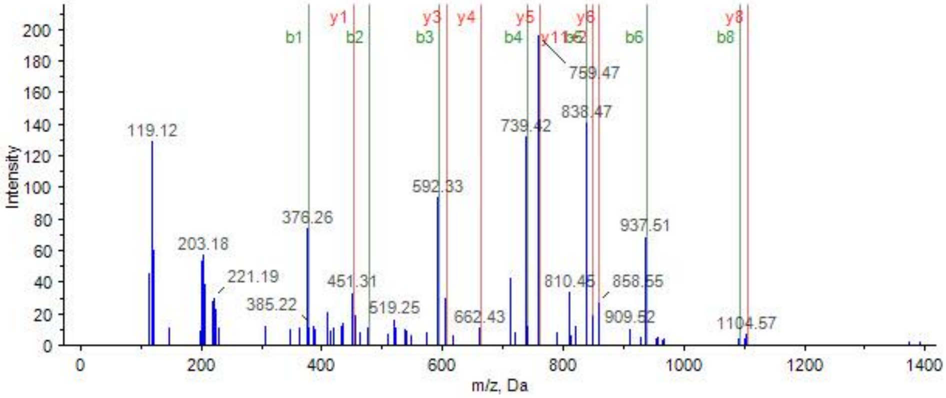

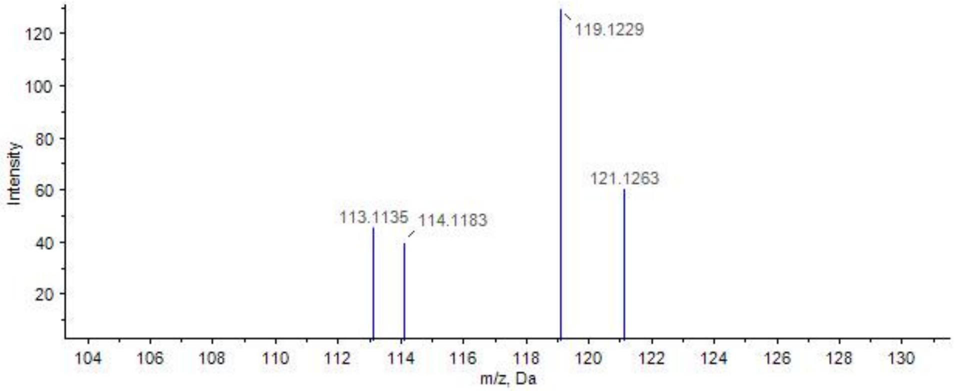

Calr

Peptide: EEEEAEDKEDDDDR

Con...	Conf	Sequence
2.00	99	DEDEDEEDEK
2.00	99	DKQDEEQR
2.00	99	EDDDDRDEDEDEEDEK
2.00	99	EEDEEESPGQAK
2.00	99	EEEEAEDK
2.00	99	EEEEAEDKEDDDDR
2.00	99	EQFLDGDAWTNR
2.00	99	FVLSSGK
2.00	99	FYALSAK
2.00	99	GLQTSQDAR
2.00	99	LFPSGLDQK
2.00	99	LKEEEEDK
2.00	99	QIDNPDYK
2.00	99	SGTIFDNFLITNDEAYAEE...

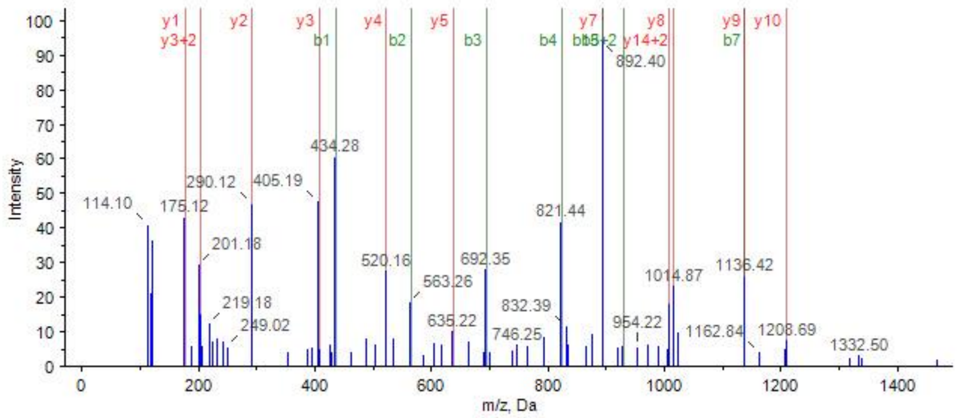

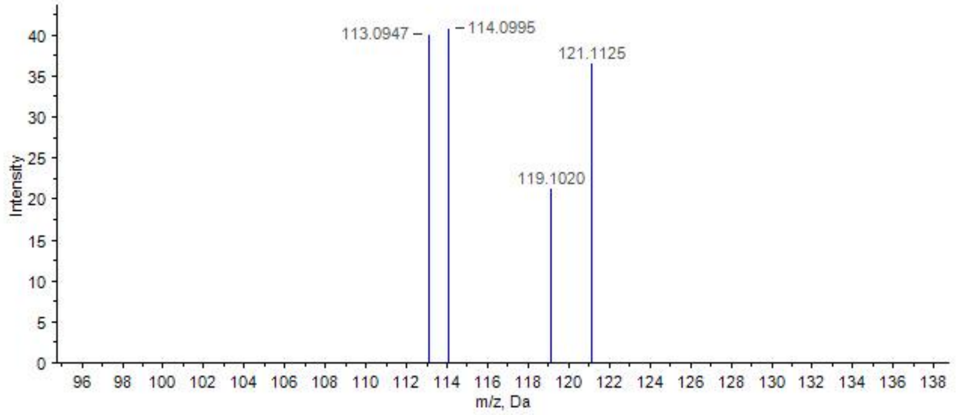

Ctnna1

Peptide: EELVVAVEDVR

Con...	Conf	Sequence
2.00	99	EELVVAVEDVR
2.00	99	FTEQVEAAVEALSSDPAQP...
2.00	99	LLSNTVMPR
2.00	99	QALQDLLSEYMGNAGR
2.00	99	TPEELDDSDFETEDFDVR
2.00	99	TSVQTEDDQLIAGQSAR

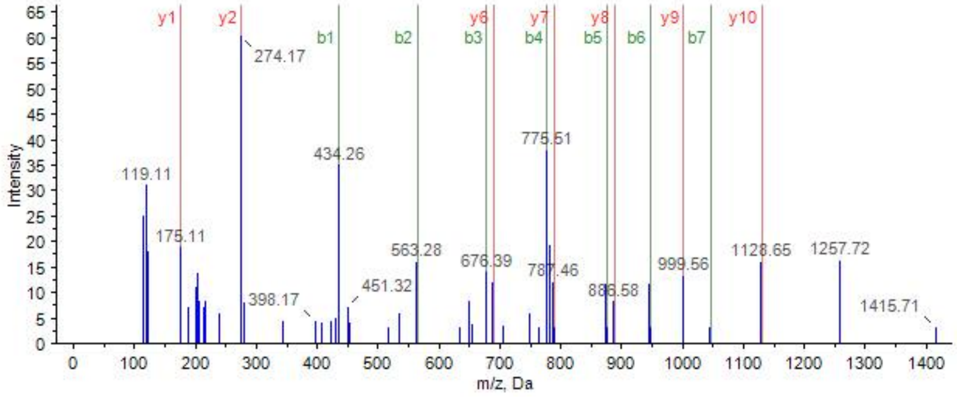

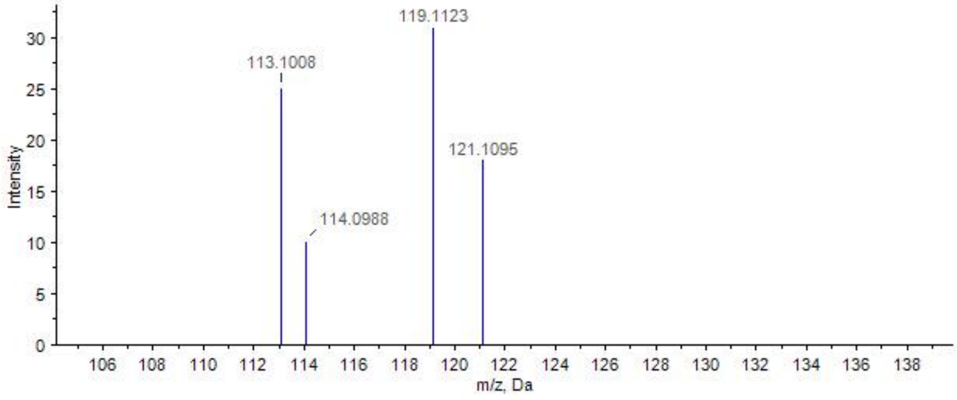

P4hb

Peptide: EADDIVNWLK[IT8]

Con...	Conf	Sequence
2.00	99	DATEESDLAQQYGVR
2.00	99	DVESDSAK
2.00	99	EADDIVNWLK
2.00	99	FFPASADR
2.00	99	IFGGEIK
2.00	99	ILEFFGLK
2.00	99	ILFIFIDSDHTDNQR
2.00	99	LITLEEEMTK
2.00	99	LLDFIK
2.00	99	MDSTANEVEAVK
2.00	99	NGDTASPK
2.00	99	QFLLAAEAIDDIPFGITSN...
2.00	99	SVSDYDGK
2.00	99	TGPAATTLSDTAAAESLVD...
2.00	99	TVIDYNGER
2.00	99	VDATEESDLAQQYGVR

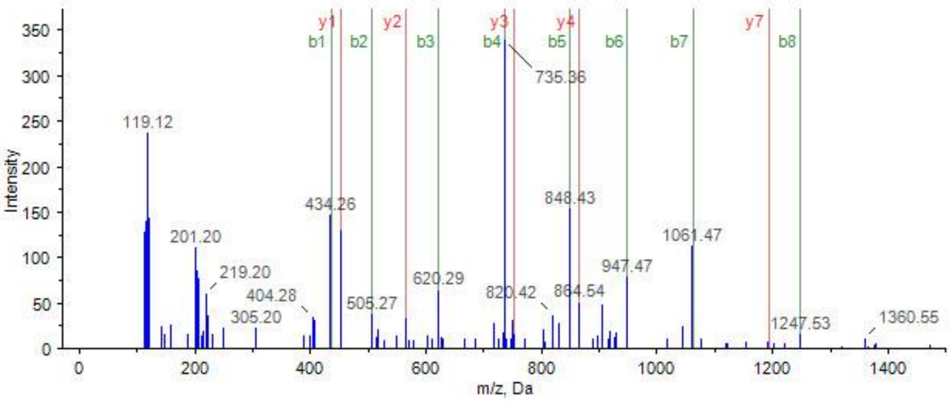

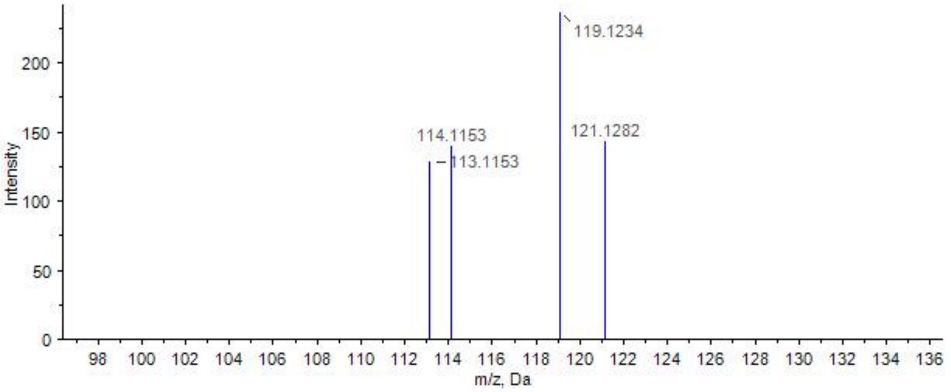

Table S1 Differential proteins identified in different experiment groups using iTRAQ–based quantitative proteomics analysis

Protein	Accession number	P value			Peptides (95%)	Regulation (ratio)			Function
		113:119	121:119	114:119		N+CHBP	IR	IR+CHBP	
Msi2	Q920Q6	0.0403	0.3885	0.8406	2	Down(0.30)	Down(0.64)	--	Binding
Cobll1	Q3UMF0	0.0879	0.0619	0.3264	8	--	Down (0.40)	--	Molecular function
Gatm	Q9D964	0.0010	0.0002	0.0008	9	Down (0.38)	Down (0.21)	Down (0.16)	Enzyme, regulator
Lamc1	P02468	3.21E-05	2.69E-06	1.51E-05	44	Up (3.63)	Up (4.41)	Up (4.02)	Binding, structural
Lamb1	P02469	0.0005	8.47E-05	0.0002	28	Up (2.19)	Up (2.65)	Up (2.15)	Binding, structural
Got2	P05202	0.0136	0.0157	0.0197	9	Down (0.44)	Down (0.52)	Down (0.36)	Binding, enzyme
Anxa5	P48036	0.5456	0.4422	0.2427	6	--	Up (1.57)	--	Binding
Slc9a3r1	P70441	0.0016	0.1006	0.0197	37	Up (1.60)	Down (0.36)	--	Binding, regulator, structural
Atp5j	P97450	0.0399	0.4554	0.0198	18	Up (1.96)	--	Up (1.82)	Enzyme, regulator
Hadhb	Q99JY0	0.0487	0.3532	0.0318	12	Down (0.53)	--	--	Binding, enzyme
Kng1	O08677	0.1759	0.0198	0.0453	4	--	Up (4.83)	Up (2.73)	Regulator
Hspg2	Q05793	0.2468	0.0104	0.4134	23	--	Up(1.94)	--	Binding
Saa2	P05367	0.2338	0.0235	0.1717	6	--	Up (16.29)	--	Binding
Idh1	O88844	0.0278	0.4667	0.1168	14	Down (0.41)	--	--	Binding, enzyme
Aldh4a1	Q8CHT0	0.0003	0.0015	0.0002	13	Down (0.43)	Down (0.52)	Down (0.40)	Binding, enzyme, regulator
Uqcrh	P99028	0.0394	0.2513	0.0767	28	Up (3.13)	Up (1.74)	Up (2.83)	Binding, enzyme
Prdx5	P99029	0.0023	0.0020	0.0100	29	Down (0.58)	Down (0.34)	Down (0.52)	Binding, enzyme, regulator
Pebp1	P70296	0.3378	0.0519	0.3863	14	--	Down (0.29)	--	Binding

Hpt	Q61646	0.4675	0.0077	0.0250	9	--	Up (31.33)	Up (19.95)	Binding regulator
Clta	O08585	0.0248	0.0707	0.0437	16	Up (2.27)	Up (1.64)	Up (2.15)	Structural
Pck1	Q9Z2V4	0.0057	0.2048	0.0040	8	Down (0.44)	Down (0.66)	Down (0.47)	Binding, enzyme
Tln1	P26039	0.0081	0.0368	0.0099	67	Up (1.84)	Up (1.61)	Up (1.58)	Binding, structural
Ctnna1	P26231	0.0499	0.9148	0.5555	11	Up(1.67)	--	--	Binding, structural
Sdhb	Q9CQA3	0.0070	0.0092	0.0017	7	Down (0.35)	Down (0.38)	Down (0.38)	Binding, enzyme, regulator
Gstm1	P10649	0.0180	0.0038	0.0007	16	Down (0.61)	Down (0.23)	Down (0.47)	Binding, enzyme, regulator
Aass	Q99K67	0.0248	0.4232	0.2783	6	Down (0.50)	--	--	Enzyme
Sod2	P09671	0.0408	0.0202	0.0407	4	Down (0.58)	Down (0.59)	Down (0.65)	Binding, enzyme
Hba	P01942	0.0276	0.0377	0.0195	59	Up (5.92)	Up (12.02)	Up (6.67)	Binding, regulator
Hspb1	P14602	0.1012	0.0302	0.1564	7	--	Up (4.61)	--	Binding, regulator
Selenbp1	P17563	0.0183	0.6660	0.0296	9	Down (0.44)	--	Down (0.43)	Binding
Sdpr	Q63918	0.5382	0.0399	0.4304	4	--	Down (0.26)	--	Binding
Hnrnpab	Q99020	0.0166	0.0962	0.0249	16	Up (1.67)	--	Up (1.69)	Binding, regulator
Aldoa	P05064	0.0389	0.3213	0.1453	14	Down (0.46)	Up (1.71)	--	Binding, enzyme
Slc27a2	O35488	0.0038	0.0720	0.3557	5	Down (0.61)	--	--	Binding, enzyme, regulator
Eno1	P17182	0.0053	0.1105	0.0124	38	Down (0.57)	Down (0.67)	--	Binding, enzyme
Ezr	P26040	0.2916	0.0030	0.1702	25	--	Up (2.21)	--	Binding
Msn	P26041	0.3578	9.84E-05	0.0126	24	--	Up (3.98)	Up (2.61)	Binding
Mdh2	P08249	0.0093	0.1641	0.1284	42	Down (0.64)	--	--	Binding, enzyme
Anxa2	P07356	0.1015	0.1465	0.8399	6	--	Up (2.49)	--	Binding, regulator
Atp6v1f	Q9D1K2	0.3358	0.0104	0.2345	4	--	Down (0.14)	--	Enzyme, regulator
Ttc36	Q8VBW8	0.5323	0.0108	0.9694	5	--	Down (0.26)	--	Molecular function

Apoa1	Q00623	0.0243	0.0001	0.0058	42	Up (2.65)	Up (4.70)	Up (3.13)	Binding, regulator
Bdh1	Q80XN0	0.0468	0.1166	0.0861	4	Down (0.41)	Down (0.36)	--	Binding, enzyme
Serpinh1	P19324	0.0920	0.1049	0.0498	12	--	Up (7.38)	Up (5.50)	Binding, regulator
Flnb	Q80X90	0.7919	0.0017	0.3289	16	--	Up (1.91)	--	Binding
Hspd1	P63038	0.0164	0.0167	0.8191	144	--	Down (0.51)	--	Binding
Fabp3	P11404	0.8774	0.0462	0.9240	13	--	Down (0.24)	--	Binding, regulator
Uqcrc1	Q9CZ13	0.0106	0.0237	0.0259	32	Down (0.65)	Down (0.62)	--	Binding, enzyme
Aco1	P28271	0.0173	0.0244	0.0131	8	Down (0.44)	Down (0.42)	Down (0.44)	Binding, enzyme
Ldhd	Q7TNG8	0.1432	0.0067	0.3122	28	--	Down (0.63)	--	Binding, enzyme
Calu	O35887	0.4645	0.0313	0.0037	7	--	Down (0.54)	Down (0.51)	Binding
Oxct1	Q9D0K2	0.0204	0.0326	0.0279	7	Down (0.36)	Down (0.56)	Down (0.60)	Binding, enzyme
Cltb	Q6IRU5	0.0156	0.1021	0.0147	13	Up (2.40)	Up (1.80)	Up (2.23)	Binding, structural
Smarce1	O54941	0.0455	0.0623	0.0628	3	Up (4.09)	Up (3.34)	Up (3.60)	Binding, enzyme
Plec	Q9QXS1	0.0349	1.00E-05	0.0027	10	Up (1.51)	Up (3.84)	Up (1.67)	Binding
Atp5b	P56480	0.0567	1.18E-06	0.0127	159	--	Down (0.37)	--	Binding, enzyme
Emilin1	Q99K41	0.0948	0.0057	0.0527	12	--	Up (5.20)	--	Binding, structural
Des	P31001	0.4489	0.0791	0.3842	13	--	Up (1.64)	--	Binding, structural
Hsp90ab1	P11499	0.0455	0.0222	0.0332	23	Down (0.47)	Down (0.66)	Down (0.63)	Binding, regulator
Cat	P24270	5.42E-06	2.64E-06	7.52E-06	22	Down (0.41)	Down (0.49)	Down (0.38)	Binding, enzyme, regulator
Hpx	Q91X72	0.0374	0.0002	0.0138	9	Up (2.38)	Up (11.91)	Up (3.87)	Binding, enzyme
Pabpc1	P29341	0.2546	0.1173	0.2480	9	--	Down (0.46)	--	Binding
Akr1a1	Q9JII6	0.0008	4.45E-05	0.0015	24	Down (0.35)	Down (0.43)	Down (0.52)	Enzyme
Hspa5	P20029	0.0220	0.0546	0.0978	48	Up (1.60)	Up (1.54)	--	Binding

Gpd1	P13707	0.0482	0.0666	0.0554	6	Down (0.49)	Down (0.53)	Down (0.39)	Binding, enzyme
Acadm	P45952	0.0007	0.1363	0.0010	21	Down (0.46)	--	Down (0.51)	Binding, enzyme
Idh2	P54071	0.0035	0.0113	0.0625	10	Down (0.42)	Down (0.51)	Down (0.40)	Binding, enzyme
Aldob	Q91Y97	0.0049	0.0077	0.0226	39	Down (0.51)	Down (0.61)	--	Binding, enzyme
Akap2	O54931	0.0227	0.0421	0.8678	9	Up (1.87)	Up (1.89)	--	Binding
Hadha	Q8BMS1	0.0475	0.0192	0.0149	26	--	Down (0.46)	Down (0.41)	Binding, enzyme
Aldh6a1	Q9EQ20	4.73E-06	2.18E-05	6.97E-05	25	Down (0.39)	Down (0.52)	Down (0.50)	Enzyme
Ivd	Q9JHI5	0.0100	0.0148	0.0044	10	--	--	Down (0.64)	Binding, enzyme
Cox5a	P12787	0.3729	0.2667	0.3383	27	--	Down (0.64)	--	Binding, enzyme
Apoa4	P06728	0.0363	0.0727	0.0180	9	Up (6.25)	Up (4.97)	Up (6.31)	Binding, regulator
Atp6v1b2	P62814	0.0388	0.6429	0.0111	13	Down (0.57)	--	Down (0.53)	Binding, enzyme
Atp1a1	Q8VDN2	1.27E-05	3.04E-05	6.15E-09	43	Down (0.51)	Down (0.52)	Down (0.12)	Binding, enzyme
Dbi	P31786	0.0032	0.0111	0.0020	8	Up (2.33)	Up (2.05)	Up (2.61)	Binding
Acss1	Q99NB1	0.0043	0.0402	0.0044	1	Down (0.60)	Down (0.56)	Down (0.62)	Binding, enzyme
Mdh1	P14152	0.0016	0.0014	0.0015	16	Down (0.47)	Down (0.34)	Down (0.36)	Binding, enzyme
Pgk1	P09411	0.0407	0.1974	0.0846	19	Down (0.61)	--	--	Binding, enzyme
Myh11	O08638	0.1411	0.0022	0.5618	33	--	Up (1.79)	--	Binding, regulator, structural
Cdh16	O88338	0.7363	0.0169	0.1819	15	--	Down (0.38)	--	Binding
Gsn	P13020	0.0834	0.0067	0.9938	5	--	Up (2.36)	--	Binding
Myh9	Q8VDD5	0.0925	0.0004	8.09E-05	117	--	Up (3.28)	Up (1.69)	Binding, enzyme, regulator
Acsm2	Q8K0L3	2.86E-06	1.38E-05	1.71E-05	34	Down (0.36)	Down (0.44)	Down (0.43)	Binding, enzyme
Gpx3	P46412	0.0369	0.2918	0.3330	3	Down (0.18)	--		Binding, enzyme
Atp6v1a	P50516	6.34E-05	0.0001	9.66E-07	18	Down (0.33)	Down (0.52)	Down (0.29)	Binding, enzyme

Nudt19	P11930	0.0158	0.1581	0.0567	5	Down (0.40)	Down (0.49)	Down (0.52)	Binding, enzyme
Mif	P34884	0.3444	0.0574	0.1317	20	--	Down (0.52)	--	Binding, enzyme, regulator
Flna	Q8BTM8	0.0889	9.25E-05	0.0286	23	--	Up (4.53)	Up (2.13)	Binding, regulator
Ass1	P16460	0.0003	0.0047	0.0011	21	Down (0.35)	Down (0.40)	Down (0.29)	Binding, enzyme
Fus	P56959	0.0015	0.0282	0.0074	7	Up (3.47)	Up (2.47)	Up (2.73)	Binding
Lrp2	A2ARV4	0.0627	0.0011	0.0669	27	--	Down (0.44)	--	Binding, regulator
Lmna	P48678	0.0111	5.87E-05	0.0217	26	Up (1.54)	Up (2.42)	Up (1.61)	Binding, structural
Fn1	P11276	0.0126	0.0036	0.0021	26	Up (2.75)	Up (13.30)	Up (5.45)	Binding, regulator
Hdgf	P51859	0.0160	0.0388	0.0156	7	Up (3.34)	Up (2.70)	Up (3.22)	Binding, regulator
Ttr	P07309	0.0871	0.0182	0.2763	10	--	Up (5.01)	--	Binding, regulator
Calr	P14211	0.6220	0.9081	0.3555	29	--	--	Down (0.65)	Binding
Mep1a	P28825	0.2471	0.0042	0.0450	22	--	Down (0.27)	Down (0.38)	Binding, enzyme
Krt18	P05784	0.5594	0.0326	0.1671	13	--	Up (2.36)	--	Binding, structural
Serpina3k	P07759	0.5604	0.0015	0.4422	23	--	Up (2.68)	--	Binding, regulator
Krt8	P11679	0.3345	3.20E-05	0.0970	19	--	Up (5.06)	--	Binding, structural
Gapdh	P16858	0.0009	0.0111	0.0024	26	Down (0.54)	--	Down (0.58)	Binding, enzyme
Vil1	Q62468	0.0031	0.0079	0.0743	29	Down (0.63)	--	--	Binding, regulator
Acy1	Q99JW2	0.0095	0.0020	0.0134	16	Down (0.38)	Down (0.20)	Down (0.51)	Binding, enzyme
Ehhadh	Q9DBM2	4.39E-05	0.0071	1.33E-05	18	Down (0.26)	--	Down (0.29)	Binding, enzyme
Vim	P20152	0.1433	4.40E-05	0.0074	46	--	Up (3.91)	Up (2.58)	Binding, structural
Rab11fip4	Q8BQP8	0.0037	0.0704	0.5031	1	Down (0.09)	Down (0.16)	--	Binding
Hbb-b1	P02088	0.0857	0.0063	0.0693	86	--	Up (7.45)	--	Binding, regulator
Hbb-b2	P02089	0.2149	0.0035	0.1720	69	--	Up (4.09)	--	Binding, regulator

Ech1	O35459	0.0285	0.1321	0.1599	6	Down (0.32)	Down (0.62)	--	Binding, enzyme
Calb1	P12658	0.0382	0.5323	0.1562	22	Up (1.58)	--	--	Binding
Fgb	Q8K0E8	0.7567	0.0016	0.1029	6	--	Up (5.25)	--	Binding
Glo1	Q9CPU0	0.6098	0.0081	0.9345	10	--	Down (0.60)	--	Binding, enzyme
Pc	Q05920	9.56E-07	0.0009	2.17E-05	43	Down (0.46)	Down (0.63)	Down (0.47)	Binding, enzyme
Cltc	Q68FD5	0.0363	0.2358	0.2455	5	Down (0.24)	Down (0.57)	--	Binding, structural
Acaa2	Q8BWT1	0.0372	0.0003	0.4983	29	--	Down (0.61)	--	Enzyme
Alb	P07724	0.6991	1.14E-09	0.0614	37	--	Up (2.40)	--	Binding
Hspe1	Q64433	0.0043	0.4189	0.0302	23	Up (2.47)	--	Up (2.07)	Binding
Phb	P67778	0.0214	0.0082	0.0526	30	--	Down (0.43)	--	Binding, regulator
Akap12	Q9WTQ5	0.0227	0.0006	0.0067	12	Up (3.08)	Up (7.38)	Up (3.37)	Binding, regulator
Pdzk1	Q9JIL4	0.0191	4.36E-08	0.0029	53	--	Down (0.21)	--	Binding
Mup1	P11588	0.0016	8.09E-06	0.0013	13	Up (4.61)	Up (15.42)	Up (5.35)	Binding, regulator
Hdlbp	Q8VDJ3	0.1896	0.3805	0.7429	10	--	Down (0.58)	--	Binding
P4hb	P09103	0.8517	0.1977	0.0191	33	--	--	Down (0.60)	Enzyme
Ndufs4	Q9CXZ1	0.1719	0.1015	0.6972	16	--	Down (0.39)	--	Enzyme
Clu	Q06890	0.1294	0.0005	0.0247	10	--	Up (6.61)	Up (3.10)	Binding, enzyme
Aldh1l1	Q8R0Y6	0.0030	0.0260	0.0073	8	Down (0.29)	Down (0.31)	Down (0.44)	Binding, enzyme
Ndufs6	P52503	0.0363	0.8914	0.0215	12	Up (1.67)	--	Up (1.60)	Molecular function
Abcd3	P55096	0.0030	0.3040	0.0155	2	Down (0.29)	--	Down (0.33)	Binding, enzyme
Amacr	O09174	0.0334	0.0323	0.2785	12	Down (0.61)	Down (0.41)	--	Binding, enzyme
Etfb	Q9DCW4	0.5360	0.0385	0.8484	22	--	Down (0.60)	--	Regulator
Scp2	P32020	0.5232	0.0240	0.5857	23	--	Down (0.49)	--	Binding, enzyme, regulator

Apoa2	P09813	0.0007	0.0039	0.0037	26	Up (6.03)	Up (5.50)	Up (5.50)	Binding, regulator
Aco2	Q99KI0	0.0226	0.0673	0.0063	35	Down (0.62)	--	Down (0.53)	Binding, enzyme
Tgm2	P21981	0.9050	0.0248	0.2232	6	--	Up (3.47)	--	Binding, enzyme
Acat2	Q8CAY6	0.5960	0.0515	0.1533	3	--	Up (2.03)	--	Enzyme
Serpina1b	P22599	0.7413	0.0186	0.1902	15	--	Up (1.87)	--	Binding, regulator
Cgnl1	Q6AW69	0.0251	0.7811	0.0016	11	Up (1.58)	--	Up (1.61)	Molecular function regulator

Binding including: DNA binding, RNA binding, protein binding, enzyme binding, fatty acid binding, pyridoxal phosphate binding, ATP binding, cell surface binding, ion binding, drug binding and toxin binding.

--indicating: 0.67 < ratio < 1.5, no significantly statistical difference

Table S2 Sequences of the primers

Gene name		Primer sequence
NDUFS6	Sense	GTGGAGCACCGCATCATA
	Antisense	CAAGGGACACCATTCACAGA
AASS	Sense	GCCACGGTTGAGTCTTATGT
	Antisense	AGGGTATGCTTCTCTGTTGATG
ABCD3	Sense	TCACAACAGGAGAAGGGTATTG
	Antisense	GGGTCGCTGAGGAACATAAA
GAPDH	Sense	TATGAGGAACCGCATCGCTG
	Antisense	TAGCATGAGTTGGCACCCACTG

Table S3 Proteomics data

N	Unused	Total	%Cov	%Cov(50)	%Cov(95)	Accession	Name	Species	Peptides(95%)	113:119	PVal 113:119	EF 113:119	LowerCI 113:119	UpperCI 113:119	114:119	PVal 114:119	EF 114:119	LowerCI 114:119	UpperCI 114:119	121:119	PVal 121:119	EF 121:119	LowerCI 121:119	UpperCI 121:119
1	148.88	148.88	60.4399979	46.7200011	39.320001	sp|P16546|SI	Spectrin alpha chain, brain OS=Mus musculus GN=Sptan1 PE=1 SV=4	MOUSE	127	0.99083197	0.37147221	1.01859105	0.972747207	1.009253025	0.99083197	0.67565018	1.01859105	0.972747207	1.009253025	0.96382898	0.02141551	1.01859105	0.946237087	0.981747925
2	103.39	103.39	50.0999987	34.9000007	30.8200002	sp|Q8VDD5|M	Myosin-9 OS=Mus musculus GN=Myh9 PE=1 SV=4	MOUSE	117	1.31825697	0.09248436	1.158777	1.137627006	1.499685049	1.69044101	8.09E-05	1.19124198	1.419057012	1.976969957	3.28095293	0.00041356	1.247383	2.630268097	4.207265854
3	86.86	86.86	97.7299988	88.8499975	79.2100012	sp|P56480|AT	ATP synthase subunit beta, mitochondrial OS=Mus musculus GN=Atp5b PE=1 SV=2	MOUSE	159	0.79432821	0.0567412	1.07646501	0.731139123	0.855066717	0.73113912	0.01274646	1.08642602	0.672976673	0.794328213	0.36643761	1.18E-06	1.12719798	0.328095287	0.413047493
4	84.17	84.38	88.3099973	79.5799971	75.0400007	sp|P63038|CH	60 kDa heat shock protein, mitochondrial OS=Mus musculus GN=Hspd1 PE=1 SV=1	MOUSE	144	1.36772895	0.01636689	1.12719798	1.213389039	1.51356101	1.04712904	0.81909621	1.08642602	0.963828981	1.14815402	0.51050502	0.01672303	1.25892496	0.428548515	0.642687678
5	83.64	83.82	47.9299992	28.9999992	25.6999999	sp|P26039|TL	Talin-1 OS=Mus musculus GN=Tln1 PE=1 SV=2	MOUSE	67	1.83653796	0.00806666	1.19124198	1.541700006	2.187762022	1.58489299	0.00989098	1.20226395	1.318256974	1.940886021	1.61435902	0.03676244	1.20226395	1.342764974	1.958845019
6	77.73	77.73	77.0299971	65.1000023	61.8399978	sp|Q03265|AT	ATP synthase subunit alpha, mitochondrial OS=Mus musculus GN=Atp5a1 PE=1 SV=1	MOUSE	117	0.7798301	0.151215	1.08642602	0.724435985	0.847227395	0.95499259	0.78002048	1.08642602	0.88715601	1.037528038	0.80909592	0.50634903	1.07646501	0.744732022	0.870963573
7	70.25	70.25	84.3699992	70.1200008	63.7799978	sp|P63017|HS	Heat shock cognate 71 kDa protein OS=Mus musculus GN=Hspa8 PE=1 SV=1	MOUSE	61	1.31825697	0.1706132	1.13762701	1.158776999	1.527565956	1.30617094	0.1382765	1.14815402	1.137627006	1.485936046	1.07646501	0.25632832	1.13762701	0.946237087	1.224616051
8	67.01	68.93	82.7700019	60.680002	56.5500021	sp|P38647|GI	Stress-70 protein, mitochondrial OS=Mus musculus GN=Hspa9 PE=1 SV=2	MOUSE	79	1.31825697	0.01590598	1.11686301	1.180320978	1.485936046	1.19124198	0.79746741	1.10662401	1.076465011	1.318256974	0.69183099	0.08416687	1.11686301	0.625172675	0.772680581
9	61.79	62.02	35.3399992	17.7699998	14.26	sp|Q62261|SI	Spectrin beta chain, brain 1 OS=Mus musculus GN=Sptbn1 PE=1 SV=2	MOUSE	37	0.97274721	0.44516599	1.158777	0.839460015	1.127197981	1.01859105	0.6357289	1.158777	0.879022479	1.180320978	1.00925303	0.3514207	1.158777	0.870963573	1.16949904
10	61.5	61.79	56.9599986	35.74	32.2600007	sp|Q05920|PY	Pyruvate carboxylase, mitochondrial OS=Mus musculus GN=Pc PE=1 SV=1	MOUSE	43	0.4613176	9.56E-07	1.21338904	0.380189389	0.55975759	0.46989411	2.17E-05	1.19124198	0.390840888	0.55975759	0.62517268	0.00088779	1.18032098	0.529663384	0.73790431
11	61.04	61.04	72.4500001	66.2800014	62.0400012	sp|Q9JIL4|NH	Na(+)/H(+) exchange regulatory cofactor NHE-RF3 OS=Mus musculus GN=Pdzk1 PE=1 SV=1	MOUSE	53	0.79432821	0.01911968	1.11686301	0.711213529	0.88715601	0.77268058	0.00290554	1.11686301	0.691830993	0.862978518	0.214783	4.36E-08	1.27057397	0.149968505	0.27289781
12	53.98	53.99	77.8999984	61.3699973	59.4399989	sp|P20152|VI	Vimentin OS=Mus musculus GN=Vim PE=1 SV=3	MOUSE	46	2.03235698	0.1432565	1.29419601	1.570363045	2.964831114	2.58225989	0.00743703	1.35518897	1.905460954	3.80189395	3.90840912	4.40E-05	1.39315701	2.805433989	5.495409012
13	52.28	52.28	89.0699983	84.799999	76.8000007	sp|P60710|AC	Actin, cytoplasmic 1 OS=Mus musculus GN=Actb PE=1 SV=1	MOUSE	83															
14	50.83	50.94	57.04	36.7700011	33.7700009	sp|Q64727|VI	Vinculin OS=Mus musculus GN=Vcl PE=1 SV=4	MOUSE	42	1.72186899	0.06735776	1.18032098	1.458814025	2.070141077	1.77010906	0.03462567	1.20226395	1.472311974	2.208004951	1.65958703	0.07452098	1.18032098	1.406048059	1.995262027
15	46.83	46.83	46.8199998	26.6900003	25.3199995	sp|Q8VDN2|A	Sodium/potassium-transporting ATPase subunit alpha-1 OS=Mus musculus GN=Atp1a1 PE=1 SV=1	MOUSE	43	0.50582469	1.27E-05	1.13762701	0.416869402	0.57543987	0.1235947	6.15E-09	1.55596602	0.073790424	0.192309201	0.51999599	3.04E-05	1.158777	0.416869402	0.602559626
16	45.3	45.49	63.5599971	55.4499984	54.2599976	sp|P27773|PD	Protein disulfide-isomerase A3 OS=Mus musculus GN=Pdia3 PE=1 SV=2	MOUSE	43	0.95499259	0.26568741	1.158777	0.824138105	1.106624007	0.7177943	0.02153185	1.16949904	0.613762021	0.839460015	0.83176369	0.07661135	1.18032098	0.711213529	0.981747925
17	43.85	43.85	99.3200004	94.5599973	94.5599973	sp|P02088|HB	Hemoglobin subunit beta-1 OS=Mus musculus GN=Hbb-b1 PE=1 SV=2	MOUSE	86	3.73250198	0.08571038	1.64437199	2.269865036	4.920394897	2.83139205	0.06925754	1.36772895	2.070141077	3.53183198	7.44731998	0.0063132	1.599558	4.655860901	11.58777046
18	42.95	49.49	63.8199985	50.0800014	44.5800006	sp|P20029|GI	78 kDa glucose-regulated protein OS=Mus musculus GN=Hspa5 PE=1 SV=3	MOUSE	48	1.599558	0.0219557	1.19124198	1.342764974	1.923092008	1.58489299	0.09779444	1.18032098	1.342764974	1.905460954	1.54170001	0.05458778	1.18032098	1.30617094	1.819700956
19	42.88	42.99	52.3599982	34.5800012	31.6799998	sp|Q62468|VI	Villin-1 OS=Mus musculus GN=Vil1 PE=1 SV=3	MOUSE	29	0.62517268	0.00314632	1.19124198	0.48305881	0.744732022	0.76559663	0.07425024	1.16949904	0.660693526	0.895364821	0.79432821	0.00786849	1.158777	0.685488224	0.920449615
20	41.68	41.68	30.5599988	15.5399993	13.3200005	sp|P11276|FI	Fibronectin OS=Mus musculus GN=Fn1 PE=1 SV=3	MOUSE	26	2.75422907	0.01256511	1.29419601	2.128139019	3.698282003	5.44502592	0.00208097	1.73780096	3.133285999	9.375619888	13.3045397	0.00364136	1.78648806	7.447319984	22.69865036
21	40.27	40.27	84.7899973	74.6500015	70.4200029	sp|P70441|NH	Na(+)/H(+) exchange regulatory cofactor NHE-RF1 OS=Mus musculus GN=Slc9a3r1 PE=1 SV=3	MOUSE	37	1.599558	0.00158922	1.18032098	1.355188966	2.013724089	1.36772895	0.01969134	1.14815402	1.19124198	1.721868992	0.35645109	0.1005836	1.38038397	0.267916799	0.492039502
22	39.48	39.63	48.0300009	35.3599995	34.0499997	sp|P07724|AL	Serum albumin OS=Mus musculus GN=Alb PE=1 SV=3	MOUSE	37	0.85506672	0.69912797	1.12719798	0.765596628	0.963828981	0.82413811	0.06139456	1.13762701	0.73790431	0.937561989	2.39883304	1.14E-09	1.18032098	2.032356977	2.992264986
23	39.37	39.37	23.3899996	8.86299983	7.27500022	sp|A2ARV4|L	Low-density lipoprotein receptor-related protein 2 OS=Mus musculus GN=Lrp2 PE=1 SV=1	MOUSE	27	0.83946002	0.06265821	1.247383	0.666806817	1.047129035	0.66680682	0.06688844	1.33045399	0.524807513	0.88715601	0.44055489	0.00110904	1.38038397	0.288403213	0.608134985
24	39.22	39.39	35.7199997	19.5999995	18.4799999	sp|P02468|LA	Laminin subunit gamma-1 OS=Mus musculus GN=Lamc1 PE=1 SV=2	MOUSE	44	3.63077998	3.21E-05	1.52756596	2.376840115	5.445025921	4.0179081	1.51E-05	1.55596602	2.582259893	6.137619972	4.40554905	2.69E-06	1.52756596	2.884032011	6.486343861
25	39.2	39.3	48.0100006	36.0500008	31.7200005	sp|P26041|MO	Moesin OS=Mus musculus GN=Msn PE=1 SV=3	MOUSE	24	1.75388098	0.35777599	1.43218803	1.224616051	2.630268097	2.60615301	0.01264112	1.64437199	1.584892988	4.786301136	3.98107195	9.84E-05	1.73780096	2.290868044	7.379042149
26	38.82	38.82	77.4699986	63.4599984	58.5200012	sp|Q91Y97|A	Fructose-bisphosphate aldolase B OS=Mus musculus GN=Aldob PE=1 SV=3	MOUSE	39	0.51050502	0.0049005	1.19124198	0.405508488	0.608134985	0.67920363	0.02256067	1.18032098	0.570164323	0.801678121	0.60813499	0.00765397	1.20226395	0.478630096	0.731139123
27	38.76	38.76	74.5000005	65.7199979	64.3100023	sp|O88569|RO	Heterogeneous nuclear ribonucleoproteins A2/B1 OS=Mus musculus GN=Hnrnpa2b1 PE=1 SV=2	MOUSE	31	1.39315701	0.1835248	1.19124198	1.16949904	1.706081986	0.98174793	0.80323482	1.18032098	0.839460015	1.158776999	0.68548822	0.1389682	1.19124198	0.544502676	0.816582382
28	38.71	38.84	79.0300012	61.7500007	58.9900017	sp|P17182|EN	Alpha-enolase OS=Mus musculus GN=Eno1 PE=1 SV=3	MOUSE	38	0.57016432	0.00528639	1.18032098	0.461317599	0.672976673	0.67920363	0.01240376	1.16949904	0.586138189	0.794328213	0.66680682	0.1105092	1.16949904	0.57543987	0.779830098
29	38.55	38.55	99.2999971	95.7700014	95.7700014	sp|P01942|HB	Hemoglobin subunit alpha OS=Mus musculus GN=Hba PE=1 SV=2	MOUSE	59	5.91561699	0.02759403	1.62929595	3.630779982	10.76465034	6.66806698	0.01954766	1.599558	4.168694019	11.58777046	12.0226402	0.03772434	1.64437199	7.311390877	22.28434944
30	38.31	38.31	60.5199993	45.0399995	38.7800008	sp|Q8K0L3|A	Acyl-coenzyme A synthetase ACSM2, mitochondrial OS=Mus musculus GN=Acsm2 PE=2 SV=1	MOUSE	34	0.35645109	2.86E-06	1.36772895	0.235504895	0.487528503	0.4325138	1.71E-05	1.25892496	0.283139199	0.544502676	0.44463131	1.38E-05	1.27057397	0.299226493	0.564936996
31	38.04	38.05	64.4999981	55.9199989	52.0699978	sp|P08249|MI	Malate dehydrogenase, mitochondrial OS=Mus musculus GN=Mdh2 PE=1 SV=3	MOUSE	42	0.63679552	0.00929278	1.19124198	0.529663384	0.758577585	0.80909592	0.1283934	1.14815402	0.691830993	0.928966403	0.72443599	0.164069	1.16949904	0.613762021	0.847227395
32	37.58	46.36	41.6299999	16.6800007	14.4500002	sp|O08638|M	Myosin-11 OS=Mus musculus GN=Myh11 PE=1 SV=1	MOUSE	33	1.21338904	0.1410591	1.21338904	1	1.499685049	0.96382898	0.56180102	1.30617094	0.73790431	1.258924961	1.78648806	0.00216835	1.247383	1.432188034	2.333457947
33	37.07	37.07	60.9099984	36.8999988	27.4300009	sp|Q9D0E1|H	Heterogeneous nuclear ribonucleoprotein M OS=Mus musculus GN=Hnrnpm PE=1 SV=3	MOUSE	20	1.21338904	0.57280397	1.19124198	1.018591046	1.458814025	1.28233099	0.00445811	1.21338904	1.056818008	1.55596602	1.09647799	0.385721	1.19124198	0.920449615	1.318256974
34	36.81	37.66	60.5099976	49.7099996	37.9200012	sp|P09103|PD	Protein disulfide-isomerase OS=Mus musculus GN=P4hb PE=1 SV=2	MOUSE	33	1.09647799	0.85167038	1.18032098	0.928966403	1.29419601	0.59703529	0.01910167	1.20226395	0.452897608	0.717794299	0.7798301	0.1977305	1.18032098	0.636795521	0.920449615
35	36.11	36.2	68.7900007	46.360001	38.319999	sp|Q9EQ20|M	Methylmalonate-semialdehyde dehydrogenase [acylating], mitochondrial OS=Mus musculus GN=Aldh6a1 PE=	MOUSE	25	0.39084089	4.73E-06	1.25892496	0.277971298	0.492039502	0.49659231	6.97E-05	1.21338904	0.401790798	0.602559626	0.51522863	2.18E-05	1.20226395	0.416869402	0.619441092
36	35.83	35.94	47.6900011	31.9200009	28.5899997	sp|Q99KI0|AC	Aconitate hydratase, mitochondrial OS=Mus musculus GN=Aco2 PE=1 SV=1	MOUSE	35	0.61944109	0.02260534	1.16949904	0.505824685	0.724435985	0.52966338	0.00625925	1.20226395	0.413047493	0.636795521	0.66680682	0.06733625	1.158777	0.57543987	0.772680581
37	35.29	35.3	62.529999	40.0700003	30.2700013	sp|Q01853|TE	Transitional endoplasmic reticulum ATPase OS=Mus musculus GN=Vcp PE=1 SV=4	MOUSE	27	0.51999599	0.1916129	1.31825697	0.340408206	0.685488224	0.88715601	0.6974628	1.27057397	0.698232412	1.127197981	1.04712904	0.474345	1.27057397	0.824138105	1.330453992
38	34.67	34.69	67.2200024	32.3300004	29.4699997	sp|P48678|LM	Prelamin-A/C OS=Mus musculus GN=Lmna PE=1 SV=2	MOUSE	26	1.54170001	0.01109692	1.27057397	1.213389039	2.128139019	1.61435902	0.02168971	1.28233099	1.258924961	2.269865036	2.42102909	5.87E-05	1.31825697	1.836537957	3.630779982
39	34.37	34.37	53.7100017	46.0700005	45.1700002	sp|P68372|TE	Tubulin beta-4B chain OS=Mus musculus GN=Tubb4b PE=1 SV=1	MOUSE	42	0.89536482	0.3038052	1.22461605	0.724435985	1.096477985	0.97274721	0.78882217	1.247383	0.765596628	1.213389039	1.09647799	0.79422772	1.12719798	0.972747207	1.235947013
40	34.18	34.65	27.7700007	12.37	10.8599998	sp|P02469|LA	Laminin subunit beta-1 OS=Mus musculus GN=Lamb1 PE=1 SV=3	MOUSE	28	2.18776202	0.00051437	1.21338904	1.803017974	2.83139205	2.14783001	0.00019152	1.22461605	1.753880978	2.83139205	2.6546061	8.47E-05	1.22461605	2.167704105	3.46736908
41	34	34.2	86.1500025	68.4599996	61.5400016	sp|Q8BFU2|H	Histone H2A type 3 OS=Mus musculus GN=Hist3h2a PE=1 SV=3	MOUSE	27															
41	0	34.2	86.1500025	68.4599996	61.5400016	sp|P22752|H2	Histone H2A type 1 OS=Mus musculus GN=Hist1h2ab PE=1 SV=3	MOUSE	27															
42	33.75	33.75	64.0900016	48.3200014	47.9900003	sp|P51881|AD	ADP/ATP translocase 2 OS=Mus musculus GN=Slc25a5 PE=1 SV=3	MOUSE	25	0.87902248	0.4827551	1.55596602	0.580764413	1.367728949	0.95499259	0.60094011	1.54170001	0.625172675	1.472311974	0.87096357	0.42682719	1.54170001	0.570164323	1.342764974
43	33.17	33.17	65.9099996	50.8300006	47.7299988	sp|Q7TNG8|L	Probable D-lactate dehydrogenase, mitochondrial OS=Mus musculus GN=Ldhd PE=1 SV=1	MOUSE	28	0.80909592	0.14318501	1.14815402	0.704693079	0.928966403	0.88715601	0.31223449	1.14815402	0.772680581	1.018591046	0.63095743	0.00670648	1.18032098	0.539510608	0.744732022
44	31.93	31.94	49.8899996	29.9299985	26.2100011	sp|P57780|AC	Alpha-actinin-4 OS=Mus musculus GN=Actn4 PE=1 SV=1	MOUSE	25	0.69823241	0.2190221	1.22461605	0.515228629	0.855066717	0.73113912	0.06594732	1.21338904	0.608134985	0.88715601	1.30617094	0.12574551	1.18032098	1.106624007	1.541700006
45	31.85	32.46	25.8300006	11.7200002	8.22400004	sp|Q80X90|FI	Filamin-B OS=Mus musculus GN=Flnb PE=1 SV=3	MOUSE	16	1.04712904	0.79188412	1.30617094	0.801678121	1.367728949	1.33045399	0.32888401	1.31825697	1.009253025	1.737800956	1.90546095	0.00173288	1.33045399	1.432188034	2.606153011
46	31.69	31.87	81.8199992	69.7000027	62.1200025	sp|Q00623|AI	Apolipoprotein A-I OS=Mus musculus GN=Apoa1 PE=1 SV=1	MOUSE	42	2.6546061	0.02434563	1.247383	2.128139019	3.597492933	3.133286	0.00575497	1.29419601	2.421029091	4.446312904	4.69894123	0.00013043	1.34276497	3.499452114	6.854881763
47	31.61	31.93	21.0999996	7.52599984	6.31200001	sp|Q05793|PG	Basement membrane-specific heparan sulfate proteoglycan core protein OS=Mus musculus GN=Hspg2 PE=1	MOUSE	23	1.16949904	0.24680351	1.25892496	0.928966403	1.55596602	0.95499259	0.41343519	1.31825697	0.731139123	1.258924961	1.94088602	0.0103559	1.34276497	1.445440054	2.754229069
48	31.57	31.84	40.0000006	13.8899997	10.8400002	sp|Q99PL5|R	Ribosome-binding protein 1 OS=Mus musculus GN=Rrbp1 PE=2 SV=2	MOUSE	16	1.43218803	0.1203125	1.23594701	1.158776999	1.753880978	1.62929595	0.01778673	1.22461605	1.330453992	1.976969957	1.54170001	0.04227196	1.22461605	1.258924961	1.870682001
49	31.32	31.55	52.640003	39.4199997	37.7400011	sp|P14211|CA	Calreticulin OS=Mus musculus GN=Calr PE=1 SV=1	MOUSE	29	1.14815402	0.62202448	1.20226395	0.954992592	1.380383968	0.65463609	0.35545349	1.28233099	0.501187205	0.839460015	0.94623709	0.90814853	1.21338904	0.787045777	1.14815402
50	31.24	33.02	61.8200004	31.310001	26.1599988	sp|Q8CAQ8|I	Mitochondrial inner membrane protein OS=Mus musculus GN=Immt PE=1 SV=1	MOUSE	22	0.87902248	0.59361529	1.20226395	0.704693079	1.056818008	1.10662401	0.60658407	1.16949904	0.946237087	1.355188966	1.02801597	0.89580798	1.22461605	0.839460015	1.270573974
51	30.77	30.77	47.8599995	24.0600005	20.3199998	sp|Q3U0V1|F	Far upstream element-binding protein 2 OS=Mus musculus GN=Khsrp PE=1 SV=2	MOUSE	17	1.99526203	0.0525831	1.29419601	1.541700006	2.805433989	2.051162	0.04822623	1.30617094	1.570363045	2.91071701	1.54170001	0.28389001	1.28233099	1.202263951	2.147830009
52	29.96	29.96	75.3099978	56.6799998	56.6799998	sp|Q8BWT1|T	3-ketoacyl-CoA thiolase, mitochondrial OS=Mus musculus GN=Acaa2 PE=1 SV=3	MOUSE	29	0.81658238	0.03717378	1.16949904	0.691830993	0.954992592	0.89536482	0.49826649	1.158777	0.765596628	1.037528038	0.61376202	0.00034226	1.22461605	0.501187205	0.751622915
53	29.65	29.66	56.9299996	40.7999992	37.0000005	sp|P24270|CA	Catalase OS=Mus musculus GN=Cat PE=1 SV=4	MOUSE	22	0.41304749	5.42E-06	1.25892496	0.208929598	0.519995987	0.38370732	7.52E-06	1.30617094	0.1786488	0.501187205	0.4875285	2.64E-06	1.21338904	0.296483099	0.591561615
54	29.1	29.37	68.3099985	50	40.4900014	sp|P21107|TP	Tropomyosin alpha-3 chain OS=Mus musculus GN=Tpm3 PE=1 SV=2	MOUSE	24	0.91201079	0.95272398	1.29419601	0.698232412	1.180320978	1.19124198	0.47171161	1.30617094	0.912010789	1.541700006	0.9289664	0.59262472	1.28233099	0.731139123	1.19124198
55	28.93	28.93	76.5999973	57.1699977	54.2999983	sp|Q9DB77|Q	Cytochrome b-c1 complex subunit 2, mitochondrial OS=Mus musculus GN=Uqcrc2 PE=1 SV=1	MOUSE	24	0.73790431	0.45683509	1.25892496	0.591561615	0.928966403	0.89536482	0.94355339	1.22461605	0.724435985	1.096477985	0.87902248	0.97017032	1.247383	0.711213529	1.096477985
56	28.61	28.8	43.1899995	25.7200003	24.21	sp|Q91VD9|N	NADH-ubiquinone oxidoreductase 75 kDa subunit, mitochondrial OS=Mus musculus GN=Ndufs1 PE=1 SV=2	MOUSE	21	0.67920363	0.1323141	1.21338904	0.534564376	0.824138105	0.73790431	0.1000125	1.20226395	0.608134985	0.88715601	0.60813499	0.05422737	1.23594701	0.48305881	0.751622915
57	28.38	29.06	41.6799992	28.4399986	23.9800006	sp|Q8BMS1|E	Trifunctional enzyme subunit alpha, mitochondrial OS=Mus musculus GN=Hadha PE=1 SV=1	MOUSE	26	0.66680682	0.04744983	1.28233099	0.510505021	0.855066717	0.40550849	0.01487237	1.39315701	0.288403213	0.564936996	0.4570882	0.01915492	1.39315701	0.304789513	0.636795521
58	28.18	28.18	61.5400016	56.6200018	51.9999981	sp|Q9JII6|AK	Alcohol dehydrogenase [NADP+] OS=Mus musculus GN=Akr1a1 PE=1 SV=3	MOUSE	24	0.34673679	0.00081601	1.33045399	0.258226007	0.461317599	0.52480751	0.00153095	1.23594701	0.416869402	0.648634374	0.4168694	4.45E-05	1.33045399	0.307609707	0.55462569
59	28.01	28.09	66.2699997	55.1900029	42.2199994	sp|Q8QZT1|T	Acetyl-CoA acetyltransferase, mitochondrial OS=Mus musculus GN=Acat1 PE=1 SV=1	MOUSE	68	1.00925303	0.9356817	1.12719798	0.895364821	1.14815402	0.9289664	0.95017427	1.13762701	0.816582382	1.056818008	0.93756199	0.93442053	1.13762701	0.824138105	1.066596031
60	27.6	27.6	55.0300002	43.0000007	29.9800009	sp|P97807|FU	Fumarate hydratase, mitochondrial OS=Mus musculus GN=Fh PE=1 SV=3	MOUSE	28	0.73113912	0.151631	1.22461605	0.580764413	0.895364821	0.77268058	0.00772502	1.20226395	0.630957425	0.928966403	0.74473202	0.01754782	1.23594701	0.597035289	0.920449615
61	27.5	30.86	28.0699998	11.4500001	11.1100003	sp|Q8BTM8|F	Filamin-A OS=Mus musculus GN=Flna PE=1 SV=5	MOUSE	23	1.99526203	0.08885442	1.51356101	1.318256974	3.221069098	2.12813902	0.02855814	1.43218803	1.485936046	3.133285999	4.52897596	9.25E-05	1.57036305	2.884032011	9.908319473
62	27.19	27.19	63.2600009	35.5599999	32.22	sp|O08749|DI	Dihydrolipoyl dehydrogenase, mitochondrial OS=Mus musculus GN=Dld PE=1 SV=2	MOUSE	25	0.87902248	0.73034018	1.19124198	0.73790431	1.047129035	1	0.98198462	1.19124198	0.839460015	1.202263951	0.70469308	0.1742792	1.19124198	0.580764413	0.839460015
63	27.05	27.08	76.0999978	64.3400013	56.25	sp|P67778|PH	Prohibitin OS=Mus musculus GN=Phb PE=1 SV=1	MOUSE	30	0.7177943	0.02136748	1.21338904	0.591561615	0.870963573	0.69183099	0.05259373	1.21338904	0.564936996	0.839460015	0.4325138	0.008164	1.28233099	0.340408206	0.55462569
64	26.58	26.58	58.130002	41.2499994	39.7899985	sp|Q9CZ13|Q	Cytochrome b-c1 complex subunit 1, mitochondrial OS=Mus musculus GN=Uqcrc1 PE=1 SV=2	MOUSE	32	0.64863437	0.01058597	1.22461605	0.534564376	0.794328213	0.73113912	0.02587202	1.158777	0.613762021	0.847227395	0.61944109	0.02366034	1.21338904	0.492039502	0.751622915
65	26.42	45.77	38.9699996	17.1599999	13.8699994	sp|Q61879|M	Myosin-10 OS=Mus musculus GN=Myh10 PE=1 SV=2	MOUSE	26	0.85506672	0.55813748	1.35518897	0.619441092	1.158776999	0.94623709	0.72756141	1.22461605	0.765596628	1.158776999	1.21338904	0.14016239	1.28233099	0.946237087	1.570363045
66	26.13	26.25	43.0799991	21.6700003	19.0599993	sp|P98078|DA	Disabled homolog 2 OS=Mus musculus GN=Dab2 PE=1 SV=2	MOUSE	16	1.33045399	0.30594599	1.22461605	1.08642602	1.629295945	1.28233099	0.42762339	1.21338904	1.056818008	1.570363045	0.67920363	0.69817102	1.21338904	0.564936996	0.824138105
67	26.09	26.09	53.3200026	31.7699999	26.7399997	sp|P50516|VA	V-type proton ATPase catalytic subunit A OS=Mus musculus GN=Atp6v1a PE=1 SV=2	MOUSE	18	0.3311311	6.34E-05	1.36772895	0.224905506	0.452897608	0.28840321	9.66E-07	1.36772895	0.157036304	0.39445731	0.51999599	0.00011184	1.22461605	0.405508488	0.636795521
68	25.86	26.1	37.6199991	25.1800001	23.1999993	sp|P09405|NU	Nucleolin OS=Mus musculus GN=Ncl PE=1 SV=2	MOUSE	22	0.99083197	0.65139133	1.28233099	0.787045777	1.270573974	1.18032098	0.3238427	1.27057397	0.928966403	1.690441012	1.43218803	0.1700608	1.29419601	1.106624007	2.128139019
69	25.67	25.83	40.0599986	28.7299991	24.3100002	sp|P11499|HS	Heat shock protein HSP 90-beta OS=Mus musculus GN=Hsp90ab1 PE=1 SV=3	MOUSE	23	0.474242	0.04551698	1.30617094	0.363078088	0.619441092	0.63095743	0.03315151	1.30617094	0.48305881	0.824138105	0.66069353	0.02219816	1.30617094	0.505824685	0.862978518
70	25.53	25.55	23.0199993	6.26699999	4.76099998	sp|Q61001|LA	Laminin subunit alpha-5 OS=Mus musculus GN=Lama5 PE=1 SV=4	MOUSE	13	1.77010906	0.90993381	1.35518897	1.30617094	2.421029091	1.52756596	0.121257	1.36772895	1.116863012	2.089296103	2.72897792	0.06172915	1.40604806	1.940886021	3.872575998
71	25.3	25.3	89.6300018	66.4600015	52.4399996	sp|P17742|PP	Peptidyl-prolyl cis-trans isomerase A OS=Mus musculus GN=Ppia PE=1 SV=2	MOUSE	40	1.13762701	0.42932081	1.158777	0.981747925	1.330453992	1.11686301	0.52670163	1.158777	0.963828981	1.29419601	1.05681801	0.77661109	1.158777	0.912010789	1.224616051
72	25.25	25.64	44.8500007	23.5400006	20.0599998	sp|Q9DBM2|E	Peroxisomal bifunctional enzyme OS=Mus musculus GN=Ehhadh PE=1 SV=4	MOUSE	18	0.2558586	4.39E-05	1.44544005	0.177010894	0.369828194	0.29107171	1.33E-05	1.44544005	0.161435902	0.420726597	0.69183099	0.00708992	1.14815402	0.597035289	0.794328213
73	24.88	25.18	57.6600015	51.9500017	49.849999	sp|P16858|G3	Glyceraldehyde-3-phosphate dehydrogenase OS=Mus musculus GN=Gapdh PE=1 SV=2	MOUSE	26	0.54450268	0.00093074	1.20226395	0.424619585	0.654636085	0.57543987	0.00234951	1.21338904	0.469894111	0.698232412	0.69823241	0.01106083	1.16949904	0.591561615	0.816582382
74	24.82	24.84	56.3499987	46.5200007	43.4100002	sp|P09411|PG	Phosphoglycerate kinase 1 OS=Mus musculus GN=Pgk1 PE=1 SV=4	MOUSE	19	0.60813499	0.04072703	1.247383	0.469894111	0.758577585	0.7177943	0.08461178	1.21338904	0.55975759	0.870963573	0.79432821	0.1973629	1.18032098	0.654636085	0.937561989
75	24.67	26.92	58.4999979	29.2499989	22.9599997	sp|P14733|LM	Lamin-B1 OS=Mus musculus GN=Lmnb1 PE=1 SV=3	MOUSE	17	1.25892496	0.5347479	1.29419601	0.972747207	1.659587026	1.18032098	0.49443051	1.30617094	0.90364939	1.599557996	1.09647799	0.53566313	1.29419601	0.847227395	1.406048059
76	24.61	24.68	75.9199977	61.8700028	59.5300019	sp|P17751|TP	Triosephosphate isomerase OS=Mus musculus GN=Tpi1 PE=1 SV=4	MOUSE	23	0.8472274	0.3118684	1.13762701	0.717794299	0.963828981	0.87096357	0.3866227	1.11686301	0.765596628	0.972747207	0.50582469	0.02294076	1.35518897	0.334194988	0.685488224

77	24.35	24.35	47.7800012	34.2999995	30.6400001	sp|P47738|AL	Aldehyde dehydrogenase, mitochondrial OS=Mus musculus GN=Aldh2 PE=1 SV=1	MOUSE	19	0.46989411	0.1377805	1.30617094	0.340408206	0.613762021	0.4570882	0.09229548	1.39315701	0.299226493	0.636795521	0.46989411	0.06874593	1.36772895	0.340408206	0.642687678
78	24.22	24.22	71.7000008	39.6200001	39.6200001	sp|O35143|AT	ATPase inhibitor, mitochondrial OS=Mus musculus GN=Atpif1 PE=2 SV=1	MOUSE	16	1.21338904	0.77300638	1.30617094	0.928966403	1.599557996	1	0.77280521	1.29419601	0.772680581	1.30617094	0.59156162	0.20296539	1.33045399	0.420726597	0.787045777
79	24.12	24.3	80.1800013	72.6700008	60.3600025	sp|Q99LC5|E	Electron transfer flavoprotein subunit alpha, mitochondrial OS=Mus musculus GN=Etfa PE=1 SV=2	MOUSE	35	0.93756199	0.61198658	1.19124198	0.794328213	1.116863012	0.98174793	0.85503972	1.18032098	0.831763685	1.158776999	0.34355801	0.03238093	1.41905701	0.26546061	0.487528503
80	23.74	23.74	58.4299982	35.3300005	30.9500009	sp|Q9Z2I8|SU	Succinyl-CoA ligase [GDP-forming] subunit beta, mitochondrial OS=Mus musculus GN=Suclg2 PE=2 SV=3	MOUSE	15	0.79432821	0.27524829	1.21338904	0.648634374	0.963828981	0.79432821	0.07721831	1.23594701	0.636795521	0.981747925	0.7177943	0.07584876	1.23594701	0.570164323	0.88715601
81	23.57	29.6	47.27	33.9599997	24.4000003	sp|P26040|EZ	Ezrin OS=Mus musculus GN=Ezr PE=1 SV=3	MOUSE	25	1.09647799	0.29164791	1.247383	0.879022479	1.419057012	1.47231197	0.1701974	1.29419601	1.137627006	2.089296103	2.20800495	0.00297662	1.36772895	1.614359021	3.46736908
82	23.54	23.63	66.5000022	53.399998	43.689999	sp|P16460|AS	Argininosuccinate synthase OS=Mus musculus GN=Ass1 PE=1 SV=1	MOUSE	21	0.34994519	0.00031281	1.31825697	0.251188606	0.461317599	0.28575909	0.00111202	1.58489299	0.190546095	0.452897608	0.4017908	0.00471177	1.34276497	0.288403213	0.539510608
83	23.47	30.14	43.9500004	13.4000003	9.20000002	sp|Q6URW6|I	Myosin-14 OS=Mus musculus GN=Myh14 PE=1 SV=1	MOUSE	19	0.96382898	0.83471811	1.21338904	0.794328213	1.16949904	1	0.86982042	1.25892496	0.794328213	1.258924961	0.9289664	0.39354259	1.29419601	0.717794299	1.202263951
84	23.4	23.4	53.2999992	44.6700007	40.6100005	sp|Q62433|NI	Protein NDRG1 OS=Mus musculus GN=Ndrg1 PE=1 SV=1	MOUSE	23	0.92044962	0.44472119	1.16949904	0.779830098	1.076465011	0.96382898	0.29096299	1.22461605	0.787045777	1.180320978	0.36643761	0.04295561	1.40604806	0.251188606	0.515228629
85	23.19	23.31	48.33	33.4899992	31.5800011	sp|P07759|SI	Serine protease inhibitor A3K OS=Mus musculus GN=Serpina3k PE=1 SV=2	MOUSE	23	1.16949904	0.56037319	1.21338904	0.963828981	1.445440054	1.14815402	0.44217211	1.22461605	0.937561989	1.419057012	2.67916799	0.00149378	1.29419601	2.070141077	3.944572926
86	22.7	22.7	76.5600026	59.3800008	54.6899974	sp|P19536|C(	Cytochrome c oxidase subunit 5B, mitochondrial OS=Mus musculus GN=Cox5b PE=1 SV=1	MOUSE	21	1.33045399	0.86213172	1.22461605	1.08642602	1.629295945	1.41905701	0.8584736	1.23594701	1.14815402	1.753880978	0.63095743	0.49818781	1.28233099	0.510505021	0.809095919
87	22.67	26.67	52.4500012	29.3900013	27.3499996	sp|P11679|K2	Keratin, type II cytoskeletal 8 OS=Mus musculus GN=Krt8 PE=1 SV=4	MOUSE	19	1.10662401	0.33453989	1.247383	0.88715601	1.472311974	1.52756596	0.09703626	1.29419601	1.180320978	2.992264986	5.05824709	3.20E-05	1.55596602	3.250873089	11.48153973
88	22.46	22.46	53.6599994	43.0299997	37.1199995	sp|P05784|K1	Keratin, type I cytoskeletal 18 OS=Mus musculus GN=Krt18 PE=1 SV=5	MOUSE	13	1.19124198	0.55935538	1.29419601	0.920449615	1.570363045	1.49968505	0.1670913	1.30617094	1.14815402	1.958845019	2.3550489	0.03258526	1.34276497	1.753880978	3.133285999
89	22.36	23.03	37.6399994	18.0199996	15.5200005	sp|Q9JKR6|H	Hypoxia up-regulated protein 1 OS=Mus musculus GN=Hyou1 PE=1 SV=1	MOUSE	15	1.04712904	0.27492821	1.31825697	0.794328213	1.380383968	1.33045399	0.29829121	1.34276497	0.990831971	1.803017974	1.10662401	0.48898339	1.34276497	0.824138105	1.485936046
90	22.19	22.33	64.1499996	41.4700002	31.5299988	sp|P61979|HI	Heterogeneous nuclear ribonucleoprotein K OS=Mus musculus GN=Hnrnpk PE=1 SV=1	MOUSE	20	1.23594701	0.35750151	1.23594701	1	1.51356101	1.62929595	0.08023528	1.28233099	1.270573974	2.147830009	1.28233099	0.2450459	1.247383	1.028015971	1.659587026
91	22.08	22.15	76.5500009	62.8300011	56.190002	sp|P50518|VA	V-type proton ATPase subunit E 1 OS=Mus musculus GN=Atp6v1e1 PE=1 SV=2	MOUSE	19	0.86297852	0.1420943	1.20226395	0.717794299	1.037528038	1.04712904	0.9861728	1.22461605	0.855066717	1.28233099	0.85506672	0.2095124	1.23594701	0.698232412	1.056818008
92	21.65	21.65	44.0699995	40.9200013	29.3000013	sp|P22599|A1	Alpha-1-antitrypsin 1-2 OS=Mus musculus GN=Serpina1b PE=1 SV=2	MOUSE	15	0.73790431	0.74129242	1.48593605	0.501187205	1.096477985	1.22461605	0.1901796	1.39315701	0.879022479	1.737800956	1.870682	0.01858524	1.47231197	1.270573974	2.805433989
93	21.41	21.59	42.9699987	23.1600002	19.54	sp|P28825|MI	Meprin A subunit alpha OS=Mus musculus GN=Mep1a PE=1 SV=4	MOUSE	22	0.59156162	0.2471495	1.247383	0.465586096	0.73790431	0.3767038	0.04500468	1.35518897	0.260615289	0.510505021	0.2679168	0.00416356	1.599558	0.149968505	0.428548515
94	21.29	21.41	59.2499971	24.3799999	24.3799999	sp|Q8CHT0|A	Delta-1-pyrroline-5-carboxylate dehydrogenase, mitochondrial OS=Mus musculus GN=Aldh4a1 PE=1 SV=3	MOUSE	13	0.4325138	0.00029884	1.29419601	0.237683997	0.55975759	0.4017908	0.00017726	1.33045399	0.242102906	0.534564376	0.52480751	0.00147876	1.21338904	0.346736789	0.636795521
95	21.28	25.13	82.2600007	53.6300004	41.1300004	sp|Q6IRU2|TI	Tropomyosin alpha-4 chain OS=Mus musculus GN=Tpm4 PE=2 SV=3	MOUSE	19	0.71121353	0.1576228	1.22461605	0.57543987	0.870963573	0.65463609	0.08497442	1.22461605	0.539510608	0.801678121	0.90364939	0.75880683	1.21338904	0.744732022	1.096477985
96	20.99	21.89	51.9200027	37.3600006	35.710001	sp|P05064|AL	Fructose-bisphosphate aldolase A OS=Mus musculus GN=Aldoa PE=1 SV=2	MOUSE	14	0.4570882	0.03894703	1.62929595	0.260615289	0.744732022	0.87902248	0.1452518	1.35518897	0.630957425	1.19124198	1.70608199	0.321329	1.43218803	1.19124198	2.398833036
97	20.94	20.94	49.0999997	35.3300005	33.5299999	sp|P14152|MI	Malate dehydrogenase, cytoplasmic OS=Mus musculus GN=Mdh1 PE=1 SV=3	MOUSE	16	0.4655861	0.00155497	1.28233099	0.325087309	0.597035289	0.35645109	0.00150019	1.41905701	0.188799098	0.505824685	0.34040821	0.00139192	1.47231197	0.207014099	0.501187205
98	20.88	23.21	35.2200002	3.30399983	2.34500002	sp|Q9QXS1|F	Plectin OS=Mus musculus GN=Plec PE=1 SV=2	MOUSE	10	1.48593605	0.03492251	1.20226395	1.235947013	2.421029091	1.67494297	0.00268005	1.35518897	1.235947013	2.511885881	3.8370719	1.00E-05	1.45881403	2.630268097	6.854881763
99	20.75	20.75	39.4400001	28.8700014	27.8200001	sp|Q60928|G	Gamma-glutamyltranspeptidase 1 OS=Mus musculus GN=Ggt1 PE=1 SV=1	MOUSE	21	1.01859105	0.94711709	1.158777	0.879022479	1.202263951	0.90364939	0.80525827	1.20226395	0.73790431	1.08642602	0.96382898	0.64365	1.20226395	0.794328213	1.158776999
100	20.5	20.51	64.5699978	44.0899998	42.5199986	sp|O09174|AI	Alpha-methylacyl-CoA racemase OS=Mus musculus GN=Amacr PE=1 SV=4	MOUSE	12	0.61376202	0.03344125	1.39315701	0.432513803	0.855066717	0.73113912	0.2785058	1.27057397	0.564936996	0.928966403	0.41304749	0.03226692	1.40604806	0.293765008	0.580764413
101	20.49	21.7	47.8500009	27.7799994	24.3699998	sp|P26443|DI	Glutamate dehydrogenase 1, mitochondrial OS=Mus musculus GN=Glud1 PE=1 SV=1	MOUSE	13	0.59703529	0.50839531	1.31825697	0.457088202	0.787045777	0.65463609	0.1917218	1.34276497	0.48305881	0.879022479	0.7798301	0.91210371	1.31825697	0.597035289	1.028015971
102	20.23	21.03	37.2900009	10.0199997	8.32000002	sp|Q6AW69|(	Cingulin-like protein 1 OS=Mus musculus GN=Cgnl1 PE=1 SV=2	MOUSE	11	1.58489299	0.0251139	1.30617094	1.213389039	2.089296103	1.61435902	0.00163146	1.30617094	1.235947013	2.108628035	1.06659603	0.7810685	1.30617094	0.816582382	1.380383968
103	20.23	20.23	75.3199995	62.9899979	54.549998	sp|P08228|S(	Superoxide dismutase [Cu-Zn] OS=Mus musculus GN=Sod1 PE=1 SV=2	MOUSE	30	2.16770411	0.07480797	1.247383	1.737800956	2.679167986	1.870682	0.1051048	1.23594701	1.51356101	2.312064886	1.00925303	0.38788679	1.20226395	0.839460015	1.202263951
104	19.98	20.03	39.6400005	36.9800001	31.3600004	sp|Q9QXD6|F	Fructose-1,6-bisphosphatase 1 OS=Mus musculus GN=Fbp1 PE=2 SV=3	MOUSE	18	0.67297667	0.05155736	1.21338904	0.519995987	0.816582382	0.58613819	0.01795752	1.27057397	0.461317599	0.744732022	0.52966338	0.02148346	1.29419601	0.39445731	0.685488224
105	19.95	23.78	32.2899997	19.8300004	15.8399999	sp|P08113|EI	Endoplasmin OS=Mus musculus GN=Hsp90b1 PE=1 SV=2	MOUSE	21	0.54450268	0.1824216	1.40604806	0.310455889	0.765596628	0.73113912	0.95909071	1.33045399	0.549540877	0.972747207	1.16949904	0.32860741	1.27057397	0.920449615	1.499685049
106	19.94	19.94	60.3500009	31.2299997	31.2299997	sp|Q99020|R(	Heterogeneous nuclear ribonucleoprotein A/B OS=Mus musculus GN=Hnrnpab PE=1 SV=1	MOUSE	16	1.67494297	0.01660488	1.247383	1.342764974	2.443430901	1.69044101	0.02490906	1.33045399	1.270573974	2.290868044	1.247383	0.09616962	1.29419601	0.963828981	1.644371986
107	19.84	20.75	98.6299992	91.1000013	88.3599997	sp|P12787|C(	Cytochrome c oxidase subunit 5A, mitochondrial OS=Mus musculus GN=Cox5a PE=1 SV=2	MOUSE	27	1.21338904	0.37288779	1.158777	1.047129035	1.393157005	1.06659603	0.3383016	1.158777	0.920449615	1.224616051	0.64268768	0.2667059	1.19124198	0.515228629	0.765596628
108	19.82	19.82	28.8800001	19.5600003	18.4599996	sp|P32020|NI	Non-specific lipid-transfer protein OS=Mus musculus GN=Scp2 PE=1 SV=3	MOUSE	23	1.04712904	0.52318883	1.16949904	0.895364821	1.213389039	1.02801597	0.5857181	1.13762701	0.90364939	1.16949904	0.4875285	0.02401435	1.33045399	0.340408206	0.648634374
109	19.68	19.73	60.5300009	21.2300003	17.1399996	sp|P29341|PA	Polyadenylate-binding protein 1 OS=Mus musculus GN=Pabpc1 PE=1 SV=2	MOUSE	9	1.08642602	0.25463799	1.23594701	0.879022479	1.342764974	0.80167812	0.24800859	1.27057397	0.613762021	1.018591046	0.4325138	0.117315	1.49968505	0.288403213	0.648634374
110	19.59	19.59	72.3800004	55.2399993	53.3299983	sp|P99029|PI	Peroxiredoxin-5, mitochondrial OS=Mus musculus GN=Prdx5 PE=1 SV=2	MOUSE	29	0.57543987	0.0022641	1.21338904	0.469894111	0.698232412	0.51999599	0.00997118	1.20226395	0.432513803	0.625172675	0.34040821	0.0019816	1.23594701	0.26546061	0.420726597
111	19.49	19.49	85.710001	63.4899974	46.8300015	sp|Q8CGP1|I	Histone H2B type 1-K OS=Mus musculus GN=Hist1h2bk PE=1 SV=3	MOUSE	25	0.97274721	0.6316852	1.247383	0.779830098	1.213389039	0.67920363	0.57329178	1.29419601	0.465586096	0.879022479	0.97274721	0.43811771	1.36772895	0.711213529	1.330453992
111	0.02	18.63	85.710001	63.4899974	52.3800015	sp|Q6ZWY9|I	Histone H2B type 1-C/E/G OS=Mus musculus GN=Hist1h2bc PE=1 SV=3	MOUSE	26															
111	0.02	18.63	81.7499995	60.3200018	52.3800015	sp|Q64478|H	Histone H2B type 1-H OS=Mus musculus GN=Hist1h2bh PE=1 SV=3	MOUSE	26															
111	0.02	18.63	81.7499995	63.4899974	52.3800015	sp|Q64525|H	Histone H2B type 2-B OS=Mus musculus GN=Hist2h2bb PE=1 SV=3	MOUSE	26															
111	0.02	18.63	81.7499995	63.4899974	52.3800015	sp|P10854|H2	Histone H2B type 1-M OS=Mus musculus GN=Hist1h2bm PE=1 SV=2	MOUSE	26															
111	0	19.38	85.710001	63.4899974	46.8300015	sp|P10853|H2	Histone H2B type 1-F/J/L OS=Mus musculus GN=Hist1h2bf PE=1 SV=2	MOUSE	25															
111	0	19.38	77.7800024	60.3200018	46.8300015	sp|Q64475|H	Histone H2B type 1-B OS=Mus musculus GN=Hist1h2bb PE=1 SV=3	MOUSE	25															
111	0	18.69	76.9800007	58.7300003	46.8300015	sp|Q8CGP2|I	Histone H2B type 1-P OS=Mus musculus GN=Hist1h2bp PE=1 SV=3	MOUSE	25															
112	19.48	19.6	26.730001	13.4599999	13.4599999	sp|Q04857|C(	Collagen alpha-1(VI) chain OS=Mus musculus GN=Col6a1 PE=2 SV=1	MOUSE	14	1.57036305	0.1070523	1.47231197	1.066596031	2.85758996	1.80301797	0.08686897	1.52756596	1.180320978	3.28095293	2.07014108	0.05365455	1.52756596	1.355188966	3.630779982
113	19.48	19.48	75.2399981	60.4799986	60.4799986	sp|Q8VCR7|A	Abhydrolase domain-containing protein 14B OS=Mus musculus GN=Abhd14b PE=2 SV=1	MOUSE	16	0.50582469	0.1140606	1.34276497	0.337287307	0.679203629	0.43651581	0.1010031	1.47231197	0.258226007	0.642687678	0.1367729	0.05127378	2.46603894	0.061944108	0.337287307
114	19.34	19.42	54.1199982	42.750001	40.0000006	sp|Q9DCW4|I	Electron transfer flavoprotein subunit beta OS=Mus musculus GN=Etfb PE=1 SV=3	MOUSE	22	1.04712904	0.53599137	1.18032098	0.88715601	1.235947013	1.02801597	0.84841591	1.16949904	0.879022479	1.213389039	0.60255963	0.03851702	1.21338904	0.496592313	0.731139123
115	19.34	19.34	71.359998	68.3399975	64.8199976	sp|Q9WVA4|T	Transgelin-2 OS=Mus musculus GN=Tagln2 PE=1 SV=4	MOUSE	27	1.870682	0.06946436	1.27057397	1.472311974	2.376840115	1.97696996	0.02978178	1.30617094	1.51356101	2.511885881	1.80301797	0.2234166	1.27057397	1.419057012	2.22843504
116	19.05	19.05	48.4600008	37.9999995	33.4899992	sp|P45952|A(	Medium-chain specific acyl-CoA dehydrogenase, mitochondrial OS=Mus musculus GN=Acadm PE=1 SV=1	MOUSE	21	0.45289761	0.00070369	1.23594701	0.359749287	0.55975759	0.51050502	0.00100866	1.25892496	0.398107201	0.642687678	0.77268058	0.1362951	1.14815402	0.660693526	0.88715601
117	18.89	18.95	90.0699973	82.1200013	64.2400026	sp|Q60605|M	Myosin light polypeptide 6 OS=Mus musculus GN=Myl6 PE=1 SV=3	MOUSE	17	1.25892496	0.79324877	1.22461605	1.028015971	1.541700006	1.47231197	0.96829188	1.23594701	1.19124198	1.803017974	1.62929595	0.83520269	1.23594701	1.318256974	2.013724089
118	18.57	18.63	42.050001	32.6499999	32.6499999	sp|O88338|C	Cadherin-16 OS=Mus musculus GN=Cdh16 PE=2 SV=1	MOUSE	15	0.87902248	0.7363044	1.21338904	0.717794299	1.066596031	0.71121353	0.1819336	1.247383	0.55975759	0.88715601	0.38018939	0.01686807	1.34276497	0.275422901	0.510505021
119	18.37	18.42	52.8999984	35.7499987	30.9199989	sp|O88844|ID	Isocitrate dehydrogenase [NADP] cytoplasmic OS=Mus musculus GN=Idh1 PE=1 SV=2	MOUSE	14	0.40550849	0.0278105	1.36772895	0.283139199	0.55462569	0.2779713	0.1168161	1.38038397	0.203235701	0.383707315	0.69183099	0.46670899	1.30617094	0.524807513	0.90364939
120	18.13	18.17	43.6100006	19.7799996	16.6700006	sp|Q8BMF4|(	Dihydrolipoyllysine-residue acetyltransferase component of pyruvate dehydrogenase complex, mitochondrial O	MOUSE	14	0.82413811	0.53472942	1.25892496	0.654636085	1.037528038	0.73113912	0.71204418	1.27057397	0.580764413	0.928966403	0.52480751	0.71824843	1.31825697	0.401790798	0.691830993
121	17.99	17.99	45.2100009	38.6999995	36.6400003	sp|Q61937|NI	Nucleophosmin OS=Mus musculus GN=Npm1 PE=1 SV=1	MOUSE	15	1.13762701	0.31837079	1.29419601	0.879022479	1.472311974	0.98174793	0.79598051	1.28233099	0.765596628	1.258924961	1.00925303	0.9367404	1.31825697	0.765596628	1.29419601
122	17.89	18.83	59.4299972	51.2499988	44.479999	sp|P57776|EI	Elongation factor 1-delta OS=Mus musculus GN=Eef1d PE=1 SV=3	MOUSE	10	1.36772895	0.16239341	1.28233099	1.066596031	1.853531957	1.35518897	0.2484871	1.38038397	0.981747925	1.923092008	1.19124198	0.421956	1.41905701	0.839460015	1.690441012
123	17.68	17.83	30.4800004	13.8999999	10.4800001	sp|Q922D8|C	C-1-tetrahydrofolate synthase, cytoplasmic OS=Mus musculus GN=Mthfd1 PE=1 SV=4	MOUSE	8	0.80167812	0.1093396	1.29419601	0.613762021	1.037528038	0.90364939	0.41020659	1.28233099	0.698232412	1.158776999	0.60813499	0.1597438	1.44544005	0.424619585	0.879022479
124	17.67	17.82	45.7899988	30.7200015	30.7200015	sp|P62814|VA	V-type proton ATPase subunit B, brain isoform OS=Mus musculus GN=Atp6v1b2 PE=1 SV=1	MOUSE	13	0.57016432	0.03883971	1.25892496	0.424619585	0.717794299	0.53456438	0.01105782	1.28233099	0.380189389	0.685488224	0.83946002	0.64293838	1.27057397	0.660693526	1.066596031
125	17.64	17.64	47.3699987	31.5800011	29.0499985	sp|Q99JY0|E(	Trifunctional enzyme subunit beta, mitochondrial OS=Mus musculus GN=Hadhb PE=1 SV=1	MOUSE	12	0.53456438	0.04868333	1.39315701	0.301995188	0.744732022	0.85506672	0.03178808	1.247383	0.679203629	1.066596031	1.04712904	0.35320181	1.25892496	0.831763685	1.330453992
126	17.52	17.52	36.4100009	16.5199995	15.0700003	sp|P11087|C(	Collagen alpha-1(I) chain OS=Mus musculus GN=Col1a1 PE=1 SV=4	MOUSE	21	1.18032098	0.54791242	1.247383	0.946237087	1.51356101	1.33045399	0.4476611	1.34276497	0.990831971	1.853531957	2.48885703	0.04259274	1.57036305	1.584892988	4.285484791
127	17.47	17.5	48.2800007	43.6800003	41.7600006	sp|P12658|C	Calbindin OS=Mus musculus GN=Calb1 PE=1 SV=2	MOUSE	22	1.58489299	0.0382311	1.21338904	1.30617094	1.976969957	1.31825697	0.156224	1.19124198	1.106624007	1.644371986	0.83946002	0.53226697	1.19124198	0.704693079	1
128	17.44	17.51	48.3099997	39.8900002	32.8700006	sp|P60335|P(	Poly(rC)-binding protein 1 OS=Mus musculus GN=Pcbp1 PE=1 SV=1	MOUSE	10	1.67494297	0.29905281	1.43218803	1.16949904	2.728977919	1.22461605	0.49495411	1.52756596	0.801678121	1.976969957	0.85506672	0.5114519	1.51356101	0.570164323	1.29419601
129	17.41	17.48	58.7199986	46.7900008	40.3699994	sp|P10649|G	Glutathione S-transferase Mu 1 OS=Mus musculus GN=Gstm1 PE=1 SV=2	MOUSE	16	0.61376202	0.01797259	1.23594701	0.469894111	0.758577585	0.474242	0.00068938	1.22461605	0.349945188	0.580764413	0.2290868	0.00380107	1.48593605	0.135518894	0.340408206
130	17.11	17.18	69.9500024	58.2199991	48.3599991	sp|Q9DB20|A	ATP synthase subunit O, mitochondrial OS=Mus musculus GN=Atp5o PE=1 SV=1	MOUSE	16	0.60255963	0.06399167	1.33045399	0.452897608	0.801678121	0.76559663	0.220512	1.31825697	0.570164323	1.009253025	0.82413811	0.3180413	1.31825697	0.619441092	1.08642602
131	17.06	17.27	52.5900006	35.8500004	31.3699991	sp|Q9JHI5|IV	Isovaleryl-CoA dehydrogenase, mitochondrial OS=Mus musculus GN=Ivd PE=1 SV=1	MOUSE	10	0.69183099	0.01004206	1.27057397	0.534564376	0.879022479	0.63679552	0.00444137	1.247383	0.496592313	0.794328213	0.67920363	0.0148346	1.19124198	0.55975759	0.809095919
132	17.03	17.03	38.1000012	24.8500004	22.8900001	sp|Q8K2B3|D	Succinate dehydrogenase [ubiquinone] flavoprotein subunit, mitochondrial OS=Mus musculus GN=Sdha PE=1	MOUSE	21	0.39445731	0.1863962	1.61435902	0.244343102	0.636795521	0.28054339	0.1065084	1.70608199	0.194088593	0.478630096	0.53456438	0.00475771	1.31825697	0.405508488	0.704693079
133	17.01	17.01	77.7800024	70.8999991	51.849997	sp|Q99LX0|P	Protein DJ-1 OS=Mus musculus GN=Park7 PE=1 SV=1	MOUSE	15	1.11686301	0.79686552	1.20226395	0.928966403	1.342764974	1.27057397	0.24993371	1.20226395	1.056818008	1.55596602	0.72443599	0.358455	1.20226395	0.602559626	0.870963573
134	16.97	16.97	59.6800029	50.4000008	43.1499988	sp|Q9D6J6|N	NADH dehydrogenase [ubiquinone] flavoprotein 2, mitochondrial OS=Mus musculus GN=Ndufv2 PE=1 SV=2	MOUSE	16	0.74473202	0.197704	1.28233099	0.57543987	0.954992592	0.81658238	0.27229789	1.28233099	0.630957425	1.047129035	0.35974929	0.0454452	1.39315701	0.263026804	0.501187205
135	16.91	16.91	76.5200019	76.5200019	76.5200019	sp|P99027|RI	60S acidic ribosomal protein P2 OS=Mus musculus GN=Rplp2 PE=1 SV=3	MOUSE	25	2.18776202	0.0647651	1.29419601	1.690441012	2.884032011	2.3550489	0.03236039	1.29419601	1.819700956	3.104559898	2.051162	0.1203146	1.28233099	1.599557996	2.754229069
136	16.88	16.94	30.5299997	26.0500014	24.3699998	sp|Q64442|DI	Sorbitol dehydrogenase OS=Mus musculus GN=Sord PE=1 SV=3	MOUSE	9	1.09647799	0.198819	1.29419601	0.847227395	1.419057012	0.89536482	0.15549479	1.30617094	0.648634374	1.16949904	0.70469308	0.06569607	1.31825697	0.496592313	0.928966403
137	16.72	16.72	65.4100001	33.7799996	33.7799996	sp|Q9CZ44|N	NSFL1 cofactor p47 OS=Mus musculus GN=Nsfl1c PE=1 SV=1	MOUSE	15	1.69044101	0.1300191	1.36772895	1.235947013	2.333457947	1.47231197	0.36210519	1.35518897	1.08642602	2.013724089	0.9289664	0.8884331	1.34276497	0.691830993	1.247382998
138	16.59	16.59	41.9200003	39.3000007	39.3000007	sp|Q6IRU5|C	Clathrin light chain B OS=Mus musculus GN=Cltb PE=2 SV=1	MOUSE	13	2.39883304	0.01555602	1.65958703	1.445440054	4.405549049	2.22843504	0.0147126	1.61435902	1.380383968	4.092607021	1.80301797	0.1021293	1.57036305	1.14815402	2.992264986
139	16.54	16.6	74.5100021	69.6099997	64.7099972	sp|P09813|AI	Apolipoprotein A-II OS=Mus musculus GN=Apoa2 PE=1 SV=1	MOUSE	26	6.02559614	0.00067656	1.25892496	4.786301136	7.79830122	5.49540901	0.00369779	1.28233099	4.285484791	7.112134933	5.49540901	0.0038746	1.25892496	4.365159035	7.04693079
140	16.53	16.59	55.0899983	47.6000011	32.04	sp|P16125|LD	L-lactate dehydrogenase B chain OS=Mus musculus GN=Ldhb PE=1 SV=2	MOUSE	14	0.91201079	0.30591551	1.38038397	0.666806817	1.258924961	0.87902248	0.2953158	1.39315701	0.636795521	1.224616051	0.70469308	0.06716425	1.38038397	0.515228629	0.972747207
141	16.5	16.5	40.93	26.3599992	23.1800005	sp|P80314|T(	T-complex protein 1 subunit beta OS=Mus musculus GN=Cct2 PE=1 SV=4	MOUSE	12	1.08642602	0.75110322	1.31825697	0.824138105	1.432188034	1.31825697	0.58554	1.33045399	0.990831971	1.737800956	1.58489299	0.3321251	1.33045399	1.19124198	2.089296103
142	16.46	16.59	38.8700008	24.8500004	21.4900002	sp|P50544|A(	Very long-chain specific acyl-CoA dehydrogenase, mitochondrial OS=Mus musculus GN=Acadvl PE=1 SV=3	MOUSE	12	0.2290868	0.27804911	1.49968505	0.149968505	0.343558013	0.73113912	0.1063578	1.35518897	0.510505021	0.990831971	0.564937	0.01048036	1.41905701	0.319153786	0.801678121
143	16.39	16.65	35.4000002	11.8000001	11.8000001	sp|Q99K41|E	EMILIN-1 OS=Mus musculus GN=Emilin1 PE=1 SV=1	MOUSE	12	2.60615301	0.09482122	1.44544005	1.803017974	3.872575998	3.2210691	0.05272887	1.45881403	2.208004951	4.698941231	5.19996023	0.00573591	1.54170001	3.372873068	8.241380692
144	16.33	16.44	41.9099987	32.8200012	30.1600009	sp|P05213|TE	Tubulin alpha-1B chain OS=Mus musculus GN=Tuba1b PE=1 SV=2	MOUSE	18	1.14815402	0.369432	1.34276497	0.855066717	1.629295945	1.08642602	0.84834403	1.39315701	0.779830098	1.51356101	1.48593605	0.14879289	1.40604806	1.056818008	2.089296103
144	0.16	15.81	41.6500002	32.5199992	25.6099999	sp|P68373|TE	Tubulin alpha-1C chain OS=Mus musculus GN=Tuba1c PE=1 SV=1	MOUSE	17															
144	0	14.3	41.4600015	29.7100008	27.0500004	sp|P68369|TE	Tubulin alpha-1A chain OS=Mus musculus GN=Tuba1a PE=1 SV=1	MOUSE	17															
145	16.26	16.54	33.8499993	23.8900006	23.8900006	sp|Q8BFR5|E	Elongation factor Tu, mitochondrial OS=Mus musculus GN=Tufm PE=1 SV=1	MOUSE	12	0.80167812	0.73531288	1.38038397	0.57543987	1.106624007	0.4655861	0.07899162	1.54170001	0.218776196	0.717794299	0.58613819	0.3151753	1.45881403	0.366437614	0.855066717
146	16.2	37.98	99.3200004	94.5599973	90.4799998	sp|P02089|HI	Hemoglobin subunit beta-2 OS=Mus musculus GN=Hbb-b2 PE=1 SV=2	MOUSE	69	1.77010906	0.21485309	1.36772895	1.29419601	3.191538095	1.80301797	0.1719953	1.34276497	1.342764974	2.937649965	4.09260702	0.00346618	1.48593605	2.754229069	8.241380692
147	16.17	16.2	40.200001	30.1499993	30.1499993	sp|Q99JW2|A	Aminoacylase-1 OS=Mus musculus GN=Acy1 PE=1 SV=1	MOUSE	16	0.38370732	0.00946856	1.36772895	0.258226007	0.524807513	0.50582469	0.01336608	1.28233099	0.366437614	0.648634374	0.2032357	0.00201169	1.55596602	0.081658237	0.316227794

148	16.15	16.22	86.1199975	59.1799974	50.6099999	sp|P63101|14	14-3-3 protein zeta/delta OS=Mus musculus GN=Ywhaz PE=1 SV=1	MOUSE	16	0.65463609	0.76011813	1.36772895	0.478630096	0.895364821	0.74473202	0.53606153	1.33045399	0.55462569	0.990831971	0.76559663	0.85627848	1.36772895	0.55975759	1.047129035
149	16.13	16.15	22.7300003	11.0699996	11.0699996	sp|P58252|EF	Elongation factor 2 OS=Mus musculus GN=Eef2 PE=1 SV=2	MOUSE	10	0.72443599	0.1258854	1.22461605	0.586138189	0.88715601	0.69823241	0.109165	1.33045399	0.510505021	0.928966403	0.87096357	0.49719361	1.27057397	0.685488224	1.106624007
150	16.03	16.04	34.8100007	14.4999996	12.4300003	sp|Q91ZA3|P	Propionyl-CoA carboxylase alpha chain, mitochondrial OS=Mus musculus GN=Pcca PE=2 SV=2	MOUSE	9	0.62517268	0.05220307	1.30617094	0.474242002	0.816582382	0.43651581	0.02618948	1.43218803	0.296483099	0.625172675	0.41304749	0.07504622	1.44544005	0.304789513	0.597035289
151	15.67	15.76	26.2600005	9.46400017	8.67500007	sp|Q8VDJ3|V	Vigilin OS=Mus musculus GN=Hdlbp PE=1 SV=1	MOUSE	10	1.22461605	0.18962351	1.33045399	0.920449615	1.67494297	0.73113912	0.74291712	1.44544005	0.505824685	1.056818008	0.54954088	0.38051459	1.45881403	0.390840888	0.801678121
152	15.54	15.95	50	34.1399998	32.4099988	sp|Q8BH95|E	Enoyl-CoA hydratase, mitochondrial OS=Mus musculus GN=Echs1 PE=1 SV=1	MOUSE	11	0.71121353	0.1346112	1.31825697	0.524807513	0.937561989	0.66069353	0.1404444	1.31825697	0.48305881	0.870963573	0.64268768	0.106671	1.23594701	0.492039502	0.794328213
153	15.53	15.93	29.9600005	20.8499998	19.8400006	sp|Q9JLJ2|AL	4-trimethylaminobutyraldehyde dehydrogenase OS=Mus musculus GN=Aldh9a1 PE=1 SV=1	MOUSE	9	0.7798301	0.09712909	1.23594701	0.613762021	0.963828981	0.78704578	0.1466919	1.31825697	0.580764413	1.037528038	0.75162292	0.100925	1.31825697	0.549540877	0.990831971
154	15.46	15.51	39.9599999	26.699999	21.3599995	sp|Q60864|ST	Stress-induced-phosphoprotein 1 OS=Mus musculus GN=Stip1 PE=1 SV=1	MOUSE	13	1.47231197	0.03662163	1.27057397	1.158776999	1.976969957	1.30617094	0.41761571	1.27057397	1.028015971	1.721868992	0.91201079	0.3417587	1.247383	0.711213529	1.137627006
155	15.44	15.51	26.1299998	9.56100002	9.56100002	sp|Q9WTQ5|A	A-kinase anchor protein 12 OS=Mus musculus GN=Akap12 PE=1 SV=1	MOUSE	12	3.07609701	0.02266569	1.77010906	1.737800956	6.025596142	3.37287307	0.00673432	1.73780096	1.940886021	5.754398823	7.37904215	0.00064904	1.80301797	4.092607021	13.06171036
156	15.44	15.5	54.8200011	51.8100023	51.8100023	sp|P18760|C	Cofilin-1 OS=Mus musculus GN=Cfl1 PE=1 SV=3	MOUSE	12	0.83176369	0.77159518	1.33045399	0.597035289	1.106624007	0.97274721	0.8964057	1.29419601	0.751622915	1.258924961	1.20226395	0.44530249	1.27057397	0.946237087	1.541700006
157	15.42	15.47	30.2399993	17.2199994	14.0200004	sp|P26231|CT	Catenin alpha-1 OS=Mus musculus GN=Ctnna1 PE=1 SV=1	MOUSE	11	1.67494297	0.04986423	1.44544005	1.158776999	2.466038942	1.43218803	0.5555436	1.43218803	1	2.089296103	1.16949904	0.91481429	1.40604806	0.831763685	1.644371986
158	15.38	15.55	49.2399991	28.5400003	18.9500004	sp|Q02819|N	Nucleobindin-1 OS=Mus musculus GN=Nucb1 PE=1 SV=2	MOUSE	12	1.30617094	0.43232909	1.31825697	0.990831971	1.721868992	0.98174793	0.97197712	1.30617094	0.751622915	1.28233099	0.90364939	0.76902932	1.27057397	0.711213529	1.14815402
159	15.24	15.24	96.5200007	89.5699978	66.960001	sp|P34884|M	Macrophage migration inhibitory factor OS=Mus musculus GN=Mif PE=1 SV=2	MOUSE	20	1.36772895	0.34444681	1.30617094	1.047129035	1.870682001	1.48593605	0.1317482	1.35518897	1.096477985	2.032356977	0.51522863	0.05738208	1.35518897	0.380189389	0.698232412
160	15.21	15.21	86.6699994	66.6700006	56.190002	sp|P62897|CY	Cytochrome c, somatic OS=Mus musculus GN=Cycs PE=1 SV=2	MOUSE	27	1.55596602	0.1112127	1.25892496	1.235947013	1.958845019	0.82413811	0.1509216	1.247383	0.660693526	1.028015971	0.69183099	0.09656677	1.22461605	0.55975759	0.847227395
161	15.12	15.56	27.9599994	12.2199997	8.99299979	sp|Q60597|O	2-oxoglutarate dehydrogenase, mitochondrial OS=Mus musculus GN=Ogdh PE=1 SV=3	MOUSE	10	0.61376202	0.1196045	1.30617094	0.452897608	0.801678121	0.80909592	0.3896271	1.247383	0.648634374	1.009253025	0.65463609	0.2444744	1.28233099	0.510505021	0.839460015
162	15.01	15.36	42.8000003	14.1299993	13.3100003	sp|Q99K30|E	Epidermal growth factor receptor kinase substrate 8-like protein 2 OS=Mus musculus GN=Eps8l2 PE=1 SV=1	MOUSE	7	1.90546095	0.1779612	1.52756596	1.247382998	2.992264986	1.36772895	0.1417881	1.51356101	0.90364939	2.070141077	1.25892496	0.68196058	1.52756596	0.824138105	1.923092008
163	14.92	14.92	49.7500002	39.199999	37.1899992	sp|P35700|PF	Peroxiredoxin-1 OS=Mus musculus GN=Prdx1 PE=1 SV=1	MOUSE	14	0.59703529	0.16767441	1.27057397	0.452897608	0.758577585	0.57543987	0.19045331	1.31825697	0.444631308	0.758577585	0.61944109	0.39143649	1.29419601	0.465586096	0.801678121
164	14.85	14.85	54.5000017	30.0999999	23.5300005	sp|Q8VC30|D	Bifunctional ATP-dependent dihydroxyacetone kinase/FAD-AMP lyase (cyclizing) OS=Mus musculus GN=Dak	MOUSE	9	1.01859105	0.08852519	1.40604806	0.724435985	1.419057012	1.45881403	0.37900671	1.39315701	1.047129035	2.032356977	1.05681801	0.48067612	1.54170001	0.685488224	1.499685049
165	14.79	15.04	47.7299988	42.3000008	28.0999988	sp|P47199|Q	Quinone oxidoreductase OS=Mus musculus GN=Cryz PE=2 SV=1	MOUSE	17	0.65463609	0.05193326	1.27057397	0.478630096	0.831763685	0.9289664	0.73348951	1.23594701	0.751622915	1.14815402	0.58613819	0.06762147	1.27057397	0.4487454	0.744732022
166	14.79	14.98	33.5900009	12.9700005	10.9800004	sp|Q8R0Y6|A	Cytosolic 10-formyltetrahydrofolate dehydrogenase OS=Mus musculus GN=Aldh1l1 PE=2 SV=1	MOUSE	8	0.29376501	0.00303238	1.85353196	0.125892505	0.544502676	0.44463131	0.00731494	1.47231197	0.258226007	0.654636085	0.30760971	0.02601963	1.94088602	0.169044107	0.597035289
167	14.78	21.76	54.1599989	37.529999	26.0100007	sp|P31001|DE	Desmin OS=Mus musculus GN=Des PE=1 SV=3	MOUSE	13	1.21338904	0.4489302	1.28233099	0.946237087	1.737800956	1.25892496	0.38424391	1.30617094	0.963828981	1.803017974	1.64437199	0.07907374	1.38038397	1.19124198	2.511885881
168	14.58	14.58	81.0800016	46.8499988	46.8499988	sp|P26350|PT	Prothymosin alpha OS=Mus musculus GN=Ptma PE=1 SV=2	MOUSE	25	2.33345795	0.18367641	1.45881403	1.599557996	3.732501984	2.6546061	0.1577408	1.48593605	1.786488056	4.130475044	2.12813902	0.2137128	1.43218803	1.485936046	3.435580015
169	14.57	14.57	52.5900006	28.2999992	25	sp|Q921H8|T	3-ketoacyl-CoA thiolase A, peroxisomal OS=Mus musculus GN=Acaa1a PE=2 SV=1	MOUSE	14	0.88715601	0.5477339	1.30617094	0.672976673	1.158776999	1	0.59836739	1.27057397	0.787045777	1.29419601	1.12719798	0.34929371	1.34276497	0.839460015	1.51356101
170	14.56	14.9	93.1400001	85.2900028	74.5100021	sp|Q64433|C	10 kDa heat shock protein, mitochondrial OS=Mus musculus GN=Hspe1 PE=1 SV=2	MOUSE	23	2.46603894	0.00429957	1.25892496	1.958845019	3.66437602	2.07014108	0.03017869	1.23594701	1.67494297	3.162277937	1.14815402	0.4189117	1.18032098	0.972747207	1.499685049
171	14.55	14.56	87.5800014	60.8699977	57.1399987	sp|Q9DCX2|A	ATP synthase subunit d, mitochondrial OS=Mus musculus GN=Atp5h PE=1 SV=3	MOUSE	13	0.87902248	0.09057529	1.20226395	0.724435985	1.056818008	0.69823241	0.01753686	1.22461605	0.570164323	0.855066717	0.75857759	0.0093587	1.20226395	0.613762021	0.912010789
172	14.45	14.59	43.2200015	25.4599989	15.0199994	sp|Q60598|S	Src substrate cortactin OS=Mus musculus GN=Cttn PE=1 SV=2	MOUSE	6	1.20226395	0.26152059	1.247383	0.963828981	1.541700006	1.20226395	0.1289866	1.22461605	0.981747925	1.499685049	0.7798301	0.42368591	1.35518897	0.57543987	1.056818008
173	14.43	14.43	33.039999	23.0399996	19.3499997	sp|Q91X72|H	Hemopexin OS=Mus musculus GN=Hpx PE=1 SV=2	MOUSE	9	2.37684012	0.03735408	1.85353196	1.28233099	3.732501984	3.872576	0.01382374	2.24905491	1.721868992	8.953647614	11.9124203	0.00022641	2.46603894	4.830587864	23.33457947
174	14.33	14.46	35.0499988	19.6099997	15.9199998	sp|Q9Z2V4|P	Phosphoenolpyruvate carboxykinase, cytosolic [GTP] OS=Mus musculus GN=Pck1 PE=2 SV=1	MOUSE	8	0.44055489	0.00570521	1.36772895	0.304789513	0.602559626	0.46989411	0.00399638	1.38038397	0.310455889	0.648634374	0.66069353	0.20480061	1.34276497	0.487528503	0.88715601
175	14.32	14.39	41.1500007	26.5399993	16.3499996	sp|Q9D0K2|S	Succinyl-CoA:3-ketoacid-coenzyme A transferase 1, mitochondrial OS=Mus musculus GN=Oxct1 PE=1 SV=1	MOUSE	7	0.35974929	0.02039536	1.47231197	0.229086801	0.529663384	0.59703529	0.02793332	1.33045399	0.432513803	0.794328213	0.55975759	0.0325578	1.35518897	0.387257606	0.758577585
176	14.28	14.38	76.6799986	38.9800012	35.1399988	sp|Q9Z204|H	Heterogeneous nuclear ribonucleoproteins C1/C2 OS=Mus musculus GN=Hnrnpc PE=1 SV=1	MOUSE	10	1.61435902	0.08131453	1.58489299	1.018591046	2.754229069	1.61435902	0.1828575	1.64437199	0.981747925	2.728977919	1.38038397	0.2530441	1.54170001	0.895364821	2.269865036
177	14.24	14.3	28.1300008	17.9199994	17.9199994	sp|Q9CPY7|A	Cytosol aminopeptidase OS=Mus musculus GN=Lap3 PE=1 SV=3	MOUSE	9	0.2831392	0.05158858	2.29086804	0.120226398	0.648634374	0.197697	0.04660761	3.1045599	0.1086426	0.613762021	0.25822601	0.06108016	2.58225989	0.165958703	0.666806817
178	14.22	14.45	56.8199992	34.5400006	32.8700006	sp|Q9D051|O	Pyruvate dehydrogenase E1 component subunit beta, mitochondrial OS=Mus musculus GN=Pdhb PE=1 SV=1	MOUSE	23	0.75162292	0.07704937	1.28233099	0.580764413	0.963828981	0.82413811	0.14203019	1.27057397	0.642687678	1.047129035	0.75162292	0.15595549	1.30617094	0.570164323	0.981747925
179	14.21	14.27	33.6899996	19.6799994	18.0600002	sp|Q99L47|F	Hsc70-interacting protein OS=Mus musculus GN=St13 PE=2 SV=1	MOUSE	8	1.34276497	0.66547972	1.45881403	0.920449615	1.958845019	1.54170001	0.40362081	1.49968505	1.028015971	2.312064886	0.63679552	0.4106178	1.51356101	0.390840888	0.963828981
180	14.17	14.17	75	32.6599985	29.4400007	sp|Q6PDM2|S	Serine/arginine-rich splicing factor 1 OS=Mus musculus GN=Srsf1 PE=1 SV=3	MOUSE	8	1.81970096	0.26068279	1.34276497	1.355188966	2.488857031	2.051162	0.1372952	1.35518897	1.51356101	2.83139205	1.23594701	0.97265881	1.30617094	0.946237087	1.614359021
181	14.16	14.21	47.8899986	23.1000006	22.8200004	sp|Q60668|H	Heterogeneous nuclear ribonucleoprotein D0 OS=Mus musculus GN=Hnrnpd PE=1 SV=2	MOUSE	12	1.09647799	0.74076378	1.40604806	0.779830098	1.570363045	1.05681801	0.65914148	1.34276497	0.787045777	1.499685049	0.65463609	0.158199	1.34276497	0.390840888	0.879022479
182	14.11	20.45	64.3299997	34.1399998	30.6300014	sp|Q9DCV7|K	Keratin, type II cytoskeletal 7 OS=Mus musculus GN=Krt7 PE=1 SV=1	MOUSE	13	1.13762701	0.53255439	1.31825697	0.862978518	1.527565956	1.28233099	0.30471131	1.30617094	0.981747925	1.659587026	1.51356101	0.2590718	1.33045399	1.137627006	2.070141077
183	14.07	14.24	42.2899991	24.2300004	21.3699997	sp|Q9D2G2|C	Dihydrolipoyllysine-residue succinyltransferase component of 2-oxoglutarate dehydrogenase complex, mitocho	MOUSE	15	0.54954088	0.2394872	1.28233099	0.424619585	0.704693079	0.53456438	0.04875816	1.30617094	0.40926069	0.698232412	0.4017908	0.01953365	1.39315701	0.283139199	0.55975759
184	14.03	14.03	77.5900006	68.0999994	68.0999994	sp|P52503|N	NADH dehydrogenase [ubiquinone] iron-sulfur protein 6, mitochondrial OS=Mus musculus GN=Ndufs6 PE=1 S	MOUSE	12	1.67494297	0.03630267	1.19124198	1.406048059	2.051162004	1.599558	0.02153641	1.14815402	1.393157005	1.905460954	1.06659603	0.89143229	1.16949904	0.912010789	1.247382998
185	13.88	13.93	37.3100013	24.3799999	20.9000006	sp|Q99KJ8|D	Dynactin subunit 2 OS=Mus musculus GN=Dctn2 PE=1 SV=3	MOUSE	8	1.80301797	0.09180625	1.29419601	1.393157005	2.355048895	1.31825697	0.79113632	1.28233099	1.028015971	1.690441012	1.20226395	0.88653421	1.28233099	0.937561989	1.55596602
186	13.77	13.77	46.509999	40.1199997	40.1199997	sp|Q3THE2|M	Myosin regulatory light chain 12B OS=Mus musculus GN=Myl12b PE=1 SV=2	MOUSE	10	0.35974929	0.60784411	2.58225989	0.135518894	0.928966403	0.48305881	0.4898504	1.65958703	0.185353205	0.801678121	0.88715601	0.80763137	1.51356101	0.529663384	1.342764974
187	13.64	13.64	62.4199987	36.9599998	33.5399985	sp|P62960|YB	Nuclease-sensitive element-binding protein 1 OS=Mus musculus GN=Ybx1 PE=1 SV=3	MOUSE	12	2.01372409	0.05885048	1.54170001	1.30617094	3.28095293	1.55596602	0.45966381	1.67494297	0.928966403	2.630268097	1.47231197	0.31475139	1.61435902	0.912010789	2.421029091
188	13.62	13.92	39.4199997	22.1699998	14.0000001	sp|O54931|A	A-kinase anchor protein 2 OS=Mus musculus GN=Akap2 PE=1 SV=3	MOUSE	9	1.870682	0.02270835	1.31825697	1.419057012	2.511885881	1.33045399	0.86781979	1.31825697	1.009253025	1.770109057	1.88799095	0.04206936	1.31825697	1.432188034	2.53512907
189	13.61	14.71	30.7099998	9.81900021	6.67700023	sp|Q3UMF0|C	Cordon-bleu protein-like 1 OS=Mus musculus GN=Cobll1 PE=1 SV=2	MOUSE	8	1.08642602	0.08785651	1.35518897	0.801678121	1.458814025	0.95499259	0.32635969	1.28233099	0.731139123	1.224616051	0.4017908	0.06186479	1.54170001	0.214782998	0.619441092
190	13.56	13.56	41.4299995	17.8900003	14.6899998	sp|P52480|K	Pyruvate kinase isozymes M1/M2 OS=Mus musculus GN=Pkm2 PE=1 SV=4	MOUSE	8	0.51050502	0.1148827	1.64437199	0.316227794	0.839460015	0.98174793	0.35388851	1.47231197	0.672976673	1.445440054	0.98174793	0.4126184	1.51356101	0.648634374	1.485936046
191	13.49	17.05	45.1200008	23.7499997	23.7499997	sp|Q8BG05|R	Heterogeneous nuclear ribonucleoprotein A3 OS=Mus musculus GN=Hnrnpa3 PE=1 SV=1	MOUSE	12	0.86297852	0.53304452	1.29419601	0.672976673	1.116863012	0.7177943	0.2844137	1.29419601	0.534564376	0.928966403	0.49659231	0.08986329	1.40604806	0.356451094	0.698232412
192	13.47	13.55	46.5499997	31.0299993	31.0299993	sp|Q9CZU6|C	Citrate synthase, mitochondrial OS=Mus musculus GN=Cs PE=1 SV=1	MOUSE	12	0.4875285	0.1565555	1.51356101	0.270395786	0.73790431	0.67297667	0.34290329	1.39315701	0.457088202	0.937561989	0.79432821	0.52126801	1.38038397	0.55462569	1.096477985
193	13.44	13.47	42.9899991	32.2400004	29.550001	sp|Q99L13|3H	3-hydroxyisobutyrate dehydrogenase, mitochondrial OS=Mus musculus GN=Hibadh PE=2 SV=1	MOUSE	12	0.85506672	0.57810849	1.34276497	0.630957425	1.14815402	0.87902248	0.39416221	1.31825697	0.666806817	1.158776999	0.69183099	0.2376748	1.40604806	0.492039502	0.972747207
194	13.42	13.45	38.8900012	27.5000006	23.33	sp|P55302|AM	Alpha-2-macroglobulin receptor-associated protein OS=Mus musculus GN=Lrpap1 PE=1 SV=1	MOUSE	10	0.93756199	0.78306621	1.25892496	0.73790431	1.180320978	1.06659603	0.65016228	1.40604806	0.758577585	1.499685049	0.23120651	0.1158717	1.88799095	0.129419595	0.436515808
195	13.38	13.38	35.3300005	35.3300005	35.3300005	sp|Q9CPU0|L	Lactoylglutathione lyase OS=Mus musculus GN=Glo1 PE=1 SV=3	MOUSE	10	1.41905701	0.60980898	1.27057397	1.116863012	1.786488056	1.51356101	0.93451142	1.27057397	1.19124198	1.905460954	0.60255963	0.00810328	1.30617094	0.387257606	0.787045777
196	13.35	13.35	32.6099992	18.7600002	15.0099993	sp|Q61033|LA	Lamina-associated polypeptide 2, isoforms alpha/zeta OS=Mus musculus GN=Tmpo PE=1 SV=4	MOUSE	12	6.85488176	0.08812351	1.97696996	3.46736908	19.23092079	6.13761997	0.1058415	2.01372409	3.047894955	17.06081963	7.31139088	0.07271058	2.03235698	3.597492933	20.51161957
197	13.33	13.33	52.2400022	52.2400022	52.2400022	sp|P56395|CY	Cytochrome b5 OS=Mus musculus GN=Cyb5a PE=1 SV=2	MOUSE	21	0.80909592	0.32044479	1.16949904	0.691830993	0.946237087	0.50582469	0.090893	1.28233099	0.383707315	0.648634374	0.31332859	0.0318563	1.62929595	0.194088593	0.510505021
198	13.25	13.26	34.8699987	23.33	23.0800003	sp|P35486|O	Pyruvate dehydrogenase E1 component subunit alpha, somatic form, mitochondrial OS=Mus musculus GN=Pd	MOUSE	9	0.89536482	0.3124285	1.27057397	0.698232412	1.137627006	0.88715601	0.2234775	1.28233099	0.672976673	1.137627006	0.95499259	0.88931167	1.36772895	0.698232412	1.30617094
199	13.21	13.22	28.9600015	15.0099993	12.9999995	sp|Q8K310|M	Matrin-3 OS=Mus musculus GN=Matr3 PE=1 SV=1	MOUSE	13	1.08642602	0.38375041	1.23594701	0.879022479	1.342764974	1.11686301	0.248339	1.247383	0.895364821	1.393157005	1.00925303	0.71441251	1.14815402	0.879022479	1.158776999
200	13.19	13.19	82.8800023	69.3700016	53.1499982	sp|Q9D855|Q	Cytochrome b-c1 complex subunit 7 OS=Mus musculus GN=Uqcrb PE=1 SV=3	MOUSE	16	1	0.55221468	1.22461605	0.816582382	1.224616051	1.20226395	0.26479799	1.16949904	1.028015971	1.419057012	0.72443599	0.09097871	1.247383	0.57543987	0.90364939
201	13.17	13.17	55.6100011	39.9100006	30.9399992	sp|Q9WUR9|I	Adenylate kinase isoenzyme 4, mitochondrial OS=Mus musculus GN=Ak4 PE=2 SV=1	MOUSE	6	0.69823241	0.4257341	1.27057397	0.398107201	0.88715601	0.8472274	0.5719946	1.247383	0.654636085	1.056818008	0.4325138	0.1369845	1.49968505	0.2013724	0.648634374
202	13.14	13.17	87.7600014	72.7900028	72.7900028	sp|P07309|TT	Transthyretin OS=Mus musculus GN=Ttr PE=1 SV=1	MOUSE	10	2.77971292	0.0870585	1.55596602	1.786488056	5.199960232	1.33045399	0.2763195	1.38038397	0.963828981	2.22843504	5.01187182	0.01815352	1.599558	3.133285999	8.790225029
203	13.1	13.23	50	26.6000003	24.1099998	sp|Q9CQA3|D	Succinate dehydrogenase [ubiquinone] iron-sulfur subunit, mitochondrial OS=Mus musculus GN=Sdhb PE=1 S	MOUSE	7	0.34994519	0.00701428	1.39315701	0.253512889	0.487528503	0.38018939	0.00165863	1.35518897	0.242102906	0.515228629	0.38018939	0.00916983	1.38038397	0.26546061	0.524807513
204	13.08	13.24	39.2800003	29.6400011	25.060001	sp|Q9Z2X1|H	Heterogeneous nuclear ribonucleoprotein F OS=Mus musculus GN=Hnrnpf PE=1 SV=3	MOUSE	9	0.61376202	0.1212797	1.54170001	0.349945188	0.946237087	0.51050502	0.09497619	1.75388098	0.263026804	0.895364821	0.45289761	0.03417811	1.44544005	0.242102906	0.654636085
205	13.02	13.44	23.7599999	13.5600001	10.2799997	sp|Q01149|C	Collagen alpha-2(I) chain OS=Mus musculus GN=Col1a2 PE=2 SV=2	MOUSE	13	1.70608199	0.57732362	1.81970096	0.937561989	3.944572926	1.40604806	0.93657631	1.80301797	0.779830098	3.133285999	3.31131101	0.08095932	1.90546095	1.737800956	7.311390877
206	12.99	13.01	36.6899997	23.9800006	23.9800006	sp|P19324|SE	Serpin H1 OS=Mus musculus GN=Serpinh1 PE=1 SV=3	MOUSE	12	4.7424202	0.09201261	1.90546095	2.488857031	15.27565956	5.49540901	0.04984071	2.07014108	2.654606104	15.55965996	7.37904215	0.1049461	1.99526203	3.698282003	21.28138924
207	12.95	12.96	88.3300006	68.3300018	40.5600011	sp|P11588|M	Major urinary protein 1 OS=Mus musculus GN=Mup1 PE=1 SV=1	MOUSE	13	4.61317587	0.00163548	1.99526203	2.312064886	9.375619888	5.345644	0.00130312	1.97696996	2.703958035	11.69499016	15.4169998	8.09E-06	1.75388098	8.790225029	38.01893997
207	0	11.31	84.4399989	64.4400001	36.6699994	sp|P02762|M	Major urinary protein 6 OS=Mus musculus GN=Mup6 PE=1 SV=2	MOUSE	12															
207	0	11.25	75.5599976	55.5599988	36.6699994	sp|P11589|M	Major urinary protein 2 OS=Mus musculus GN=Mup2 PE=1 SV=1	MOUSE	12															
207	0	10.97	88.0800009	72.8500009	43.7099993	sp|P04938|M	Major urinary proteins 11 and 8 (Fragment) OS=Mus musculus GN=Mup8 PE=1 SV=1	MOUSE	12															
208	12.9	13.13	49.7500002	47.2600013	47.2600013	sp|P11352|G	Glutathione peroxidase 1 OS=Mus musculus GN=Gpx1 PE=1 SV=2	MOUSE	10	0.62517268	0.22411139	1.247383	0.474242002	0.779830098	0.52480751	0.27682921	1.35518897	0.383707315	0.711213529	0.44463131	0.0683174	1.43218803	0.299226493	0.636795521
209	12.81	13.03	29.3500006	6.44799992	5.28099984	sp|Q61292|L	Laminin subunit beta-2 OS=Mus musculus GN=Lamb2 PE=2 SV=2	MOUSE	9	1.67494297	0.1979205	1.61435902	1.037528038	4.325138092	1.870682	0.1168539	1.73780096	1.076465011	4.207265854	2.60615301	0.04368323	1.77010906	1.472311974	6.08134985
210	12.8	12.85	67.4600005	45.5599994	40.2399987	sp|P19783|C	Cytochrome c oxidase subunit 4 isoform 1, mitochondrial OS=Mus musculus GN=Cox4i1 PE=1 SV=2	MOUSE	9	0.73790431	0.46578619	1.28233099	0.55462569	0.946237087	0.49659231	0.1049469	1.41905701	0.27289781	0.704693079	0.82413811	0.29776931	1.23594701	0.625172675	1.018591046
211	12.71	12.71	84.9600017	58.6499989	49.6199995	sp|P11404|F	Fatty acid-binding protein, heart OS=Mus musculus GN=Fabp3 PE=1 SV=5	MOUSE	13	1.08642602	0.8774311	1.158777	0.937561989	1.28233099	0.8472274	0.92404449	1.22461605	0.691830993	1.037528038	0.2398833	0.04616475	1.599558	0.162929595	0.383707315
212	12.62	12.68	57.0200026	38.7199998	31.9099993	sp|O08585|C	Clathrin light chain A OS=Mus musculus GN=Clta PE=1 SV=1	MOUSE	16	2.26986504	0.02481689	1.40604806	1.614359021	3.53183198	2.14783001	0.04368211	1.39315701	1.541700006	3.311311007	1.64437199	0.07071568	1.36772895	1.202263951	2.249054909
213	12.5	12.55	46.5299994	24.1699994	15.7100007	sp|Q99L45|IF	Eukaryotic translation initiation factor 2 subunit 2 OS=Mus musculus GN=Eif2s2 PE=1 SV=1	MOUSE	7	0.97274721	0.57691199	1.58489299	0.613762021	1.541700006	1.20226395	0.51687568	1.55596602	0.772680581	1.870682001	1.23594701	0.392625	1.55596602	0.794328213	1.923092008
214	12.46	25.63	65.140003	37.6800001	25.6999999	sp|P58771|T	Tropomyosin alpha-1 chain OS=Mus musculus GN=Tpm1 PE=1 SV=1	MOUSE	22	0.4325138	0.05092121	1.47231197	0.277971298	0.636795521	0.31915379	0.02686186	1.54170001	0.151356101	0.492039502	0.474242	0.06268422	1.43218803	0.319153786	0.679203629
215	12.46	12.6	20.5799997	10.8000003	9.674	sp|P28271|A	Cytoplasmic aconitate hydratase OS=Mus musculus GN=Aco1 PE=1 SV=3	MOUSE	8	0.44055489	0.01734526	1.41905701	0.293765008	0.625172675	0.44055489	0.01314447	1.31825697	0.313328594	0.580764413	0.42461959	0.02437451	1.45881403	0.270395786	0.619441092
216	12.45	12.46	68.1500018	59.2599988	59.2599988	sp|P52760|U	Ribonuclease UK114 OS=Mus musculus GN=Hrsp12 PE=1 SV=3	MOUSE	18	1.11686301	0.84737551	1.22461605	0.912010789	1.393157005	1.01859105	0.89835399	1.30617094	0.779830098	1.330453992	0.98174793	0.88116098	1.28233099	0.765596628	1.258924961
217	12.4	12.4	63.1099999	53.2800019	48.3599991	sp|P05366|S	Serum amyloid A-1 protein OS=Mus musculus GN=Saa1 PE=2 SV=2	MOUSE	10	4.4463129	0.43677649	4.78630114	0.928966403	23.33457947	5.19996023	0.43269929	5.29663515	0.981747925	22.90867996	12.8233099	0.1170107	5.05824709	2.53512907	48.75284958
218	12.38	12.38	38.2200003	17.1800003	12.3599999	sp|P56959|F	RNA-binding protein FUS OS=Mus musculus GN=Fus PE=2 SV=1	MOUSE	7	3.46736908	0.00147379	1.75388098	1.976969957	8.790225029	2.72897792	0.00741977	1.72186899	1.584892988	7.04693079	2.46603894	0.0282441	1.65958703	1.485936046	4.742420197
219	12.35	12.4	42.7899987	20.9299997	17.9100007	sp|P05202|A	Aspartate aminotransferase, mitochondrial OS=Mus musculus GN=Got2 PE=1 SV=1	MOUSE	9	0.43651581	0.01358297	1.29419601	0.334194988	0.564936996	0.35974929	0.01967841	1.28233099	0.251188606	0.461317599	0.52480751	0.01569691	1.19124198	0.420726597	0.625172675
220	12.29	12.33	96.4299977	86.9000018	81.5500021	sp|Q9D3D9|A	ATP synthase subunit delta, mitochondrial OS=Mus musculus GN=Atp5d PE=1 SV=1	MOUSE	23	1.28233099	0.45928189	1.19124198	1.076465011	1.599557996	1.14815402	0.55164939	1.22461605	0.937561989	1.419057012	0.46989411	0.06653616	1.43218803	0.299226493	0.672976673
221	12.26	12.27	48.0800003	42.3099995	38.4600014	sp|P62983|R	Ubiquitin-40S ribosomal protein S27a OS=Mus musculus GN=Rps27a PE=1 SV=2	MOUSE	10	1.48593605	0.1842262	1.29419601	1.14815402	1.976969957	1.22461605	0.4345535	1.28233099	0.954992592	1.614359021	1.158777	0.48721579	1.39315701	0.831763685	1.584892988
221	0	12.09	66.4099991	51.5600026	46.8800008	sp|P62984|R	Ubiquitin-60S ribosomal protein L40 OS=Mus musculus GN=Uba52 PE=1 SV=2	MOUSE	10															
221	0	12.09	83.6499989	82.4299991	75.0699997	sp|P0CG50|U	Polyubiquitin-C OS=Mus musculus GN=Ubc PE=1 SV=2	MOUSE	10															
221	0	12.09	86.559999	86.559999	78.6899984	sp|P0CG49|U	Polyubiquitin-B OS=Mus musculus GN=Ubb PE=1 SV=1	MOUSE	10															

222	12.23	12.27	45.9600002	43.4300005	35.3500009	sp|Q61171|PI	Peroxiredoxin-2 OS=Mus musculus GN=Prdx2 PE=1 SV=3	MOUSE	8	0.73113912	0.07967689	1.27057397	0.570164323	0.928966403	0.87096357	0.54987139	1.21338904	0.711213529	1.056818008	0.64268768	0.02843435	1.25892496	0.48305881	0.809095919
223	12.2	12.2	32.8599989	28.5699993	28.5699993	sp|P19157|GS	Glutathione S-transferase P 1 OS=Mus musculus GN=Gstp1 PE=1 SV=2	MOUSE	11	0.76559663	0.48079059	1.23594701	0.613762021	0.946237087	0.63679552	0.07487462	1.23594701	0.496592313	0.787045777	0.53951061	0.0540731	1.25892496	0.405508488	0.679203629
224	12.17	12.17	49.1699994	28.61	25.7699996	sp|Q9D964|G	Glycine amidinotransferase, mitochondrial OS=Mus musculus GN=Gatm PE=1 SV=1	MOUSE	9	0.3767038	0.00100535	1.41905701	0.207014099	0.534564376	0.1584893	0.00083548	2.42102909	0.072443597	0.383707315	0.2089296	0.00022945	1.85353196	0.075857759	0.387257606
225	12.14	12.48	23.7399995	7.3710002	6.02400005	sp|Q8BL66|E	Early endosome antigen 1 OS=Mus musculus GN=Eea1 PE=2 SV=2	MOUSE	12	1.10662401	0.41347531	1.27057397	0.870963573	1.406048059	1	0.77728301	1.34276497	0.744732022	1.342764974	0.97274721	0.81253278	1.48593605	0.654636085	1.445440054
226	12.13	12.13	51.3599992	43.1899995	41.6299999	sp|Q9CQ60|6	6-phosphogluconolactonase OS=Mus musculus GN=Pgls PE=2 SV=1	MOUSE	9	0.91201079	0.72556758	1.599558	0.570164323	1.458814025	0.94623709	0.94414771	1.57036305	0.602559626	1.485936046	0.95499259	0.95024377	1.33045399	0.717794299	1.270573974
227	12	12	50.7300019	33.9399993	31.0200006	sp|Q9CR68|U	Cytochrome b-c1 complex subunit Rieske, mitochondrial OS=Mus musculus GN=Uqcrfs1 PE=1 SV=1	MOUSE	11	1.14815402	0.463101	1.55596602	0.73790431	1.786488056	1.07646501	0.41113141	1.55596602	0.691830993	1.67494297	0.87096357	0.3274532	1.40604806	0.619441092	1.224616051
228	11.99	12.08	41.2400007	21.4399993	13.2599995	sp|Q8BGD9|I	Eukaryotic translation initiation factor 4B OS=Mus musculus GN=Eif4b PE=1 SV=1	MOUSE	6	4.05508518	0.1188871	2.39883304	1.690441012	6.08134985	2.67916799	0.1908192	2.10862804	1.270573974	3.80189395	0.97274721	0.56774747	1.39315701	0.672976673	1.355188966
229	11.92	11.96	30.8200002	22.1499994	19.9000001	sp|P40142|TK	Transketolase OS=Mus musculus GN=Tkt PE=1 SV=1	MOUSE	9	1.01859105	0.874825	1.36772895	0.744732022	1.393157005	0.82413811	0.328152	1.43218803	0.564936996	1.180320978	1.19124198	0.2786555	1.45881403	0.816582382	1.737800956
230	11.88	12.04	42.0599997	18.8800007	14.0200004	sp|Q8VIJ6|SF	Splicing factor, proline- and glutamine-rich OS=Mus musculus GN=Sfpq PE=1 SV=1	MOUSE	8	1.55596602	0.4154827	1.40604806	1.106624007	2.208004951	1.57036305	0.26570422	1.40604806	1.116863012	2.249054909	1.34276497	0.42736709	1.38038397	0.972747207	1.853531957
231	11.81	11.89	52.2300005	42.8600013	42.8600013	sp|O08709|PI	Peroxiredoxin-6 OS=Mus musculus GN=Prdx6 PE=1 SV=3	MOUSE	13	0.4875285	0.124941	1.33045399	0.2558586	0.648634374	0.2964831	0.04194792	1.51356101	0.129419595	0.4487454	0.19408859	0.07576755	1.78648806	0.058613818	0.346736789
232	11.8	11.8	82.0200026	80.9000015	66.2899971	sp|P99028|Q	Cytochrome b-c1 complex subunit 6, mitochondrial OS=Mus musculus GN=Uqcrh PE=1 SV=2	MOUSE	28	3.133286	0.03936512	1.35518897	2.312064886	4.285484791	2.83139205	0.07666831	1.34276497	2.108628035	3.80189395	1.73780096	0.25129139	1.31825697	1.318256974	2.167704105
233	11.68	11.68	38.350001	15.4699996	13.7700006	sp|P17563|SE	Selenium-binding protein 1 OS=Mus musculus GN=Selenbp1 PE=1 SV=2	MOUSE	9	0.44463131	0.01828539	1.33045399	0.325087309	0.591561615	0.4325138	0.02964901	1.40604806	0.299226493	0.608134985	0.89536482	0.66599548	1.29419601	0.698232412	1.158776999
233	0	10.47	34.5299989	12.7100006	11.0200003	sp|Q63836|SI	Selenium-binding protein 2 OS=Mus musculus GN=Selenbp2 PE=1 SV=2	MOUSE	7															
234	11.64	11.82	60.8900011	56.4400017	53.7800014	sp|O70251|EI	Elongation factor 1-beta OS=Mus musculus GN=Eef1b PE=1 SV=5	MOUSE	11	0.564937	0.2719177	1.67494297	0.349945188	0.946237087	0.83176369	0.34200901	1.29419601	0.630957425	1.076465011	0.7177943	0.31586951	1.38038397	0.501187205	0.990831971
235	11.55	11.55	48.0100006	20.4899997	17.7399993	sp|O35459|E	Delta(3,5)-Delta(2,4)-dienoyl-CoA isomerase, mitochondrial OS=Mus musculus GN=Ech1 PE=2 SV=1	MOUSE	6	0.3221069	0.02854247	1.43218803	0.203235701	0.461317599	0.64863437	0.15985461	1.27057397	0.496592313	0.824138105	0.61944109	0.13212921	1.27057397	0.469894111	0.787045777
236	11.47	11.88	40.6800002	26.7800003	25.4200012	sp|P14206|RS	40S ribosomal protein SA OS=Mus musculus GN=Rpsa PE=1 SV=4	MOUSE	8	0.69823241	0.4253085	1.35518897	0.510505021	0.946237087	0.79432821	0.48412749	1.29419601	0.586138189	1.028015971	1.04712904	0.29621521	1.31825697	0.794328213	1.393157005
237	11.4	11.7	43.6199993	25.5199999	18.9899996	sp|Q93092|TA	Transaldolase OS=Mus musculus GN=Taldo1 PE=1 SV=2	MOUSE	10	0.73790431	0.28049511	1.40604806	0.501187205	1.037528038	1.01859105	0.91400188	1.19124198	0.855066717	1.202263951	1.20226395	0.1830198	1.18032098	1.018591046	1.485936046
238	11.4	11.4	33.4699988	22.5299999	16.4199993	sp|Q9D1A2|C	Cytosolic non-specific dipeptidase OS=Mus musculus GN=Cndp2 PE=1 SV=1	MOUSE	7	0.57016432	0.31764439	1.27057397	0.452897608	0.724435985	0.67297667	0.16861039	1.247383	0.524807513	0.839460015	0.7798301	0.36310881	1.18032098	0.654636085	0.920449615
239	11.37	11.46	44.5600003	26.0800004	19.4900006	sp|P06728|AI	Apolipoprotein A-IV OS=Mus musculus GN=Apoa4 PE=2 SV=3	MOUSE	9	6.2517271	0.03628016	1.73780096	3.597492933	12.70573997	6.30957413	0.01797178	1.88799095	3.34194994	15.13560963	4.96592283	0.07266401	1.69044101	2.937649965	11.58777046
240	11.33	11.33	84.5700026	56.5699995	50.2900004	sp|Q9CXZ1|N	NADH dehydrogenase [ubiquinone] iron-sulfur protein 4, mitochondrial OS=Mus musculus GN=Ndufs4 PE=1 S	MOUSE	16	1.22461605	0.1719434	1.25892496	0.972747207	1.55596602	1.01859105	0.69722217	1.247383	0.816582382	1.29419601	0.39445731	0.1014948	1.29419601	0.291071713	0.510505021
241	11.3	23.19	42.3700005	27.6199997	21.7800006	sp|P26043|RA	Radixin OS=Mus musculus GN=Rdx PE=1 SV=3	MOUSE	22	1.27057397	0.3496823	1.30617094	0.972747207	1.706081986	1.49968505	0.06626764	1.34276497	1.116863012	2.013724089	1.158777	0.82482398	1.31825697	0.879022479	1.527565956
242	11.28	13.36	96.7299998	64.0500009	60.1300001	sp|O89086|RI	Putative RNA-binding protein 3 OS=Mus musculus GN=Rbm3 PE=1 SV=1	MOUSE	12	1.158777	0.81290031	1.33045399	0.870963573	1.541700006	1.55596602	0.2340685	1.247383	1.247382998	2.249054909	2.18776202	0.24192689	1.35518897	1.614359021	3.162277937
243	11.28	11.28	71.1899996	54.2400003	54.2400003	sp|Q9CR51|V	V-type proton ATPase subunit G 1 OS=Mus musculus GN=Atp6v1g1 PE=2 SV=3	MOUSE	14	1.62929595	0.1284852	1.40604806	1.158776999	2.421029091	1.77010906	0.1508542	1.36772895	1.29419601	2.606153011	1.22461605	0.17800599	1.51356101	0.809095919	1.853531957
244	11.26	11.43	48.4299988	31.099999	28.7400007	sp|Q9DCM0|E	Protein ETHE1, mitochondrial OS=Mus musculus GN=Ethe1 PE=1 SV=2	MOUSE	8	0.98174793	0.96382701	1.158777	0.839460015	1.137627006	0.82413811	0.45587	1.158777	0.698232412	0.954992592	0.39445731	0.09405561	1.41905701	0.233345807	0.55975759
245	11.16	24.54	55.2299976	35.7300013	27.1499991	sp|Q61696|HS	Heat shock 70 kDa protein 1A OS=Mus musculus GN=Hspa1a PE=1 SV=2	MOUSE	21	0.65463609	0.80065769	1.35518897	0.48305881	0.88715601	0.96382898	0.89608192	1.43218803	0.711213529	1.380383968	1.25892496	0.3668448	1.36772895	0.920449615	1.753880978
245	0	24.54	58.099997	35.6700003	27.0999998	sp|P17879|HS	Heat shock 70 kDa protein 1B OS=Mus musculus GN=Hspa1b PE=1 SV=3	MOUSE	21															
246	11.14	11.43	20.0000003	8.07700008	6.53799996	sp|P13020|GI	Gelsolin OS=Mus musculus GN=Gsn PE=1 SV=3	MOUSE	5	1.67494297	0.08341516	1.45881403	1.14815402	3.34194994	1.20226395	0.99378109	1.51356101	0.794328213	2.187762022	2.3550489	0.00668549	1.54170001	1.527565956	4.325138092
247	11.09	11.42	30.4399997	22.4800006	20.3500003	sp|Q8VCT4|C	Carboxylesterase 3 OS=Mus musculus GN=Ces3 PE=1 SV=1	MOUSE	10	1.11686301	0.41122079	1.58489299	0.704693079	1.786488056	0.51050502	0.47086161	2.10862804	0.2558586	1.076465011	1.05681801	0.54789442	1.58489299	0.666806817	1.690441012
248	11.05	13.15	74.849999	44.1700012	44.1700012	sp|Q9D0S9|H	Histidine triad nucleotide-binding protein 2, mitochondrial OS=Mus musculus GN=Hint2 PE=2 SV=1	MOUSE	8	1.11686301	0.29285881	1.61435902	0.691830993	1.786488056	1.16949904	0.40481871	1.47231197	0.794328213	1.753880978	1.247383	0.3708846	1.47231197	0.847227395	1.870682001
249	11.03	12.62	33.2199991	22.1499994	18.7900007	sp|Q91VR2|A	ATP synthase subunit gamma, mitochondrial OS=Mus musculus GN=Atp5c1 PE=1 SV=1	MOUSE	7	0.72443599	0.1361527	1.16949904	0.608134985	0.847227395	0.61376202	0.248208	1.69044101	0.363078088	1.037528038	0.60255963	0.04610514	1.47231197	0.405508488	0.88715601
250	11.01	11.01	33.039999	18.9700007	17.4099997	sp|Q06890|CI	Clusterin OS=Mus musculus GN=Clu PE=1 SV=1	MOUSE	10	2.22843504	0.12942091	1.54170001	1.445440054	3.630779982	3.1045599	0.02472907	1.57036305	1.976969957	5.445025921	6.60693502	0.00052648	1.599558	4.130475044	12.13389015
251	10.99	10.99	31.310001	21.0600004	18.9799994	sp|P17225|PT	Polypyrimidine tract-binding protein 1 OS=Mus musculus GN=Ptbp1 PE=1 SV=2	MOUSE	12	0.81658238	0.70360518	20.3235703	0.046989411	16.59586906	1.41905701	0.53230488	4.61317587	0.307609707	6.546361923	0.86297852	0.79230249	2.10862804	0.413047493	1.819700956
252	10.96	10.96	45.7700014	25.0800014	16.9300005	sp|P48036|AI	Annexin A5 OS=Mus musculus GN=Anxa5 PE=1 SV=1	MOUSE	6	0.80167812	0.54555428	1.27057397	0.630957425	1.018591046	0.53456438	0.242681	1.36772895	0.405508488	0.731139123	1.57036305	0.44221771	1.31825697	1.19124198	2.147830009
253	10.77	10.77	35.2699995	20.6499994	16.7699993	sp|P97855|G3	Ras GTPase-activating protein-binding protein 1 OS=Mus musculus GN=G3bp1 PE=1 SV=1	MOUSE	6	3.49945211	0.05381428	1.88799095	1.853531957	6.025596142	2.48885703	0.2815983	1.75388098	1.419057012	4.246195793	3.59749293	0.04793142	1.92309201	1.870682001	6.918310165
254	10.74	10.78	38.6099994	19.6199998	17.5099999	sp|P51855|GS	Glutathione synthetase OS=Mus musculus GN=Gss PE=2 SV=1	MOUSE	6	0.62517268	0.2532675	1.80301797	0.337287307	1.127197981	0.31915379	0.2466443	1.95884502	0.162929595	0.625172675	0.50582469	0.2226209	1.85353196	0.263026804	0.937561989
255	10.73	10.73	66.2299991	49.3499994	49.3499994	sp|P63242|IF	Eukaryotic translation initiation factor 5A-1 OS=Mus musculus GN=Eif5a PE=1 SV=2	MOUSE	16	0.64268768	0.4222742	1.55596602	0.373250186	1	0.57016432	0.21995629	1.54170001	0.366437614	0.879022479	0.61944109	0.2257614	1.43218803	0.376703799	0.88715601
256	10.72	10.76	22.7300003	17.0499995	15.9099996	sp|Q922R8|P	Protein disulfide-isomerase A6 OS=Mus musculus GN=Pdia6 PE=1 SV=3	MOUSE	8	0.61944109	0.1786228	1.38038397	0.432513803	0.855066717	0.7177943	0.62644142	1.43218803	0.496592313	1.028015971	1	0.72172362	1.19124198	0.839460015	1.19124198
257	10.69	10.69	47.8100002	24.0400001	20.4899997	sp|Q9D6R2|II	Isocitrate dehydrogenase [NAD] subunit alpha, mitochondrial OS=Mus musculus GN=Idh3a PE=1 SV=1	MOUSE	8	1.13762701	0.6414296	1.30617094	0.870963573	1.527565956	0.92044962	0.83423013	1.33045399	0.691830993	1.224616051	1.11686301	0.9529531	1.31825697	0.847227395	1.499685049
258	10.68	10.68	50.9599984	34.0999991	26.4400005	sp|Q64105|SI	Sepiapterin reductase OS=Mus musculus GN=Spr PE=1 SV=1	MOUSE	10	0.70469308	0.34433499	1.39315701	0.469894111	0.981747925	0.69183099	0.56531799	1.45881403	0.380189389	1.009253025	0.34673679	0.06395597	1.44544005	0.2013724	0.501187205
259	10.67	10.67	65.3199971	40.7299995	27.0200014	sp|Q9WUK2|I	Eukaryotic translation initiation factor 4H OS=Mus musculus GN=Eif4h PE=1 SV=3	MOUSE	7	0.95499259	0.487225	1.31825697	0.724435985	1.258924961	0.9289664	0.51749033	1.33045399	0.704693079	1.235947013	0.67920363	0.25932121	1.33045399	0.510505021	0.90364939
260	10.6	10.6	35.710001	22.6699993	19.2499995	sp|Q99JR1|S	Sideroflexin-1 OS=Mus musculus GN=Sfxn1 PE=1 SV=3	MOUSE	5	0.76559663	0.26451021	1.48593605	0.510505021	1.137627006	0.73113912	0.07345379	1.45881403	0.469894111	1.066596031	0.91201079	0.26890791	1.48593605	0.608134985	1.355188966
261	10.59	10.68	51.4599979	36.3999993	29.7100008	sp|Q9WTP6|K	Adenylate kinase 2, mitochondrial OS=Mus musculus GN=Ak2 PE=1 SV=5	MOUSE	15	0.63095743	0.27833861	1.31825697	0.4487454	0.831763685	0.67920363	0.32956579	1.31825697	0.496592313	0.895364821	0.42854852	0.07987566	1.39315701	0.304789513	0.597035289
262	10.55	10.55	25	11.93	11.2199999	sp|Q61847|M	Meprin A subunit beta OS=Mus musculus GN=Mep1b PE=1 SV=2	MOUSE	8	0.69823241	0.1285737	1.16949904	0.597035289	0.816582382	0.60813499	0.1044723	1.36772895	0.436515808	0.831763685	0.44463131	0.04315883	1.41905701	0.313328594	0.630957425
263	10.53	10.58	41.7600006	32.3900014	26.1400014	sp|Q9JHJ0|TI	Tropomodulin-3 OS=Mus musculus GN=Tmod3 PE=1 SV=1	MOUSE	19	2.53512907	0.26015231	1.62929595	1.55596602	4.786301136	1.99526203	0.237177	1.58489299	1.258924961	3.872575998	2.26986504	0.37770659	1.599558	1.419057012	3.908409119
264	10.5	11.6	22.4399999	7.93699995	4.25000005	sp|Q6NZJ6|IF	Eukaryotic translation initiation factor 4 gamma 1 OS=Mus musculus GN=Eif4g1 PE=1 SV=1	MOUSE	8	1.51356101	0.4477903	1.54170001	0.981747925	2.488857031	1.78648806	0.204124	1.52756596	1.16949904	2.937649965	1.61435902	0.32799199	1.55596602	1.037528038	2.558585882
265	10.49	11.7	30.5299997	20.6699997	20.6699997	sp|P54728|RI	UV excision repair protein RAD23 homolog B OS=Mus musculus GN=Rad23b PE=1 SV=2	MOUSE	9	1.25892496	0.13815109	1.35518897	0.928966403	1.753880978	1.41905701	0.09048562	1.21338904	1.16949904	1.870682001	0.9289664	0.82856512	1.22461605	0.613762021	1.137627006
266	10.43	10.46	34.2000008	28.5699993	24.6800005	sp|P10126|EF	Elongation factor 1-alpha 1 OS=Mus musculus GN=Eef1a1 PE=1 SV=3	MOUSE	14	0.64268768	0.1684881	1.27057397	0.48305881	0.816582382	0.66680682	0.15297461	1.29419601	0.496592313	0.862978518	1.158777	0.69577187	1.23594701	0.937561989	1.472311974
267	10.4	10.6	22.9800001	9.83399972	8.38499963	sp|P97449|AI	Aminopeptidase N OS=Mus musculus GN=Anpep PE=1 SV=4	MOUSE	7	0.75857759	0.1047316	1.33045399	0.564936996	1.009253025	0.55975759	0.224494	1.62929595	0.299226493	0.912010789	0.64863437	0.03152279	1.22461605	0.515228629	0.794328213
268	10.38	10.38	39.1600013	27.0000011	24.7099996	sp|Q61792|LA	LIM and SH3 domain protein 1 OS=Mus musculus GN=Lasp1 PE=1 SV=1	MOUSE	6	2.18776202	0.07255757	1.35518897	1.614359021	3.28095293	2.16770411	0.1138942	1.35518897	1.599557996	3.162277937	1.55596602	0.2454845	1.31825697	1.180320978	2.089296103
269	10.35	10.36	48.5599995	19.8100001	17.8900003	sp|Q8K1Z0|C	Ubiquinone biosynthesis protein COQ9, mitochondrial OS=Mus musculus GN=Coq9 PE=1 SV=1	MOUSE	8	0.87902248	0.79125691	1.31825697	0.660693526	1.158776999	0.83176369	0.76294392	1.47231197	0.55975759	1.224616051	0.61376202	0.387725	1.52756596	0.398107201	0.937561989
270	10.34	10.6	20.4699993	17.7699998	7.10700005	sp|P35564|CA	Calnexin OS=Mus musculus GN=Canx PE=1 SV=1	MOUSE	6	1.45881403	0.59810919	1.54170001	0.946237087	2.333457947	1.28233099	0.67761099	1.48593605	0.862978518	2.032356977	2.37684012	0.1132198	1.58489299	1.499685049	3.908409119
271	10.28	10.46	31.5299988	9.89599973	8.9759998	sp|Q9WU78|P	Programmed cell death 6-interacting protein OS=Mus musculus GN=Pdcd6ip PE=1 SV=3	MOUSE	8	0.99083197	0.91786939	1.16949904	0.847227395	1.158776999	0.97274721	0.64513671	1.14815402	0.847227395	1.116863012	1.40604806	0.01503681	1.28233099	1.096477985	1.803017974
272	10.23	30.75	58.3899975	47.9900003	44.63	sp|P48962|AI	ADP/ATP translocase 1 OS=Mus musculus GN=Slc25a4 PE=1 SV=4	MOUSE	22	0.69823241	0.30040491	1.47231197	0.452897608	1.028015971	0.76559663	0.30682191	1.69044101	0.457088202	1.29419601	0.67920363	0.24348719	1.58489299	0.40926069	1.076465011
273	10.21	10.3	28.9600015	8.28799978	6.88899979	sp|Q9WV92|E	Band 4.1-like protein 3 OS=Mus musculus GN=Epb41l3 PE=1 SV=1	MOUSE	8	1.47231197	0.06829703	1.29419601	1.137627006	1.958845019	1.33045399	0.2011734	1.38038397	0.963828981	1.853531957	0.4487454	0.27782911	1.72186899	0.263026804	0.772680581
274	10.2	10.2	33.8099986	15.7100007	10.0699998	sp|Q62318|TI	Transcription intermediary factor 1-beta OS=Mus musculus GN=Trim28 PE=1 SV=3	MOUSE	6	5.15228701	0.0733645	2.33345795	2.208004951	12.58924961	5.05824709	0.01831138	2.42102909	2.089296103	13.18257046	4.6558609	0.1047101	2.42102909	1.923092008	11.2719698
275	10.12	10.12	47.330001	24.6900007	21.8099996	sp|P40630|TF	Transcription factor A, mitochondrial OS=Mus musculus GN=Tfam PE=1 SV=2	MOUSE	6	1.39315701	0.1488211	1.25892496	1.106624007	1.770109057	0.87902248	0.4304705	1.36772895	0.648634374	1.202263951	0.73113912	0.19448671	1.21338904	0.597035289	0.88715601
276	10.1	10.1	67.3300028	59.4099999	52.4800003	sp|Q9D0J8|P	Parathymosin OS=Mus musculus GN=Ptms PE=2 SV=3	MOUSE	13	2.24905491	0.21387319	1.43218803	1.570363045	5.445025921	2.0892961	0.2389383	1.40604806	1.485936046	5.445025921	1.88799095	0.39951009	1.47231197	1.28233099	4.285484791
277	10.09	10.34	24.8799995	5.50399981	5.50399981	sp|Q62417|SI	Sorbin and SH3 domain-containing protein 1 OS=Mus musculus GN=Sorbs1 PE=1 SV=2	MOUSE	6	4.40554905	0.1940286	1.92309201	2.290868044	9.462371826	4.78630114	0.07490477	1.95884502	2.443430901	11.06624031	4.7424202	0.06620492	1.95884502	2.421029091	10.37528038
278	10.08	10.08	72.1899986	50.2699971	45.9899992	sp|P70296|PE	Phosphatidylethanolamine-binding protein 1 OS=Mus musculus GN=Pebp1 PE=1 SV=3	MOUSE	14	0.88715601	0.33782491	1.41905701	0.630957425	1.258924961	0.81658238	0.38634399	1.47231197	0.549540877	1.202263951	0.2754229	0.05192602	1.57036305	0.1786488	0.432513803
279	10.07	10.07	40.5699998	25.2999991	18.1400001	sp|P35505|FA	Fumarylacetoacetase OS=Mus musculus GN=Fah PE=1 SV=2	MOUSE	10	0.73790431	0.19204269	1.22461605	0.597035289	0.90364939	0.50582469	0.03611004	1.45881403	0.334194988	0.73790431	0.74473202	0.2802276	1.28233099	0.57543987	0.954992592
280	10.01	10.72	34.77	16.2000001	12.5300005	sp|Q9Z2I9|SL	Succinyl-CoA ligase [ADP-forming] subunit beta, mitochondrial OS=Mus musculus GN=Sucla2 PE=1 SV=2	MOUSE	8	0.88715601	0.46553609	1.29419601	0.685488224	1.14815402	0.7177943	0.16818181	1.29419601	0.544502676	0.928966403	0.76559663	0.1648991	1.23594701	0.613762021	0.946237087
281	10.01	10.12	25.5800009	12.6900002	12.6900002	sp|Q99K28|A	ADP-ribosylation factor GTPase-activating protein 2 OS=Mus musculus GN=Arfgap2 PE=1 SV=1	MOUSE	6	1.29419601	0.20852961	1.36772895	0.946237087	1.803017974	1.20226395	0.36849371	1.40604806	0.855066717	1.706081986	0.51522863	0.45444199	1.67494297	0.310455889	0.862978518
282	10.01	10.09	38.5399997	21.6600001	19.7500005	sp|Q61425|H	Hydroxyacyl-coenzyme A dehydrogenase, mitochondrial OS=Mus musculus GN=Hadh PE=1 SV=2	MOUSE	10	0.73113912	0.2180872	1.35518897	0.539510608	0.990831971	0.60813499	0.2111579	1.39315701	0.424619585	0.847227395	0.65463609	0.2752139	1.35518897	0.474242002	0.88715601
283	10.01	10.01	68.2900012	62.1999979	60.9799981	sp|Q62425|NI	NADH dehydrogenase [ubiquinone] 1 alpha subcomplex subunit 4 OS=Mus musculus GN=Ndufa4 PE=1 SV=2	MOUSE	7	0.73113912	0.424588	1.28233099	0.564936996	0.937561989	0.51999599	0.84643871	1.57036305	0.331131101	0.816582382	0.70469308	0.57770199	1.38038397	0.501187205	0.972747207
284	10	10.03	53.1099975	41.1500007	41.1500007	sp|P14602|HS	Heat shock protein beta-1 OS=Mus musculus GN=Hspb1 PE=1 SV=3	MOUSE	7	2.18776202	0.1012091	1.55596602	1.406048059	3.40408206	2.46603894	0.1563745	1.599558	1.541700006	3.944572926	4.61317587	0.03024604	1.55596602	2.964831114	7.311390877
285	10	10	38.6999995	27.5900006	27.5900006	sp|O70439|S	Syntaxin-7 OS=Mus musculus GN=Stx7 PE=1 SV=3	MOUSE	5	1.18032098	0.03111925	1.30617094	0.90364939	1.541700006	1.14815402	0.03882434	1.28233099	0.895364821	1.472311974	1.27057397	0.0040094	1.599558	0.794328213	2.032356977
286	9.96	10.04	52.5099993	48.0399996	44.690001	sp|Q9CQJ8|N	NADH dehydrogenase [ubiquinone] 1 beta subcomplex subunit 9 OS=Mus musculus GN=Ndufb9 PE=1 SV=3	MOUSE	9	0.93756199	0.80473769	1.30617094	0.711213529	1.224616051	1.01859105	0.53043908	1.247383	0.816582382	1.270573974	0.4325138	0.03828809	1.64437199	0.246603906	0.711213529
287	9.94	10.01	34.3899995	22.1499994	17.0900002	sp|P40124|CA	Adenylyl cyclase-associated protein 1 OS=Mus musculus GN=Cap1 PE=1 SV=4	MOUSE	7	0.8472274	0.3271893	1.39315701	0.586138189	1.180320978	0.4786301	0.49326551	1.64437199	0.291071713	0.787045777	1.31825697	0.65067929	1.61435902	0.816582382	2.147830009
288	9.82	9.88	50.4999995	36.4499986	22.4099994	sp|O35129|PI	Prohibitin-2 OS=Mus musculus GN=Phb2 PE=1 SV=1	MOUSE	8	0.64863437	0.08847264	1.41905701	0.401790798	0.920449615	0.73790431	0.08383641	1.39315701	0.515228629	1.028015971	0.78704578	0.25111911	1.39315701	0.55975759	1.096477985
289	9.74	9.77	38.4900004	29.2800009	22.3700002	sp|P14094|AT	Sodium/potassium-transporting ATPase subunit beta-1 OS=Mus musculus GN=Atp1b1 PE=1 SV=1	MOUSE	9	0.96382898	0.71259707	1.20226395	0.794328213	1.158776999	0.60255963	0.09229646	1.31825697	0.376703799	0.794328213	0.42461959	0.07641041	1.65958703	0.208929598	0.704693079
290	9.68	10.21	72.2199976	52.7800024	42.5900012	sp|P97450|AT	ATP synthase-coupling factor 6, mitochondrial OS=Mus musculus GN=Atp5j PE=1 SV=1	MOUSE	18	1.95884502	0.03987896	1.30617094	1.499685049	2.654606104	1.81970096	0.01984526	1.28233099	1.419057012	2.466038942	0.81658238	0.4553948	1.27057397	0.642687678	1.037528038
291	9.67	9.68	45.0899988	25.7699996	23.0100006	sp|P70372|EI	ELAV-like protein 1 OS=Mus musculus GN=Elavl1 PE=1 SV=2	MOUSE	6	1.03752804	0.48990041	1.19124198	0.870963573	1.247382998	0.81658238	0.362506	1.36772895	0.591561615	1.116863012	0.83176369	0.31610379	1.41905701	0.591561615	1.180320978
292	9.59	9.64	32.6700002	23.1000006	23.1000006	sp|Q99MZ7|P	Peroxisomal trans-2-enoyl-CoA reductase OS=Mus musculus GN=Pecr PE=2 SV=1	MOUSE	6	0.3981072	0.1039922	1.36772895	0.251188606	0.544502676	0.31332859	0.07937564	1.47231197	0.170608193	0.461317599	0.4875285	0.162852	1.20226395	0.366437614	0.586138189
293	9.52	9.61	58.5399985	31.0900003	22.4099994	sp|P11930|NI	Nucleoside diphosphate-linked moiety X motif 19, mitochondrial OS=Mus musculus GN=Nudt19 PE=1 SV=2	MOUSE	5	0.3981072	0.01579339	1.61435902	0.242102906	0.642687678	0.51522863	0.05669935	1.57036305	0.299226493	0.809095919	0.4920395	0.1580808	1.58489299	0.288403213	0.779830098
294	9.49	9.64	38.1900013	12.9700005	12.9700005	sp|Q3ULD5|M	Methylcrotonoyl-CoA carboxylase beta chain, mitochondrial OS=Mus musculus GN=Mccc2 PE=2 SV=1	MOUSE	8	0.40550849	0.1280535	1.73780096	0.2108628	0.704693079	0.38725761	0.2211296	1.75388098	0.197696999	0.679203629	0.42461959	0.07410201	1.36772895	0.216770396	0.580764413
295	9.46	14.34	38.4999991	21.6800004	18.3599994	sp|P54071|ID	Isocitrate dehydrogenase [NADP], mitochondrial OS=Mus musculus GN=Idh2 PE=1 SV=3	MOUSE	10	0.42461959	0.00352068	1.36772895	0.275422901	0.580764413	0.4017908	0.06245529	1.44544005	0.26546061	0.580764413	0.50582469	0.01126948	1.34276497	0.363078088	0.679203629
296	9.44	9.44	18.5399994	6.81499988	6.81499988	sp|Q99KN9|E	Clathrin interactor 1 OS=Mus musculus GN=Clint1 PE=1 SV=2	MOUSE	7	1.870682	0.3032223	1.62929595	1.14815402	3.732501984	1.61435902	0.471636	1.61435902	1	3.28095293	1.70608199	0.53477329	1.62929595	1.047129035	3.162277937
297	9.4	9.57	23.59	11.5800001	9.15099978	sp|Q60902|EI	Epidermal growth factor receptor substrate 15-like 1 OS=Mus musculus GN=Eps15l1 PE=1 SV=3	MOUSE	8	1.52756596	0.09153894	1.41905701	1.076465011	2.22843504	1.73780096	0.04496348	1.41905701	1.224616051	2.53512907	1.18032098	0.2596733	1.36772895	0.862978518	1.614359021
298	9.38	15.02	54.0300012	16.11	13.7600005	sp|P21619|LM	Lamin-B2 OS=Mus musculus GN=Lmnb2 PE=1 SV=2	MOUSE	8	1.00925303	0.72803003	1.14815402	0.879022479	1.158776999	1.00925303	0.83811009	1.12719798	0.895364821	1.137627006	0.91201079	0.31165919	1.16949904	0.779830098	1.066596031
299	9.34	11.5	38.319999	25.09	17.3500001	sp|Q8R317|U	Ubiquilin-1 OS=Mus musculus GN=Ubqln1 PE=1 SV=1	MOUSE	11	1.40604806	0.18353181	1.45881403	0.963828981	2.089296103	1.19124198	0.30508271	1.57036305	0.758577585	1.887990952	0.9289664	0.59292418	1.62929595	0.549540877	1.51356101

300	9.31	9.37	50.6099999	33.5399985	28.0499995	sp|P84104|SI	Serine/arginine-rich splicing factor 3 OS=Mus musculus GN=Srsf3 PE=1 SV=1	MOUSE	7	2.29086804	0.42207471	1.69044101	1.355188966	3.80189395	2.22843504	0.40803629	1.69044101	1.318256974	3.732501984	1.29419601	0.65668279	1.62929595	0.794328213	2.089296103
301	9.29	9.33	38.1099999	20.9199995	18.9099997	sp|P13707|GI	Glycerol-3-phosphate dehydrogenase [NAD+], cytoplasmic OS=Mus musculus GN=Gpd1 PE=1 SV=3	MOUSE	6	0.4875285	0.04818416	1.25892496	0.343558013	0.613762021	0.38725761	0.05538452	1.38038397	0.267916799	0.534564376	0.52966338	0.06659386	1.25892496	0.369828194	0.666806817
302	9.19	9.29	16.7199999	11.2000004	10.2300003	sp|Q921G7|E	Electron transfer flavoprotein-ubiquinone oxidoreductase, mitochondrial OS=Mus musculus GN=Etfdh PE=1 S\	MOUSE	6	0.8472274	0.31976789	1.22461605	0.685488224	1.037528038	0.87096357	0.32060939	1.14815402	0.751622915	1	0.91201079	0.4318203	1.14815402	0.794328213	1.047129035
303	9.14	9.19	22.3000005	9.47600007	8.80699977	sp|P42567|EI	Epidermal growth factor receptor substrate 15 OS=Mus musculus GN=Eps15 PE=1 SV=1	MOUSE	6	1.27057397	0.8324551	1.70608199	0.744732022	2.22843504	1.67494297	0.38722739	1.72186899	0.972747207	2.992264986	1.23594701	0.869744	1.65958703	0.744732022	2.187762022
304	9.13	9.25	32.7499986	23.77	17.1000004	sp|P29699|FE	Alpha-2-HS-glycoprotein OS=Mus musculus GN=Ahsg PE=1 SV=1	MOUSE	7	1.39315701	0.48016691	1.41905701	0.981747925	4.487453938	1.77010906	0.29126239	1.75388098	1.009253025	5.546257019	3.46736908	0.06962069	2.16770411	1.599557996	11.2719698
305	9.11	9.11	20.4799995	5.78299984	3.85500006	sp|P10493|NI	Nidogen-1 OS=Mus musculus GN=Nid1 PE=1 SV=2	MOUSE	6	0.9289664	0.80927408	1.45881403	0.660693526	1.355188966	1.05681801	0.56144011	1.45881403	0.724435985	1.629295945	1.75388098	0.06916002	1.22461605	1.432188034	3.435580015
306	9.05	9.1	37.3899996	21.6199994	21.6199994	sp|Q9CR00|F	26S proteasome non-ATPase regulatory subunit 9 OS=Mus musculus GN=Psmd9 PE=1 SV=1	MOUSE	7	1.48593605	0.40037951	1.67494297	0.88715601	2.606153011	1.69044101	0.35571811	1.65958703	1.018591046	2.937649965	0.89536482	0.60988128	1.62929595	0.549540877	1.458814025
307	8.99	9.13	27.6499987	10.2600001	6.37099966	sp|Q99K67|A	Alpha-aminoadipic semialdehyde synthase, mitochondrial OS=Mus musculus GN=Aass PE=2 SV=1	MOUSE	6	0.49659231	0.0248173	1.33045399	0.356451094	0.660693526	0.52966338	0.2782535	1.599558	0.337287307	0.847227395	0.81658238	0.42324451	1.22461605	0.660693526	1
308	8.98	8.98	41.2200004	26.6900003	24.3200004	sp|Q60932|VI	Voltage-dependent anion-selective channel protein 1 OS=Mus musculus GN=Vdac1 PE=1 SV=3	MOUSE	8	0.44055489	0.07832092	1.41905701	0.301995188	0.625172675	0.38018939	0.01662119	1.43218803	0.263026804	0.544502676	0.46989411	0.03263283	1.41905701	0.310455889	0.666806817
309	8.97	10.51	22.8799999	13.5199994	12.1799998	sp|P14824|AI	Annexin A6 OS=Mus musculus GN=Anxa6 PE=1 SV=3	MOUSE	7	0.77268058	0.42921439	1.58489299	0.48305881	1.224616051	0.9289664	0.44764641	1.58489299	0.597035289	1.472311974	1.43218803	0.54234558	1.69044101	0.847227395	2.443430901
310	8.92	8.92	65.7199979	27.8400004	12.1100001	sp|Q91VM5|F	Heterogeneous nuclear ribonucleoprotein G-like 1 OS=Mus musculus GN=Rbmxl1 PE=2 SV=1	MOUSE	5	1.64437199	0.2510154	1.61435902	1.018591046	2.703958035	1.64437199	0.06044684	1.64437199	1	3.221069098	1.69044101	0.05323197	1.58489299	1.066596031	3.435580015
310	0	6.62	60.3100002	24.2300004	8.50500017	sp|O35479|HI	Heterogeneous nuclear ribonucleoprotein G OS=Mus musculus GN=Rbmx PE=1 SV=1	MOUSE	4															
311	8.85	8.89	30.6300014	12.5	10.2499999	sp|Q8VEK3|H	Heterogeneous nuclear ribonucleoprotein U OS=Mus musculus GN=Hnrnpu PE=1 SV=1	MOUSE	11	1.870682	0.90611339	1.90546095	0.981747925	4.092607021	1.88799095	0.4249582	1.67494297	1.127197981	4.446312904	2.0892961	0.3966504	1.88799095	1.106624007	4.365159035
312	8.82	9.03	62.4199987	59.0600014	54.3600023	sp|P62204|C/	Calmodulin OS=Mus musculus GN=Calm1 PE=1 SV=2	MOUSE	9	1.35518897	0.3516773	1.20226395	1.127197981	1.721868992	0.97274721	0.55458552	1.44544005	0.648634374	1.406048059	0.76559663	0.74576902	1.62929595	0.363078088	1.247382998
313	8.73	8.78	32.6200008	17.8499997	17.8499997	sp|P38060|HI	Hydroxymethylglutaryl-CoA lyase, mitochondrial OS=Mus musculus GN=Hmgcl PE=1 SV=2	MOUSE	8	2.24905491	0.2033376	1.43218803	1.570363045	3.311311007	2.48885703	0.1847889	1.43218803	1.737800956	3.698282003	1.39315701	0.31806651	1.41905701	0.981747925	1.940886021
314	8.73	8.73	59.5899999	59.5899999	50	sp|P56389|CI	Cytidine deaminase OS=Mus musculus GN=Cda PE=1 SV=2	MOUSE	9	0.99083197	0.93418932	1.43218803	0.704693079	1.419057012	0.88715601	0.84891719	1.41905701	0.619441092	1.258924961	0.4570882	0.2353434	1.61435902	0.280543387	0.73790431
315	8.7	8.71	38.3300006	18.1799993	15.4799998	sp|Q9CY58|P	Plasminogen activator inhibitor 1 RNA-binding protein OS=Mus musculus GN=Serbp1 PE=1 SV=2	MOUSE	6	1.35518897	0.0810255	1.158777	1.16949904	1.570363045	1.31825697	0.15013041	1.25892496	1.047129035	1.690441012	1.34276497	0.1390429	1.18032098	1.137627006	1.599557996
316	8.65	8.75	17.9700002	3.94000001	3.10399998	sp|Q68FD5|C	Clathrin heavy chain 1 OS=Mus musculus GN=Cltc PE=1 SV=3	MOUSE	5	0.237684	0.03628992	1.75388098	0.066680677	0.416869402	0.12589251	0.2455136	1.69044101	0.042854849	0.212813899	0.57016432	0.2358416	1.36772895	0.366437614	0.779830098
317	8.63	8.68	58.9999974	31.4200014	22.2200006	sp|O08756|H	3-hydroxyacyl-CoA dehydrogenase type-2 OS=Mus musculus GN=Hsd17b10 PE=1 SV=4	MOUSE	10	0.88715601	0.85513651	1.35518897	0.648634374	1.202263951	0.87096357	0.932522	1.41905701	0.591561615	1.235947013	0.77268058	0.42215431	1.27057397	0.591561615	0.981747925
318	8.54	8.62	61.1800015	40.93	36.71	sp|P51859|HI	Hepatoma-derived growth factor OS=Mus musculus GN=Hdgf PE=1 SV=2	MOUSE	7	3.34194994	0.01602002	1.61435902	2.070141077	5.105050087	3.2210691	0.01556901	1.599558	2.013724089	4.920394897	2.70395804	0.03883066	1.64437199	1.644371986	4.285484791
319	8.5	8.5	36.4199996	18.0999994	16.3399994	sp|Q9QYR9|/	Acyl-coenzyme A thioesterase 2, mitochondrial OS=Mus musculus GN=Acot2 PE=1 SV=2	MOUSE	4	0.74473202	0.61294758	2.0892961	0.35318321	1.55596602	0.86297852	0.77236342	2.10862804	0.413047493	1.819700956	1.21338904	0.75076991	2.0892961	0.580764413	2.558585882
320	8.47	9.42	29.7399998	6.87000006	5.54299988	sp|O08788|D	Dynactin subunit 1 OS=Mus musculus GN=Dctn1 PE=1 SV=3	MOUSE	4	1.23594701	0.50749773	3.63077998	0.340408206	4.528975964	1.69044101	0.1940583	2.03235698	0.831763685	3.499452114	1.64437199	0.21131671	3.16227794	0.519995987	5.248075008
321	8.47	8.54	36.1699998	8.71199966	7.38599971	sp|P57016|L/	Ladinin-1 OS=Mus musculus GN=Lad1 PE=1 SV=1	MOUSE	6	2.051162	0.2273223	1.54170001	1.330453992	3.311311007	2.03235698	0.1775247	1.41905701	1.432188034	3.076097012	1.77010906	0.21518751	1.35518897	1.30617094	2.53512907
322	8.47	8.47	68.4199989	48.0300009	35.5300009	sp|Q01768|NI	Nucleoside diphosphate kinase B OS=Mus musculus GN=Nme2 PE=1 SV=1	MOUSE	6	0.2964831	0.4781538	1.99526203	0.172186896	0.591561615	0.67920363	0.36896581	1.21338904	0.524807513	0.824138105	0.59156162	0.29870302	1.35518897	0.359749287	0.801678121
323	8.43	8.43	49.0200013	33.3299995	27.4500012	sp|Q64152|B	Transcription factor BTF3 OS=Mus musculus GN=Btf3 PE=2 SV=3	MOUSE	10	1.19124198	0.69847149	1.14815402	1.037528038	1.380383968	0.85506672	0.90829498	1.43218803	0.564936996	1.224616051	0.98174793	0.88775218	1.35518897	0.717794299	1.330453992
324	8.41	8.53	26.8999994	11.5000002	10.2700003	sp|Q8BH00|A	Aldehyde dehydrogenase family 8 member A1 OS=Mus musculus GN=Aldh8a1 PE=1 SV=1	MOUSE	6	1.25892496	0.97463131	1.80301797	0.698232412	2.376840115	1.61435902	0.70276278	1.81970096	0.88715601	2.992264986	1.65958703	0.37318641	1.80301797	0.920449615	3.133285999
325	8.4	8.47	71.6400027	43.2799995	37.3100013	sp|Q9DB15|R	39S ribosomal protein L12, mitochondrial OS=Mus musculus GN=Mrpl12 PE=1 SV=2	MOUSE	7	1.21338904	0.30454221	1.40604806	0.862978518	1.706081986	1.21338904	0.60636902	1.40604806	0.862978518	1.706081986	0.58076441	0.66220599	1.39315701	0.416869402	0.809095919
326	8.37	32.4	52.8100014	39.3299997	39.3299997	sp|Q9CWF2|	Tubulin beta-2B chain OS=Mus musculus GN=Tubb2b PE=1 SV=1	MOUSE	38	0.52966338	0.4856849	1.61435902	0.237683997	0.855066717	0.77268058	0.6337871	1.78648806	0.432513803	1.380383968	1.20226395	0.70863807	1.39315701	0.862978518	1.721868992
326	0	32.4	52.8100014	39.3299997	39.3299997	sp|Q7TMM9|	Tubulin beta-2A chain OS=Mus musculus GN=Tubb2a PE=1 SV=1	MOUSE	38															
327	8.34	8.63	34.3499988	13.6199996	11.3799997	sp|O88746|T	Target of Myb protein 1 OS=Mus musculus GN=Tom1 PE=1 SV=1	MOUSE	4	1.04712904	0.84966427	1.38038397	0.758577585	1.445440054	1.27057397	0.43643281	2.10862804	0.602559626	2.703958035	1.27057397	0.43999109	1.77010906	0.717794299	2.269865036
328	8.3	8.58	63.6799991	23.8800004	15.4200003	sp|P37804|T/	Transgelin OS=Mus musculus GN=Tagln PE=1 SV=3	MOUSE	6	1.49968505	0.1221924	1.62929595	0.920449615	2.53512907	1.38038397	0.58533257	1.61435902	0.855066717	2.333457947	1.36772895	0.41048521	1.57036305	0.870963573	2.269865036
329	8.29	8.57	19.8500007	14.7699997	10.65	sp|P05201|A/	Aspartate aminotransferase, cytoplasmic OS=Mus musculus GN=Got1 PE=1 SV=3	MOUSE	4	0.77268058	0.27136409	1.39315701	0.55462569	1.076465011	0.81658238	0.47090781	1.47231197	0.55462569	1.202263951	0.91201079	0.70231539	1.30617094	0.698232412	1.19124198
330	8.25	8.38	24.8199999	8.94199982	8.02899972	sp|P42932|T	T-complex protein 1 subunit theta OS=Mus musculus GN=Cct8 PE=1 SV=3	MOUSE	4	0.63095743	0.33689541	1.55596602	0.39445731	0.981747925	0.44055489	0.19574	1.65958703	0.207014099	0.731139123	0.75162292	0.73047048	1.55596602	0.478630096	1.16949904
331	8.22	10.14	65.3299987	33.3299995	33.3299995	sp|Q9EQX4|/	Allograft inflammatory factor 1-like OS=Mus musculus GN=Aif1l PE=2 SV=1	MOUSE	5	2.26986504	0.2111347	1.49968505	1.51356101	3.40408206	2.58225989	0.1725944	1.52756596	1.690441012	3.908409119	1.94088602	0.42445391	1.49968505	1.29419601	2.884032011
332	8.22	8.3	28.0200005	16.2200004	16.2200004	sp|P07356|AI	Annexin A2 OS=Mus musculus GN=Anxa2 PE=1 SV=2	MOUSE	6	0.51050502	0.1015191	1.99526203	0.181970105	1.018591046	1.11686301	0.83994073	1.38038397	0.809095919	1.770109057	2.51188588	0.1465057	1.77010906	1.419057012	4.875285149
333	8.22	8.28	47.9600012	24.1400003	21.9400004	sp|Q99KP3|C	Lambda-crystallin homolog OS=Mus musculus GN=Cryl1 PE=2 SV=3	MOUSE	5	0.51050502	0.2201681	2.14783001	0.177010894	1.096477985	0.1614359	0.03897218	2.93764997	0.01180321	0.474242002	0.34673679	0.09670499	1.99526203	0.063679554	0.691830993
334	8.2	8.23	23.4500006	12.8600001	12.8600001	sp|Q9R0H0|A	Peroxisomal acyl-coenzyme A oxidase 1 OS=Mus musculus GN=Acox1 PE=1 SV=5	MOUSE	6	0.8472274	0.4149448	1.41905701	0.564936996	1.202263951	0.9289664	0.71425909	1.40604806	0.619441092	1.30617094	1.39315701	0.2227845	1.247383	1.116863012	1.836537957
335	8.18	8.22	12.85	3.33800018	3.01799998	sp|P70670|N/	Nascent polypeptide-associated complex subunit alpha, muscle-specific form OS=Mus musculus GN=Naca PE	MOUSE	6	1.12719798	0.56789732	1.35518897	0.831763685	1.527565956	0.90364939	0.85698998	1.40604806	0.636795521	1.270573974	0.98174793	0.74414247	1.36772895	0.724435985	1.342764974
335	0	8.13	43.2599992	30.7000011	30.7000011	sp|Q60817|N/	Nascent polypeptide-associated complex subunit alpha OS=Mus musculus GN=Naca PE=1 SV=1	MOUSE	6															
336	8.14	8.26	25.0699997	11.5199998	6.85100034	sp|P21981|T	Protein-glutamine gamma-glutamyltransferase 2 OS=Mus musculus GN=Tgm2 PE=1 SV=4	MOUSE	6	0.98174793	0.90498692	1.49968505	0.586138189	1.472311974	1.75388098	0.22321171	1.58489299	1.106624007	3.191538095	3.46736908	0.0247886	1.70608199	2.032356977	7.516229153
337	8.13	8.18	13.47	3.96899991	3.96899991	sp|P01027|C	Complement C3 OS=Mus musculus GN=C3 PE=1 SV=3	MOUSE	8	1.11686301	0.55273902	1.23594701	0.90364939	1.380383968	1.21338904	0.41159871	1.27057397	0.954992592	1.570363045	2.26986504	0.100314	1.51356101	1.499685049	3.564511061
338	8.1	8.52	9.19099972	1.987	1.822	sp|Q9JLB4|C	Cubilin OS=Mus musculus GN=Cubn PE=1 SV=3	MOUSE	5	0.93756199	0.84663582	1.30617094	0.717794299	1.224616051	1.05681801	0.68455797	1.247383	0.847227395	1.318256974	0.49659231	0.2358084	1.58489299	0.313328594	0.787045777
339	8.1	8.18	22.0300004	8.37000012	6.90200031	sp|Q9QXD1|/	Peroxisomal acyl-coenzyme A oxidase 2 OS=Mus musculus GN=Acox2 PE=1 SV=2	MOUSE	4	0.59156162	0.06105523	1.47231197	0.334194988	0.870963573	0.28840321	0.14543471	1.97696996	0.1419058	0.570164323	0.79432821	0.3580668	1.19124198	0.608134985	0.946237087
340	8.09	8.26	23.4200001	10.8199999	8.4420003	sp|Q61316|H	Heat shock 70 kDa protein 4 OS=Mus musculus GN=Hspa4 PE=1 SV=1	MOUSE	7	1.55596602	0.46236429	1.57036305	0.990831971	2.443430901	1.45881403	0.3973892	1.57036305	0.928966403	2.290868044	1.21338904	0.4251456	1.58489299	0.765596628	1.905460954
341	8.06	8.2	25.5400002	5.61299995	5.61299995	sp|Q8K370|A	Acyl-CoA dehydrogenase family member 10 OS=Mus musculus GN=Acad10 PE=2 SV=1	MOUSE	4	0.36307809	0.06096453	1.97696996	0.091201082	0.717794299	0.62517268	0.49152601	1.97696996	0.296483099	1.235947013	0.3981072	0.06104052	2.051162	0.106659599	0.816582382
342	8.06	8.06	32.4600011	32.4600011	32.4600011	sp|Q9CQH0|I	PDZK1-interacting protein 1 OS=Mus musculus GN=Pdzk1ip1 PE=2 SV=1	MOUSE	7	0.76559663	0.60160309	1.20226395	0.586138189	0.920449615	0.474242	0.29043999	1.47231197	0.185353205	0.698232412	0.50118721	0.26220501	1.43218803	0.207014099	0.717794299
343	8.05	8.05	45.42	17.5600007	17.5600007	sp|Q9R0U0|S	Serine/arginine-rich splicing factor 10 OS=Mus musculus GN=Srsf10 PE=1 SV=2	MOUSE	5	1.27057397	0.72303373	1.43218803	0.88715601	1.836537957	1.158777	0.66047847	1.44544005	0.801678121	1.690441012	1.00925303	0.86273313	1.18032098	0.855066717	1.202263951
344	8.05	8.05	44.1500008	26.76	26.76	sp|Q8VCI5|PI	Peroxisomal biogenesis factor 19 OS=Mus musculus GN=Pex19 PE=1 SV=1	MOUSE	7	1	0.57918692	1.38038397	0.724435985	1.380383968	0.99083197	0.99196398	1.48593605	0.672976673	1.472311974	0.86297852	0.48568669	1.43218803	0.602559626	1.235947013
345	8.04	8.18	43.3499992	27.0000011	19.7699994	sp|Q9DCT2|N	NADH dehydrogenase [ubiquinone] iron-sulfur protein 3, mitochondrial OS=Mus musculus GN=Ndufs3 PE=1 S	MOUSE	6	0.41304749	0.1558193	1.57036305	0.224905506	0.648634374	0.50582469	0.1725215	1.49968505	0.288403213	0.758577585	0.33728731	0.1041085	1.69044101	0.162929595	0.570164323
346	8.04	8.06	56.6299975	46.9900012	46.9900012	sp|P62077|TI	Mitochondrial import inner membrane translocase subunit Tim8 B OS=Mus musculus GN=Timm8b PE=2 SV=1	MOUSE	7	0.4875285	0.85119712	2.12813902	0.183653802	1.037528038	1.03752804	0.91498393	1.21338904	0.855066717	1.258924961	0.51522863	0.53924328	1.51356101	0.319153786	0.779830098
347	8.04	8.04	48.5399991	19.6600005	17.4799994	sp|Q07417|A	Short-chain specific acyl-CoA dehydrogenase, mitochondrial OS=Mus musculus GN=Acads PE=2 SV=2	MOUSE	5	0.4017908	0.09752784	1.65958703	0.235504895	0.666806817	0.57016432	0.19676121	1.52756596	0.366437614	0.870963573	0.64863437	0.3426697	1.44544005	0.428548515	0.937561989
348	8.01	8.01	46.3499993	30.8999986	19.7400004	sp|Q9D6Y7|M	Mitochondrial peptide methionine sulfoxide reductase OS=Mus musculus GN=Msra PE=1 SV=1	MOUSE	5	0.72443599	0.152574	1.38038397	0.487528503	1	0.57543987	0.1445868	1.62929595	0.349945188	0.937561989	0.68548822	0.06801037	1.23594701	0.534564376	0.847227395
349	8	8.03	24.5000005	10.2300003	10.2300003	sp|Q60936|AI	Chaperone activity of bc1 complex-like, mitochondrial OS=Mus musculus GN=Adck3 PE=2 SV=2	MOUSE	5	1.21338904	0.80277008	1.16949904	1.037528038	1.419057012	1.08642602	0.83670777	1.43218803	0.758577585	1.55596602	0.474242	0.38414389	1.75388098	0.2558586	0.831763685
350	8	8.03	29.1500002	12.8299996	11.0799998	sp|Q80XN0|B	D-beta-hydroxybutyrate dehydrogenase, mitochondrial OS=Mus musculus GN=Bdh1 PE=1 SV=2	MOUSE	4	0.41304749	0.04682804	1.72186899	0.218776196	0.711213529	0.474242	0.08607271	1.69044101	0.246603906	0.801678121	0.35974929	0.1165767	1.55596602	0.1127197	0.55975759
351	8	8	31.8800002	11.8299998	11.8299998	sp|P18572|B/	Basigin OS=Mus musculus GN=Bsg PE=1 SV=2	MOUSE	4	1.51356101	0.2335576	1.33045399	1.137627006	2.128139019	0.53951061	0.68853182	1.85353196	0.310455889	1	1.04712904	0.99340987	1.34276497	0.779830098	1.367728949
352	7.97	36.97	78.1899989	71.5399981	47.6099998	sp|P63268|A	Actin, gamma-enteric smooth muscle OS=Mus musculus GN=Actg2 PE=2 SV=1	MOUSE	54	0.37325019	0.37307471	4.52897596	0.074473187	1.690441012	0.28575909	0.31367251	4.69894123	0.044463132	1.342764974	0.68548822	0.83606583	4.4463129	0.154170096	3.047894955
352	0	37.13	78.2500029	71.6199994	47.4799991	sp|P68033|A	Actin, alpha cardiac muscle 1 OS=Mus musculus GN=Actc1 PE=1 SV=1	MOUSE	57															
352	0	36.96	78.2500029	71.6199994	47.4799991	sp|P62737|A	Actin, aortic smooth muscle OS=Mus musculus GN=Acta2 PE=1 SV=1	MOUSE	54															
353	7.97	7.98	36.559999	29.0699989	22.0300004	sp|Q9CRB9|	Coiled-coil-helix-coiled-coil-helix domain-containing protein 3, mitochondrial OS=Mus musculus GN=Chchd3 PI	MOUSE	6	0.94623709	0.4577907	1.12719798	0.839460015	1.066596031	0.85506672	0.00368992	1.16949904	0.73790431	1	0.87902248	0.33956981	1.158777	0.758577585	1.018591046
354	7.94	8.14	28.0900002	9.5569998	7.22599998	sp|Q61699|H	Heat shock protein 105 kDa OS=Mus musculus GN=Hsph1 PE=1 SV=2	MOUSE	4	0.89536482	0.85365832	1.48593605	0.619441092	1.330453992	0.95499259	0.60033059	1.57036305	0.613762021	1.499685049	0.92044962	0.88879597	1.55596602	0.591561615	1.432188034
355	7.94	7.96	49.0399987	39.4199997	39.4199997	sp|P61458|PI	Pterin-4-alpha-carbinolamine dehydratase OS=Mus musculus GN=Pcbd1 PE=1 SV=2	MOUSE	4	1.20226395	0.40402871	1.25892496	0.954992592	1.541700006	0.78704578	0.20060921	1.35518897	0.580764413	1.066596031	0.52966338	0.33321211	1.43218803	0.288403213	0.758577585
356	7.86	7.86	40.23	19.1699997	19.1699997	sp|Q9D172|E	ES1 protein homolog, mitochondrial OS=Mus musculus GN=D10Jhu81e PE=1 SV=1	MOUSE	5	2.07014108	0.64417148	1.90546095	1.08642602	3.80189395	2.10862804	0.64267641	1.90546095	1.106624007	3.872575998	1.62929595	0.8694998	1.870682	0.870963573	2.992264986
357	7.84	7.84	32.3700011	19.3200007	19.3200007	sp|Q60590|A	Alpha-1-acid glycoprotein 1 OS=Mus musculus GN=Orm1 PE=1 SV=1	MOUSE	4	5.86138201	0.28723729	1.90546095	3.076097012	22.08004951	8.31763744	0.1776952	1.97696996	4.207265854	30.19952011	20.3235703	0.1020702	2.07014108	9.817479134	46.9894104
358	7.82	7.85	44.4999993	16.7400003	12.1600002	sp|Q62418|DI	Drebrin-like protein OS=Mus musculus GN=Dbnl PE=1 SV=2	MOUSE	4	0.87096357	0.67205632	1.29419601	0.672976673	1.127197981	0.94623709	0.52672273	1.247383	0.758577585	1.180320978	0.63095743	0.26324651	1.44544005	0.387257606	0.912010789
359	7.82	7.83	67.96	41.7499989	41.7499989	sp|P62806|H	Histone H4 OS=Mus musculus GN=Hist1h4a PE=1 SV=2	MOUSE	6	0.62517268	0.1224258	1.36772895	0.413047493	0.855066717	0.45289761	0.03405223	1.47231197	0.280543387	0.666806817	0.83176369	0.42090529	1.21338904	0.666806817	1.009253025
360	7.81	7.82	24.5199993	14.0300006	14.0300006	sp|O35488|S	Very long-chain acyl-CoA synthetase OS=Mus musculus GN=Slc27a2 PE=1 SV=2	MOUSE	5	0.61376202	0.00383561	1.45881403	0.376703799	0.895364821	0.89536482	0.35571221	1.43218803	0.613762021	1.28233099	0.81658238	0.07198287	1.67494297	0.469894111	1.367728949
361	7.74	10.2	32.8299999	18.9799994	15.0600001	sp|P06151|LI	L-lactate dehydrogenase A chain OS=Mus musculus GN=Ldha PE=1 SV=3	MOUSE	6	0.67297667	0.20549209	1.58489299	0.420726597	1.066596031	0.75857759	0.26979399	1.44544005	0.519995987	1.096477985	0.89536482	0.71736097	1.58489299	0.55975759	1.419057012
362	7.74	7.75	23.1999993	7.51599968	7.51599968	sp|O88487|D	Cytoplasmic dynein 1 intermediate chain 2 OS=Mus musculus GN=Dync1i2 PE=2 SV=1	MOUSE	4	1.10662401	0.95800781	1.64437199	0.672976673	1.819700956	1.41905701	0.58169311	1.44544005	0.981747925	2.108628035	1.77010906	0.26491341	1.43218803	1.235947013	2.679167986
363	7.74	7.74	47.3100007	47.3100007	43.0099994	sp|Q91VW3|S	SH3 domain-binding glutamic acid-rich-like protein 3 OS=Mus musculus GN=Sh3bgrl3 PE=1 SV=1	MOUSE	4	2.42102909	0.53579593	1.75388098	1.380383968	5.152287006	2.12813902	0.72955358	1.70608199	1.247382998	4.092607021	3.37287307	0.1445448	1.78648806	1.887990952	7.870458126
364	7.73	7.73	35.2100015	35.2100015	30.28	sp|Q9CQI6|C	Coactosin-like protein OS=Mus musculus GN=Cotl1 PE=1 SV=3	MOUSE	5	1.13762701	0.52551198	1.40604806	0.809095919	1.614359021	1.04712904	0.717942	1.29419601	0.809095919	1.419057012	0.88715601	0.47953159	1.40604806	0.630957425	1.247382998
365	7.72	7.95	47.9999989	28.3100009	26.7699987	sp|Q9D0M3|	Cytochrome c1, heme protein, mitochondrial OS=Mus musculus GN=Cyc1 PE=1 SV=1	MOUSE	5	0.58076441	0.3275919	1.21338904	0.405508488	0.704693079	0.36307809	0.21272931	1.55596602	0.136772901	0.564936996	0.52966338	0.3477982	1.31825697	0.307609707	0.698232412
366	7.68	7.79	87.9499972	73.4899998	55.4199994	sp|Q9CQR2|I	40S ribosomal protein S21 OS=Mus musculus GN=Rps21 PE=2 SV=1	MOUSE	8	2.6546061	0.05156441	1.67494297	1.584892988	4.168694019	3.872576	0.0723936	1.83653796	2.108628035	6.79203701	4.0179081	0.1311858	1.83653796	2.187762022	6.854881763
367	7.67	7.7	30.8699995	16.0699993	16.0699993	sp|O54724|P	Polymerase I and transcript release factor OS=Mus musculus GN=Ptrf PE=1 SV=1	MOUSE	7	1.04712904	0.47288799	1.28233099	0.816582382	1.342764974	0.89536482	0.61425972	1.67494297	0.534564376	1.499685049	0.99083197	0.76600868	1.45881403	0.679203629	1.445440054
368	7.65	7.65	37.5800014	27.8499991	22.8200004	sp|Q9CPV4|G	Glyoxalase domain-containing protein 4 OS=Mus musculus GN=Glod4 PE=2 SV=1	MOUSE	6	1.34276497	0.09654079	1.47231197	0.912010789	1.995262027	1.33045399	0.09386242	1.38038397	0.963828981	1.836537957	1.20226395	0.03560348	1.25892496	0.954992592	1.51356101
369	7.62	7.81	59.1400027	23.6599997	23.6599997	sp|Q8VBW8|	Tetratricopeptide repeat protein 36 OS=Mus musculus GN=Ttc36 PE=2 SV=1	MOUSE	5	1.19124198	0.53234309	1.31825697	0.90364939	1.599557996	0.91201079	0.96935469	1.30617094	0.685488224	1.19124198	0.25822601	0.01077879	1.64437199	0.134276494	0.424619585
370	7.6	11.7	44.1300005	35.2200002	29.9600005	sp|P61982|14	14-3-3 protein gamma OS=Mus musculus GN=Ywhag PE=1 SV=2	MOUSE	8	0.90364939	0.96403879	1.69044101	0.505824685	1.527565956	1.36772895	0.51300103	1.78648806	0.765596628	2.443430901	1.65958703	0.2096635	1.48593605	1.116863012	2.558585882
371	7.55	7.57	22.2399995	9.33799967	9.33799967	sp|Q76MZ3|2	Serine/threonine-protein phosphatase 2A 65 kDa regulatory subunit A alpha isoform OS=Mus musculus GN=P	MOUSE	6	0.91201079	0.67187119	1.51356101	0.591561615	1.380383968	0.82413811	0.2484123	1.27057397	0.648634374	1.047129035	0.85506672	0.71535319	1.77010906	0.474242002	1.51356101
372	7.53	7.7	18.6000004	2.57600006	2.57600006	sp|Q6PDN3|M	Myosin light chain kinase, smooth muscle OS=Mus musculus GN=Mylk PE=1 SV=3	MOUSE	5	0.564937	0.20985951	2.91071701	0.149968505	1.644371986	0.55462569	0.25397629	4.48745394	0.077983007	2.488857031	0.75857759	0.43358171	4.4463129	0.170608193	3.372873068
373	7.52	7.78	25.6900013	7.64300004	5.43299988	sp|Q61941|NI	NAD(P) transhydrogenase, mitochondrial OS=Mus musculus GN=Nnt PE=1 SV=2	MOUSE	7	0.83946002	0.6111449	1.51356101	0.549540877	1.270573974	0.40926069	0.3192316	1.78648806	0.233345807	0.731139123	0.95499259	0.97409463	1.34276497	0.704693079	1.28233099
374	7.52	7.61	22.4199995	5.38499989	5.38499989	sp|Q78PY7|S	Staphylococcal nuclease domain-containing protein 1 OS=Mus musculus GN=Snd1 PE=1 SV=1	MOUSE	4	0.31332859	0.25174579	29.1071701	0.027797131	9.120108604	0.43651581	0.3219544	3.8370719	0.111686297	1.67494297	1.158777	0.71307921	15.9955797	0.072443597	16.59586906

375	7.47	7.47	19.8799998	15.2700007	14.7	sp|Q61646|H	Haptoglobin OS=Mus musculus GN=Hp PE=1 SV=1	MOUSE	9	8.31763744	0.4674665	4.48745394	1.853531957	15.99557972	19.9526196	0.02501639	4.7424202	4.207265854	37.32501984	31.332861	0.00774907	3.34194994	9.375619888	53.45643997
376	7.46	7.55	21.2899998	8.51600021	7.61300027	sp|Q99LI8|H	Hepatocyte growth factor-regulated tyrosine kinase substrate OS=Mus musculus GN=Hgs PE=1 SV=2	MOUSE	5	0.95499259	0.99632841	5.97035313	0.161435902	5.70164299	1.45881403	0.52727139	2.91071701	0.501187205	4.207265854	0.86297852	0.80668509	2.80543399	0.307609707	2.421029091
377	7.41	7.41	35.3700012	23.4799996	14.6300003	sp|Q91Z53|G	Glyoxylate reductase/hydroxypyruvate reductase OS=Mus musculus GN=Grhpr PE=1 SV=1	MOUSE	5	0.61376202	0.07960457	1.41905701	0.413047493	0.870963573	0.69183099	0.05095807	1.29419601	0.524807513	0.895364821	0.61376202	0.13560501	1.51356101	0.398107201	0.928966403
378	7.4	7.43	43.1600004	20.0000003	16.4900005	sp|Q9CWS0|I	N(G),N(G)-dimethylarginine dimethylaminohydrolase 1 OS=Mus musculus GN=Ddah1 PE=1 SV=3	MOUSE	6	0.63095743	0.72352618	1.70608199	0.251188606	1.076465011	0.67297667	0.54094589	1.28233099	0.424619585	0.862978518	0.4570882	0.29700661	1.49968505	0.08550667	0.685488224
379	7.32	7.92	43.810001	28.5699993	23.1700003	sp|O35887|C	Calumenin OS=Mus musculus GN=Calu PE=1 SV=1	MOUSE	7	0.89536482	0.4645249	1.22461605	0.731139123	1.096477985	0.51050502	0.00368708	1.62929595	0.301995188	0.831763685	0.53951061	0.03127765	1.52756596	0.334194988	0.824138105
380	7.28	12.14	35.7899994	17.5099999	10.4500003	sp|Q91WJ8|F	Far upstream element-binding protein 1 OS=Mus musculus GN=Fubp1 PE=1 SV=1	MOUSE	7	0.98174793	0.90962732	1.12719798	0.870963573	1.106624007	1.05681801	0.47212011	1.23594701	0.855066717	1.30617094	1.02801597	0.64376319	1.12719798	0.912010789	1.158776999
381	7.26	7.26	16.9400007	6.02600016	6.02600016	sp|Q61656|D	Probable ATP-dependent RNA helicase DDX5 OS=Mus musculus GN=Ddx5 PE=1 SV=2	MOUSE	4	0.4920395	0.1649345	1.49968505	0.319153786	0.73790431	0.564937	0.09469648	1.44544005	0.369828194	0.816582382	0.96382898	0.90351111	1.29419601	0.73790431	1.247382998
382	7.24	7.28	27.0599991	11.6499998	6.98899999	sp|P06745|G	Glucose-6-phosphate isomerase OS=Mus musculus GN=Gpi PE=1 SV=4	MOUSE	5	0.52480751	0.34329969	1.43218803	0.366437614	0.751622915	0.63679552	0.1609333	1.39315701	0.416869402	0.88715601	0.83176369	0.41801041	1.12719798	0.73790431	0.937561989
383	7.24	7.27	31.6199988	19.3700001	17.6599994	sp|Q8VDQ1|P	Prostaglandin reductase 2 OS=Mus musculus GN=Ptgr2 PE=1 SV=2	MOUSE	7	0.87902248	0.71374261	1.43218803	0.597035289	1.258924961	0.88715601	0.98240233	1.51356101	0.55462569	1.342764974	0.38370732	0.06811223	1.64437199	0.113762699	0.630957425
384	7.21	7.59	36.8999988	19.7600007	10.7100003	sp|P81117|N	Nucleobindin-2 OS=Mus musculus GN=Nucb2 PE=1 SV=2	MOUSE	4	0.96382898	0.76748723	1.31825697	0.731139123	1.270573974	0.9289664	0.48227349	1.40604806	0.654636085	1.30617094	1.08642602	0.85441119	1.39315701	0.779830098	1.51356101
385	7.19	7.19	28.0099988	11.4799999	11.4799999	sp|Q8VEM8|M	Phosphate carrier protein, mitochondrial OS=Mus musculus GN=Slc25a3 PE=1 SV=1	MOUSE	5	0.4168694	0.1530097	1.22461605	0.313328594	0.510505021	0.1513561	0.06321842	1.64437199	0.09289664	0.248885706	0.31332859	0.1030347	1.43218803	0.190546095	0.4487454
386	7.18	11.64	34.9700004	26.0600001	26.0600001	sp|O35737|H	Heterogeneous nuclear ribonucleoprotein H OS=Mus musculus GN=Hnrnph1 PE=1 SV=3	MOUSE	11															
387	7.16	7.28	30.3200007	7.78800026	6.95699975	sp|Q61768|K	Kinesin-1 heavy chain OS=Mus musculus GN=Kif5b PE=1 SV=3	MOUSE	5	0.95499259	0.6424256	1.31825697	0.724435985	1.258924961	1.01859105	0.78534383	1.247383	0.816582382	1.270573974	0.9289664	0.68365502	1.23594701	0.751622915	1.14815402
388	7.09	7.09	81.4000011	75.5800009	39.5300001	sp|P56391|C	Cytochrome c oxidase subunit 6B1 OS=Mus musculus GN=Cox6b1 PE=1 SV=2	MOUSE	4	1.01859105	0.1011364	1.34276497	0.758577585	1.355188966	0.80167812	0.2367139	1.34276497	0.597035289	1.076465011	0.80167812	0.08541717	1.34276497	0.602559626	1.076465011
389	7.08	7.09	25.3500015	6.33699969	6.33699969	sp|P23780|B	Beta-galactosidase OS=Mus musculus GN=Glb1 PE=2 SV=1	MOUSE	5	1.69044101	0.59342933	1.55596602	1.08642602	2.83139205	1.78648806	0.3872382	1.55596602	1.14815402	3.019952059	1.04712904	0.95249832	1.51356101	0.691830993	1.67494297
390	7.01	7.04	22.5899994	11.8500002	6.97399974	sp|Q99MR8|M	Methylcrotonoyl-CoA carboxylase subunit alpha, mitochondrial OS=Mus musculus GN=Mccc1 PE=2 SV=2	MOUSE	4	0.87902248	0.78943312	1.29419601	0.679203629	1.137627006	0.87902248	0.71796417	1.31825697	0.654636085	1.158776999	0.7798301	0.3446736	1.29419601	0.591561615	1.009253025
391	6.97	7.13	37.8300011	14.4500002	10.65	sp|P10852|4	4F2 cell-surface antigen heavy chain OS=Mus musculus GN=Slc3a2 PE=1 SV=1	MOUSE	6	0.73790431	0.3938745	1.51356101	0.465586096	1.116863012	0.65463609	0.1860707	1.39315701	0.436515808	0.912010789	0.64863437	0.1400165	1.30617094	0.474242002	0.847227395
392	6.97	7.06	51.5900016	36.5099996	36.5099996	sp|P56812|P	Programmed cell death protein 5 OS=Mus musculus GN=Pdcd5 PE=1 SV=3	MOUSE	5	1.33045399	0.47449759	1.34276497	0.990831971	1.853531957	0.91201079	0.6776647	1.45881403	0.625172675	1.330453992	1.16949904	0.54088491	1.39315701	0.839460015	1.629295945
393	6.96	31.72	79.2299986	66.9200003	55.3799987	sp|Q64522|H	Histone H2A type 2-B OS=Mus musculus GN=Hist2h2ab PE=1 SV=3	MOUSE	27	1.05681801	0.50741857	1.70608199	0.619441092	1.803017974	0.80167812	0.58133918	1.28233099	0.625172675	1.028015971	0.93756199	0.94761151	1.20226395	0.772680581	1.127197981
394	6.88	6.91	14.1900003	7.50399977	7.50399977	sp|Q68FL4|S	Putative adenosylhomocysteinase 3 OS=Mus musculus GN=Ahcyl2 PE=1 SV=1	MOUSE	4	1.20226395	0.43999919	1.43218803	0.839460015	1.721868992	1.08642602	0.50991827	2.70395804	0.401790798	2.964831114	0.73113912	0.1417937	2.72897792	0.251188606	1.995262027
395	6.84	6.86	18.2699993	13.7099996	13.7099996	sp|P50580|P	Proliferation-associated protein 2G4 OS=Mus musculus GN=Pa2g4 PE=1 SV=3	MOUSE	4	0.4920395	0.35502231	1.99526203	0.242102906	0.981747925	0.86297852	0.78104711	1.69044101	0.510505021	1.458814025	1.18032098	0.52982599	1.30617094	0.90364939	1.541700006
396	6.84	6.84	31.1500013	25.7699996	22.6899996	sp|P00920|C	Carbonic anhydrase 2 OS=Mus musculus GN=Ca2 PE=1 SV=4	MOUSE	4	0.66680682	0.31104791	1.23594701	0.529663384	0.824138105	0.72443599	0.387025	1.19124198	0.602559626	0.862978518	0.7177943	0.42532969	1.25892496	0.55975759	0.90364939
397	6.78	6.82	29.9100012	26.0699987	26.0699987	sp|Q80XU3|N	Nuclear ubiquitous casein and cyclin-dependent kinases substrate OS=Mus musculus GN=Nucks1 PE=1 SV=1	MOUSE	5	1.34276497	0.55603099	1.48593605	0.90364939	2.013724089	1.30617094	0.56501168	1.75388098	0.744732022	2.312064886	1.07646501	0.80504519	1.45881403	0.73790431	1.570363045
398	6.76	6.78	40.7099992	20.5500007	14.6200001	sp|Q9QYB1|C	Chloride intracellular channel protein 4 OS=Mus musculus GN=Clic4 PE=1 SV=3	MOUSE	4	0.55975759	0.41427699	1.599558	0.280543387	0.895364821	0.23334581	0.28940511	2.70395804	0.06251727	0.630957425	0.4613176	0.35707849	1.72186899	0.216770396	0.794328213
399	6.74	6.76	22.8200004	8.29899982	8.29899982	sp|P53395|O	Lipoamide acyltransferase component of branched-chain alpha-keto acid dehydrogenase complex, mitochondri	MOUSE	4	0.81658238	0.0060312	1.31825697	0.619441092	1.076465011	0.97274721	0.17896169	1.61435902	0.602559626	1.570363045	1.08642602	0.1679422	1.41905701	0.765596628	1.584892988
400	6.72	6.72	25.8100003	16.4900005	16.1300004	sp|Q99LB2|D	Dehydrogenase/reductase SDR family member 4 OS=Mus musculus GN=Dhrs4 PE=2 SV=2	MOUSE	4	0.65463609	0.27593049	1.64437199	0.390840888	1.076465011	0.4207266	0.25342491	1.92309201	0.216770396	0.809095919	1.10662401	0.66643262	1.14815402	0.963828981	1.28233099
401	6.7	6.75	26.4200002	14.5099998	10.8800001	sp|P97315|C	Cysteine and glycine-rich protein 1 OS=Mus musculus GN=Csrp1 PE=1 SV=3	MOUSE	5	1.04712904	0.72072321	1.22461605	0.855066717	1.28233099	0.80909592	0.2462582	1.33045399	0.608134985	1.076465011	1.04712904	0.82062608	1.158777	0.90364939	1.213389039
402	6.68	6.75	93.0999994	68.9700007	57.4699998	sp|P31786|A	Acyl-CoA-binding protein OS=Mus musculus GN=Dbi PE=1 SV=2	MOUSE	8	2.33345795	0.00321304	1.30617094	1.786488056	2.937649965	2.60615301	0.00201581	1.30617094	1.995262027	3.311311007	2.051162	0.01114831	1.33045399	1.541700006	2.582259893
403	6.67	6.79	26.730001	19.4999993	8.17599967	sp|Q91XE4|A	Aspartoacylase-2 OS=Mus musculus GN=Acy3 PE=1 SV=1	MOUSE	4	0.33419499	0.08408276	1.41905701	0.195884496	0.474242002	0.34994519	0.06800956	1.41905701	0.220800504	0.496592313	0.19952621	0.03522556	1.55596602	0.119124196	0.310455889
404	6.64	6.69	86.7299974	43.3600009	38.0499989	sp|Q9Z1P6|N	NADH dehydrogenase [ubiquinone] 1 alpha subcomplex subunit 7 OS=Mus musculus GN=Ndufa7 PE=1 SV=3	MOUSE	5	0.97274721	0.71940303	1.31825697	0.73790431	1.28233099	0.85506672	0.48502269	1.23594701	0.691830993	1.056818008	0.8472274	0.4051511	1.247383	0.672976673	1.056818008
405	6.62	6.67	21.2200001	6.66700006	6.66700006	sp|P51660|D	Peroxisomal multifunctional enzyme type 2 OS=Mus musculus GN=Hsd17b4 PE=1 SV=3	MOUSE	4	0.4655861	0.41928551	1.70608199	0.277971298	0.794328213	0.65463609	0.09494067	1.29419601	0.487528503	0.847227395	1.00925303	0.56352109	1.61435902	0.625172675	1.629295945
406	6.62	6.63	29.6299994	16.8400005	11.4500001	sp|Q99J99|T	3-mercaptopyruvate sulfurtransferase OS=Mus musculus GN=Mpst PE=1 SV=3	MOUSE	4	0.83946002	0.26007399	1.52756596	0.544502676	1.28233099	0.93756199	0.74529618	1.247383	0.751622915	1.16949904	0.96382898	0.6781379	1.247383	0.772680581	1.202263951
407	6.59	6.66	22.0699996	10.9399997	7.67799988	sp|O08795|G	Glucosidase 2 subunit beta OS=Mus musculus GN=Prkcsh PE=1 SV=1	MOUSE	5	0.99083197	0.65141612	1.57036305	0.625172675	1.55596602	0.83946002	0.98946619	1.47231197	0.524807513	1.235947013	1.34276497	0.72018403	1.35518897	0.990831971	1.940886021
408	6.57	6.57	47.7400005	18.5200006	14.8100004	sp|P62908|R	40S ribosomal protein S3 OS=Mus musculus GN=Rps3 PE=1 SV=1	MOUSE	6	1.16949904	0.81613439	1.47231197	0.794328213	1.721868992	1.18032098	0.72496748	1.40604806	0.839460015	1.659587026	1.27057397	0.28540501	1.36772895	0.928966403	1.737800956
409	6.56	6.62	35.4299992	29.1299999	24.4100004	sp|Q9DBJ1|P	Phosphoglycerate mutase 1 OS=Mus musculus GN=Pgam1 PE=1 SV=3	MOUSE	7	0.4920395	0.2342329	1.81970096	0.27289781	0.895364821	0.7798301	0.2382991	1.47231197	0.519995987	1.14815402	1.06659603	0.63156033	1.58489299	0.672976673	1.706081986
410	6.56	6.6	20.7599998	9.20599997	9.20599997	sp|P34914|H	Epoxide hydrolase 2 OS=Mus musculus GN=Ephx2 PE=1 SV=2	MOUSE	6	0.74473202	0.2045847	1.31825697	0.487528503	0.981747925	0.69823241	0.14602891	1.18032098	0.501187205	0.824138105	0.42854852	0.30309591	1.80301797	0.237683997	0.772680581
411	6.51	6.74	44.0899998	23.6399993	17.7300006	sp|Q9CYZ2|T	Tumor protein D54 OS=Mus musculus GN=Tpd52l2 PE=1 SV=1	MOUSE	6	1.67494297	0.1015921	1.35518897	1.235947013	2.466038942	1.51356101	0.1716848	1.34276497	1.127197981	2.167704105	1.11686301	0.66662139	1.48593605	0.751622915	1.527565956
412	6.51	6.6	46.7500001	13.2499993	10.12	sp|Q91WK0|L	Leucine-rich repeat flightless-interacting protein 2 OS=Mus musculus GN=Lrrfip2 PE=1 SV=1	MOUSE	3	1.44544005	0.19659171	1.18032098	1.224616051	1.786488056	1.47231197	0.1127641	1.29419601	1.137627006	1.976969957	1.69044101	0.0224435	1.44544005	1.16949904	2.53512907
413	6.49	6.52	34.6599996	21.1199999	18.7299997	sp|O09164|S	Extracellular superoxide dismutase [Cu-Zn] OS=Mus musculus GN=Sod3 PE=1 SV=1	MOUSE	6	1.04712904	0.95677161	1.41905701	0.73790431	1.485936046	1.10662401	0.68713468	1.599558	0.691830993	1.803017974	0.42461959	0.65809971	2.4434309	0.172186896	1.037528038
414	6.48	6.77	43.720001	28.9600015	22.4000007	sp|P23198|C	Chromobox protein homolog 3 OS=Mus musculus GN=Cbx3 PE=1 SV=2	MOUSE	5	1.13762701	0.34175029	1.23594701	0.920449615	1.406048059	1.06659603	0.50518131	1.23594701	0.862978518	1.318256974	1.05681801	0.58056343	1.22461605	0.862978518	1.29419601
415	6.47	6.47	59.2800021	31.6700012	19.9100003	sp|Q62093|S	Serine/arginine-rich splicing factor 2 OS=Mus musculus GN=Srsf2 PE=1 SV=4	MOUSE	11	1.52756596	0.1189687	1.27057397	1.202263951	2.070141077	1.36772895	0.14052761	1.23594701	1.106624007	1.753880978	1.02801597	0.98729372	1.28233099	0.801678121	1.330453992
416	6.46	6.65	22.6300001	6.08300008	5.44300005	sp|D3YXK2|S	Scaffold attachment factor B1 OS=Mus musculus GN=Safb PE=2 SV=2	MOUSE	3	1.20226395	0.57939768	2.60615301	0.461317599	3.133285999	0.99083197	0.85528231	1.69044101	0.586138189	1.67494297	0.96382898	0.97187012	1.44544005	0.666806817	1.393157005
417	6.44	6.46	22.5999996	12.4300003	7.15600029	sp|P80317|T	T-complex protein 1 subunit zeta OS=Mus musculus GN=Cct6a PE=1 SV=3	MOUSE	3	1.20226395	0.88217551	1.72186899	0.698232412	2.167704105	1.03752804	0.82674217	1.599558	0.648634374	1.690441012	1.97696996	0.1747596	1.62929595	1.213389039	3.80189395
418	6.42	6.52	34.8500013	11.5199998	9.09100026	sp|Q91VM9|I	Inorganic pyrophosphatase 2, mitochondrial OS=Mus musculus GN=Ppa2 PE=2 SV=1	MOUSE	5	0.51999599	0.29636881	2.72897792	0.190546095	1.419057012	0.90364939	0.70032001	1.90546095	0.474242002	1.721868992	0.8472274	0.50941372	1.23594701	0.679203629	1.047129035
419	6.42	6.42	59.5899999	56.160003	40.4100001	sp|Q9CQX2|C	Cytochrome b5 type B OS=Mus musculus GN=Cyb5b PE=1 SV=1	MOUSE	7	0.96382898	0.81370902	1.35518897	0.717794299	1.30617094	0.78704578	0.66023523	1.35518897	0.57543987	1.066596031	0.67920363	0.70203412	1.38038397	0.457088202	0.937561989
420	6.38	6.63	14.0499994	3.49900015	1.87999997	sp|Q6PDQ2|C	Chromodomain-helicase-DNA-binding protein 4 OS=Mus musculus GN=Chd4 PE=1 SV=1	MOUSE	3	1.43218803	0.04019295	1.51356101	0.946237087	2.167704105	1.43218803	0.0489407	1.72186899	0.831763685	2.466038942	1.20226395	0.00476745	1.30617094	0.920449615	1.55596602
421	6.36	6.36	74.4400024	65.5600011	26.67	sp|P62073|T	Mitochondrial import inner membrane translocase subunit Tim10 OS=Mus musculus GN=Timm10 PE=1 SV=1	MOUSE	6	1.158777	0.26746649	1.29419601	0.895364821	1.499685049	1.14815402	0.27765101	1.25892496	0.912010789	1.445440054	1.05681801	0.56019223	1.247383	0.847227395	1.318256974
422	6.35	6.61	17.7499995	7.16700032	6.03	sp|Q9Z2H5|E	Band 4.1-like protein 1 OS=Mus musculus GN=Epb41l1 PE=1 SV=2	MOUSE	4	1.599558	0.18527129	1.12719798	1.419057012	1.836537957	1.35518897	0.2866298	1.30617094	1.037528038	1.770109057	1.13762701	0.434077	1.43218803	0.794328213	1.629295945
423	6.35	6.43	21.2799996	5.15099987	3.79000008	sp|Q9DBR7|M	Protein phosphatase 1 regulatory subunit 12A OS=Mus musculus GN=Ppp1r12a PE=1 SV=2	MOUSE	4	1.45881403	0.4436298	1.97696996	0.73790431	3.162277937	2.20800495	0.2391216	1.97696996	1.116863012	6.79203701	1.81970096	0.39158839	2.051162	0.88715601	5.058247089
424	6.35	6.37	32.190001	8.98699984	7.8429997	sp|Q9Z0X1|A	Apoptosis-inducing factor 1, mitochondrial OS=Mus musculus GN=Aifm1 PE=1 SV=1	MOUSE	6	0.64863437	0.2749708	1.33045399	0.474242002	0.862978518	0.55975759	0.23679631	1.40604806	0.390840888	0.787045777	0.38018939	0.25115621	1.48593605	0.267916799	0.564936996
425	6.34	6.44	22.3900005	5.46999984	4.43800017	sp|Q8C8U0|L	Liprin-beta-1 OS=Mus musculus GN=Ppfibp1 PE=1 SV=3	MOUSE	5	1.80301797	0.36629379	1.38038397	1.30617094	2.511885881	1.78648806	0.38972619	1.75388098	1.018591046	3.133285999	2.14783001	0.30912599	1.47231197	1.458814025	3.221069098
426	6.28	6.61	40.0299996	7.54700005	7.54700005	sp|Q8K1M6|D	Dynamin-1-like protein OS=Mus musculus GN=Dnm1l PE=1 SV=2	MOUSE	4	1.02801597	0.79351848	2.72897792	0.376703799	2.805433989	1.27057397	0.50787997	2.72897792	0.465586096	3.46736908	1.00925303	0.6459598	1.72186899	0.586138189	1.737800956
427	6.28	6.33	21.8899995	13.6099994	13.6099994	sp|Q8R4N0|C	Citrate lyase subunit beta-like protein, mitochondrial OS=Mus musculus GN=Clybl PE=2 SV=2	MOUSE	4	0.64863437	0.2852554	1.29419601	0.492039502	0.839460015	0.51522863	0.25012142	1.83653796	0.267916799	0.946237087	0.4207266	0.15913691	1.870682	0.205116197	0.787045777
428	6.28	6.28	30.0300002	16.1500007	13.3100003	sp|Q9NYQ2|H	Hydroxyacid oxidase 2 OS=Mus musculus GN=Hao2 PE=2 SV=1	MOUSE	3	0.66680682	0.2066007	1.34276497	0.487528503	0.895364821	0.62517268	0.67702973	1.62929595	0.373250186	1.018591046	0.41304749	0.21074121	1.61435902	0.2558586	0.666806817
429	6.28	6.28	37.6300007	19.5899993	19.5899993	sp|Q60829|P	Protein phosphatase 1 regulatory subunit 1B OS=Mus musculus GN=Ppp1r1b PE=1 SV=2	MOUSE	4	3.73250198	0.2883662	29.9226494	0.124738403	39.4457283	2.37684012	0.4381882	24.8885708	0.095499262	30.4789505	1.31825697	0.79868448	2.20800495	0.597035289	3.191538095
430	6.27	6.44	26.0199994	8.36199969	6.50399998	sp|Q9EQK5|M	Major vault protein OS=Mus musculus GN=Mvp PE=1 SV=4	MOUSE	3	1.48593605	0.3148084	2.48885703	0.597035289	5.970353127	1.95884502	0.1550817	3.1915381	0.613762021	9.20449543	3.73250198	0.04474275	2.51188588	1.485936046	16.44371986
431	6.24	6.24	21.4499995	20.07	18.3400005	sp|P42125|E	Enoyl-CoA delta isomerase 1, mitochondrial OS=Mus musculus GN=Eci1 PE=2 SV=1	MOUSE	6	0.78704578	0.38787481	1.78648806	0.401790798	1.406048059	0.77268058	0.4354963	1.85353196	0.380189389	1.432188034	1.41905701	0.30884549	1.35518897	1.047129035	2.032356977
432	6.18	6.18	30.3900003	7.71699995	7.71699995	sp|Q9R069|B	Basal cell adhesion molecule OS=Mus musculus GN=Bcam PE=2 SV=1	MOUSE	4	1.34276497	0.2263905	1.55596602	0.862978518	2.128139019	1.30617094	0.28020969	1.52756596	0.855066717	1.995262027	1.33045399	0.26492241	1.40604806	0.946237087	1.887990952
433	6.16	6.97	37.7999991	13.4000003	7.17699975	sp|Q63918|S	Serum deprivation-response protein OS=Mus musculus GN=Sdpr PE=1 SV=3	MOUSE	4	1.29419601	0.53822202	1.80301797	0.717794299	2.558585882	0.72443599	0.43042731	2.26986504	0.299226493	1.644371986	0.25822601	0.03987329	3.63077998	0.048752852	0.937561989
434	6.16	6.54	18.5499996	3.92399989	2.25900002	sp|B2RY56|R	RNA-binding protein 25 OS=Mus musculus GN=Rbm25 PE=1 SV=1	MOUSE	3	2.67916799	0.2152186	1.58489299	1.690441012	4.487453938	2.20800495	0.2502048	2.01372409	1.096477985	4.655860901	2.48885703	0.21888401	2.70395804	0.920449615	6.982324123
435	6.16	6.32	21.6700003	3.11200004	2.41400003	sp|Q6P5H2|N	Nestin OS=Mus musculus GN=Nes PE=1 SV=1	MOUSE	5	1.10662401	0.83261532	1.51356101	0.731139123	1.67494297	1.49968505	0.46588481	1.44544005	1.037528038	2.167704105	1.73780096	0.38484731	1.55596602	1.116863012	2.728977919
436	6.15	6.2	37.7900004	20.2299997	12.9800007	sp|P57759|E	Endoplasmic reticulum resident protein 29 OS=Mus musculus GN=Erp29 PE=1 SV=2	MOUSE	3	2.20800495	0.7818619	1.47231197	1.499685049	3.221069098	1.13762701	0.23957411	1.77010906	0.642687678	1.753880978	1.14815402	0.30675519	1.78648806	0.642687678	1.770109057
437	6.15	6.16	17.2600001	8.7559998	7.61400014	sp|P51125|I	Calpastatin OS=Mus musculus GN=Cast PE=1 SV=2	MOUSE	3	1.31825697	0.59478432	1.62929595	0.809095919	2.147830009	1.41905701	0.51183587	2.42102909	0.586138189	3.435580015	1.41905701	0.55300582	2.67916799	0.529663384	3.80189395
438	6.14	8.16	81.190002	25.6900013	25.6900013	sp|Q9JJW6|R	RNA and export factor-binding protein 2 OS=Mus musculus GN=Refbp2 PE=1 SV=1	MOUSE	5	1.247383	0.57488322	1.49968505	0.831763685	1.923092008	1.23594701	0.50846583	2.18776202	0.564936996	2.703958035	1.19124198	0.59285092	1.99526203	0.597035289	2.398833036
438	0	8.18	63.1399989	21.9600007	21.9600007	sp|O08583|T	THO complex subunit 4 OS=Mus musculus GN=Thoc4 PE=1 SV=3	MOUSE	5															
439	6.14	6.62	37.8600001	29.1299999	26.2100011	sp|P85094|I	Isochorismatase domain-containing protein 2A, mitochondrial OS=Mus musculus GN=Isoc2a PE=2 SV=1	MOUSE	8	0.85506672	0.67525238	1.31825697	0.648634374	1.127197981	0.8472274	0.648525	1.47231197	0.570164323	1.247382998	0.60255963	0.26779029	1.40604806	0.420726597	0.847227395
440	6.14	6.15	32.0100009	21.2200001	21.2200001	sp|O35658|C	Complement component 1 Q subcomponent-binding protein, mitochondrial OS=Mus musculus GN=C1qbp PE=	MOUSE	7	0.96382898	0.98620743	1.28233099	0.751622915	1.235947013	1.02801597	0.71177381	1.36772895	0.751622915	1.406048059	0.90364939	0.63070321	1.28233099	0.704693079	1.158776999
441	6.12	6.12	53.1599999	39.2399997	39.2399997	sp|P97816|S	Protein S100-G OS=Mus musculus GN=S100g PE=2 SV=3	MOUSE	5	0.86297852	0.4472498	1.80301797	0.4487454	1.55596602	0.62517268	0.2957899	1.870682	0.296483099	1.16949904	0.28840321	0.0973108	2.72897792	0.081658237	0.787045777
442	6.1	6.17	42.7700013	10.2899998	10.2899998	sp|P08226|A	Apolipoprotein E OS=Mus musculus GN=Apoe PE=1 SV=2	MOUSE	4	1.21338904	0.4690696	1.43218803	0.847227395	1.737800956	1.08642602	0.54149002	1.19124198	0.912010789	1.29419601	1.30617094	0.1122065	1.47231197	0.88715601	1.923092008
443	6.1	6.12	17.4600005	7.65699968	7.65699968	sp|Q64213|S	Splicing factor 1 OS=Mus musculus GN=Sf1 PE=1 SV=6	MOUSE	4	1.02801597	0.941001	1.61435902	0.636795521	1.644371986	1.33045399	0.19713371	1.27057397	1.047129035	1.721868992	0.35974929	0.1194008	1.69044101	0.212813899	0.608134985
444	6.1	6.1	65.5700028	28.2999992	28.2999992	sp|Q8K3J1|N	NADH dehydrogenase [ubiquinone] iron-sulfur protein 8, mitochondrial OS=Mus musculus GN=Ndufs8 PE=1 S	MOUSE	8	0.61944109	0.52473438	1.31825697	0.432513803	0.816582382	0.49659231	0.35314381	1.51356101	0.296483099	0.751622915	0.1294196	0.1722348	3.698282	0.060813501	0.478630096
445	6.09	6.1	41.99	12.8700003	6.99800029	sp|P36552|H	Coproporphyrinogen-III oxidase, mitochondrial OS=Mus musculus GN=Cpox PE=1 SV=2	MOUSE	3	0.76559663	0.42111799	1.38038397	0.55975759	1.056818008	0.75162292	0.35969079	1.599558	0.469894111	1.202263951	0.67297667	0.25437811	1.65958703	0.401790798	1.116863012
446	6.08	6.08	40.7900006	19.7400004	19.7400004	sp|P61089|U	Ubiquitin-conjugating enzyme E2 N OS=Mus musculus GN=Ube2n PE=1 SV=1	MOUSE	3	0.94623709	0.8682887	1.13762701	0.831763685	1.076465011	1.03752804	0.83423662	1.13762701	0.912010789	1.180320978	1.00925303	0.85532409	1.13762701	0.88715601	1.14815402
447	6.07	6.07	39.8000002	10.1999998	10.1999998	sp|Q7TNC4|L	Putative RNA-binding protein Luc7-like 2 OS=Mus musculus GN=Luc7l2 PE=1 SV=1	MOUSE	4	1.13762701	0.4541747	1.31825697	0.862978518	1.499685049	1.05681801	0.63378161	1.77010906	0.597035289	1.870682001	1.07646501	0.68197578	1.247383	0.862978518	1.342764974
448	6.04	6.15	28.9900005	9.73099992	9.73099992	sp|Q80W22|T	Threonine synthase-like 2 OS=Mus musculus GN=Thnsl2 PE=2 SV=1	MOUSE	3	0.75162292	0.51876593	1.52756596	0.474242002	1.14815402	0.4570882	0.1756413	1.78648806	0.235504895	0.816582382	0.17701089	0.0370729	2.48885703	0.058613818	0.440554887
449	6.04	6.1	15.4799998	9.2050001	9.2050001	sp|O08663|A	Methionine aminopeptidase 2 OS=Mus musculus GN=Metap2 PE=1 SV=1	MOUSE	3	0.67297667	0.51484489	2.10862804	0.322106898	1.419057012	0.72443599	0.64548743	2.77971292	0.263026804	2.013724089	0.71121353	0.56675959	2.10862804	0.340408206	1.499685049
450	6.04	6.06	35.4699999	16.8899998	16.8899998	sp|Q3TLP5|E	Enoyl-CoA hydratase domain-containing protein 2, mitochondrial OS=Mus musculus GN=Echdc2 PE=2 SV=2	MOUSE	5	0.60255963	0.1065998	1.45881403	0.405508488	0.879022479	0.66680682	0.1376313	1.38038397	0.474242002	0.920449615	0.92044962	0.80530292	1.14815402	0.801678121	1.056818008
451	6.03	9.11	56.5599978	48.3599991	43.4399992	sp|P05367|S	Serum amyloid A-2 protein OS=Mus musculus GN=Saa2 PE=1 SV=1	MOUSE	6	3.46736908	0.23380169	2.85758996	1.213389039	18.70681953	3.31131101	0.1717034	2.55858588	1.29419601	11.80321026	16.2929592	0.02354352	2.88403201	5.649370193	64.86344147
452	6.03	6.1	15.5900002	3.82299982	3.82299982	sp|Q6PGL7|F	WASH complex subunit FAM21 OS=Mus musculus GN=Fam21 PE=1 SV=1	MOUSE	4	0.87096357	0.8024748	2.0892961	0.416869402	1.819700956	0.73113912	0.57830882	2.0892961	0.349945188	1.527565956	0.76559663	0.62587339	2.0892961	0.366437614	1.599557996
453	6.03	6.1	19.5199996	10.1599999	7.61900023	sp|Q3V0K9|P	Plastin-1 OS=Mus musculus GN=Pls1 PE=2 SV=1	MOUSE	3	0.57016432	0.2880441	3.28095293	0.157036304	1.870682001	1.04712904	0.74591661	3.94457293	0.26546061	4.130475044	0.39445731	0.26848951	4.40554905	0.073113911	1.737800956

454	6.03	6.09	71.4299977	37.8199995	37.8199995	sp|Q9D1K2|V	V-type proton ATPase subunit F OS=Mus musculus GN=Atp6v1f PE=1 SV=2	MOUSE	4	1.03752804	0.33582711	1.72186899	0.602559626	1.803017974	1.03752804	0.2345243	1.72186899	0.602559626	1.786488056	0.1367729	0.01043124	2.26986504	0.065463617	0.310455889
455	6.03	6.03	18.8500002	7.35599995	7.35599995	sp|O89017|L(	Legumain OS=Mus musculus GN=Lgmn PE=1 SV=1	MOUSE	3	0.92044962	0.53254038	1.48593605	0.619441092	1.367728949	1.06659603	0.70246619	1.64437199	0.648634374	1.753880978	1.30617094	0.4770605	3.1045599	0.420726597	4.092607021
456	6.02	6.13	33.5299999	13.5299996	11.4699997	sp|Q61249|IG	Immunoglobulin-binding protein 1 OS=Mus musculus GN=Igbp1 PE=1 SV=1	MOUSE	4	1.03752804	0.54511339	1.75388098	0.591561615	1.819700956	0.89536482	0.74655533	1.51356101	0.586138189	1.355188966	0.80167812	0.73360282	1.94088602	0.413047493	1.55596602
457	6.02	6.02	34.0900004	30.3000003	30.3000003	sp|P63323|RS	40S ribosomal protein S12 OS=Mus musculus GN=Rps12 PE=1 SV=2	MOUSE	7	1.04712904	0.8561213	1.14815402	0.912010789	1.202263951	1.09647799	0.61951339	1.14815402	0.954992592	1.258924961	1.05681801	0.66523188	1.14815402	0.920449615	1.213389039
458	6.02	6.02	15.4400006	7.90999979	7.90999979	sp|Q6ZQM8|U	UDP-glucuronosyltransferase 1-7C OS=Mus musculus GN=Ugt1a7c PE=2 SV=1	MOUSE	5	0.68548822	0.29768941	1.48593605	0.452897608	1.018591046	0.63679552	0.218813	1.62929595	0.387257606	1.037528038	0.79432821	0.34353659	1.55596602	0.510505021	1.235947013
458	0.02	4.14	17.4500004	7.12900013	5.81599996	sp|P70691|UI	UDP-glucuronosyltransferase 1-2 OS=Mus musculus GN=Ugt1 PE=1 SV=1	MOUSE	4															
458	0	4.04	21.6600001	5.83800003	5.83800003	sp|Q64435|UI	UDP-glucuronosyltransferase 1-6 OS=Mus musculus GN=Ugt1a6 PE=1 SV=1	MOUSE	4															
458	0	4.04	16.0999999	7.57599995	5.87100014	sp|Q62452|UI	UDP-glucuronosyltransferase 1-9 OS=Mus musculus GN=Ugt1a9 PE=1 SV=3	MOUSE	4															
458	0	4.02	16.0699993	5.79399988	5.79399988	sp|Q63886|UI	UDP-glucuronosyltransferase 1-1 OS=Mus musculus GN=Ugt1a1 PE=2 SV=2	MOUSE	4															
459	6.02	6.02	44.9999988	41.4299995	31.4300001	sp|P62962|PI	Profilin-1 OS=Mus musculus GN=Pfn1 PE=1 SV=2	MOUSE	5	0.67920363	0.32639509	1.36772895	0.492039502	0.928966403	0.63679552	0.32333779	1.44544005	0.432513803	0.920449615	0.93756199	0.83412111	1.14815402	0.816582382	1.076465011
460	6.01	6.01	46.39	29.3799996	24.7400001	sp|Q9R0Y5|K	Adenylate kinase isoenzyme 1 OS=Mus musculus GN=Ak1 PE=1 SV=1	MOUSE	3	1.22461605	0.05209407	1.247383	0.981747925	1.541700006	1.08642602	0.09241444	1.14815402	0.946237087	1.247382998	0.86297852	0.01518577	1.28233099	0.672976673	1.106624007
461	6	12.35	23.4400004	18.4200004	18.4200004	sp|Q91WP6|S	Serine protease inhibitor A3N OS=Mus musculus GN=Serpina3n PE=1 SV=1	MOUSE	8	1.34276497	0.411625	2.80543399	0.478630096	3.80189395	1.02801597	0.88196158	4.24619579	0.242102906	4.365159035	2.85758996	0.06911127	1.85353196	1.541700006	5.395105839
462	6	8.27	42.6999986	23.6000001	19.1	sp|Q8BL97|S	Serine/arginine-rich splicing factor 7 OS=Mus musculus GN=Srsf7 PE=1 SV=1	MOUSE	5	1.07646501	0.51397198	1.48593605	0.724435985	1.644371986	0.77268058	0.99206603	1.52756596	0.492039502	1.180320978	0.32809529	0.18129981	2.12813902	0.104712903	0.698232412
463	6	6.18	26.5899986	10.8199999	10.8199999	sp|Q8VCA8|S	Secernin-2 OS=Mus musculus GN=Scrn2 PE=2 SV=1	MOUSE	4	1.09647799	0.96464932	1.92309201	0.570164323	2.089296103	1.13762701	0.73880518	1.83653796	0.619441092	2.108628035	0.97274721	0.79209918	2.24905491	0.432513803	2.187762022
464	6	6.1	26.2300015	19.2599997	19.2599997	sp|Q9CQE8|(	UPF0568 protein C14orf166 homolog OS=Mus musculus PE=2 SV=1	MOUSE	6	0.9289664	0.75366741	1.35518897	0.685488224	1.258924961	0.88715601	0.57789493	1.23594701	0.717794299	1.096477985	0.76559663	0.231594	1.38038397	0.55462569	1.056818008
465	6	6.07	58.950001	53.6800027	42.1099991	sp|P62075|TI	Mitochondrial import inner membrane translocase subunit Tim13 OS=Mus musculus GN=Timm13 PE=1 SV=1	MOUSE	6	0.9289664	0.91389942	2.10862804	0.444631308	1.958845019	1.05681801	0.8775456	2.10862804	0.501187205	2.208004951	0.80167812	0.76678431	2.0892961	0.380189389	1.67494297
466	6	6.03	40.14	36.62	36.62	sp|Q9CPQ3|T	Mitochondrial import receptor subunit TOM22 homolog OS=Mus musculus GN=Tomm22 PE=2 SV=3	MOUSE	4	0.83946002	0.79196012	1.31825697	0.636795521	1.106624007	1.08642602	0.69967347	1.247383	0.870963573	1.355188966	0.74473202	0.60480332	1.25892496	0.586138189	0.937561989
467	6	6.02	48.8200009	28.2400012	28.2400012	sp|Q9CQU0|T	Thioredoxin domain-containing protein 12 OS=Mus musculus GN=Txndc12 PE=2 SV=1	MOUSE	3	1.01859105	0.93748468	1.44544005	0.704693079	1.472311974	1.09647799	0.4807114	1.23594701	0.88715601	1.355188966	1	0.97790372	1.18032098	0.847227395	1.180320978
468	6	6.02	65.9699976	22.9200006	22.9200006	sp|Q9ERS2|N	NADH dehydrogenase [ubiquinone] 1 alpha subcomplex subunit 13 OS=Mus musculus GN=Ndufa13 PE=1 SV	MOUSE	3	0.564937	0.35457671	1.39315701	0.387257606	0.787045777	0.36643761	0.2461182	1.57036305	0.188799098	0.57543987	0.43651581	0.1976849	1.39315701	0.270395786	0.608134985
469	6	6	73.4899998	46.9900012	46.9900012	sp|P48771|CX	Cytochrome c oxidase subunit 7A2, mitochondrial OS=Mus musculus GN=Cox7a2 PE=1 SV=2	MOUSE	4	0.52966338	0.34230071	1.49968505	0.293765008	0.794328213	0.61376202	0.51011032	1.43218803	0.359749287	0.879022479	0.42461959	0.26220921	1.65958703	0.175388098	0.704693079
470	6	6	28.4799993	21.2099999	21.2099999	sp|P10518|HI	Delta-aminolevulinic acid dehydratase OS=Mus musculus GN=Alad PE=1 SV=1	MOUSE	5															
471	6	6	10.43	10.43	10.43	sp|Q9WTX5|S	S-phase kinase-associated protein 1 OS=Mus musculus GN=Skp1 PE=1 SV=3	MOUSE	5	1.11686301	0.39886779	1.27057397	0.879022479	1.419057012	1.13762701	0.4220877	1.247383	0.912010789	1.406048059	1.11686301	0.3969835	1.49968505	0.744732022	1.67494297
472	6	6	22.3800004	13.9899999	13.9899999	sp|P47754|C/	F-actin-capping protein subunit alpha-2 OS=Mus musculus GN=Capza2 PE=1 SV=3	MOUSE	4	0.77268058	0.69287008	1.55596602	0.492039502	1.202263951	0.83946002	0.76923209	1.38038397	0.602559626	1.158776999	1	0.97023553	1.43218803	0.698232412	1.432188034
473	6	6	34.8100007	20.7399994	20.7399994	sp|P16045|LE	Galectin-1 OS=Mus musculus GN=Lgals1 PE=1 SV=3	MOUSE	3	0.64268768	0.169917	1.73780096	0.380189389	1.116863012	1.69044101	0.06245068	1.65958703	1.018591046	2.91071701	2.39883304	0.04892234	1.69044101	1.419057012	4.207265854
474	6	6	58.8199973	58.8199973	58.8199973	sp|O08997|AT	Copper transport protein ATOX1 OS=Mus musculus GN=Atox1 PE=2 SV=1	MOUSE	5	1.11686301	0.78834349	1.49968505	0.744732022	1.690441012	1.49968505	0.3396807	1.19124198	1.258924961	1.976969957	1.23594701	0.56648302	1.33045399	0.928966403	1.803017974
475	5.94	5.97	25.1599997	8.3329998	6.6990003	sp|Q9DC50|C	Peroxisomal carnitine O-octanoyltransferase OS=Mus musculus GN=Crot PE=1 SV=1	MOUSE	4	1.43218803	0.54323483	8.79022503	0.162929595	12.70573997	1.70608199	0.42224211	5.15228701	0.331131101	9.036495209	1.47231197	0.51506418	21.8776207	0.067297667	23.33457947
476	5.92	5.92	33.9100003	29.4099987	24.5700002	sp|Q9EQU5|S	Protein SET OS=Mus musculus GN=Set PE=1 SV=1	MOUSE	7	1.47231197	0.40944371	1.92309201	0.765596628	2.85758996	1.57036305	0.44132751	1.95884502	0.801678121	3.076097012	1.19124198	0.46106869	1.77010906	0.672976673	2.187762022
477	5.91	5.91	46.0500002	36.1799985	27.6300013	sp|Q9CQ92|F	Mitochondrial fission 1 protein OS=Mus musculus GN=Fis1 PE=1 SV=1	MOUSE	6	0.63095743	0.47640261	2.75422907	0.226986498	1.737800956	0.30199519	0.25482899	19.2309208	0.03019952	5.80764389	0.52480751	0.38732421	2.0892961	0.248885706	1.096477985
478	5.88	5.9	39.6800011	28.3399999	25.5100012	sp|O35381|AI	Acidic leucine-rich nuclear phosphoprotein 32 family member A OS=Mus musculus GN=Anp32a PE=1 SV=1	MOUSE	5	2.12813902	0.27534509	1.52756596	1.393157005	3.221069098	1.38038397	0.49864599	1.51356101	0.912010789	2.089296103	0.93756199	0.73164368	1.58489299	0.432513803	1.485936046
479	5.86	6.58	22.6099998	6.71899989	4.26900014	sp|Q64331|M	Myosin-VI OS=Mus musculus GN=Myo6 PE=1 SV=1	MOUSE	4	0.85506672	0.73032099	1.40604806	0.608134985	1.202263951	0.73790431	0.03727866	1.72186899	0.428548515	1.270573974	0.75162292	0.58027142	1.83653796	0.40926069	1.380383968
480	5.85	5.88	22.7699995	17.8599998	15.6299993	sp|Q9D8B3|C	Charged multivesicular body protein 4b OS=Mus musculus GN=Chmp4b PE=2 SV=2	MOUSE	3	1.99526203	0.3613103	2.03235698	0.981747925	4.786301136	1.48593605	0.45769641	1.25892496	1.180320978	2.032356977	1.55596602	0.45148411	1.75388098	0.88715601	3.162277937
481	5.83	5.93	26.1000007	18.7800005	17.0699999	sp|P31428|DI	Dipeptidase 1 OS=Mus musculus GN=Dpep1 PE=1 SV=1	MOUSE	3	0.8472274	0.8289035	1.47231197	0.57543987	1.247382998	0.52966338	0.51723528	2.03235698	0.253512889	1.076465011	0.237684	0.3660599	4.57088184	0.04786301	1.08642602
482	5.83	5.93	20.5599994	6.38599992	4.2059999	sp|Q91X17|U	Uromodulin OS=Mus musculus GN=Umod PE=1 SV=1	MOUSE	3	0.83946002	0.49055579	1.69044101	0.469894111	1.419057012	1.67494297	0.356801	1.55596602	1.076465011	4.168694019	2.99226499	0.08305734	1.85353196	1.614359021	7.655965805
483	5.82	5.82	27.9799998	12.5499994	8.84800032	sp|Q9WVE8|I	Protein kinase C and casein kinase substrate in neurons protein 2 OS=Mus musculus GN=Pacsin2 PE=1 SV=1	MOUSE	3	1.27057397	0.33987039	1.35518897	0.937561989	1.737800956	0.73113912	0.70887131	1.64437199	0.39445731	1.202263951	0.83176369	0.56969088	1.11686301	0.73790431	0.928966403
484	5.81	6.06	17.6300004	9.46199969	6.49399981	sp|Q9DBF1|A	Alpha-aminoadipic semialdehyde dehydrogenase OS=Mus musculus GN=Aldh7a1 PE=1 SV=4	MOUSE	3	0.3311311	0.08128784	2.01372409	0.084722742	0.666806817	0.43651581	0.1218035	1.65958703	0.154170096	0.724435985	0.58076441	0.178214	1.33045399	0.369828194	0.772680581
485	5.8	6.76	38.2600009	26.1700004	21.48	sp|P54227|ST	Stathmin OS=Mus musculus GN=Stmn1 PE=1 SV=2	MOUSE	3	1.33045399	0.45337519	1.22461605	1.08642602	1.644371986	1.85353196	0.1825458	1.35518897	1.367728949	2.558585882	1.65958703	0.21985111	1.27057397	1.30617094	2.167704105
486	5.75	5.78	32.9400003	5.31800017	5.31800017	sp|Q61584|FX	Fragile X mental retardation syndrome-related protein 1 OS=Mus musculus GN=Fxr1 PE=1 SV=2	MOUSE	3	1.39315701	0.95200372	2.26986504	0.613762021	3.372873068	1.247383	0.37703899	2.22843504	0.55975759	2.91071701	1.69044101	0.41351759	2.26986504	0.744732022	4.168694019
487	5.69	8.11	21.1199999	9.07500014	9.07500014	sp|Q91VA0|A	Acyl-coenzyme A synthetase ACSM1, mitochondrial OS=Mus musculus GN=Acsm1 PE=1 SV=1	MOUSE	5	0.51050502	0.12944271	1.94088602	0.260615289	0.990831971	0.58076441	0.1588959	1.44544005	0.398107201	0.839460015	0.51999599	0.09627897	1.47231197	0.346736789	0.765596628
488	5.67	7.17	47.7299988	25.7600009	25.7600009	sp|P04117|FA	Fatty acid-binding protein, adipocyte OS=Mus musculus GN=Fabp4 PE=1 SV=3	MOUSE	4	0.74473202	0.64276022	1.36772895	0.529663384	1.018591046	0.81658238	0.75530732	1.40604806	0.57543987	1.14815402	0.26546061	0.13412911	2.14783001	0.130617097	0.570164323
489	5.66	5.75	28.9200008	5.2579999	4.52199988	sp|Q569Z6|TI	Thyroid hormone receptor-associated protein 3 OS=Mus musculus GN=Thrap3 PE=1 SV=1	MOUSE	6	1.92309201	0.18104079	1.51356101	1.270573974	3.076097012	1.81970096	0.1482479	1.47231197	1.235947013	2.85758996	1.44544005	0.33288959	1.39315701	1.037528038	2.187762022
490	5.66	5.71	35.0499988	20.1000005	14.4299999	sp|P62082|RS	40S ribosomal protein S7 OS=Mus musculus GN=Rps7 PE=2 SV=1	MOUSE	4	0.96382898	0.1089736	1.23594701	0.779830098	1.19124198	0.87902248	0.00518864	1.29419601	0.679203629	1.137627006	0.80909592	0.1571327	1.45881403	0.55462569	1.180320978
491	5.65	5.73	42.8600013	29.460001	26.789999	sp|Q62393|TI	Tumor protein D52 OS=Mus musculus GN=Tpd52 PE=1 SV=2	MOUSE	3	0.99083197	0.83386523	1.57036305	0.630957425	1.55596602	0.89536482	0.7384119	1.19124198	0.758577585	1.066596031	0.86297852	0.60913342	1.23594701	0.698232412	1.066596031
492	5.65	5.65	18.8899994	8.74800012	6.36200011	sp|Q8VCF0|M	Mitochondrial antiviral-signaling protein OS=Mus musculus GN=Mavs PE=1 SV=1	MOUSE	3	0.92044962	0.99444288	1.99526203	0.465586096	1.836537957	0.79432821	0.53081209	2.70395804	0.291071713	2.147830009	0.70469308	0.36856321	2.14783001	0.331131101	1.51356101
493	5.63	5.67	26.6299993	13.3200005	13.3200005	sp|Q9Z0P4|P	Paralemmin-1 OS=Mus musculus GN=Palm PE=1 SV=1	MOUSE	4	1.04712904	0.75442702	1.69044101	0.619441092	1.770109057	0.93756199	0.8259176	1.51356101	0.619441092	1.419057012	0.88715601	0.56322539	1.599558	0.55462569	1.419057012
494	5.62	5.68	63.3300006	19.63	17.4099997	sp|O35326|SI	Serine/arginine-rich splicing factor 5 OS=Mus musculus GN=Srsf5 PE=1 SV=1	MOUSE	6	1.04712904	0.87782317	1.47231197	0.711213529	1.527565956	1.22461605	0.32020351	1.25892496	0.972747207	1.770109057	1.23594701	0.4251827	1.36772895	0.90364939	1.870682001
495	5.56	5.7	17.7100003	3.15200016	1.94799993	sp|P39447|ZC	Tight junction protein ZO-1 OS=Mus musculus GN=Tjp1 PE=1 SV=2	MOUSE	3	1.29419601	0.3422825	1.83653796	0.704693079	2.443430901	1.00925303	0.60425669	1.870682	0.539510608	1.905460954	1.25892496	0.479424	1.75388098	0.717794299	2.22843504
496	5.55	5.6	32.9100013	12.6599997	9.49399993	sp|P45376|AL	Aldose reductase OS=Mus musculus GN=Akr1b1 PE=1 SV=3	MOUSE	3	0.4207266	0.09715687	2.03235698	0.175388098	0.855066717	0.2964831	0.15260591	2.29086804	0.114815399	0.679203629	0.81658238	0.8246398	1.94088602	0.424619585	1.584892988
497	5.54	5.66	32.7800006	7.40199983	6.34400025	sp|Q62167|DI	ATP-dependent RNA helicase DDX3X OS=Mus musculus GN=Ddx3x PE=1 SV=3	MOUSE	5	0.83946002	0.1224769	1.65958703	0.505824685	1.393157005	0.83946002	0.3473824	1.48593605	0.564936996	1.247382998	1	0.54853863	1.55596602	0.642687678	1.55596602
498	5.51	5.55	14.6699995	5.59800006	5.59800006	sp|O08677|KI	Kininogen-1 OS=Mus musculus GN=Kng1 PE=1 SV=1	MOUSE	4	2.07014108	0.17585871	1.99526203	1.037528038	3.981071949	2.72897792	0.04529629	1.90546095	1.432188034	5.152287006	4.83058786	0.01978802	1.88799095	2.558585882	9.036495209
499	5.51	5.52	22.2499996	11.5599997	9.8269999	sp|Q9WUM5|	Succinyl-CoA ligase [ADP/GDP-forming] subunit alpha, mitochondrial OS=Mus musculus GN=Suclg1 PE=1 SV	MOUSE	4	0.61944109	0.171368	1.45881403	0.420726597	0.90364939	0.64268768	0.19797809	1.23594701	0.524807513	0.794328213	0.62517268	0.1289451	1.23594701	0.496592313	0.772680581
500	5.51	5.51	45.8299995	39.5799995	39.5799995	sp|P18608|HI	Non-histone chromosomal protein HMG-14 OS=Mus musculus GN=Hmgn1 PE=1 SV=2	MOUSE	3	1.10662401	0.75666231	1.38038397	0.801678121	1.527565956	1.13762701	0.72265577	1.38038397	0.824138105	1.570363045	1.11686301	0.79480892	1.38038397	0.809095919	1.541700006
501	5.46	5.49	26.0399997	15.4699996	7.35799968	sp|Q80SW1|S	Putative adenosylhomocysteinase 2 OS=Mus musculus GN=Ahcyl1 PE=1 SV=1	MOUSE	3															
502	5.46	5.48	25.1100004	16.3000003	16.3000003	sp|P00405|C(	Cytochrome c oxidase subunit 2 OS=Mus musculus GN=Mtco2 PE=1 SV=1	MOUSE	3	0.33728731	0.1166133	1.52756596	0.190546095	0.515228629	0.1247384	0.0555177	1.77010906	0.048752852	0.220800504	0.33419499	0.1127261	1.41905701	0.205116197	0.474242002
503	5.45	6.14	48.5500008	30.7099998	28.2200009	sp|Q9Z1Q5|C	Chloride intracellular channel protein 1 OS=Mus musculus GN=Clic1 PE=1 SV=3	MOUSE	4	0.83946002	0.74485898	2.0892961	0.401790798	1.753880978	1.11686301	0.80543351	2.0892961	0.534564376	2.333457947	1.08642602	0.85637373	2.0892961	0.519995987	2.269865036
504	5.44	5.44	42.6099986	16.8099999	14.2000005	sp|P18894|OX	D-amino-acid oxidase OS=Mus musculus GN=Dao PE=1 SV=3	MOUSE	5	0.25118861	0.05222189	1.80301797	0.109647803	0.452897608	0.2013724	0.04333015	2.07014108	0.087902263	0.416869402	0.1294196	0.0178814	1.95884502	0.049203951	0.253512889
505	5.44	5.44	34.5499992	21.8199998	18.7900007	sp|P35979|RI	60S ribosomal protein L12 OS=Mus musculus GN=Rpl12 PE=1 SV=2	MOUSE	3	0.90364939	0.93967319	1.599558	0.564936996	1.445440054	0.85506672	0.51166838	1.247383	0.685488224	1.066596031	0.99083197	0.90835458	1.247383	0.794328213	1.235947013
506	5.39	20	38.6099994	19.9200004	16.2300006	sp|P07901|HS	Heat shock protein HSP 90-alpha OS=Mus musculus GN=Hsp90aa1 PE=1 SV=4	MOUSE	16	0.86297852	0.76269639	2.37684012	0.363078088	2.051162004	0.31915379	0.2184772	2.37684012	0.134276494	0.758577585	0.4920395	0.32187599	14.3218803	0.044463132	7.04693079
507	5.39	5.48	26.8700004	5.74200004	4.76699993	sp|Q921U8|S	Smoothelin OS=Mus musculus GN=Smtn PE=2 SV=2	MOUSE	4	0.94623709	0.78168201	1.80301797	0.529663384	1.706081986	0.86297852	0.53664953	2.01372409	0.428548515	1.737800956	1.22461605	0.49667501	2.01372409	0.608134985	2.466038942
508	5.37	7.05	26.4499992	19.28	19.28	sp|P54726|RI	UV excision repair protein RAD23 homolog A OS=Mus musculus GN=Rad23a PE=1 SV=2	MOUSE	4	1.62929595	0.4633207	2.14783001	0.758577585	3.53183198	1.27057397	0.66628933	1.38038397	0.920449615	1.753880978	1.20226395	0.73648292	3.49945211	0.343558013	4.246195793
509	5.36	5.44	79.0099978	45.6800014	30.8600009	sp|P29595|NI	NEDD8 OS=Mus musculus GN=Nedd8 PE=1 SV=2	MOUSE	5	3.80189395	0.36642799	1.65958703	2.290868044	5.970353127	3.94457293	0.3221029	1.67494297	2.355048895	6.367955208	3.28095293	0.42766962	1.72186899	1.905460954	5.445025921
510	5.31	5.31	36.0599995	13.38	13.38	sp|Q02013|A(	Aquaporin-1 OS=Mus musculus GN=Aqp1 PE=1 SV=3	MOUSE	3	0.67297667	0.53692752	1.25892496	0.515228629	0.847227395	0.40926069	0.2973246	1.49968505	0.2013724	0.613762021	0.2443431	0.1593719	1.90546095	0.077268057	0.465586096
511	5.24	5.24	22.8200004	9.73199979	7.54999965	sp|Q8BJ64|C	Choline dehydrogenase, mitochondrial OS=Mus musculus GN=Chdh PE=1 SV=1	MOUSE	3	0.564937	0.1745158	1.65958703	0.296483099	0.937561989	0.61376202	0.4028084	1.52756596	0.366437614	0.937561989	0.35974929	0.31322429	2.12813902	0.188799098	0.765596628
512	5.22	5.22	28.5699993	23.6300007	23.6300007	sp|Q9QXT0|C	Protein canopy homolog 2 OS=Mus musculus GN=Cnpy2 PE=2 SV=1	MOUSE	4	0.94623709	0.76141381	1.33045399	0.717794299	1.258924961	0.8472274	0.86656952	1.21338904	0.704693079	1.028015971	0.70469308	0.58503658	1.35518897	0.515228629	0.954992592
513	5.19	5.22	39.2100006	11.5999997	11.5999997	sp|Q9D312|K	Keratin, type I cytoskeletal 20 OS=Mus musculus GN=Krt20 PE=1 SV=1	MOUSE	3	0.99083197	0.78218633	1.43218803	0.691830993	1.419057012	1.21338904	0.48142529	1.78648806	0.679203629	2.249054909	1.47231197	0.44230649	2.37684012	0.619441092	3.40408206
514	5.17	5.18	19.9399993	6.7429997	6.59999996	sp|Q921I1|TF	Serotransferrin OS=Mus musculus GN=Tf PE=1 SV=1	MOUSE	4	1.02801597	0.84522218	1.35518897	0.758577585	1.393157005	1.09647799	0.83119792	1.55596602	0.704693079	1.706081986	2.42102909	0.3366147	1.65958703	1.458814025	4.017908096
515	5.15	5.15	34.7799987	23.3400002	16.9300005	sp|Q9DBG5|F	Perilipin-3 OS=Mus musculus GN=Plin3 PE=1 SV=1	MOUSE	4	1.13762701	0.79569137	2.51188588	0.452897608	2.85758996	1.10662401	0.84786391	2.77971292	0.398107201	3.076097012	0.96382898	0.9392221	2.0892961	0.461317599	2.013724089
516	5.11	5.11	30.7900012	14.4400001	14.4400001	sp|Q8CG76|A	Aflatoxin B1 aldehyde reductase member 2 OS=Mus musculus GN=Akr7a2 PE=1 SV=3	MOUSE	4	0.55975759	0.1540377	1.36772895	0.349945188	0.765596628	0.59156162	0.14358079	1.58489299	0.325087309	0.937561989	0.61944109	0.2075986	1.29419601	0.416869402	0.801678121
517	5.1	18.61	43.4500009	41.2600011	27.4300009	sp|Q00896|A1	Alpha-1-antitrypsin 1-3 OS=Mus musculus GN=Serpina1c PE=1 SV=2	MOUSE	14	0.9289664	0.89584673	2.0892961	0.444631308	1.940886021	0.67920363	0.50985462	2.0892961	0.322106898	1.419057012	0.82413811	0.71459073	2.0892961	0.390840888	1.721868992
517	0	18.49	38.4999991	31.9599986	26.1500001	sp|P07758|A1	Alpha-1-antitrypsin 1-1 OS=Mus musculus GN=Serpina1a PE=1 SV=4	MOUSE	14															
518	5.1	5.41	24.4900003	4.21699993	4.21699993	sp|O54988|SI	STE20-like serine/threonine-protein kinase OS=Mus musculus GN=Slk PE=1 SV=2	MOUSE	4	1.08642602	0.80536681	2.12813902	0.510505021	2.355048895	2.03235698	0.172546	2.01372409	1.009253025	4.655860901	1.20226395	0.65524697	1.94088602	0.619441092	2.511885881
519	5.1	5.1	64.7599995	38.1000012	20.95	sp|P10639|TI	Thioredoxin OS=Mus musculus GN=Txn PE=1 SV=3	MOUSE	8	1.43218803	0.42087379	1.38038397	1.037528038	1.976969957	1.43218803	0.56503052	1.38038397	1.037528038	2.013724089	1.158777	0.54500377	1.35518897	0.855066717	1.570363045
520	5.08	5.22	31.0400009	5.99600002	3.77199985	sp|Q02788|C(	Collagen alpha-2(VI) chain OS=Mus musculus GN=Col6a2 PE=2 SV=3	MOUSE	3	2.37684012	0.27735421	1.78648806	1.330453992	4.246195793	1.65958703	0.5613302	1.90546095	0.870963573	3.104559898	3.133286	0.06070831	1.78648806	1.753880978	5.597576141
521	5.08	5.08	67.6800013	65.6599999	65.6599999	sp|Q99LT0|D	Protein dpy-30 homolog OS=Mus musculus GN=Dpy30 PE=2 SV=1	MOUSE	5	1.01859105	0.94883877	2.0892961	0.487528503	2.128139019	1.33045399	0.58215892	2.0892961	0.636795521	2.805433989	1.08642602	0.83571708	2.0892961	0.519995987	2.269865036
522	5.07	5.07	33.2599998	17.8800002	16.6299999	sp|Q8K0E8|F	Fibrinogen beta chain OS=Mus musculus GN=Fgb PE=2 SV=1	MOUSE	6	1.09647799	0.75673091	1.34276497	0.816582382	2.208004951	1.55596602	0.1029372	1.247383	1.247382998	3.698282003	5.24807501	0.00158814	1.54170001	3.40408206	15.55965996
523	5.03	5.07	34.2400014	15.5900002	11.1900002	sp|Q9DCY0|K	Glycine N-acyltransferase-like protein Keg1 OS=Mus musculus GN=Keg1 PE=1 SV=1	MOUSE	3	0.36307809	0.39617401	1.73780096	0.2013724	0.630957425	0.474242	0.42581141	1.30617094	0.340408206	0.619441092	0.62517268	0.56413388	1.33045399	0.452897608	0.831763685
524	5.01	5.33	53.2500029	31.1699987	27.2700012	sp|O70591|PI	Prefoldin subunit 2 OS=Mus musculus GN=Pfdn2 PE=2 SV=2	MOUSE	3	1.04712904	0.92311537	1.57036305	0.666806817	1.644371986	1.29419601	0.53330171	1.49968505	0.862978518	1.958845019	0.97274721	0.73289013	1.52756596	0.636795521	1.485936046
525	4.98	5.06	37.0400012	7.89000019	6.76300004	sp|O88428|P/	Bifunctional 3'-phosphoadenosine 5'-phosphosulfate synthase 2 OS=Mus musculus GN=Papss2 PE=1 SV=2	MOUSE	4	0.73790431	0.30997381	1.44544005	0.505824685	1.066596031	0.95499259	0.83143067	1.62929595	0.586138189	1.55596602	0.83946002	0.51996207	1.36772895	0.613762021	1.14815402
526	4.98	5.02	42.3400015	24.4499996	13.8699994	sp|Q9Z2Y8|P	Proline synthase co-transcribed bacterial homolog protein OS=Mus musculus GN=Prosc PE=1 SV=1	MOUSE	3	1	0.35589901	1.20226395	0.831763685	1.202263951	1	0.91808307	1.14815402	0.870963573	1.14815402	0.96382898	0.69660091	1.20226395	0.801678121	1.158776999
527	4.96	13.33	30.2700013	6.10500015	4.25300002	sp|Q71LX4|TI	Talin-2 OS=Mus musculus GN=Tln2 PE=1 SV=3	MOUSE	11	1.25892496	0.26214269	1.97696996	0.636795521	2.488857031	1.38038397	0.1727265	2.01372409	0.685488224	2.779712915	1.35518897	0.38207099	2.16770411	0.625172675	2.937649965
528	4.92	5	26.3599992	5.11600003	5.11600003	sp|Q8R361|R	Rab11 family-interacting protein 5 OS=Mus musculus GN=Rab11fip5 PE=1 SV=2	MOUSE	3	2.46603894	0.21187	1.55596602	1.584892988	5.011871815	3.133286	0.24675269	1.58489299	1.976969957	6.251727104	1.02801597	0.45018381	1.55596602	0.660693526	1.905460954

529	4.92	4.93	21.8700007	6.4000003	6.4000003	sp|P00329|AI	Alcohol dehydrogenase 1 OS=Mus musculus GN=Adh1 PE=2 SV=2	MOUSE	3	0.50582469	0.1219474	1.67494297	0.304789513	0.847227395	0.70469308	0.1382917	1.40604806	0.474242002	0.990831971	0.78704578	0.2732102	1.28233099	0.597035289	1.009253025
530	4.89	4.99	17.0000002	3.90900001	2.72700004	sp|Q8CGF7|T	Transcription elongation regulator 1 OS=Mus musculus GN=Tcerg1 PE=1 SV=2	MOUSE	4	1.58489299	0.1289036	1.85353196	0.855066717	2.992264986	1.16949904	0.46925601	1.52756596	0.765596628	1.836537957	1.39315701	0.1707418	1.54170001	0.90364939	2.187762022
531	4.86	4.86	27.5599986	16.7600006	9.65899974	sp|P24452|C	Macrophage-capping protein OS=Mus musculus GN=Capg PE=1 SV=2	MOUSE	3	1.29419601	0.2689862	3.872576	0.334194988	5.395105839	1.47231197	0.2124455	4.28548479	0.343558013	6.606935024	2.51188588	0.1233399	4.48745394	0.55975759	11.58777046
532	4.85	4.87	21.6000006	7.34300017	7.34300017	sp|Q07076|AI	Annexin A7 OS=Mus musculus GN=Anxa7 PE=2 SV=2	MOUSE	3	0.80909592	0.70076102	1.43218803	0.529663384	1.158776999	1.158777	0.39252469	1.18032098	0.981747925	1.406048059	1.97696996	0.1252012	1.28233099	1.541700006	2.754229069
533	4.8	5	17.3700005	4.16299999	2.48199999	sp|Q8K1N2|P	Pleckstrin homology-like domain family B member 2 OS=Mus musculus GN=Phldb2 PE=1 SV=2	MOUSE	3	1.31825697	0.46373281	10.5681801	0.124738403	13.93157005	1.30617094	0.36483511	2.80543399	0.465586096	3.698282003	1.03752804	0.91656292	2.0892961	0.496592313	2.167704105
534	4.8	4.88	36.4100009	18.2099998	12.6599997	sp|Q61081|C	Hsp90 co-chaperone Cdc37 OS=Mus musculus GN=Cdc37 PE=2 SV=1	MOUSE	6	1.02801597	0.91021431	1.54170001	0.666806817	1.584892988	0.97274721	0.8624056	1.49968505	0.648634374	1.458814025	1.11686301	0.60474622	1.19124198	0.937561989	1.330453992
535	4.79	7.05	33.5500002	19.5999995	17.6100001	sp|Q9Z130|H	Heterogeneous nuclear ribonucleoprotein D-like OS=Mus musculus GN=Hnrpdl PE=1 SV=1	MOUSE	6	1.247383	0.68994212	6.02559614	0.207014099	7.516229153	0.99083197	0.99057329	2.0892961	0.474242002	2.070141077	0.94623709	0.89423299	2.0892961	0.452897608	1.976969957
536	4.76	4.86	67.110002	59.2100024	19.7400004	sp|Q9CPQ1|(	Cytochrome c oxidase subunit 6C OS=Mus musculus GN=Cox6c PE=1 SV=3	MOUSE	2	1.23594701	0.50572872	1.41905701	0.870963573	1.786488056	0.97274721	0.66417491	1.39315701	0.704693079	1.355188966	0.83946002	0.54032219	1.35518897	0.613762021	1.137627006
537	4.76	4.84	16.3800001	4.06499989	3.25199999	sp|Q8K4G5|A	Actin-binding LIM protein 1 OS=Mus musculus GN=Ablim1 PE=1 SV=1	MOUSE	3	4.7424202	0.1308714	5.15228701	0.920449615	22.49054909	4.61317587	0.0771224	5.15228701	0.895364821	21.8776207	3.90840912	0.02261555	5.19996023	0.751622915	18.70681953
538	4.76	4.81	18.75	4.09500003	4.09500003	sp|Q91YT0|N	NADH dehydrogenase [ubiquinone] flavoprotein 1, mitochondrial OS=Mus musculus GN=Ndufv1 PE=1 SV=1	MOUSE	4	0.61944109	0.28179339	1.29419601	0.461317599	0.801678121	0.35974929	0.03984364	1.41905701	0.235504895	0.510505021	0.54450268	0.05605572	1.25892496	0.39445731	0.685488224
539	4.75	4.82	14.8800001	4.41399999	4.41399999	sp|Q99KC8|V	von Willebrand factor A domain-containing protein 5A OS=Mus musculus GN=Vwa5a PE=1 SV=2	MOUSE	3	1.19124198	0.73302972	2.10862804	0.564936996	2.488857031	0.54954088	0.38937151	2.0892961	0.260615289	1.14815402	0.8472274	0.76232922	2.0892961	0.401790798	1.770109057
540	4.74	4.78	35.2200002	11.8299998	11.8299998	sp|Q9EPC1|F	Alpha-parvin OS=Mus musculus GN=Parva PE=1 SV=1	MOUSE	4	1.33045399	0.73730612	1.25892496	1.056818008	1.690441012	1.22461605	0.8305558	1.34276497	0.912010789	1.644371986	1.33045399	0.75538999	1.35518897	0.981747925	1.803017974
541	4.71	4.71	16.0600007	7.29900003	7.29900003	sp|O54941|SI	SWI/SNF-related matrix-associated actin-dependent regulator of chromatin subfamily E member 1 OS=Mus mu	MOUSE	3	4.09260702	0.04545556	4.69894123	0.870963573	18.87990952	3.59749293	0.06284192	4.83058786	0.744732022	17.86487961	3.34194994	0.06229585	4.78630114	0.698232412	16.29295921
542	4.7	4.7	53.5099983	26.3200015	21.0500002	sp|P47955|RI	60S acidic ribosomal protein P1 OS=Mus musculus GN=Rplp1 PE=1 SV=1	MOUSE	6	0.99083197	0.73556173	1.28233099	0.772680581	1.270573974	0.90364939	0.84855467	1.19124198	0.751622915	1.076465011	1.06659603	0.73066771	1.13762701	0.937561989	1.213389039
543	4.69	4.74	19.4800004	5.93699999	5.93699999	sp|P80315|T(	T-complex protein 1 subunit delta OS=Mus musculus GN=Cct4 PE=1 SV=3	MOUSE	3	1	0.74269098	1.35518897	0.73790431	1.355188966	1.07646501	0.65430808	1.21338904	0.88715601	1.30617094	1.33045399	0.4063569	1.44544005	0.920449615	1.905460954
544	4.68	4.7	28.4999996	23.4999999	23.0000004	sp|Q9JM14|N	5'(3')-deoxyribonucleotidase, cytosolic type OS=Mus musculus GN=Nt5c PE=1 SV=1	MOUSE	2															
545	4.64	4.95	18.4200004	2.733	2.02399995	sp|O70318|E	Band 4.1-like protein 2 OS=Mus musculus GN=Epb41l2 PE=1 SV=1	MOUSE	2	1.55596602	0.42511201	2.58225989	0.602559626	3.981071949	1.29419601	0.57587272	2.4434309	0.529663384	3.162277937	1.77010906	0.36744541	2.10862804	0.839460015	3.698282003
546	4.64	4.64	24.1899997	11.1599997	5.58100008	sp|P63158|HI	High mobility group protein B1 OS=Mus musculus GN=Hmgb1 PE=1 SV=2	MOUSE	2	1.58489299	0.4519189	1.47231197	1.076465011	2.91071701	1.16949904	0.93971407	1.45881403	0.801678121	1.923092008	0.75857759	0.73994082	1.51356101	0.452897608	1.14815402
546	0	4.3	28.5699993	8.57099965	5.71400002	sp|P30681|HI	High mobility group protein B2 OS=Mus musculus GN=Hmgb2 PE=1 SV=3	MOUSE	2															
547	4.62	4.62	30.5700004	27.4599999	19.1699997	sp|Q60648|S	Ganglioside GM2 activator OS=Mus musculus GN=Gm2a PE=1 SV=2	MOUSE	4	1.28233099	0.1729587	1.48593605	0.862978518	1.905460954	1.28233099	0.16170751	1.38038397	0.928966403	1.770109057	1.07646501	0.78234202	1.247383	0.862978518	1.342764974
548	4.52	4.6	14.35	7.98799992	5.91700003	sp|Q9QXX4|(	Calcium-binding mitochondrial carrier protein Aralar2 OS=Mus musculus GN=Slc25a13 PE=1 SV=1	MOUSE	2	0.58076441	0.43697089	21.8776207	0.040179081	12.70573997	0.52480751	0.37973869	19.5884495	0.039810721	10.28015995	0.76559663	0.64917189	2.0892961	0.363078088	1.599557996
549	4.52	4.52	42.9100007	7.61199966	5.53600006	sp|P23492|PI	Purine nucleoside phosphorylase OS=Mus musculus GN=Pnp PE=1 SV=2	MOUSE	2	0.4613176	0.2383168	1.599558	0.277971298	0.73790431	0.2355049	0.03222203	1.70608199	0.120226398	0.401790798	0.57016432	0.32211009	1.25892496	0.4487454	0.717794299
550	4.51	4.55	17.5300002	6.61899969	5.18799983	sp|Q64516|G	Glycerol kinase OS=Mus musculus GN=Gk PE=2 SV=2	MOUSE	3	0.76559663	0.5259043	1.30617094	0.570164323	1	0.63095743	0.31104949	1.67494297	0.35318321	1.056818008	0.58613819	0.44514719	1.90546095	0.291071713	1.116863012
551	4.5	6.75	20.7399994	6.26299977	3.18300016	sp|P17710|H	Hexokinase-1 OS=Mus musculus GN=Hk1 PE=1 SV=3	MOUSE	4	0.40550849	0.1230941	2.26986504	0.170608193	0.920449615	0.3981072	0.05859707	2.20800495	0.162929595	0.879022479	0.66069353	0.30778721	2.16770411	0.301995188	1.432188034
552	4.47	4.54	14.7	2.23600008	1.829	sp|P28665|M	Murinoglobulin-1 OS=Mus musculus GN=Mug1 PE=1 SV=3	MOUSE	2	1.14815402	0.81523049	1.599558	0.717794299	1.853531957	1.41905701	0.46323481	2.22843504	0.636795521	3.162277937	2.10862804	0.30703589	1.38038397	1.527565956	2.937649965
552	0	2.37	10.1999998	1.37799997	0.96490001	sp|P28666|M	Murinoglobulin-2 OS=Mus musculus GN=Mug2 PE=2 SV=2	MOUSE	1															
553	4.46	4.63	26.3500005	19.6500003	9.50300023	sp|Q91WD5|	NADH dehydrogenase [ubiquinone] iron-sulfur protein 2, mitochondrial OS=Mus musculus GN=Ndufs2 PE=1 S	MOUSE	3	0.44463131	0.28986439	2.10862804	0.212813899	0.937561989	0.55975759	0.38263831	2.0892961	0.267916799	1.16949904	0.87096357	0.79958642	2.0892961	0.416869402	1.819700956
554	4.43	4.62	19.3800002	3.74399982	2.86299996	sp|P97927|L	Laminin subunit alpha-4 OS=Mus musculus GN=Lama4 PE=1 SV=2	MOUSE	3	1.158777	0.8298288	1.73780096	0.666806817	2.032356977	1.78648806	0.39561161	2.39883304	0.744732022	4.405549049	1.99526203	0.3368431	2.051162	0.972747207	4.168694019
555	4.41	4.44	47.3699987	28.9499998	21.7099994	sp|Q80Y14|G	Glutaredoxin-related protein 5, mitochondrial OS=Mus musculus GN=Glrx5 PE=2 SV=2	MOUSE	4	1.34276497	0.57114023	1.35518897	0.990831971	2.070141077	1.12719798	0.6226269	1.33045399	0.847227395	1.599557996	0.58613819	0.84042192	1.88799095	0.190546095	1.106624007
556	4.36	5.1	34.3400002	16.8400005	14.8100004	sp|P52196|T	Thiosulfate sulfurtransferase OS=Mus musculus GN=Tst PE=1 SV=3	MOUSE	3	0.78704578	0.46624589	1.55596602	0.505824685	1.224616051	0.54450268	0.27813599	2.18776202	0.248885706	1.19124198	0.72443599	0.37593499	1.35518897	0.534564376	0.981747925
557	4.35	4.37	17.2199994	9.27200019	5.51900007	sp|P16331|P	Phenylalanine-4-hydroxylase OS=Mus musculus GN=Pah PE=1 SV=4	MOUSE	5	0.34673679	0.226459	2.0892961	0.164437205	0.724435985	0.4786301	0.31498459	2.10862804	0.229086801	1.009253025	0.1191242	0.1160875	2.0892961	0.0564937	0.248885706
558	4.34	4.34	94.3899989	62.620002	37.380001	sp|Q9QUH0|(	Glutaredoxin-1 OS=Mus musculus GN=Glrx PE=1 SV=3	MOUSE	6	0.87902248	0.59079558	1.51356101	0.57543987	1.330453992	1.01859105	0.95268953	1.27057397	0.801678121	1.29419601	0.87902248	0.63397902	1.78648806	0.487528503	1.570363045
559	4.32	4.32	31.7400008	10.08	8.31200033	sp|Q8CAY6|T	Acetyl-CoA acetyltransferase, cytosolic OS=Mus musculus GN=Acat2 PE=1 SV=2	MOUSE	3	0.85506672	0.59591007	1.70608199	0.432513803	1.458814025	1.40604806	0.15327661	1.54170001	0.912010789	3.076097012	2.01372409	0.05148413	1.99526203	1.009253025	5.395105839
560	4.31	4.71	12.7000004	0.7187	0.33819999	sp|Q91XQ0|D	Dynein heavy chain 8, axonemal OS=Mus musculus GN=Dnahc8 PE=2 SV=2	MOUSE	2	1.03752804	0.9615227	1.30617094	0.794328213	1.355188966	0.94623709	0.7902438	1.29419601	0.731139123	1.224616051	0.94623709	0.82920152	1.247383	0.758577585	1.180320978
561	4.3	4.77	13.9899999	2.35399995	2.08500009	sp|P55937|G	Golgin subfamily A member 3 OS=Mus musculus GN=Golga3 PE=1 SV=3	MOUSE	3	0.87902248	0.819188	9.5499258	0.092044957	8.394599915	0.69823241	0.56272483	21.4783001	0.042461962	14.99685001	1.12719798	0.80346209	7.04693079	0.1599558	7.943282127
562	4.3	4.33	40.0999993	18.2699993	18.2699993	sp|O88952|LI	Protein lin-7 homolog C OS=Mus musculus GN=Lin7c PE=1 SV=2	MOUSE	4	0.93756199	0.72748739	1.20226395	0.779830098	1.127197981	1.01859105	0.49895909	1.14815402	0.88715601	1.16949904	0.97274721	0.05998111	1.16949904	0.831763685	1.137627006
563	4.28	4.28	62.7300024	47.27	41.8199986	sp|Q9Z172|S	Small ubiquitin-related modifier 3 OS=Mus musculus GN=Sumo3 PE=2 SV=1	MOUSE	3	2.31206489	0.1821308	1.38038397	1.67494297	3.191538095	2.53512907	0.17058	1.38038397	1.836537957	3.564511061	1.62929595	0.55909509	1.48593605	1.096477985	2.249054909
563	0	4.28	72.6300001	54.7399998	48.4200001	sp|P61957|S	Small ubiquitin-related modifier 2 OS=Mus musculus GN=Sumo2 PE=2 SV=1	MOUSE	3															
564	4.27	4.27	31.4799994	31.4799994	31.4799994	sp|P48428|T	Tubulin-specific chaperone A OS=Mus musculus GN=Tbca PE=2 SV=3	MOUSE	7	2.22843504	0.45598471	1.73780096	1.28233099	28.84030914	2.93764997	0.36644211	2.10862804	1.393157005	35.31832123	2.31206489	0.44802651	1.77010906	1.30617094	32.80952835
565	4.26	4.34	20.9999993	4.58699986	2.77600009	sp|Q9JKF1|I(	Ras GTPase-activating-like protein IQGAP1 OS=Mus musculus GN=Iqgap1 PE=1 SV=1	MOUSE	3	0.63095743	0.4683688	3.66437602	0.173780099	2.312064886	0.8472274	0.74532819	3.66437602	0.229086801	3.104559898	0.88715601	0.84701431	2.10862804	0.424619585	1.870682001
566	4.26	4.26	31.130001	12.06	9.33900029	sp|P20108|P	Thioredoxin-dependent peroxide reductase, mitochondrial OS=Mus musculus GN=Prdx3 PE=1 SV=1	MOUSE	3	0.63679552	0.3323887	1.247383	0.416869402	0.794328213	0.59703529	0.2762852	1.43218803	0.313328594	0.855066717	0.31622779	0.13240971	1.94088602	0.04965923	0.613762021
567	4.25	4.56	19.2100003	4.41000015	3.43800001	sp|Q9JIX8|A(	Apoptotic chromatin condensation inducer in the nucleus OS=Mus musculus GN=Acin1 PE=1 SV=3	MOUSE	5	1.13762701	0.6514166	1.22461605	0.928966403	1.393157005	1.13762701	0.84242213	1.27057397	0.895364821	1.458814025	0.87096357	0.53107607	1.30617094	0.660693526	1.137627006
568	4.23	4.23	45.3299999	19.0300003	16.9599995	sp|Q9CWE0|I	Protein FAM54B OS=Mus musculus GN=Fam54b PE=1 SV=1	MOUSE	4	1.81970096	0.49497521	1.92309201	0.946237087	3.767038107	1.58489299	0.61722308	1.58489299	1	2.779712915	0.85506672	0.9662115	1.99526203	0.416869402	1.706081986
569	4.22	7.06	24.1400003	3.68500017	3.02300006	sp|P08032|S	Spectrin alpha chain, erythrocyte OS=Mus musculus GN=Spta1 PE=2 SV=3	MOUSE	4	1.20226395	0.68181121	2.6546061	0.452897608	3.191538095	1.18032098	0.70446682	2.0892961	0.564936996	2.466038942	1.54170001	0.4258633	3.56451106	0.432513803	5.495409012
570	4.21	4.21	41.2200004	11.84	11.84	sp|Q8JZV9|B	3-hydroxybutyrate dehydrogenase type 2 OS=Mus musculus GN=Bdh2 PE=1 SV=1	MOUSE	4	0.67920363	0.1752598	1.25892496	0.529663384	0.855066717	0.64863437	0.18187361	1.19124198	0.534564376	0.772680581	0.61944109	0.1355675	1.16949904	0.519995987	0.724435985
571	4.19	6.17	39.1099989	12.2599997	12.2599997	sp|Q99K48|N	Non-POU domain-containing octamer-binding protein OS=Mus musculus GN=Nono PE=1 SV=3	MOUSE	4	1.08642602	0.33278731	1.52756596	0.711213529	1.659587026	1.247383	0.1582035	1.75388098	0.711213529	2.187762022	1.30617094	0.03925358	1.70608199	0.765596628	2.22843504
572	4.19	4.2	37.5	12.19	5.62499985	sp|Q9Z1D1|E	Eukaryotic translation initiation factor 3 subunit G OS=Mus musculus GN=Eif3g PE=1 SV=2	MOUSE	2	1.10662401	0.59662771	1.19124198	0.928966403	1.318256974	1.07646501	0.75811929	1.20226395	0.895364821	1.29419601	1.07646501	0.84750837	1.21338904	0.88715601	1.30617094
573	4.18	4.6	30.2300006	3.94599997	2.60300003	sp|P59242|C	Cingulin OS=Mus musculus GN=Cgn PE=1 SV=1	MOUSE	3	2.42102909	0.24044851	1.77010906	1.367728949	4.325138092	2.51188588	0.2263424	1.78648806	1.406048059	4.446312904	2.70395804	0.38696811	1.78648806	1.51356101	4.830587864
574	4.17	4.64	11.97	9.97800008	9.97800008	sp|Q99JX3|G	Golgi reassembly-stacking protein 2 OS=Mus musculus GN=Gorasp2 PE=1 SV=3	MOUSE	3	0.97274721	0.97427768	4.20726585	0.229086801	4.092607021	0.54450268	0.38988459	2.10862804	0.260615289	1.14815402	0.66069353	0.50561291	7.51622915	0.087902263	4.965922832
575	4.14	4.18	14.8399994	7.03099966	3.90600003	sp|P26638|S	Seryl-tRNA synthetase, cytoplasmic OS=Mus musculus GN=Sars PE=2 SV=3	MOUSE	2	0.83946002	0.27714241	1.247383	0.666806817	1.047129035	0.82413811	0.35994741	1.23594701	0.666806817	1.018591046	1.01859105	0.75228179	1.18032098	0.862978518	1.202263951
576	4.14	4.17	70.7099974	31.310001	24.2400006	sp|Q9CQ75|	NADH dehydrogenase [ubiquinone] 1 alpha subcomplex subunit 2 OS=Mus musculus GN=Ndufa2 PE=1 SV=3	MOUSE	3	0.92044962	0.4191125	1.158777	0.794328213	1.066596031	0.9289664	0.17673869	1.11686301	0.831763685	1.037528038	0.94623709	0.61956471	1.13762701	0.831763685	1.076465011
577	4.13	4.48	9.52399969	4.19000015	3.04799993	sp|Q8BMP6|(	Golgi resident protein GCP60 OS=Mus musculus GN=Acbd3 PE=1 SV=3	MOUSE	3	1.20226395	0.71579331	2.70395804	0.444631308	3.28095293	1.23594701	0.76666522	4.69894123	0.263026804	5.754398823	1.21338904	0.75367791	3.73250198	0.325087309	4.570881844
578	4.13	4.25	12.0700002	3.29300016	2.63400003	sp|Q8R1B4|E	Eukaryotic translation initiation factor 3 subunit C OS=Mus musculus GN=Eif3c PE=1 SV=1	MOUSE	2	1.13762701	0.66680843	1.28233099	0.88715601	1.458814025	1.45881403	0.30580589	1.35518897	1.076465011	2.013724089	1.55596602	0.28217289	1.39315701	1.116863012	2.249054909
579	4.13	4.21	38.1500006	4.95700017	4.95700017	sp|Q91YI0|A	Argininosuccinate lyase OS=Mus musculus GN=Asl PE=2 SV=1	MOUSE	2	0.69823241	0.21351141	1.72186899	0.401790798	1.202263951	0.66069353	0.09756591	1.35518897	0.48305881	0.895364821	0.80167812	0.25166419	1.27057397	0.630957425	1.018591046
580	4.13	4.17	31.1800003	20.1499999	20.1499999	sp|Q66JS6|E	Eukaryotic translation initiation factor 3 subunit J OS=Mus musculus GN=Eif3j PE=2 SV=1	MOUSE	3	1.41905701	0.39081579	2.80543399	0.505824685	4.285484791	1.25892496	0.47432351	2.80543399	0.4487454	3.66437602	0.4168694	0.93625629	3.07609701	0.065463617	1.28233099
581	4.12	4.12	26.3399988	11.1599997	8.48200023	sp|Q62446|F	Peptidyl-prolyl cis-trans isomerase FKBP3 OS=Mus musculus GN=Fkbp3 PE=1 SV=2	MOUSE	2	1.30617094	0.36218759	1.54170001	0.847227395	2.013724089	1.36772895	0.29084149	1.36772895	1	1.870682001	1.19124198	0.38743171	1.18032098	1.009253025	1.406048059
582	4.11	4.27	24.5000005	4.45399992	3.28699984	sp|A6H6E2|M	Multimerin-2 OS=Mus musculus GN=Mmrn2 PE=2 SV=1	MOUSE	2															
583	4.11	4.11	18.4400007	13.8699994	5.58400005	sp|Q8VI36|P	Paxillin OS=Mus musculus GN=Pxn PE=1 SV=1	MOUSE	3	1.01859105	0.9884007	2.10862804	0.48305881	2.128139019	0.87096357	0.8752647	2.0892961	0.413047493	1.819700956	0.25822601	0.53320932	29.3764992	0.021478301	7.585775852
584	4.11	4.11	17.5600007	7.25200027	5.34400009	sp|P09242|P	Alkaline phosphatase, tissue-nonspecific isozyme OS=Mus musculus GN=Alpl PE=1 SV=2	MOUSE	2	1.16949904	0.7151615	1.52756596	0.765596628	2.013724089	0.97274721	0.96378422	1.599558	0.549540877	1.55596602	0.59156162	0.50577092	1.80301797	0.319153786	1.066596031
585	4.11	4.11	39.4899994	31.279999	31.279999	sp|P70444|B	BH3-interacting domain death agonist OS=Mus musculus GN=Bid PE=1 SV=2	MOUSE	4	1.95884502	0.3346363	24.6603909	0.079432823	27.79713058	1.58489299	0.44636262	22.6986504	0.069823243	24.66039085	0.72443599	0.56183893	21.2813892	0.043651581	15.41699982
586	4.1	4.1	42.5900012	25	25	sp|P26883|F	Peptidyl-prolyl cis-trans isomerase FKBP1A OS=Mus musculus GN=Fkbp1a PE=2 SV=2	MOUSE	2	1.27057397	0.42848599	1.62929595	0.779830098	2.128139019	1.12719798	0.70827907	1.45881403	0.772680581	1.770109057	1.13762701	0.65398628	1.39315701	0.816582382	1.706081986
587	4.09	4.28	14.8300007	7.41600022	6.51699975	sp|O09173|H	Homogentisate 1,2-dioxygenase OS=Mus musculus GN=Hgd PE=1 SV=2	MOUSE	2	0.78704578	0.2038932	1.51356101	0.510505021	1.19124198	0.63679552	0.08428039	1.21338904	0.496592313	0.772680581	0.50118721	0.2658065	1.70608199	0.26546061	0.855066717
588	4.09	4.12	52.2499979	31.0799986	18.0199996	sp|P09671|S(	Superoxide dismutase [Mn], mitochondrial OS=Mus musculus GN=Sod2 PE=1 SV=3	MOUSE	4	0.57543987	0.04078968	1.39315701	0.398107201	0.801678121	0.64863437	0.040728	1.18032098	0.539510608	0.765596628	0.58613819	0.02020571	1.36772895	0.401790798	0.801678121
589	4.08	4.15	15.23	5.02399989	4.08199988	sp|Q9JLQ0|C	CD2-associated protein OS=Mus musculus GN=Cd2ap PE=1 SV=3	MOUSE	2	1.07646501	0.88333768	1.49968505	0.717794299	1.614359021	1.22461605	0.50560647	1.38038397	0.88715601	1.690441012	1.14815402	0.63923109	1.38038397	0.831763685	1.584892988
590	4.07	13.43	38.1700009	29.2400003	23.8800004	sp|P68368|T	Tubulin alpha-4A chain OS=Mus musculus GN=Tuba4a PE=1 SV=1	MOUSE	15	0.2831392	0.82730198	2.18776202	0.144544005	0.619441092	0.57016432	0.8909449	1.69044101	0.316227794	0.963828981	0.42854852	0.58473092	1.85353196	0.205116197	0.794328213
591	4.07	4.35	10.87	4.44200002	3.87500003	sp|Q02053|U	Ubiquitin-like modifier-activating enzyme 1 OS=Mus musculus GN=Uba1 PE=1 SV=1	MOUSE	3	0.72443599	0.5715065	2.0892961	0.346736789	1.51356101	1.06659603	0.88228577	2.0892961	0.510505021	2.22843504	1.19124198	0.71928632	2.0892961	0.570164323	2.488857031
592	4.07	4.16	25.2400011	3.95600013	2.72899997	sp|Q9R1Z8|V	Vinexin OS=Mus musculus GN=Sorbs3 PE=1 SV=1	MOUSE	2	1.07646501	0.87533307	2.10862804	0.510505021	2.249054909	0.8472274	0.75862187	2.0892961	0.401790798	1.770109057	1.13762701	0.7818616	2.48885703	0.457088202	2.85758996
593	4.07	4.16	29.9499989	11.9800001	9.21700001	sp|Q99LP6|G	GrpE protein homolog 1, mitochondrial OS=Mus musculus GN=Grpel1 PE=1 SV=1	MOUSE	3	1.62929595	0.3722899	1.78648806	0.912010789	2.91071701	1.45881403	0.40632749	1.78648806	0.816582382	2.606153011	1.13762701	0.80755037	1.77010906	0.642687678	2.032356977
594	4.06	4.15	15.3699994	4.62599993	2.99299993	sp|Q9QYC0|	Alpha-adducin OS=Mus musculus GN=Add1 PE=1 SV=2	MOUSE	2	2.20800495	0.35615331	1.77010906	1.247382998	5.861382008	2.46603894	0.267203	1.78648806	1.380383968	6.606935024	1.75388098	0.78138077	1.73780096	1.009253025	4.207265854
595	4.06	4.1	18.9300001	3.91700007	3.91700007	sp|Q99LB7|S	Sarcosine dehydrogenase, mitochondrial OS=Mus musculus GN=Sardh PE=1 SV=1	MOUSE	2	0.2558586	0.1788154	2.18776202	0.098174803	0.55975759	0.31622779	0.2170376	28.0543404	0.02558586	8.871560097	0.2355049	0.16967499	27.5422897	0.022080051	6.486343861
596	4.05	4.31	51.9599974	21.0800007	15.1999995	sp|Q99PT1|G	Rho GDP-dissociation inhibitor 1 OS=Mus musculus GN=Arhgdia PE=1 SV=3	MOUSE	5	1.22461605	0.80638361	1.40604806	0.870963573	1.721868992	1.35518897	0.87809402	1.40604806	0.963828981	1.905460954	1.69044101	0.69348413	1.41905701	1.19124198	2.376840115
597	4.05	4.12	25.6900013	5.6880001	5.6880001	sp|P80318|T(	T-complex protein 1 subunit gamma OS=Mus musculus GN=Cct3 PE=1 SV=1	MOUSE	2	0.83176369	0.61708331	2.10862804	0.398107201	1.753880978	0.92044962	0.98818553	2.96483111	0.310455889	2.728977919	0.87096357	0.72122192	2.0892961	0.416869402	1.819700956
598	4.05	4.12	14.4199997	4.24900018	4.24900018	sp|P55096|A	ATP-binding cassette sub-family D member 3 OS=Mus musculus GN=Abcd3 PE=1 SV=2	MOUSE	2	0.29376501	0.00296003	2.39883304	0.089536473	0.704693079	0.33419499	0.01547355	2.26986504	0.04965923	0.758577585	0.69823241	0.30397061	1.65958703	0.390840888	1.158776999
599	4.05	4.11	15.67	2.08899993	2.08899993	sp|Q62470|IT	Integrin alpha-3 OS=Mus musculus GN=Itga3 PE=1 SV=1	MOUSE	3	1.14815402	0.77485329	1.25892496	0.912010789	1.445440054	1.16949904	0.81332898	1.22461605	0.954992592	1.419057012	1.92309201	0.4823764	1.36772895	1.406048059	2.630268097
600	4.05	4.05	40.7999992	16.7999998	16.7999998	sp|P62852|R	40S ribosomal protein S25 OS=Mus musculus GN=Rps25 PE=2 SV=1	MOUSE	2	1.57036305	0.41640711	1.78648806	0.879022479	3.34194994	1.10662401	0.89800948	1.58489299	0.698232412	1.976969957	1.58489299	0.34709641	1.599558	0.990831971	3.908409119
601	4.04	4.16	17.4199998	6.65400028	4.89200018	sp|Q64462|C	Cytochrome P450 4B1 OS=Mus musculus GN=Cyp4b1 PE=1 SV=1	MOUSE	2	0.8472274	0.54064989	4.4463129	0.190546095	3.767038107	0.08317637	0.1116346	4.48745394	0.02128139	0.373250186	0.4017908	0.28448981	2.67916799	0.130617097	1.076465011
602	4.04	4.11	15.6200007	2.83300001	2.06700005	sp|Q5SV85|S	Synergin gamma OS=Mus musculus GN=Synrg PE=1 SV=1	MOUSE	2	1.23594701	0.72393203	1.39315701	0.88715601	1.721868992	0.57016432	0.5207122	2.12813902	0.195884496	1.213389039	0.51522863	0.43325159	1.599558	0.149968505	0.824138105
603	4.04	4.1	19.4399998	5.79399988	4.11200002	sp|Q8BKX1|E	Brain-specific angiogenesis inhibitor 1-associated protein 2 OS=Mus musculus GN=Baiap2 PE=1 SV=2	MOUSE	2	1.38038397	0.15698659	1.47231197	0.937561989	2.032356977	1.48593605	0.1515511	1.78648806	0.831763685	2.679167986	1.31825697	0.1724771	1.52756596	0.862978518	2.032356977
604	4.04	4.06	17.9299995	7.60900006	7.60900006	sp|Q99JI6|R	Ras-related protein Rap-1b OS=Mus musculus GN=Rap1b PE=2 SV=2	MOUSE	3	0.89536482	0.83566809	2.0892961	0.428548515	1.870682001	0.8472274	0.76016581	2.0892961	0.405508488	1.770109057	0.95499259	0.94445658	2.0892961	0.457088202	1.995262027
604	0	4.06	17.9299995	7.60900006	7.60900006	sp|P62835|R	Ras-related protein Rap-1A OS=Mus musculus GN=Rap1a PE=2 SV=1	MOUSE	3															

605	4.03	8.06	36.0700011	14.7699997	11.4100002	sp|Q99NB8|U	Ubiquilin-4 OS=Mus musculus GN=Ubqln4 PE=1 SV=1	MOUSE	4																
606	4.03	4.17	30.5599988	13.1400004	13.1400004	sp|Q9DCS3|M	Trans-2-enoyl-CoA reductase, mitochondrial OS=Mus musculus GN=Mecr PE=2 SV=2	MOUSE	2	0.61944109	0.3374916	1.97696996	0.293765008	1.224616051	1.12719798	0.80803788	1.38038397	0.816582382	1.55596602	0.89536482	0.819951	1.38038397	0.648634374	1.235947013
607	4.03	4.1	26.1400014	17.6100001	14.2000005	sp|Q9DCS9|N	NADH dehydrogenase [ubiquinone] 1 beta subcomplex subunit 10 OS=Mus musculus GN=Ndufb10 PE=1 SV=	MOUSE	2	0.73113912	0.55629718	2.0892961	0.349945188	1.527565956	0.66680682	0.4745751	2.10862804	0.319153786	1.406048059	0.76559663	0.6144529	2.10862804	0.366437614	1.614359021
608	4.03	4.09	15.1700005	2.69499999	2.22600009	sp|P08122|C	Collagen alpha-2(IV) chain OS=Mus musculus GN=Col4a2 PE=2 SV=4	MOUSE	3	2.31206489	0.60053688	2.07014108	1.116863012	6.025596142	2.55858588	0.51894248	2.12813902	1.202263951	6.79203701	6.13761997	0.22601271	2.33345795	2.630268097	16.59586906
609	4.03	4.06	43.2999998	36.0799998	17.5300002	sp|P56375|A	Acylphosphatase-2 OS=Mus musculus GN=Acyp2 PE=2 SV=1	MOUSE	2	1.10662401	0.47320041	1.61435902	0.685488224	1.786488056	0.88715601	0.75899792	1.97696996	0.432513803	1.753880978	0.95499259	0.97589928	1.78648806	0.534564376	1.706081986
610	4.03	4.03	33.3299995	12.3199999	12.3199999	sp|Q9CR98|F	Protein FAM136A OS=Mus musculus GN=Fam136a PE=1 SV=1	MOUSE	3	1.35518897	0.54280537	1.51356101	0.895364821	6.486343861	2.51188588	0.1077233	2.26986504	1.106624007	9.289664268	1.31825697	0.95166642	2.24905491	0.586138189	4.325138092
611	4.02	4.88	16.9100002	2.67399997	2.67399997	sp|Q8CHD8|R	Rab11 family-interacting protein 3 OS=Mus musculus GN=Rab11fip3 PE=1 SV=2	MOUSE	3	0.90364939	0.92436832	1.36772895	0.654636085	1.235947013	0.8472274	0.96421433	1.28233099	0.654636085	1.08642602	0.39445731	0.46278599	1.73780096	0.2108628	0.685488224
612	4.02	4.23	24.2899999	5.20499982	4.4160001	sp|Q9JHS4|C	ATP-dependent Clp protease ATP-binding subunit clpX-like, mitochondrial OS=Mus musculus GN=Clpx PE=1 S	MOUSE	2	1.13762701	0.61822063	1.247383	0.912010789	1.419057012	0.89536482	0.53998089	1.61435902	0.55462569	1.445440054	0.87902248	0.2183803	1.34276497	0.654636085	1.180320978
613	4.02	4.13	22.7500007	3.04300003	2.46399995	sp|O70133|D	ATP-dependent RNA helicase A OS=Mus musculus GN=Dhx9 PE=1 SV=2	MOUSE	2	0.31622779	0.25132191	2.55858588	0.06025596	0.809095919	0.68548822	0.57841939	2.53512907	0.263026804	1.737800956	1.08642602	0.84993309	1.70608199	0.636795521	1.870682001
614	4.02	4.08	21.9300002	3.74299996	3.74299996	sp|Q91V88|N	Nephronectin OS=Mus musculus GN=Npnt PE=1 SV=1	MOUSE	2	1.06659603	0.87838632	6.66806698	0.1599558	7.112134933	0.58613819	0.39585891	23.1206493	0.03872576	13.55189037	0.82413811	0.7144413	20.3235703	0.046989411	16.7494297
615	4.02	4.07	15.6499997	3.03499997	2.55600009	sp|Q922B9|S	Sperm-specific antigen 2 homolog OS=Mus musculus GN=Ssfa2 PE=1 SV=3	MOUSE	2	0.86297852	0.7355876	5.345644	0.1599558	4.613175869	0.91201079	0.87276089	2.10862804	0.436515808	1.923092008	0.87902248	0.75698268	6.1944108	0.140604705	5.445025921
616	4.02	4.06	26.3399988	9.82099995	6.69599995	sp|Q9DC70|N	NADH dehydrogenase [ubiquinone] iron-sulfur protein 7, mitochondrial OS=Mus musculus GN=Ndufs7 PE=1 S	MOUSE	2	0.69183099	0.50551403	7.11213493	0.097274721	4.920394897	0.73790431	0.56996661	2.0892961	0.349945188	1.541700006	0.74473202	0.60604233	2.0892961	0.35318321	1.55596602
617	4.02	4.05	26.4400005	7.15700015	7.15700015	sp|Q8C165|P	Probable carboxypeptidase PM20D1 OS=Mus musculus GN=Pm20d1 PE=2 SV=1	MOUSE	4															
618	4.02	4.04	23.1000006	6.13700002	6.13700002	sp|P48758|C	Carbonyl reductase [NADPH] 1 OS=Mus musculus GN=Cbr1 PE=1 SV=3	MOUSE	2	0.83176369	0.67668092	1.34276497	0.619441092	1.116863012	1.09647799	0.9913649	1.36772895	0.801678121	1.499685049	1.29419601	0.67650378	1.52756596	0.847227395	1.976969957
619	4.02	4.03	15.4100001	3.85399982	3.85399982	sp|Q9CWK8|S	Sorting nexin-2 OS=Mus musculus GN=Snx2 PE=1 SV=2	MOUSE	2	2.20800495	0.53700662	3.73250198	0.591561615	14.72313023	2.51188588	0.495056	4.36515904	0.57543987	16.14359093	2.22843504	0.74110729	4.7424202	0.469894111	13.06171036
620	4.02	4.02	51.3000011	25.3199995	25.3199995	sp|Q9DCB8|I	Iron-sulfur cluster assembly 2 homolog, mitochondrial OS=Mus musculus GN=Isca2 PE=2 SV=2	MOUSE	4	1.05681801	0.98127258	2.0892961	0.505824685	2.208004951	0.87096357	0.89248812	1.33045399	0.642687678	1.158776999	0.57543987	0.35420531	1.75388098	0.296483099	1.009253025
621	4.02	4.02	56.7700028	28.3899993	22.5799993	sp|Q8CBY0|G	Glutamyl-tRNA(Gln) amidotransferase subunit C, mitochondrial OS=Mus musculus GN=Gatc PE=2 SV=1	MOUSE	2	1.51356101	0.57677108	1.36772895	1.106624007	2.128139019	1.41905701	0.59421188	1.64437199	0.862978518	2.376840115	0.4875285	0.64311701	2.51188588	0.197696999	1.224616051
622	4.01	4.14	30.0900012	14.35	9.25899968	sp|P24369|P	Peptidyl-prolyl cis-trans isomerase B OS=Mus musculus GN=Ppib PE=2 SV=2	MOUSE	2	0.85506672	0.27258971	3.07609701	0.277971298	2.630268097	0.89536482	0.3105444	5.64937019	0.158489302	5.058247089	1.09647799	0.54004937	2.0892961	0.524807513	2.290868044
623	4.01	4.12	24.47	6.31600022	6.31600022	sp|Q7TNV0|D	Protein DEK OS=Mus musculus GN=Dek PE=1 SV=1	MOUSE	2	1.35518897	0.58268112	2.0892961	0.648634374	2.85758996	1.09647799	0.81965369	3.1045599	0.35318321	3.435580015	0.96382898	0.95677108	2.0892961	0.461317599	2.013724089
624	4.01	4.04	27.1200001	11.53	11.53	sp|Q9DB05|S	Alpha-soluble NSF attachment protein OS=Mus musculus GN=Napa PE=1 SV=1	MOUSE	4	0.44463131	0.27727211	2.24905491	0.185353205	1	0.51522863	0.33639401	1.599558	0.310455889	0.824138105	0.2355049	0.26944271	2.37684012	0.1018591	0.55975759
625	4.01	4.04	31.220001	16.0999999	16.0999999	sp|P62821|R	Ras-related protein Rab-1A OS=Mus musculus GN=Rab1A PE=1 SV=3	MOUSE	2	0.99083197	0.93227798	2.0892961	0.474242002	2.070141077	0.71121353	0.4921813	3.76703811	0.190546095	2.679167986	0.96382898	0.93935323	2.39883304	0.401790798	2.312064886
626	4.01	4.04	48.15	17.9000005	17.9000005	sp|Q9ERR7|S	15 kDa selenoprotein OS=Mus musculus GN=Sep15 PE=1 SV=3	MOUSE	2	1.14815402	0.78290808	2.01372409	0.570164323	2.312064886	1.07646501	0.88601899	1.51356101	0.711213529	1.629295945	0.83946002	0.73164171	1.247383	0.672976673	1.047129035
627	4.01	4.03	12.4600001	4.54499982	4.54499982	sp|Q69ZS7|H	HBS1-like protein OS=Mus musculus GN=Hbs1l PE=1 SV=2	MOUSE	2															
628	4.01	4.01	8.89699981	2.88200006	2.88200006	sp|P09055|IT	Integrin beta-1 OS=Mus musculus GN=Itgb1 PE=1 SV=1	MOUSE	2	1.13762701	0.81655192	1.23594701	0.920449615	1.445440054	0.99083197	0.96023119	1.78648806	0.524807513	1.770109057	2.03235698	0.44653159	1.52756596	1.330453992	3.564511061
629	4.01	4.01	39.35	23.15	15.2799994	sp|P35278|R	Ras-related protein Rab-5C OS=Mus musculus GN=Rab5c PE=1 SV=2	MOUSE	2															
630	4.01	4.01	31.9700003	17.6899999	17.6899999	sp|P04444|H	Hemoglobin subunit beta-H1 OS=Mus musculus GN=Hbb-bh1 PE=2 SV=3	MOUSE	2															
631	4	11.92	48.7500012	22.8100002	18.75	sp|P49312|R	Heterogeneous nuclear ribonucleoprotein A1 OS=Mus musculus GN=Hnrnpa1 PE=1 SV=2	MOUSE	8	0.41304749	0.36964899	1.870682	0.218776196	0.772680581	0.45289761	0.35131979	1.41905701	0.307609707	0.642687678	0.51050502	0.37796921	1.45881403	0.343558013	0.744732022
632	4	11.7	46.509999	40.1199997	40.1199997	sp|Q9CQ19|M	Myosin regulatory light polypeptide 9 OS=Mus musculus GN=Myl9 PE=1 SV=3	MOUSE	8	0.76559663	0.59895343	1.57036305	0.492039502	1.202263951	0.75162292	0.55784518	1.29419601	0.586138189	0.972747207	1.01859105	0.94779062	1.43218803	0.711213529	1.458814025
633	4	4.1	10.9700002	1.27100004	1.27100004	sp|Q61838|A	Alpha-2-macroglobulin OS=Mus musculus GN=A2m PE=1 SV=3	MOUSE	2	1.34276497	0.82454503	1.33045399	1.009253025	1.786488056	1.45881403	0.53621221	1.158777	1.258924961	1.721868992	2.53512907	0.2395743	1.20226395	2.108628035	3.133285999
634	4	4.07	17.7200005	4.92100008	4.92100008	sp|Q8C0C7|S	Phenylalanyl-tRNA synthetase alpha chain OS=Mus musculus GN=Farsa PE=2 SV=1	MOUSE	4	0.57016432	0.3945902	2.10862804	0.27289781	1.202263951	1.02801597	0.94516099	2.0892961	0.492039502	2.147830009	0.73113912	0.58309889	2.0892961	0.349945188	1.527565956
635	4	4.06	33.5200012	15.3400004	15.3400004	sp|Q91WV0|N	Protein Dr1 OS=Mus musculus GN=Dr1 PE=2 SV=1	MOUSE	2	1.49968505	0.51493299	2.0892961	0.717794299	3.162277937	0.98174793	0.98360658	3.34194994	0.293765008	3.28095293	1.40604806	0.57783622	4.40554905	0.319153786	6.194410801
636	4	4.05	35.4699999	7.46700019	6.13300018	sp|P70460|V	Vasodilator-stimulated phosphoprotein OS=Mus musculus GN=Vasp PE=1 SV=4	MOUSE	2															
637	4	4.05	17.8100005	5.57599999	5.57599999	sp|P11983|T	T-complex protein 1 subunit alpha OS=Mus musculus GN=Tcp1 PE=1 SV=3	MOUSE	2	0.85506672	0.77731562	2.10862804	0.40926069	1.803017974	1.39315701	0.54304749	2.0892961	0.666806817	2.91071701	1.11686301	0.8070485	2.0892961	0.534564376	2.333457947
638	4	4.05	14.4199997	9.38199982	5.95000014	sp|Q9D8N0|E	Elongation factor 1-gamma OS=Mus musculus GN=Eef1g PE=1 SV=3	MOUSE	2	0.82413811	0.70929903	14.5881395	0.061376199	12.02264023	0.54954088	0.40302131	23.5504894	0.037325021	12.94196033	0.66680682	0.51525348	2.96483111	0.226986498	1.976969957
639	4	4.05	16.4399996	9.31499973	9.31499973	sp|Q9D5V6|S	Synapse-associated protein 1 OS=Mus musculus GN=Syap1 PE=1 SV=1	MOUSE	5	0.87096357	0.89298648	6.2517271	0.139315695	5.445025921	1.20226395	0.70875228	17.5388107	0.068548821	18.19701004	1.11686301	0.83255589	2.10862804	0.529663384	2.333457947
640	4	4.04	15.2500004	7.07800016	7.07800016	sp|Q6NVF9|C	Cleavage and polyadenylation specificity factor subunit 6 OS=Mus musculus GN=Cpsf6 PE=1 SV=1	MOUSE	3	1.33045399	0.61411041	1.38038397	0.963828981	1.836537957	1.12719798	0.75382477	1.45881403	0.772680581	1.659587026	1.06659603	0.8254087	1.47231197	0.724435985	1.584892988
641	4	4.04	17.8900003	8.94600004	8.94600004	sp|Q3TC72|F	Fumarylacetoacetate hydrolase domain-containing protein 2A OS=Mus musculus GN=Fahd2 PE=1 SV=1	MOUSE	4	1.23594701	0.5785768	1.30617094	0.946237087	1.614359021	1.07646501	0.91779357	1.23594701	0.870963573	1.330453992	1.03752804	0.89273733	1.21338904	0.855066717	1.258924961
642	4	4.04	26.76	19.72	19.72	sp|Q9CQI3|G	Glia maturation factor beta OS=Mus musculus GN=Gmfb PE=1 SV=3	MOUSE	2	0.71121353	0.6113475	1.23594701	0.549540877	0.879022479	0.3311311	0.30028829	2.20800495	0.1127197	0.731139123	0.4168694	0.14353541	1.69044101	0.194088593	0.704693079
643	4	4.03	29.2100012	9.20599997	9.20599997	sp|Q6ZWX6|I	Eukaryotic translation initiation factor 2 subunit 1 OS=Mus musculus GN=Eif2s1 PE=1 SV=3	MOUSE	3	0.92044962	0.91474938	1.48593605	0.619441092	1.367728949	0.97274721	0.9496333	1.27057397	0.758577585	1.235947013	1.40604806	0.55634332	1.23594701	1.137627006	1.753880978
644	4	4.03	13.82	6.28399998	6.28399998	sp|Q61207|S	Sulfated glycoprotein 1 OS=Mus musculus GN=Psap PE=1 SV=2	MOUSE	2	1.11686301	0.81097919	6.13761997	0.181970105	6.854881763	1.29419601	0.62387699	4.13047504	0.313328594	5.345643997	1.22461605	0.68199748	6.02559614	0.203235701	7.447319984
645	4	4.03	43.7999994	20.4400003	16.0600007	sp|Q9D8S9|B	BolA-like protein 1 OS=Mus musculus GN=Bola1 PE=1 SV=1	MOUSE	2	1.25892496	0.41249991	1.39315701	0.90364939	1.737800956	1.55596602	0.30052179	2.33345795	0.666806817	3.732501984	0.87902248	0.50423282	1.48593605	0.580764413	1.30617094
646	4	4.03	23.6399993	8.78800005	8.78800005	sp|P19973|L	Lymphocyte-specific protein 1 OS=Mus musculus GN=Lsp1 PE=1 SV=2	MOUSE	2	1.58489299	0.45203051	22.6986504	0.069823243	24.43431091	2.39883304	0.274874	13.67729	0.175388098	24.43431091	1.21338904	0.70555401	12.1338902	0.100000001	14.32188034
647	4	4.02	41.5499985	8.58699977	8.58699977	sp|Q3UM45|P	Protein phosphatase 1 regulatory subunit 7 OS=Mus musculus GN=Ppp1r7 PE=1 SV=2	MOUSE	2	0.85506672	0.77520323	2.0892961	0.40926069	1.786488056	1.22461605	0.68157059	2.0892961	0.586138189	2.558585882	0.89536482	0.83572233	2.0892961	0.428548515	1.870682001
648	4	4.02	20.5899999	5.88200018	5.88200018	sp|P50136|O	2-oxoisovalerate dehydrogenase subunit alpha, mitochondrial OS=Mus musculus GN=Bckdha PE=1 SV=1	MOUSE	2	0.77268058	0.63098693	20.7014103	0.045289759	15.99557972	1.01859105	0.94818068	19.0546093	0.053456441	18.87990952	1.09647799	0.83334261	8.0909586	0.135518894	8.953647614
649	4	4.02	23.4899998	5.42199984	5.42199984	sp|O35685|N	Nuclear migration protein nudC OS=Mus musculus GN=Nudc PE=1 SV=1	MOUSE	2	1.14815402	0.83592451	1.61435902	0.711213529	1.853531957	1.36772895	0.43616211	1.39315701	0.981747925	1.887990952	1.39315701	0.4617537	1.80301797	0.772680581	2.558585882
650	4	4.02	15.0800005	7.03499988	7.03499988	sp|Q8VCN5|C	Cystathionine gamma-lyase OS=Mus musculus GN=Cth PE=1 SV=1	MOUSE	3	0.59156162	0.42753431	4.48745394	0.124738403	2.654606104	0.83176369	0.74630308	13.8038397	0.063679554	11.48153973	0.42854852	0.29981619	20.3235703	0.03499452	8.709635735
651	4	4.02	18.3500007	9.17399973	9.17399973	sp|O70400|P	PDZ and LIM domain protein 1 OS=Mus musculus GN=Pdlim1 PE=2 SV=4	MOUSE	2	1.21338904	0.67097032	11.3762703	0.106659599	13.55189037	1.44544005	0.53048569	2.10862804	0.685488224	3.019952059	1.65958703	0.3946766	20.5116196	0.080909587	22.90867996
652	4	4	25.8300006	8.94000009	8.94000009	sp|Q8CCS6|P	Polyadenylate-binding protein 2 OS=Mus musculus GN=Pabpn1 PE=2 SV=3	MOUSE	2	0.70469308	0.54931849	2.99226499	0.235504895	2.108628035	0.80909592	0.74007517	3.28095293	0.246603906	2.654606104	0.82413811	0.6731205	2.0892961	0.39445731	1.721868992
653	4	4	21.5299994	5.78700006	5.78700006	sp|P50247|S	Adenosylhomocysteinase OS=Mus musculus GN=Ahcy PE=1 SV=3	MOUSE	3	0.48305881	0.47857171	1.57036305	0.310455889	0.758577585	0.4655861	0.41766	1.55596602	0.296483099	0.724435985	0.83176369	0.69386393	1.19124198	0.691830993	0.990831971
654	4	4	30.9199989	14.1200006	14.1200006	sp|Q9D6S7|R	Ribosome-recycling factor, mitochondrial OS=Mus musculus GN=Mrrf PE=1 SV=1	MOUSE	3	0.91201079	0.89803499	2.72897792	0.334194988	2.488857031	1.158777	0.75175643	1.247383	0.928966403	1.458814025	0.77268058	0.68531787	1.78648806	0.432513803	1.380383968
655	4	4	16.6199997	5.71400002	5.71400002	sp|Q8QZS1|H	3-hydroxyisobutyryl-CoA hydrolase, mitochondrial OS=Mus musculus GN=Hibch PE=1 SV=1	MOUSE	2	0.46989411	0.30238161	4.48745394	0.105681702	2.108628035	0.3767038	0.1961187	1.41905701	0.251188606	0.534564376	0.57016432	0.37683481	2.83139205	0.197696999	1.614359021
656	4	4	34.1100007	22.4800006	22.4800006	sp|Q9CR41|H	Huntingtin-interacting protein K OS=Mus musculus GN=Hypk PE=2 SV=2	MOUSE	3	0.93756199	0.92668319	2.20800495	0.424619585	2.070141077	1.08642602	0.81870472	1.65958703	0.654636085	1.803017974	0.97274721	0.98114151	1.599558	0.608134985	1.55596602
657	4	4	11.7399998	3.627	3.627	sp|Q80WJ7|L	Protein LYRIC OS=Mus musculus GN=Mtdh PE=1 SV=1	MOUSE	2	1.20226395	0.69463033	2.18776202	0.549540877	2.654606104	1.28233099	0.66955072	2.0892961	0.613762021	2.703958035	1.51356101	0.64962232	6.36795521	0.237683997	10.28015995
658	4	4	12.6699999	8.98599997	8.98599997	sp|P59017|B	Bcl-2-like protein 13 OS=Mus musculus GN=Bcl2l13 PE=1 SV=2	MOUSE	2	1.01859105	0.76211667	1.80301797	0.564936996	1.836537957	0.87902248	0.79001677	1.75388098	0.501187205	1.541700006	0.79432821	0.355382	1.57036305	0.505824685	1.247382998
659	4	4	39.8099989	20.3700006	20.3700006	sp|Q91WS0|C	CDGSH iron-sulfur domain-containing protein 1 OS=Mus musculus GN=Cisd1 PE=1 SV=1	MOUSE	2	0.97274721	0.9506259	2.051162	0.474242002	1.995262027	0.99083197	0.97412938	1.38038397	0.717794299	1.367728949	0.9289664	0.95608902	1.67494297	0.549540877	1.55596602
660	4	4	47.5699991	22.3299995	22.3299995	sp|P63024|V	Vesicle-associated membrane protein 3 OS=Mus musculus GN=Vamp3 PE=1 SV=1	MOUSE	2	0.81658238	0.92169732	20.3235703	0.046989411	16.59586906	0.4017908	0.40774381	26.7916794	0.031332862	10.76465034	0.89536482	0.92352271	19.4088593	0.04965923	17.3780098
660	0.01	2.01	14.2199993	1.68699995	1.68699995	RRRRRsp|Q9	REVERSED Casein kinase I isoform delta OS=Mus musculus GN=Csnk1d PE=1 SV=2	MOUSE	1															
660	0.01	2.01	5.52899987	1.68299992	1.68299992	RRRRRsp|Q9	REVERSED Casein kinase I isoform epsilon OS=Mus musculus GN=Csnk1e PE=1 SV=2	MOUSE	1															
660	0	2.01	5.43499999	1.26799997	1.26799997	RRRRRsp|Q9	REVERSED Autoimmune regulator OS=Mus musculus GN=Aire PE=1 SV=1	MOUSE	1															
660	0	2	10.1700001	5.93199991	5.93199991	sp|Q62442|V	Vesicle-associated membrane protein 1 OS=Mus musculus GN=Vamp1 PE=1 SV=1	MOUSE	1															
661	4	4	38.1000012	28.5699993	28.5699993	sp|O35127|C	Protein C10 OS=Mus musculus GN=Grcc10 PE=2 SV=1	MOUSE	4															
662	4	4	28.9700002	21.5000004	21.5000004	sp|P17095|H	High mobility group protein HMG-I/HMG-Y OS=Mus musculus GN=Hmga1 PE=1 SV=4	MOUSE	2	2.55858588	0.3908484	1.99526203	1.28233099	28.57589912	3.133286	0.3352187	2.72897792	1.14815402	30.4789505	2.93764997	0.32501441	1.65958703	1.770109057	38.37071991
663	4	4	3.29400003	3.29400003	3.29400003	sp|Q60972|R	Histone-binding protein RBBP4 OS=Mus musculus GN=Rbbp4 PE=1 SV=5	MOUSE	3	4.32513809	0.72705638	4.4463129	0.972747207	19.23092079	6.2517271	0.49031901	4.48745394	1.393157005	27.79713058	6.98232412	0.44578371	4.48745394	1.55596602	30.76096916
664	4	4	15.1199997	15.1199997	15.1199997	sp|P62307|R	Small nuclear ribonucleoprotein F OS=Mus musculus GN=Snrpf PE=2 SV=1	MOUSE	2	0.18197011	0.1428663	2.20800495	0.070469297	0.401790798	0.38725761	0.28347161	4.28548479	0.090364948	1.659587026	0.30199519	0.1706598	3.872576	0.075857759	1.16949904
665	3.97	14.3	34.1399998	15.3200001	15.3200001	sp|Q9QZQ8|H	Core histone macro-H2A.1 OS=Mus musculus GN=H2afy PE=1 SV=3	MOUSE	10	0.65463609	0.31247139	1.61435902	0.398107201	1.056818008	0.69183099	0.3720575	1.599558	0.428548515	1.106624007	0.83946002	0.54511231	1.38038397	0.608134985	1.158776999
666	3.96	4.07	15.8299997	5.27499989	5.27499989	sp|Q8VCM7|F	Fibrinogen gamma chain OS=Mus musculus GN=Fgg PE=2 SV=1	MOUSE	2	0.09204496	0.57371891	6.02559614	0.034355789	0.55462569	1.95884502	0.60671938	2.37684012	0.824138105	7.870458126	5.29663515	0.2465619	2.88403201	1.836537957	27.79713058
667	3.94	4.28	28.61	5.07199988	4.16099988	sp|Q8BUV3|G	Gephyrin OS=Mus musculus GN=Gphn PE=1 SV=2	MOUSE	3	0.83176369	0.69057381	1.51356101	0.534564376	1.258924961	0.99083197	0.71702182	1.62929595	0.608134985	1.614359021	1.01859105	0.79186422	1.43218803	0.711213529	1.472311974
668	3.92	3.94	48.8299996	12.8900006	12.8900006	sp|Q9CQQ7|A	ATP synthase subunit b, mitochondrial OS=Mus musculus GN=Atp5f1 PE=1 SV=1	MOUSE	3	0.77268058	0.27035689	1.18032098	0.654636085	0.912010789	0.69823241	0.2443628	1.247383	0.55462569	0.870963573	0.61376202	0.1380447	1.247383	0.469894111	0.765596628
669	3.9	4.07	15.9899995	0.9079	0.9079	sp|P19137|L	Laminin subunit alpha-1 OS=Mus musculus GN=Lama1 PE=1 SV=1	MOUSE	2	10.6659603	0.1554248	5.15228701	2.070141077	47.42419815	8.95364761	0.21288881	5.49540901	1.629295945	42.85485077	10.1859102	0.112416	5.01187182	2.032356977	46.9894104
670	3.89	6.86	56.4700007	29.8000008	23.5300005	sp|P62259|14	14-3-3 protein epsilon OS=Mus musculus GN=Ywhae PE=1 SV=1	MOUSE	8	1.31825697	0.29912561	1.55596602	0.847227395	2.089296103	1	0.97507429	1.25892496	0.794328213	1.270573974	0.55975759	0.4933129	1.80301797	0.319153786	1.009253025
671	3.89	3.89	25.3500015	7.3239997	5.63400015	sp|Q99LC3|N	NADH dehydrogenase [ubiquinone] 1 alpha subcomplex subunit 10, mitochondrial OS=Mus musculus GN=Ndu	MOUSE	3	0.44055489	0.1068303	2.26986504	0.1458814	1	0.2355049	0.04101081	4.6558609	0.03404082	1.096477985	0.48305881	0.1301728	3.46736908	0.125892505	1.67494297
672	3.87	3.9	31.2400013	8.50299969	8.50299969	sp|P80316|T	T-complex protein 1 subunit epsilon OS=Mus musculus GN=Cct5 PE=1 SV=1	MOUSE	3	1.10662401	0.91856837	1.247383	0.88715601	1.393157005	0.95499259	0.86907297	2.53512907	0.334194988	2.421029091	2.37684012	0.45702189	2.01372409	1.180320978	5.546257019
673	3.86	6.77	17.7499995	0.86909998	0.86909998	sp|Q70FJ1|A	A-kinase anchor protein 9 OS=Mus musculus GN=Akap9 PE=2 SV=2	MOUSE	3	1.09647799	0.83494192	2.0892961	0.524807513	2.290868044	0.74473202	0.60041022	2.0892961	0.356451094	1.55596602	1.00925303	0.97351319	2.0892961	0.48305881	2.108628035
674	3.85	52.18	88.8000011	84.799999	76.8000007	sp|P63260|A	Actin, cytoplasmic 2 OS=Mus musculus GN=Actg1 PE=1 SV=1	MOUSE	82															
675	3.83	3.83	17.3800007	5.79399988	5.79399988	sp|P70290|E	55 kDa erythrocyte membrane protein OS=Mus musculus GN=Mpp1 PE=1 SV=1	MOUSE	2	1.07646501	0.87385368	3.94457293	0.27289781	4.207265854	0.85506672	0.77066672	15.55966	0.06025596	13.30453968	0.71121353	0.55804843	2.77971292	0.2558586	1.976969957
676	3.8	4.56	24.1899997	5.45900017	3.72200012	sp|Q9Z315|S	U4/U6.U5 tri-snRNP-associated protein 1 OS=Mus musculus GN=Sart1 PE=2 SV=1	MOUSE	2	1	0.96195161	1.38038397	0.724435985	1.380383968	1.18032098	0.2661086	1.38038397	0.855066717	1.629295945	1.158777	0.31455341	1.43218803	0.809095919	1.659587026
677	3.8	3.87	34.5699996	24.6900007	14.8100004	sp|O70492|S	Sorting nexin-3 OS=Mus musculus GN=Snx3 PE=1 SV=3	MOUSE	3	0.34673679	0.43556401	1.73780096	0.203235701	0.602559626	0.59156162	0.65246701	1.58489299	0.366437614	0.937561989	0.49659231	0.4942506	1.62929595	0.296483099	0.809095919
678	3.8	3.8	18.7700003	11.1100003	11.1100003	sp|Q91XF0|P	Pyridoxine-5'-phosphate oxidase OS=Mus musculus GN=Pnpo PE=1 SV=1	MOUSE	2	0.89536482	0.57546341	1.70608199	0.519995987	1.527565956	0.80167812	0.48766911	1.90546095	0.413047493	1.527565956	0.83946002	0.54808962	1.49968505	0.55975759	1.258924961
679	3.76	4.32	23.0299994	10.1999998	8.22400004	sp|Q64010|C	Adapter molecule crk OS=Mus musculus GN=Crk PE=1 SV=1	MOUSE	2	0.82413811	0.71584958	2.0892961	0.39445731	1.721868992	0.88715601	0.82604629	2.0892961	0.424619585	1.853531957	0.564937	0.3891246	2.10862804	0.270395786	1.19124198
680	3.76	3.76	26.0199994	26.0199994	21.1400002	sp|Q9CQM5|T	Thioredoxin domain-containing protein 17 OS=Mus musculus GN=Txndc17 PE=1 SV=1	MOUSE	5	0.7798301	0.74143952	1.38038397	0.55462569	1.076465011	0.8472274	0.70265842	1.12719798	0.744732022	0.954992592	0.474242	0.44376031	1.72186899	0.27289781	0.816582382

681	3.74	3.94	20.7300007	7.51700029	5.23900017	sp|P29758|O	Ornithine aminotransferase, mitochondrial OS=Mus musculus GN=Oat PE=1 SV=1	MOUSE	2	1.13762701	0.84343022	4.48745394	0.253512889	5.296635151	0.96382898	0.70526439	4.69894123	0.214782998	4.528975964	1.23594701	0.94688898	4.48745394	0.275422901	5.970353127
682	3.73	3.73	16.8300003	16.8300003	7.30199963	sp|Q91V76|C	Ester hydrolase C11orf54 homolog OS=Mus musculus PE=2 SV=1	MOUSE	3	0.78704578	0.52779162	1.67494297	0.465586096	1.318256974	0.55975759	0.1473317	2.93764997	0.188799098	1.644371986	0.85506672	0.66174579	1.870682	0.461317599	1.599557996
683	3.71	3.71	61.5400016	25.6399989	22.5600004	sp|P61022|C	Calcium-binding protein p22 OS=Mus musculus GN=Chp PE=2 SV=2	MOUSE	3	0.92044962	0.58791262	1.25892496	0.731139123	1.158776999	0.64863437	0.16477969	1.54170001	0.420726597	1	0.80167812	0.3161009	1.27057397	0.630957425	1.018591046
684	3.69	3.71	31.25	13.3200005	13.3200005	sp|Q62419|S	Endophilin-A2 OS=Mus musculus GN=Sh3gl1 PE=1 SV=1	MOUSE	2	0.95499259	0.77842277	1.870682	0.510505021	1.786488056	0.99083197	0.89737141	1.247383	0.794328213	1.235947013	0.8472274	0.47757661	1.94088602	0.436515808	1.644371986
685	3.68	3.69	37.2399986	21.3799998	21.3799998	sp|Q9CZX8|F	40S ribosomal protein S19 OS=Mus musculus GN=Rps19 PE=1 SV=3	MOUSE	4	1.11686301	0.9903903	1.36772895	0.816582382	1.527565956	0.94623709	0.58279598	1.45881403	0.648634374	1.380383968	1.02801597	0.99990392	1.14815402	0.895364821	1.180320978
686	3.67	3.67	15.7700002	8.29899982	8.29899982	sp|Q9Z2U1|P	Proteasome subunit alpha type-5 OS=Mus musculus GN=Psma5 PE=1 SV=1	MOUSE	3	1.06659603	0.49891439	1.48593605	0.717794299	1.570363045	1.16949904	0.21090061	1.83653796	0.636795521	2.147830009	1.247383	0.04167401	2.14783001	0.580764413	2.679167986
687	3.66	3.66	17.8000003	3.95499989	3.95499989	sp|Q08943|S	FACT complex subunit SSRP1 OS=Mus musculus GN=Ssrp1 PE=1 SV=2	MOUSE	2															
688	3.65	3.76	43.2599992	12.0899998	12.0899998	sp|P51174|A	Long-chain specific acyl-CoA dehydrogenase, mitochondrial OS=Mus musculus GN=Acadl PE=2 SV=2	MOUSE	7	0.49659231	0.25154162	1.43218803	0.337287307	0.711213529	0.44463131	0.1172381	1.44544005	0.299226493	0.642687678	0.4325138	0.35975549	1.58489299	0.267916799	0.685488224
689	3.64	3.7	22.8	9.41400006	8.15899968	sp|Q61655|D	ATP-dependent RNA helicase DDX19A OS=Mus musculus GN=Ddx19a PE=2 SV=2	MOUSE	3	0.67920363	0.50323927	8.39459992	0.080909587	5.70164299	0.66069353	0.47159681	22.0800495	0.041304749	14.58813953	0.55462569	0.3655614	23.5504894	0.037670381	13.06171036
690	3.64	3.64	84.0600014	30.430001	30.430001	sp|P62858|R	40S ribosomal protein S28 OS=Mus musculus GN=Rps28 PE=2 SV=1	MOUSE	6	1.70608199	0.74404073	1.54170001	1.106624007	3.019952059	2.37684012	0.59831888	1.67494297	1.419057012	5.058247089	2.42102909	0.59491259	1.70608199	1.419057012	4.965922832
691	3.64	3.64	17.5699994	8.30700025	8.30700025	sp|O54983|C	Thiomorpholine-carboxylate dehydrogenase OS=Mus musculus GN=Crym PE=1 SV=1	MOUSE	2	0.83946002	0.30834311	1.44544005	0.580764413	1.213389039	0.90364939	0.66591609	1.30617094	0.691830993	1.180320978	0.82413811	0.42678401	1.52756596	0.534564376	1.258924961
692	3.62	3.62	14.61	7.79199973	7.79199973	sp|Q9Z0S1|B	3'(2'),5'-bisphosphate nucleotidase 1 OS=Mus musculus GN=Bpnt1 PE=1 SV=2	MOUSE	2	0.51050502	0.33807451	2.0892961	0.242102906	1.066596031	0.66069353	0.48936611	2.10862804	0.316227794	1.393157005	0.64863437	0.47808459	2.10862804	0.310455889	1.367728949
693	3.61	3.62	31.279999	18.4699997	12.5599995	sp|Q9JL35|H	High mobility group nucleosome-binding domain-containing protein 5 OS=Mus musculus GN=Hmgn5 PE=1 SV	MOUSE	3	6.54636192	0.100987	4.57088184	1.432188034	29.1071701	6.13761997	0.1036147	4.61317587	1.330453992	27.54228973	5.24807501	0.03639615	4.61317587	1.137627006	23.76840019
694	3.6	3.6	36.1200005	17.6100001	12.2400001	sp|Q9CQ62|D	2,4-dienoyl-CoA reductase, mitochondrial OS=Mus musculus GN=Decr1 PE=1 SV=1	MOUSE	4	0.92044962	0.26893899	1.57036305	0.564936996	1.445440054	0.90364939	0.2901746	1.58489299	0.570164323	1.432188034	1.07646501	0.4384613	1.57036305	0.685488224	1.67494297
695	3.59	3.64	45.7599998	31.3600004	21.1899996	sp|P62774|M	Myotrophin OS=Mus musculus GN=Mtpn PE=1 SV=2	MOUSE	3	1.18032098	0.65236718	6.72976589	0.175388098	7.943282127	1.28233099	0.54176873	12.2461596	0.104712903	15.55965996	1.23594701	0.59163332	11.4815397	0.107646503	14.19058037
696	3.56	3.6	21.5599999	5.04599996	5.04599996	sp|P55131|S	Septin-7 OS=Mus musculus GN=Sept7 PE=1 SV=1	MOUSE	3	1.27057397	0.16416889	1.51356101	0.839460015	1.940886021	1.51356101	0.1097819	1.62929595	0.928966403	2.511885881	1.94088602	0.06614397	2.0892961	0.928966403	4.092607021
697	3.54	3.73	32.6400012	12.4600001	12.4600001	sp|Q9CQM9|G	Glutaredoxin-3 OS=Mus musculus GN=Glrx3 PE=1 SV=1	MOUSE	2	0.4655861	0.30596459	2.10862804	0.222843498	0.981747925	0.73790431	0.58444512	2.0892961	0.349945188	1.541700006	0.63095743	0.45496231	2.0892961	0.301995188	1.318256974
698	3.53	3.69	24.8899996	10.5499998	8.43899995	sp|Q9JIQ3|D	Diablo homolog, mitochondrial OS=Mus musculus GN=Diablo PE=1 SV=1	MOUSE	3	0.85506672	0.42410651	2.72897792	0.313328594	2.333457947	0.94623709	0.59820497	2.70395804	0.346736789	2.558585882	0.83946002	0.56865382	2.58225989	0.325087309	2.167704105
699	3.5	3.59	30.1800013	9.66499969	9.66499969	sp|Q5SRX1|T	TOM1-like protein 2 OS=Mus musculus GN=Tom1l2 PE=1 SV=1	MOUSE	2	0.63679552	0.44294119	22.2843494	0.04055085	14.19058037	1.09647799	0.83777392	15.55966	0.070469297	15.84893036	0.90364939	0.84164029	3.1915381	0.280543387	2.884032011
700	3.5	3.5	42.2800005	13.9699996	13.9699996	sp|O88696|C	Putative ATP-dependent Clp protease proteolytic subunit, mitochondrial OS=Mus musculus GN=Clpp PE=2 SV	MOUSE	3	0.93756199	0.91588408	1.78648806	0.515228629	1.67494297	0.30478951	0.4416863	2.58225989	0.075162292	0.787045777	0.83176369	0.81659782	1.40604806	0.57543987	1.16949904
701	3.5	3.5	21.1099997	5.53600006	5.53600006	sp|Q8CDN6|T	Thioredoxin-like protein 1 OS=Mus musculus GN=Txnl1 PE=1 SV=3	MOUSE	2	0.7798301	0.84436399	1.45881403	0.529663384	1.137627006	0.4207266	0.60746378	2.12813902	0.205116197	0.895364821	1.247383	0.7483477	1.38038397	0.90364939	1.737800956
702	3.49	3.49	22.5500003	15.1999995	15.1999995	sp|O54818|T	Tumor protein D53 OS=Mus musculus GN=Tpd52l1 PE=2 SV=1	MOUSE	2	1.03752804	0.97087371	4.09260702	0.253512889	4.246195793	1.13762701	0.84195548	3.46736908	0.328095287	3.944572926	0.7798301	0.51775807	2.0892961	0.369828194	1.629295945
703	3.48	3.53	47.27	19.3900004	13.9400005	sp|O70493|S	Sorting nexin-12 OS=Mus musculus GN=Snx12 PE=2 SV=1	MOUSE	2	0.66680682	0.56907588	1.65958703	0.401790798	1.106624007	0.71121353	0.61485243	1.58489299	0.452897608	1.127197981	0.41304749	0.28767961	1.39315701	0.293765008	0.57543987
704	3.46	8.24	36.7500007	22.4299997	17.6599994	sp|O55137|A	Acyl-coenzyme A thioesterase 1 OS=Mus musculus GN=Acot1 PE=1 SV=1	MOUSE	4	0.67920363	0.57019973	2.29086804	0.151356101	1.55596602	0.44463131	0.78963292	4.96592283	0.083176367	2.208004951	1.23594701	0.65521568	1.43218803	0.862978518	1.976969957
704	0	5.6	37.9599989	14.35	14.35	sp|Q9QYR7|A	Acyl-coenzyme A thioesterase 3 OS=Mus musculus GN=Acot3 PE=2 SV=1	MOUSE	3															
705	3.46	5.67	11.7600001	2.20999997	2.20999997	sp|O88322|N	Nidogen-2 OS=Mus musculus GN=Nid2 PE=1 SV=2	MOUSE	3	1.25892496	0.2090044	1.49968505	0.839460015	1.940886021	1.14815402	0.287696	1.57036305	0.731139123	1.819700956	1.35518897	0.1147595	1.65958703	0.816582382	2.333457947
706	3.44	3.45	20.2500001	8.10000002	8.10000002	sp|Q9DCU9|I	Probable 4-hydroxy-2-oxoglutarate aldolase, mitochondrial OS=Mus musculus GN=Hoga1 PE=1 SV=1	MOUSE	2	0.2443431	0.434389	2.051162	0.078704581	0.501187205	0.2290868	0.44786722	2.46603894	0.073790424	0.564936996	0.61944109	0.68595928	1.61435902	0.322106898	1
707	3.43	3.46	63.4599984	28.8500011	28.8500011	sp|P56382|A	ATP synthase subunit epsilon, mitochondrial OS=Mus musculus GN=Atp5e PE=2 SV=2	MOUSE	2	1.10662401	0.87403143	1.247383	0.88715601	1.393157005	0.93756199	0.81973189	1.40604806	0.666806817	1.318256974	0.73790431	0.37950221	1.57036305	0.461317599	1.158776999
708	3.43	3.43	20.86	13.9799997	9.24699977	sp|Q8R0N6|H	Hydroxyacid-oxoacid transhydrogenase, mitochondrial OS=Mus musculus GN=Adhfe1 PE=2 SV=2	MOUSE	4	0.90364939	0.3011997	1.34276497	0.672976673	1.213389039	0.76559663	0.00409671	1.599558	0.478630096	1.224616051	0.85506672	0.2442838	1.39315701	0.613762021	1.19124198
709	3.43	3.43	17.8399995	9.64900032	9.64900032	sp|Q8K157|G	Aldose 1-epimerase OS=Mus musculus GN=Galm PE=2 SV=1	MOUSE	2	0.40550849	0.33211309	2.12813902	0.190546095	0.862978518	0.37325019	0.30852109	24.8885708	0.031915382	9.289664268	0.60255963	0.4895035	21.0862808	0.042072661	12.70573997
710	3.42	3.87	12.4600001	12.4600001	6.66700006	sp|Q01339|A	Beta-2-glycoprotein 1 OS=Mus musculus GN=Apoh PE=1 SV=1	MOUSE	2	1.45881403	0.2909112	1.51356101	0.963828981	7.655965805	1.44544005	0.3272447	1.72186899	0.839460015	7.870458126	4.7424202	0.03017384	1.97696996	2.398833036	20.32357025
711	3.42	3.48	33.4600002	12.2699998	12.2699998	sp|O88456|C	Calpain small subunit 1 OS=Mus musculus GN=Capns1 PE=2 SV=1	MOUSE	7	0.88715601	0.93625897	17.7010899	0.053951059	15.70363045	1.78648806	0.46774131	3.53183198	0.505824685	6.309574127	1.158777	0.61110842	20.1372395	0.057543989	20.51161957
712	3.42	3.42	23.6599997	4.86200005	4.86200005	sp|Q8BH86|C	UPF0317 protein C14orf159 homolog, mitochondrial OS=Mus musculus PE=2 SV=1	MOUSE	3	0.52480751	0.75606167	23.9883308	0.035974931	12.58924961	1.16949904	0.9238736	6.08134985	0.192309201	7.04693079	0.85506672	0.88609648	2.0892961	0.405508488	1.786488056
713	3.41	3.44	29.8400015	6.04799986	6.04799986	sp|Q9Z2U0|P	Proteasome subunit alpha type-7 OS=Mus musculus GN=Psma7 PE=1 SV=1	MOUSE	2	0.85506672	0.69874311	2.0892961	0.40926069	1.786488056	0.8472274	0.67548162	14.4544001	0.063095734	12.24615955	1.00925303	0.95477623	11.0662403	0.091201082	11.16862965
714	3.37	3.49	15.6299993	3.98700014	3.98700014	sp|Q3TXS7|P	26S proteasome non-ATPase regulatory subunit 1 OS=Mus musculus GN=Psmd1 PE=1 SV=1	MOUSE	2	1.73780096	0.3874464	2.0892961	0.831763685	3.630779982	1.72186899	0.3942284	2.10862804	0.816582382	3.597492933	2.60615301	0.2458092	2.10862804	1.235947013	5.445025921
715	3.37	3.37	23.0800003	8.46199989	8.46199989	sp|Q9ERB0|S	Synaptosomal-associated protein 29 OS=Mus musculus GN=Snap29 PE=1 SV=1	MOUSE	2	1.18032098	0.74029613	2.0892961	0.564936996	2.488857031	1.19124198	0.78976017	3.34194994	0.356451094	3.981071949	1.23594701	0.76340181	4.83058786	0.2558586	5.970353127
716	3.36	3.83	27.6800007	7.81299993	4.91099991	sp|Q62376|R	U1 small nuclear ribonucleoprotein 70 kDa OS=Mus musculus GN=Snrnp70 PE=1 SV=2	MOUSE	2	1.21338904	0.68773872	1.48593605	0.816582382	1.853531957	1	0.99604702	1.27057397	0.787045777	1.258924961	1.05681801	0.99274558	1.41905701	0.744732022	1.51356101
717	3.36	3.36	23.33	8.54199976	5.83299995	sp|Q99M87|D	DnaJ homolog subfamily A member 3, mitochondrial OS=Mus musculus GN=Dnaja3 PE=1 SV=1	MOUSE	2	2.31206489	0.2753942	2.0892961	1.106624007	4.875285149	2.46603894	0.25780609	2.0892961	1.180320978	5.199960232	4.0179081	0.1739959	2.10862804	1.905460954	8.394599915
718	3.34	3.34	34.2400014	14.3999994	14.3999994	sp|Q9D1F4|A	Proline-rich AKT1 substrate 1 OS=Mus musculus GN=Akt1s1 PE=1 SV=1	MOUSE	2	1.88799095	0.53101569	8.8715601	0.212813899	16.59586906	1.75388098	0.57187831	5.70164299	0.307609707	10.18591022	1.48593605	0.69353652	5.39510584	0.275422901	8.165823936
719	3.34	3.34	27.5000006	19.4999994	16.5000007	sp|O54879|H	High mobility group protein B3 OS=Mus musculus GN=Hmgb3 PE=2 SV=3	MOUSE	2	1.92309201	0.29803062	1.48593605	1.29419601	2.884032011	1.57036305	0.41781721	1.247383	1.258924961	1.976969957	1.18032098	0.56034112	1.85353196	0.636795521	2.208004951
720	3.32	3.36	35.2800012	11.3300003	11.3300003	sp|Q99KB8|C	Hydroxyacylglutathione hydrolase, mitochondrial OS=Mus musculus GN=Hagh PE=2 SV=2	MOUSE	2	1.48593605	0.4806625	3.1915381	0.465586096	5.248075008	1.69044101	0.40604141	23.3345795	0.072443459	25.35128975	1.30617094	0.60210502	5.15228701	0.253512889	7.17794323
721	3.31	3.34	24.4900003	13.6099994	13.6099994	sp|Q9CZY3|L	Ubiquitin-conjugating enzyme E2 variant 1 OS=Mus musculus GN=Ube2v1 PE=1 SV=1	MOUSE	2	0.83946002	0.52222133	1.247383	0.672976673	1.047129035	0.76559663	0.59482229	1.72186899	0.440554887	1.318256974	0.87096357	0.64917421	1.19124198	0.731139123	1.037528038
721	0	3.32	22.0699996	13.7899995	13.7899995	sp|Q9D2M8|L	Ubiquitin-conjugating enzyme E2 variant 2 OS=Mus musculus GN=Ube2v2 PE=2 SV=4	MOUSE	2															
722	3.3	3.54	16.6199997	7.91099966	4.88899984	sp|P27546|M	Microtubule-associated protein 4 OS=Mus musculus GN=Map4 PE=1 SV=3	MOUSE	4	1.14815402	0.31946129	2.37684012	0.48305881	2.703958035	0.90364939	0.04852505	1.38038397	0.654636085	1.247382998	0.99083197	0.52061951	1.62929595	0.608134985	1.614359021
723	3.29	3.29	33.8099986	7.4500002	7.4500002	sp|Q8R1G6|F	PDZ and LIM domain protein 2 OS=Mus musculus GN=Pdlim2 PE=1 SV=1	MOUSE	2	1.05681801	0.91864842	1.14815402	0.920449615	1.213389039	0.96382898	0.99304819	1.31825697	0.731139123	1.270573974	1.01859105	0.97519159	1.33045399	0.765596628	1.342764974
724	3.28	3.33	21.7099994	4.44100015	4.44100015	sp|Q91YQ5|F	Dolichyl-diphosphooligosaccharide--protein glycosyltransferase subunit 1 OS=Mus musculus GN=Rpn1 PE=2 S	MOUSE	2	1.13762701	0.5298211	1.70608199	0.666806817	1.976969957	1.21338904	0.58825511	1.70608199	0.711213529	2.070141077	1.94088602	0.1241552	1.72186899	1.127197981	3.46736908
725	3.28	3.3	20.9800005	5.04599996	4.1170001	sp|Q9ERG0|L	LIM domain and actin-binding protein 1 OS=Mus musculus GN=Lima1 PE=1 SV=3	MOUSE	4	1.51356101	0.08308017	1.43218803	1.056818008	2.147830009	1.95884502	0.0581703	1.43218803	1.367728949	2.779712915	1.94088602	0.05687494	1.43218803	1.355188966	2.779712915
726	3.28	3.29	44.1199988	28.43	28.43	sp|P62311|L	U6 snRNA-associated Sm-like protein LSm3 OS=Mus musculus GN=Lsm3 PE=2 SV=2	MOUSE	3	0.81658238	0.7075212	2.0892961	0.390840888	1.706081986	0.80167812	0.68407422	2.0892961	0.383707315	1.67494297	0.4613176	0.30210501	2.10862804	0.220800504	0.972747207
727	3.24	3.24	26.9899994	21.6800004	14.1599998	sp|P46412|G	Glutathione peroxidase 3 OS=Mus musculus GN=Gpx3 PE=2 SV=2	MOUSE	3	0.1803018	0.03689976	2.16770411	0.094623707	0.390840888	0.58076441	0.33298889	1.43218803	0.39445731	0.831763685	0.66069353	0.2917816	1.30617094	0.496592313	0.862978518
728	3.24	3.24	11.8600003	4.63900007	4.63900007	sp|Q9QXC1|F	Fetuin-B OS=Mus musculus GN=Fetub PE=1 SV=1	MOUSE	2	5.29663515	0.50530231	4.24619579	1.247382998	26.54604912	2.6546061	0.47024339	4.20726585	0.630957425	11.37627029	28.5758991	0.1188992	3.94457293	7.244359016	67.29766846
729	3.21	3.28	28.4099996	9.47000012	5.29699996	sp|Q7TMK9|F	Heterogeneous nuclear ribonucleoprotein Q OS=Mus musculus GN=Syncrip PE=1 SV=2	MOUSE	4	1.23594701	0.85188139	11.4815397	0.107646503	14.19058037	0.99083197	0.8328222	3.80189395	0.260615289	3.767038107	1.01859105	0.83767152	5.39510584	0.188799098	5.495409012
730	3.21	3.27	14.7200003	1.90699995	1.90699995	sp|Q8BHN3|(	Neutral alpha-glucosidase AB OS=Mus musculus GN=Ganab PE=1 SV=1	MOUSE	2	0.67297667	0.50162762	21.8776207	0.042072661	14.72313023	0.66069353	0.49103779	7.72680616	0.08550667	5.105050087	0.8472274	0.75279719	2.0892961	0.401790798	1.770109057
731	3.2	3.32	21.3400006	8.83599967	6.6809997	sp|Q91YP0|L	L-2-hydroxyglutarate dehydrogenase, mitochondrial OS=Mus musculus GN=L2hgdh PE=2 SV=1	MOUSE	2	0.4487454	0.29092351	2.0892961	0.212813899	0.937561989	0.42461959	0.274295	2.0892961	0.2013724	0.88715601	0.75857759	0.61672717	2.0892961	0.363078088	1.584892988
732	3.2	3.22	23.0299994	10.3	5.99300005	sp|Q9WVK4|E	EH domain-containing protein 1 OS=Mus musculus GN=Ehd1 PE=1 SV=1	MOUSE	2	0.64268768	0.1250923	1.75388098	0.369828194	1.127197981	0.57016432	0.1177098	1.85353196	0.307609707	1.056818008	0.74473202	0.1176529	1.55596602	0.478630096	1.158776999
733	3.19	3.27	29.1000009	11.1900002	10.0699998	sp|Q9CSN1|S	SNW domain-containing protein 1 OS=Mus musculus GN=Snw1 PE=1 SV=3	MOUSE	4	1.13762701	0.35423249	1.38038397	0.824138105	1.570363045	1.33045399	0.1306705	1.47231197	0.90364939	1.940886021	1.21338904	0.197377	1.95884502	0.619441092	2.398833036
734	3.17	3.17	17.3700005	4.77899984	3.37999985	sp|P56399|U	Ubiquitin carboxyl-terminal hydrolase 5 OS=Mus musculus GN=Usp5 PE=1 SV=1	MOUSE	2	0.69823241	0.55366367	2.93764997	0.239883304	2.051162004	0.51522863	0.35621911	2.22843504	0.233345807	1.14815402	0.58613819	0.4206751	2.10862804	0.280543387	1.235947013
735	3.15	4.36	32.6000005	6.75899983	6.75899983	sp|P97384|A	Annexin A11 OS=Mus musculus GN=Anxa11 PE=1 SV=2	MOUSE	3	1.07646501	0.92617321	7.31139088	0.147231296	7.870458126	1.08642602	0.86162078	2.0892961	0.519995987	2.269865036	1.52756596	0.481994	7.11213493	0.214782998	10.76465034
736	3.12	3.65	27.2199988	24.6800005	20.8900005	sp|Q9CQH7|E	Transcription factor BTF3 homolog 4 OS=Mus musculus GN=Btf3l4 PE=2 SV=1	MOUSE	3	1.31825697	0.56756729	1.30617094	1.009253025	1.706081986	1.25892496	0.65322489	1.23594701	1.018591046	1.541700006	1.12719798	0.77997828	1.19124198	0.946237087	1.330453992
737	3.12	3.15	15.9199998	1.963	1.963	sp|Q63ZW7|I	InaD-like protein OS=Mus musculus GN=Inadl PE=1 SV=2	MOUSE	2	0.87096357	0.28851959	1.73780096	0.501187205	1.51356101	0.93756199	0.57982463	1.38038397	0.679203629	1.29419601	1.03752804	0.62425822	1.38038397	0.751622915	1.432188034
738	3.12	3.15	36.8000001	7.0220001	7.0220001	sp|Q9D7B6|A	Isobutyryl-CoA dehydrogenase, mitochondrial OS=Mus musculus GN=Acad8 PE=2 SV=2	MOUSE	3	0.73113912	0.03843611	2.26986504	0.322106898	1.659587026	0.75857759	0.03965209	2.10862804	0.359749287	1.599557996	0.79432821	0.26307371	1.64437199	0.48305881	1.30617094
739	3.11	3.17	31.0299993	5.57000004	5.57000004	sp|P27601|G	Guanine nucleotide-binding protein subunit alpha-13 OS=Mus musculus GN=Gna13 PE=1 SV=1	MOUSE	2	0.69183099	0.63853931	3.133286	0.218776196	2.167704105	0.87902248	0.75463092	1.38038397	0.636795521	1.213389039	1.00925303	0.95119011	1.38038397	0.731139123	1.393157005
739	0.03	2.21	15.4499993	1.41200004	0.97089997	sp|Q6R0H7|G	Guanine nucleotide-binding protein G(s) subunit alpha isoforms XLas OS=Mus musculus GN=Gnas PE=2 SV=	MOUSE	1															
739	0.02	2.05	13.1899998	2.90200003	2.90200003	sp|P27600|G	Guanine nucleotide-binding protein subunit alpha-12 OS=Mus musculus GN=Gna12 PE=1 SV=3	MOUSE	1															
739	0.01	2.12	8.88300017	4.06100005	2.79200003	sp|P63094|G	Guanine nucleotide-binding protein G(s) subunit alpha isoforms short OS=Mus musculus GN=Gnas PE=1 SV=	MOUSE	1															
739	0	2.08	14.4099996	3.10699996	3.10699996	sp|P18872|G	Guanine nucleotide-binding protein G(o) subunit alpha OS=Mus musculus GN=Gnao1 PE=1 SV=3	MOUSE	1															
739	0	2.07	10.2399997	2.88699996	2.88699996	sp|Q8CGK7|G	Guanine nucleotide-binding protein G(olf) subunit alpha OS=Mus musculus GN=Gnal PE=1 SV=1	MOUSE	1															
739	0	2.05	15.8199996	3.10699996	3.10699996	sp|P50149|G	Guanine nucleotide-binding protein G(t) subunit alpha-2 OS=Mus musculus GN=Gnat2 PE=2 SV=2	MOUSE	1															
739	0	2.04	15.14	3.14299986	3.14299986	sp|P20612|G	Guanine nucleotide-binding protein G(t) subunit alpha-1 OS=Mus musculus GN=Gnat1 PE=1 SV=3	MOUSE	1															
739	0	2.02	13.5600001	3.10699996	3.10699996	sp|B2RSH2|G	Guanine nucleotide-binding protein G(i) subunit alpha-1 OS=Mus musculus GN=Gnai1 PE=2 SV=1	MOUSE	1															
739	0	2.02	10.7299998	3.10699996	3.10699996	sp|Q9DC51|G	Guanine nucleotide-binding protein G(k) subunit alpha OS=Mus musculus GN=Gnai3 PE=1 SV=3	MOUSE	1															
739	0	2.02	14.4099996	3.10699996	3.10699996	sp|Q3V3I2|G	Guanine nucleotide-binding protein G(t) subunit alpha-3 OS=Mus musculus GN=Gnat3 PE=1 SV=2	MOUSE	1															
739	0	2.02	9.57700014	3.09000008	3.09000008	sp|P08752|G	Guanine nucleotide-binding protein G(i) subunit alpha-2 OS=Mus musculus GN=Gnai2 PE=1 SV=5	MOUSE	1															
740	3.11	3.14	22.8200004	4.16099988	3.35600004	sp|Q00PI9|H	Heterogeneous nuclear ribonucleoprotein U-like protein 2 OS=Mus musculus GN=Hnrnpul2 PE=1 SV=1	MOUSE	2	0.99083197	0.99601591	17.8648796	0.055462569	17.70108986	1.02801597	0.94374502	13.8038397	0.074473187	13.93157005	1.158777	0.75625253	15.2756596	0.075857759	16.29295921
741	3.1	3.22	27.5900006	19.6999997	8.86700004	sp|P34022|R	Ran-specific GTPase-activating protein OS=Mus musculus GN=Ranbp1 PE=1 SV=2	MOUSE	3	1.72186899	0.63114059	1.73780096	0.990831971	3.435580015	1.94088602	0.52315462	1.73780096	1.116863012	3.944572926	1.83653796	0.58585697	1.599558	1.14815402	3.837071896
742	3.09	3.09	25.1199991	12.5599995	12.5599995	sp|Q62422|O	Osteoclast-stimulating factor 1 OS=Mus musculus GN=Ostf1 PE=1 SV=2	MOUSE	3	0.67920363	0.4266752	21.8776207	0.042072661	14.85935974	0.57543987	0.26460481	23.3345795	0.038370721	13.42765045	0.99083197	0.73385239	3.98107195	0.248885706	3.944572926
743	3.08	3.09	19.4000006	5.22399992	4.4780001	sp|Q923I7|S	Sodium/glucose cotransporter 2 OS=Mus musculus GN=Slc5a2 PE=2 SV=1	MOUSE	3	0.46989411	0.3687436	2.07014108	0.216770396	0.972747207	0.4325138	0.3562575	2.14783001	0.194088593	0.928966403	0.43651581	0.3850036	2.26986504	0.181970105	0.990831971
744	3.07	3.23	22.5899994	3.18700001	1.68699995	sp|Q5SXY1|C	Cytospin-B OS=Mus musculus GN=Specc1 PE=1 SV=2	MOUSE	2	1.07646501	0.86628717	2.0892961	0.515228629	2.249054909	2.07014108	0.31019729	2.0892961	0.990831971	4.365159035	0.69823241	0.53240478	2.0892961	0.331131101	1.458814025
745	3.04	7.62	25.6099999	18.4900001	18.4900001	sp|P70333|H	Heterogeneous nuclear ribonucleoprotein H2 OS=Mus musculus GN=Hnrnph2 PE=1 SV=1	MOUSE	7	1.00925303	0.9752689	2.0892961	0.48305881	2.108628035	1.12719798	0.79960132	2.0892961	0.539510608	2.355048895	1.18032098	0.72689462	2.0892961	0.564936996	2.488857031
746	3.03	3.37	19.4900006	1.425	1.14000002	sp|Q9R269|P	Periplakin OS=Mus musculus GN=Ppl PE=1 SV=1	MOUSE	2	2.77971292	0.09130256	2.12813902	1.30617094	13.80383968	2.72897792	0.09533887	2.14783001	1.270573974	13.42765045	4.61317587	0.04298532	2.16770411	2.128139019	21.08628082
747	3.03	3.14	28.1399995	5.37899993	5.37899993	sp|O35691|P	Pinin OS=Mus musculus GN=Pnn PE=1 SV=4	MOUSE	3	3.04789496	0.423264	26.5460491	0.114815399	38.72576904	1.97696996	0.4903301	6.48634386	0.304789513	23.33457947	1.80301797	0.57411361	7.24435902	0.248885706	22.08004951

748	3.03	3.08	35.9800011	7.9549998	7.9549998	sp|P97351|R	40S ribosomal protein S3a OS=Mus musculus GN=Rps3a PE=1 SV=3	MOUSE	2	1.08642602	0.71166891	1.247383	0.870963573	1.355188966	0.95499259	0.90732813	1.28233099	0.744732022	1.224616051	1.12719798	0.61694193	1.20226395	0.937561989	1.355188966
749	3.02	3.05	21.2400004	14.5099998	14.5099998	sp|P97314|C	Cysteine and glycine-rich protein 2 OS=Mus musculus GN=Csrp2 PE=1 SV=3	MOUSE	2	0.9289664	0.92546642	2.10862804	0.444631308	1.958845019	0.64863437	0.5710296	7.24435902	0.087096363	4.698941231	0.88715601	0.85578471	2.10862804	0.424619585	1.870682001
750	3.01	5.33	18.0299997	2.64100004	2.64100004	sp|Q9ET47|E	Espin OS=Mus musculus GN=Espn PE=1 SV=2	MOUSE	3	1.75388098	0.3793996	2.01372409	0.870963573	3.698282003	1.70608199	0.39034581	1.35518897	1.258924961	2.511885881	0.77268058	0.57753491	1.57036305	0.39445731	1.213389039
751	3.01	3.01	57.3199987	25.6099999	25.6099999	sp|Q9CQ69|C	Cytochrome b-c1 complex subunit 8 OS=Mus musculus GN=Uqcrq PE=1 SV=3	MOUSE	3	0.58076441	0.1528011	1.81970096	0.316227794	1.056818008	0.50582469	0.02927379	1.58489299	0.313328594	0.801678121	0.73113912	0.2931667	1.62929595	0.444631308	1.19124198
752	2.98	2.98	21.2500006	6.59300014	4.94499989	sp|Q922P9|G	Putative oxidoreductase GLYR1 OS=Mus musculus GN=Glyr1 PE=2 SV=1	MOUSE	2	1.04712904	0.89382738	4.09260702	0.2558586	4.285484791	1.51356101	0.49685711	2.22843504	0.679203629	3.40408206	1.00925303	0.96522951	2.0892961	0.48305881	2.108628035
753	2.96	3.02	34.1399998	10.4400002	8.43399987	sp|P97371|P	Proteasome activator complex subunit 1 OS=Mus musculus GN=Psme1 PE=2 SV=2	MOUSE	3	1.00925303	0.84065592	1.41905701	0.711213529	1.432188034	1.09647799	0.77837062	1.35518897	0.809095919	1.485936046	1.21338904	0.64482582	1.57036305	0.772680581	1.905460954
754	2.96	3	27.9500008	12.6000002	12.6000002	sp|Q91WE2|F	Protein FAM192A OS=Mus musculus GN=Fam192a PE=2 SV=1	MOUSE	3	1.19124198	0.79351062	2.01372409	0.591561615	2.511885881	0.25822601	0.0373529	4.7424202	0.055975761	1.224616051	1.08642602	0.80104899	1.90546095	0.570164323	2.128139019
755	2.95	3.01	10.0599997	5.52400015	4.81600016	sp|Q9QYB5|A	Gamma-adducin OS=Mus musculus GN=Add3 PE=1 SV=2	MOUSE	4	0.72443599	0.4038339	2.70395804	0.26546061	1.958845019	1.41905701	0.32197249	1.25892496	1.127197981	1.770109057	1.22461605	0.53887677	1.247383	0.981747925	1.527565956
756	2.93	3.08	16.9499993	6.82900026	2.19500009	sp|Q9DBX3|S	Sushi domain-containing protein 2 OS=Mus musculus GN=Susd2 PE=1 SV=1	MOUSE	2	1.02801597	0.90334243	1.95884502	0.524807513	2.032356977	0.67297667	0.32430279	1.64437199	0.208929598	1.106624007	0.33419499	0.06198502	2.0892961	0.01028016	0.698232412
757	2.93	2.93	32.7300012	10.5099998	7.20700026	sp|Q925B0|P	PRKC apoptosis WT1 regulator protein OS=Mus musculus GN=Pawr PE=1 SV=2	MOUSE	2	1.33045399	0.60860592	2.24905491	0.591561615	3.019952059	1.25892496	0.56570578	1.64437199	0.765596628	2.089296103	1.16949904	0.75062412	1.64437199	0.711213529	1.923092008
758	2.92	5.69	43.5499996	4.57900018	2.63800006	sp|Q8CJ40|C	Rootletin OS=Mus musculus GN=Crocc PE=1 SV=2	MOUSE	5	0.86297852	0.68737418	2.0892961	0.413047493	1.803017974	0.7177943	0.41949269	2.10862804	0.343558013	1.51356101	0.7798301	0.51389992	2.0892961	0.369828194	1.629295945
759	2.91	2.95	14.5099998	4.53500003	4.53500003	sp|O54734|O	Dolichyl-diphosphooligosaccharide--protein glycosyltransferase 48 kDa subunit OS=Mus musculus GN=Ddost I	MOUSE	2	0.55975759	0.45927069	2.24905491	0.244343102	1.258924961	0.53456438	0.36310399	1.44544005	0.363078088	0.772680581	1.02801597	0.96871471	1.99526203	0.515228629	2.051162004
760	2.9	2.9	32.1399987	15.1800007	15.1800007	sp|Q9D8T7|S	SRA stem-loop-interacting RNA-binding protein, mitochondrial OS=Mus musculus GN=Slirp PE=1 SV=2	MOUSE	2	2.42102909	0.39630801	1.94088602	1.247382998	12.70573997	2.24905491	0.43272561	2.0892961	1.076465011	10.66596031	1.22461605	0.82485479	2.31206489	0.529663384	4.285484791
761	2.89	9.09	43.9000011	28.5599998	22.36	sp|P68510|14	14-3-3 protein eta OS=Mus musculus GN=Ywhah PE=1 SV=2	MOUSE	7	1.01859105	0.96025091	1.92309201	0.529663384	1.940886021	1.12719798	0.73044473	1.38038397	0.816582382	1.55596602	1.70608199	0.33309409	1.49968505	1.137627006	2.703958035
762	2.89	2.91	42.7899987	13.5100007	9.00899991	sp|Q9D0B0|S	Serine/arginine-rich splicing factor 9 OS=Mus musculus GN=Srsf9 PE=1 SV=1	MOUSE	2	0.85506672	0.73434877	2.29086804	0.373250186	1.958845019	1.27057397	0.59896529	2.0892961	0.608134985	2.654606104	1.03752804	0.91659582	2.0892961	0.496592313	2.167704105
763	2.86	3.28	14.79	0.96899997	0.66749998	sp|Q9JHU4|D	Cytoplasmic dynein 1 heavy chain 1 OS=Mus musculus GN=Dync1h1 PE=1 SV=2	MOUSE	2	1.20226395	0.70230353	2.0892961	0.57543987	2.53512907	1.05681801	0.89881033	2.0892961	0.505824685	2.208004951	0.71121353	0.55389792	2.0892961	0.340408266	1.485936046
764	2.86	2.86	9.79100019	3.69199999	2.568	sp|Q6P1B1|X	Xaa-Pro aminopeptidase 1 OS=Mus musculus GN=Xpnpep1 PE=2 SV=1	MOUSE	2	1.00925303	0.40893611	1.14815402	0.879022479	1.158776999	0.99083197	0.8074742	1.14815402	0.862978518	1.137627006	1.10662401	0.13984799	1.158777	0.954992592	1.28233099
765	2.85	3.14	30.3600013	4.55899984	3.00699994	sp|Q8CH25|S	SAFB-like transcription modulator OS=Mus musculus GN=Sltm PE=1 SV=1	MOUSE	4	0.83946002	0.55250979	1.45881403	0.570164323	1.224616051	1	0.95359999	1.75388098	0.570164323	1.737800956	1.07646501	0.70417279	3.872576	0.277971298	4.207265854
766	2.84	2.84	22.4199995	10.3	10.3	sp|Q9CY62|R	E3 ubiquitin-protein ligase RNF181 OS=Mus musculus GN=Rnf181 PE=2 SV=1	MOUSE	2	1.30617094	0.65712202	1.27057397	1.028015971	1.721868992	1.23594701	0.55979133	1.40604806	0.879022479	1.786488056	0.87096357	0.85653472	1.22461605	0.679203629	1.066596031
767	2.83	2.85	9.47199985	1.09799998	1.09799998	sp|P35822|P	Receptor-type tyrosine-protein phosphatase kappa OS=Mus musculus GN=Ptprk PE=1 SV=1	MOUSE	2	1.28233099	0.7013002	1.29419601	0.990831971	1.67494297	1.29419601	0.85023642	1.61435902	0.801678121	2.108628035	0.3981072	0.34795219	1.99526203	0.152756602	0.794328213
768	2.81	2.84	37.9599989	26.85	22.2200006	sp|P62492|R	Ras-related protein Rab-11A OS=Mus musculus GN=Rab11a PE=1 SV=3	MOUSE	3	0.80167812	0.2733393	1.41905701	0.564936996	1.137627006	0.85506672	0.42877621	1.31825697	0.648634374	1.127197981	0.95499259	0.67053503	1.27057397	0.751622915	1.213389039
769	2.79	2.79	24.0400001	24.0400001	17.3099995	sp|P84089|E	Enhancer of rudimentary homolog OS=Mus musculus GN=Erh PE=1 SV=1	MOUSE	2	0.88715601	0.70807427	1.55596602	0.564936996	1.380383968	1.06659603	0.58086038	1.78648806	0.597035289	1.905460954	0.80909592	0.4725163	1.75388098	0.461317599	1.419057012
770	2.78	2.78	18.1500003	7.11700022	7.11700022	sp|Q4VAA2|C	Protein CDV3 OS=Mus musculus GN=Cdv3 PE=1 SV=2	MOUSE	2	0.74473202	0.55368477	2.46603894	0.301995188	1.836537957	0.88715601	0.78785658	1.78648806	0.496592313	1.584892988	0.87096357	0.76587552	1.41905701	0.608134985	1.235947013
771	2.74	3.06	14.8699999	9.23099965	5.64099997	sp|Q9D826|S	Peroxisomal sarcosine oxidase OS=Mus musculus GN=Pipox PE=2 SV=1	MOUSE	2	0.52966338	0.36653361	2.20800495	0.222843498	1.16949904	0.76559663	0.64053738	7.17794323	0.103752799	5.495409012	0.72443599	0.58162898	2.0892961	0.343558013	1.51356101
772	2.73	2.74	31.310001	12.1200003	12.1200003	sp|O35988|S	Syndecan-4 OS=Mus musculus GN=Sdc4 PE=1 SV=1	MOUSE	2	1.158777	0.53437859	1.75388098	0.660693526	2.032356977	1.11686301	0.73720241	1.599558	0.698232412	1.786488056	1.00925303	0.99408752	1.247383	0.809095919	1.258924961
773	2.72	2.73	25.7600009	7.57599995	7.57599995	sp|Q6P069|S	Sorcin OS=Mus musculus GN=Sri PE=1 SV=1	MOUSE	2	1.10662401	0.99446338	1.77010906	0.625172675	1.976969957	0.71121353	0.44393149	1.62929595	0.432513803	1.158776999	0.96382898	0.86642849	1.78648806	0.544502676	1.721868992
774	2.71	2.77	14.4299999	2.09999997	2.09999997	sp|P55014|S	Solute carrier family 12 member 1 OS=Mus musculus GN=Slc12a1 PE=2 SV=2	MOUSE	2	0.53456438	0.35961759	2.33345795	0.231206506	1.247382998	0.66680682	0.50879878	2.10862804	0.319153786	1.406048059	0.4655861	0.31383669	2.10862804	0.222843498	0.981747925
775	2.71	2.75	16.2499994	4.09100018	1.44400001	sp|Q8VHF2|C	Cadherin-related family member 5 OS=Mus musculus GN=Cdhr5 PE=2 SV=1	MOUSE	1	1.47231197	0.48158869	1.41905701	1.037528038	2.128139019	1.23594701	0.62582952	1.247383	0.990831971	1.570363045	1.14815402	0.75380617	1.36772895	0.839460015	1.584892988
776	2.71	2.73	36.6400003	18.3200002	18.3200002	sp|Q9CQZ5|N	NADH dehydrogenase [ubiquinone] 1 alpha subcomplex subunit 6 OS=Mus musculus GN=Ndufa6 PE=1 SV=1	MOUSE	2	0.80167812	0.49949941	1.28233099	0.619441092	1.028015971	0.7798301	0.28779221	1.36772895	0.570164323	1.066596031	0.80909592	0.43810529	1.41905701	0.564936996	1.14815402
777	2.7	2.7	41.5699989	20.7900003	8.42700005	sp|P53996|C	Cellular nucleic acid-binding protein OS=Mus musculus GN=Cnbp PE=2 SV=2	MOUSE	1	1.14815402	0.84384638	1.27057397	0.90364939	1.458814025	1.12719798	0.333208	1.158777	0.972747207	1.30617094	0.9289664	0.39308769	1.247383	0.744732022	1.158776999
778	2.69	5.06	16.9100002	2.21699998	2.21699998	sp|Q80XI3|IF	Eukaryotic translation initiation factor 4 gamma 3 OS=Mus musculus GN=Eif4g3 PE=1 SV=2	MOUSE	3	1.27057397	0.52935618	2.29086804	0.55462569	2.964831114	1.40604806	0.5350157	1.38038397	1.018591046	1.940886021	1.40604806	0.38814631	4.4463129	0.316227794	6.251727104
779	2.68	2.9	22.8400007	5.01400009	3.06400005	sp|Q8BUK6|H	Protein Hook homolog 3 OS=Mus musculus GN=Hook3 PE=1 SV=2	MOUSE	2	0.82413811	0.82251889	1.39315701	0.591561615	1.14815402	1.10662401	0.83418471	2.29086804	0.48305881	2.582259893	1.48593605	0.65983993	2.10862804	0.704693079	3.191538095
780	2.66	2.67	20.1700002	2.50000004	0.93019996	sp|P58871|T	182 kDa tankyrase-1-binding protein OS=Mus musculus GN=Tnks1bp1 PE=1 SV=2	MOUSE	1	8.8715601	0.07945903	2.91071701	3.047894955	24.21029091	10.6659603	0.04158142	2.91071701	3.66437602	29.37649918	11.2719698	0.00411423	3.01995206	3.732501984	30.76096916
781	2.65	2.74	22.3399997	6.09100014	3.89200002	sp|Q8C854|M	Myelin expression factor 2 OS=Mus musculus GN=Myef2 PE=1 SV=1	MOUSE	2	1.12719798	0.73190051	1.90546095	0.591561615	2.147830009	1.12719798	0.79589689	1.57036305	0.717794299	1.786488056	1.16949904	0.64876741	1.67494297	0.698232412	1.976969957
782	2.64	2.79	32.4800015	14.9700001	2.86599994	sp|Q9JLZ3|A	Methylglutaconyl-CoA hydratase, mitochondrial OS=Mus musculus GN=Auh PE=2 SV=1	MOUSE	1	0.64863437	0.27346921	1.69044101	0.383707315	1.096477985	0.73790431	0.2168788	1.45881403	0.501187205	1.076465011	0.64268768	0.0857229	1.47231197	0.428548515	0.946237087
783	2.64	2.66	28.0600011	12.7599999	12.7599999	sp|P61759|P	Prefoldin subunit 3 OS=Mus musculus GN=Vbp1 PE=2 SV=2	MOUSE	3	0.8472274	0.47899801	1.22461605	0.685488224	1.037528038	0.79432821	0.3525292	1.158777	0.679203629	0.920449615	0.61944109	0.22560009	1.40604806	0.428548515	0.870963573
784	2.62	2.65	34.3600005	10.5700001	6.60799965	sp|Q9WTP7|F	GTP:AMP phosphotransferase, mitochondrial OS=Mus musculus GN=Ak3 PE=1 SV=3	MOUSE	2	0.85506672	0.51667178	1.49968505	0.564936996	1.28233099	0.87096357	0.57275951	1.58489299	0.544502676	1.380383968	0.7798301	0.4830797	1.28233099	0.57543987	1
785	2.59	2.61	27.64	20.3299999	20.3299999	sp|Q9Z0F7|S	Gamma-synuclein OS=Mus musculus GN=Sncg PE=1 SV=1	MOUSE	2	1.55596602	0.13378081	2.83139205	0.549540877	4.487453938	1.61435902	0.1195932	1.97696996	0.816582382	3.221069098	1.38038397	0.147239	2.3550489	0.586138189	3.311311007
786	2.59	2.6	11.4799999	8.37299973	6.6990003	sp|P54775|P	26S protease regulatory subunit 6B OS=Mus musculus GN=Psmc4 PE=1 SV=2	MOUSE	2	0.73113912	0.68182403	20.8929596	0.044463132	15.27565956	0.45289761	0.38388899	19.9526196	0.036643758	9.036495209	0.474242	0.3987771	17.8648796	0.039084092	8.472273827
787	2.59	2.59	10.9899998	10.9899998	10.9899998	sp|Q8C8T8|T	Pre-rRNA-processing protein TSR2 homolog OS=Mus musculus GN=Tsr2 PE=2 SV=1	MOUSE	2	3.16227794	0.84152842	27.5422897	0.114815399	42.46195984	5.10505009	0.58466488	31.0456009	0.164437205	50.11872101	3.80189395	0.94462848	29.3764992	0.129419595	43.25138092
788	2.58	2.78	22.6799995	4.41399999	1.52200004	sp|Q7TMS5|A	ATP-binding cassette sub-family G member 2 OS=Mus musculus GN=Abcg2 PE=1 SV=1	MOUSE	1	0.59703529	0.1759577	1.41905701	0.373250186	0.847227395	0.73790431	0.1267754	1.30617094	0.544502676	0.963828981	0.65463609	0.1143104	1.33045399	0.452897608	0.870963573
789	2.58	2.71	22.1000001	9.78299975	7.97099993	sp|Q9JHW2|N	Omega-amidase NIT2 OS=Mus musculus GN=Nit2 PE=1 SV=1	MOUSE	2	0.80167812	0.29453549	1.95884502	0.40926069	1.570363045	0.78704578	0.19113991	1.38038397	0.570164323	1.08642602	0.79432821	0.2472503	1.65958703	0.48305881	1.318256974
790	2.58	2.63	33.2500011	7.65599981	6.21999987	sp|P30275|K	Creatine kinase U-type, mitochondrial OS=Mus musculus GN=Ckmt1 PE=1 SV=1	MOUSE	1															
791	2.56	2.65	14.3900007	5.56799993	4.40800004	sp|Q8C1B7|S	Septin-11 OS=Mus musculus GN=Sept11 PE=1 SV=4	MOUSE	2	0.80909592	0.6988976	2.0892961	0.387257606	1.690441012	0.85506672	0.77315432	2.0892961	0.40926069	1.786488056	1.41905701	0.52687693	2.0892961	0.679203629	2.964831114
792	2.56	2.62	27.1400005	4.01099995	4.01099995	sp|P16332|M	Methylmalonyl-CoA mutase, mitochondrial OS=Mus musculus GN=Mut PE=2 SV=2	MOUSE	2	0.87902248	0.85483593	2.77971292	0.316227794	2.443430901	0.87902248	0.83403748	2.24905491	0.390840888	1.976969957	0.97274721	0.95212477	3.37287307	0.288403213	3.28095293
793	2.53	2.53	23.8800004	23.8800004	23.8800004	sp|P03930|A	ATP synthase protein 8 OS=Mus musculus GN=Mtatp8 PE=1 SV=1	MOUSE	2	1.00925303	0.96510732	1.18032098	0.855066717	1.19124198	0.82413811	0.67177862	1.39315701	0.591561615	1.14815402	1.04712904	0.82487398	1.18032098	0.88715601	1.235947013
794	2.52	2.53	17.4899995	8.37400034	3.20199989	sp|P60843|IF	Eukaryotic initiation factor 4A-I OS=Mus musculus GN=Eif4a1 PE=1 SV=1	MOUSE	2	0.40926069	0.17868461	1.80301797	0.216770396	0.73790431	0.34355801	0.23086619	2.24905491	0.148593605	0.772680581	0.55462569	0.48824301	2.12813902	0.258226007	1.180320978
794	0	2.01	26.2899995	3.19399983	3.19399983	sp|P10630|IF	Eukaryotic initiation factor 4A-II OS=Mus musculus GN=Eif4a2 PE=2 SV=2	MOUSE	2															
795	2.5	2.59	24.56	18.9300001	13.6099994	sp|Q922Q1|M	MOSC domain-containing protein 2, mitochondrial OS=Mus musculus GN=Mosc2 PE=1 SV=1	MOUSE	3	0.9289664	0.94468689	1.28233099	0.724435985	1.19124198	0.91201079	0.89248139	1.247383	0.731139123	1.137627006	0.80167812	0.68063659	1.247383	0.642687678	1
796	2.48	3.16	8.10500011	3.42499986	2.74	sp|P70168|IM	Importin subunit beta-1 OS=Mus musculus GN=Kpnb1 PE=1 SV=2	MOUSE	3															
797	2.48	2.48	45.8299995	16.6700006	10.83	sp|Q9CQ54|N	NADH dehydrogenase [ubiquinone] 1 subunit C2 OS=Mus musculus GN=Ndufc2 PE=1 SV=1	MOUSE	1	0.73113912	0.72745383	1.35518897	0.534564376	0.990831971	0.8472274	0.71799493	1.158777	0.731139123	0.981747925	0.76559663	0.67176598	1.31825697	0.580764413	1.009253025
798	2.47	2.47	71.6199994	58.1099987	58.1099987	sp|Q9CQN3|T	Mitochondrial import receptor subunit TOM6 homolog OS=Mus musculus GN=Tomm6 PE=2 SV=1	MOUSE	3	1.08642602	0.91292232	1.247383	0.870963573	1.355188966	1.19124198	0.75544709	1.70608199	0.698232412	2.032356977	1.06659603	0.95491213	1.247383	0.855066717	1.330453992
799	2.46	2.46	25.5699992	5.57399988	5.57399988	sp|Q99M28|R	RNA-binding protein with serine-rich domain 1 OS=Mus musculus GN=Rnps1 PE=2 SV=1	MOUSE	2	1.08642602	0.79860878	1.72186899	0.630957425	1.870682001	0.67920363	0.86389178	1.55596602	0.436515808	1.056818008	0.63679552	0.66317499	1.27057397	0.496592313	0.809095919
800	2.42	2.54	25.2299994	4.09500003	1.98199991	sp|Q8BGC0|H	HIV Tat-specific factor 1 homolog OS=Mus musculus GN=Htatsf1 PE=1 SV=1	MOUSE	1	11.2719698	0.07016437	5.49540901	2.051162004	49.20394897	3.2210691	0.0568011	5.64937019	0.570164323	16.44371986	4.83058786	0.04199391	6.02559614	0.801678121	25.35128975
801	2.42	2.46	18.8899994	4.83000018	3.26699987	sp|P58735|S	Sulfate anion transporter 1 OS=Mus musculus GN=Slc26a1 PE=2 SV=1	MOUSE	1															
802	2.41	2.41	13.8500005	8.10799971	5.06800003	sp|Q91XE0|G	Glycine N-acyltransferase OS=Mus musculus GN=Glyat PE=1 SV=1	MOUSE	1	0.2558586	0.24157961	3.34194994	0.059156161	0.855066717	0.03250873	0.135049	6.66806698	0.01009253	0.216770396	0.09549926	0.173408	4.78630114	0.015848931	0.457088202
803	2.4	2.48	32.2899997	27.0799995	21.8799993	sp|P62627|D	Dynein light chain roadblock-type 1 OS=Mus musculus GN=Dynlrb1 PE=1 SV=3	MOUSE	2	1.13762701	0.77899683	2.0892961	0.544502676	2.376840115	1.18032098	0.7270326	2.0892961	0.564936996	2.466038942	1.16949904	0.74578548	2.0892961	0.55975759	2.443430901
804	2.4	2.46	14.1599998	2.52800006	1.64299998	sp|Q8K4Z5|S	Splicing factor 3A subunit 1 OS=Mus musculus GN=Sf3a1 PE=1 SV=1	MOUSE	1	1.38038397	0.56198782	17.7010899	0.077983007	19.0546093	1.40604806	0.53885943	18.03018	0.077983007	19.7696991	1.41905701	0.51591861	6.48634386	0.218776196	9.120108604
805	2.39	2.43	46.6699988	16.6700006	13.8099998	sp|Q9D1J3|S	SAP domain-containing ribonucleoprotein OS=Mus musculus GN=Sarnp PE=1 SV=3	MOUSE	3	1.33045399	0.49404421	2.0892961	0.636795521	2.805433989	1.45881403	0.4155342	2.93764997	0.496592313	4.285484791	1.13762701	0.70793402	9.81747913	0.115877703	11.16862965
806	2.39	2.41	20.1399997	7.91400001	7.91400001	sp|P24668|M	Cation-dependent mannose-6-phosphate receptor OS=Mus musculus GN=M6pr PE=1 SV=1	MOUSE	2	1.52756596	0.66175962	1.25892496	1.213389039	1.905460954	1.49968505	0.68919098	1.44544005	1.037528038	2.208004951	1.26770411	0.4760738	1.30617094	1.659587026	2.937649965
807	2.38	4.48	32.7300012	26.0600001	20.0000003	sp|Q9R0P5|D	Destrin OS=Mus musculus GN=Dstn PE=1 SV=3	MOUSE	4	0.94623709	0.96481192	6.54636192	0.144544005	6.194410801	0.71121353	0.68762428	6.54636192	0.105681702	4.655860901	0.65463609	0.61485291	5.10505009	0.122461602	3.34194994
808	2.38	2.38	9.38600004	5.77600002	4.33200002	sp|Q3TEA8|H	Heterochromatin protein 1-binding protein 3 OS=Mus musculus GN=Hp1bp3 PE=1 SV=1	MOUSE	2	0.89536482	0.83778459	2.0892961	0.428548515	1.870682001	0.92044962	0.87610018	2.0892961	0.440554887	1.923092008	1.62929595	0.42629579	2.10862804	0.772680581	3.40408206
809	2.37	4.7	31.4500004	13.6500001	5.638	sp|Q9JHL1|N	Na(+)/H(+) exchange regulatory cofactor NHE-RF2 OS=Mus musculus GN=Slc9a3r2 PE=1 SV=2	MOUSE	3	1.43218803	0.96825928	1.69044101	0.847227395	2.443430901	1.16949904	0.75443971	1.49968505	0.779830098	1.836537957	1.19124198	0.62078619	1.33045399	0.895364821	1.659587026
810	2.37	2.43	36.2100005	26.7199993	16.3800001	sp|Q9CPP6|N	NADH dehydrogenase [ubiquinone] 1 alpha subcomplex subunit 5 OS=Mus musculus GN=Ndufa5 PE=1 SV=3	MOUSE	1	1.03752804	0.90067989	1.61435902	0.642687678	1.706081986	1.18032098	0.55483311	1.43218803	0.824138105	1.737800956	0.96382898	0.81285793	1.73780096	0.55462569	1.67494297
811	2.36	2.45	38.5800004	12.6900002	7.61400014	sp|Q9WVB0|R	RNA-binding protein with multiple splicing OS=Mus musculus GN=Rbpms PE=2 SV=1	MOUSE	1	1.55596602	0.49128372	2.42102909	0.642687678	4.130475044	2.4434309	0.23168241	1.58489299	1.541700006	6.194410801	2.60615301	0.2375681	2.42102909	1.076465011	7.870458126
812	2.36	2.39	37.439999	20.7699999	4.34800014	sp|Q921F2|T	TAR DNA-binding protein 43 OS=Mus musculus GN=Tardbp PE=1 SV=1	MOUSE	1	0.94623709	0.98910987	1.34276497	0.698232412	1.270573974	0.78704578	0.80779672	1.49968505	0.474242002	1.180320978	0.32809529	0.36219499	2.70395804	0.051999599	0.88715601
813	2.35	2.39	11.9000003	5.12799993	2.56399997	sp|Q3UHB1|N	5'-nucleotidase domain-containing protein 3 OS=Mus musculus GN=Nt5dc3 PE=2 SV=1	MOUSE	1	1.09647799	0.83823162	2.0892961	0.524807513	2.290868044	1.03752804	0.9236787	2.0892961	0.496592313	2.167704105	1.11686301	0.80433577	2.0892961	0.534564376	2.355048895
814	2.35	2.35	51.0200024	34.6899986	27.55	sp|P50543|S	Protein S100-A11 OS=Mus musculus GN=S100a11 PE=2 SV=1	MOUSE	6	1.13762701	0.6058858	1.28233099	0.88715601	1.472311974	1.39315701	0.8244288	1.34276497	1.037528038	1.905460954	1.52756596	0.26378039	1.31825697	1.158776999	2.032356977
815	2.33	2.44	25.029999	2.30199993	1.00100003	sp|Q921M4|G	Golgin subfamily A member 2 OS=Mus musculus GN=Golga2 PE=1 SV=3	MOUSE	1	1.158777	0.30411992	4.4463129	0.260615289	5.152287006	0.4920395	0.26624781	4.6558609	0.087096363	2.290868044	0.65463609	0.05469818	4.4463129	0.147231296	2.91071701
816	2.27	2.32	32.2600007	16.99	10.9700002	sp|P32261|A	Antithrombin-III OS=Mus musculus GN=Serpinc1 PE=1 SV=1	MOUSE	2															
817	2.27	2.28	25.4999995	6.30400032	4.29800004	sp|Q60866|P	Phosphotriesterase-related protein OS=Mus musculus GN=Pter PE=2 SV=1	MOUSE	2	0.1584893	0.30016431	4.4463129	0.035645109	0.704693079	1.40604806	0.60584033	4.13047504	0.340408206	5.915616989	1.16949904	0.82146549	2.72897792	0.428548515	3.221069098
818	2.27	2.27	9.73500013	6.78500012	6.78500012	sp|P10605|C	Cathepsin B OS=Mus musculus GN=Ctsb PE=1 SV=2	MOUSE	2	0.73790431	0.89954102	15.8489304	0.050118718	11.69499016	1.38038397	0.72325528	19.9526196	0.069183096	21.47830009	1.52756596	0.59737372	2.10862804	0.724435985	3.221069098
819	2.26	2.26	37.0099992	25.9799987	20.4699993	sp|Q03958|P	Prefoldin subunit 6 OS=Mus musculus GN=Pfdn6 PE=2 SV=1	MOUSE	2	1.99526203	0.79686427	24.6603909	0.080909587	28.05434036	2.22843504	0.78220129	25.8225994	0.086297847	29.64830971	2.10862804	0.82231402	25.3512898	0.083176367	28.84030914
820	2.26	2.26	20.2600002	10.4599997	5.88200018	sp|Q91WQ9|C	Calmodulin-like protein 4 OS=Mus musculus GN=Calml4 PE=2 SV=2	MOUSE	2	1.21338904	0.42240909	1.38038397	0.879022479	1.67494297	0.87096357	0.37909919	1.45881403	0.597035289	1.270573974	0.97274721	0.96586698	1.40604806	0.691830993	1.367728949
821	2.25	2.25	11.7399998	7.19700009	4.54499982	sp|Q9EST4|P	Proteasome assembly chaperone 2 OS=Mus musculus GN=Psmg2 PE=2 SV=1	MOUSE	1															
822	2.24	2.97	25.8100003	20.0000003	12.2599997	sp|P61148|F	Heparin-binding growth factor 1 OS=Mus musculus GN=Fgf1 PE=2 SV=1	MOUSE	2															
823	2.23	2.31	18.8999996	4.97200005	1.10499999	sp|Q811D0|D	Disks large homolog 1 OS=Mus musculus GN=Dlg1 PE=1 SV=1	MOUSE	1	1.31825697	0.60468829	2.10862804	0.625172675	2.754229069	1.158777	0.76377589	2.0892961	0.55462569	2.421029091	1.27057397	0.64146328	2.0892961	0.608134985	2.654606104
824	2.23	2.28	17.52	7.64300004	4.14000005	sp|P11862|G	Growth arrest-specific protein 2 OS=Mus musculus GN=Gas2 PE=1 SV=1	MOUSE	1	0.36982819	0.2404536	27.2897797	0.029376499	10.09253025	0.67920363	0.50834388	21.8776207	0.042072661	14.85935974	0.60255963	0.42577729	2.10862804	0.288403213	1.270573974
825	2.23	2.24	21.9600007	9.14100036	8.32199976	sp|P10637|T	Microtubule-associated protein tau OS=Mus musculus GN=Mapt PE=1 SV=3	MOUSE	5	2.07014108	0.08076617	1.64437199	1.258924961	3.872575998	1.88799095	0.1865444	1.72186899	1.096477985	3.499452114	1.51356101	0.1066947	1.64437199	0.920449615	2.728977919
826	2.22	2.48	20.8700001	11.1699998	2.91300006	sp|Q9JKR7|P	Peptidyl-prolyl cis-trans isomerase F, mitochondrial OS=Mus musculus GN=Ppif PE=2 SV=1	MOUSE	1	0.63679552	0.48998421	5.49540901	0.115877703	3.499452114	0.62517268	0.45169631	3.46736908	0.1786488	2.167704105	0.57016432	0.36998409	2.0892961	0.270395786	1.19124198

826	0	2.01	19.6700007	2.45900005	2.45900005	RRRRRsp|Q9	REVERSED Tubulin-folding cofactor B OS=Mus musculus GN=Tbcb PE=1 SV=2	MOUSE	1															
827	2.2	2.2	24.7500002	10.61	6.061	sp|P56213|AL	FAD-linked sulfhydryl oxidase ALR OS=Mus musculus GN=Gfer PE=2 SV=2	MOUSE	1	0.81658238	0.89680678	1.95884502	0.387257606	1.599557996	0.95499259	0.83579511	2.6546061	0.359749287	2.53512907	0.27039579	0.61747551	3.63077998	0.058076441	0.981747925
828	2.19	2.41	17.7900001	6.12899996	3.58700007	sp|Q3UMU9|I	Hepatoma-derived growth factor-related protein 2 OS=Mus musculus GN=Hdgfrp2 PE=1 SV=1	MOUSE	2	1.49968505	0.4726648	2.0892961	0.717794299	3.162277937	1.18032098	0.73452038	2.0892961	0.564936996	2.466038942	1.31825697	0.60188538	2.58225989	0.510505021	3.40408206
829	2.19	2.27	66.2000001	53.52	36.62	sp|Q06185|A	ATP synthase subunit e, mitochondrial OS=Mus musculus GN=Atp5i PE=1 SV=2	MOUSE	7	1.85353196	0.79852247	1.85353196	1	3.372873068	1.62929595	0.89541161	1.78648806	0.912010789	2.964831114	2.48885703	0.70061493	2.10862804	1.180320978	4.742420197
830	2.18	2.38	24.2400006	1.80500001	0.97559998	sp|Q9JMH9|M	Myosin-XVIIIa OS=Mus musculus GN=Myo18a PE=1 SV=2	MOUSE	2	1.31825697	0.3943904	1.48593605	0.88715601	1.958845019	1.31825697	0.4570019	1.72186899	0.765596628	2.290868044	1.49968505	0.276842	1.870682	0.801678121	2.805433989
831	2.17	2.56	21.0299999	3.82299982	1.07500004	sp|Q6P542|A	ATP-binding cassette sub-family F member 1 OS=Mus musculus GN=Abcf1 PE=1 SV=1	MOUSE	1	1.870682	0.44145581	4.61317587	0.405508488	9.638290405	1.81970096	0.46061519	4.61317587	0.39445731	9.20449543	0.89536482	0.67869282	4.52897596	0.199526206	4.055085182
832	2.17	2.48	19.3700001	3.33300009	1.25000002	sp|P58281|OI	Dynamin-like 120 kDa protein, mitochondrial OS=Mus musculus GN=Opa1 PE=1 SV=1	MOUSE	1	0.92044962	0.88475102	2.0892961	0.440554887	1.923092008	1.35518897	0.56823742	2.0892961	0.648634374	2.85758996	0.96382898	0.95305032	2.0892961	0.461317599	2.013724089
833	2.17	2.24	15.7600001	6.35299981	4.94100004	sp|Q9DCL9|F	Multifunctional protein ADE2 OS=Mus musculus GN=Paics PE=1 SV=4	MOUSE	2	1.158777	0.77002221	1.65958703	0.698232412	1.905460954	1.19124198	0.71516281	1.41905701	0.839460015	1.690441012	0.97274721	0.9346745	1.44544005	0.672976673	1.406048059
834	2.17	2.19	16.6700006	6.81800023	6.81800023	sp|Q7M6Y3|F	Phosphatidylinositol-binding clathrin assembly protein OS=Mus musculus GN=Picalm PE=1 SV=1	MOUSE	3	1.27057397	0.80095363	3.43558002	0.369828194	5.199960232	1.78648806	0.48717269	4.28548479	0.416869402	8.472273827	1.88799095	0.44495869	2.72897792	0.691830993	7.655965805
835	2.17	2.19	14.8399994	9.18700025	7.06700012	sp|Q9CQ65|N	S-methyl-5'-thioadenosine phosphorylase OS=Mus musculus GN=Mtap PE=2 SV=1	MOUSE	1	0.57016432	0.39340481	2.10862804	0.27289781	1.202263951	0.68548822	0.5194521	2.0892961	0.328095287	1.432188034	1.20226395	0.71008468	2.0892961	0.57543987	2.511885881
836	2.17	2.17	30.7099998	9.28599984	6.07100017	sp|O35387|H	HCLS1-associated protein X-1 OS=Mus musculus GN=Hax1 PE=1 SV=1	MOUSE	1															
837	2.17	2.17	9.70600024	5.5879999	3.52899991	sp|Q8BFZ9|E	Erlin-2 OS=Mus musculus GN=Erlin2 PE=1 SV=1	MOUSE	2	0.98174793	0.89155752	1.21338904	0.809095919	1.19124198	1.06659603	0.83341181	1.16949904	0.912010789	1.247382998	1.06659603	0.84988612	1.13762701	0.937561989	1.213389039
837	0	2.02	15.6100005	3.46800014	3.46800014	sp|Q91X78|E	Erlin-1 OS=Mus musculus GN=Erlin1 PE=2 SV=1	MOUSE	2															
838	2.16	2.17	22.9499996	8.90400037	6.50700033	sp|Q91Z38|T	Tetratricopeptide repeat protein 1 OS=Mus musculus GN=Ttc1 PE=2 SV=1	MOUSE	1	1.22461605	0.68311262	2.0892961	0.586138189	2.582259893	1.06659603	0.88774061	2.10862804	0.505824685	2.22843504	1.00925303	0.97013879	2.0892961	0.48305881	2.108628035
839	2.15	2.21	17.9000005	7.22900033	4.47500013	sp|Q505F5|LI	Leucine-rich repeat-containing protein 47 OS=Mus musculus GN=Lrrc47 PE=1 SV=1	MOUSE	2	0.88715601	0.86229378	2.10862804	0.424619585	1.870682001	1.29419601	0.68495822	2.10862804	0.613762021	2.703958035	1.36772895	0.62585157	2.0892961	0.654636085	2.85756996
840	2.14	2.42	32.4400008	3.05100009	1.339	sp|P23116|EI	Eukaryotic translation initiation factor 3 subunit A OS=Mus musculus GN=Eif3a PE=1 SV=5	MOUSE	1	1.65958703	0.1709879	3.56451106	0.465586096	6.982324123	1.70608199	0.1678033	4.0179081	0.424619585	7.655965805	2.07014108	0.03898845	4.09260702	0.505824685	9.462371826
841	2.14	2.31	10.5899997	7.76500031	2.11800002	sp|Q9WVM8|	Kynurenine/alpha-aminoadipate aminotransferase, mitochondrial OS=Mus musculus GN=Aadat PE=2 SV=1	MOUSE	1	0.89536482	0.79640412	1.58489299	0.564936996	1.419057012	0.76559663	0.80458248	1.75388098	0.420726597	1.342764974	0.74473202	0.61944908	1.36772895	0.539510608	1.018591046
842	2.14	2.2	45.0500011	12.0899998	5.12799993	sp|Q91VN4|C	Coiled-coil-helix-coiled-coil-helix domain-containing protein 6 OS=Mus musculus GN=Chchd6 PE=1 SV=1	MOUSE	1	1.02801597	0.63359261	1.247383	0.824138105	1.28233099	0.96382898	0.80751109	1.247383	0.772680581	1.202263951	0.9289664	0.82347608	1.247383	0.744732022	1.158776999
843	2.14	2.15	24.7400001	11.1500002	8.36199969	sp|Q9QZD8|	Mitochondrial dicarboxylate carrier OS=Mus musculus GN=Slc25a10 PE=2 SV=2	MOUSE	1	0.53951061	0.3631931	2.6546061	0.197696999	1.432188034	0.1659587	0.14140829	27.5422897	0.018197009	4.570881844	0.96382898	0.95553112	2.26986504	0.424619585	2.187762022
844	2.14	2.15	48.2600004	40.1199997	15.7000005	sp|P63028|T(	Translationally-controlled tumor protein OS=Mus musculus GN=Tpt1 PE=1 SV=1	MOUSE	2	0.87902248	0.85022819	1.27057397	0.691830993	1.116863012	0.94623709	0.88776159	1.18032098	0.801678121	1.116863012	0.90364939	0.896824	1.43218803	0.630957425	1.29419601
845	2.14	2.14	16.9799998	8.01900029	5.18900007	sp|Q9DCZ4|A	Apolipoprotein O OS=Mus musculus GN=Apoo PE=2 SV=1	MOUSE	1	0.87096357	0.73615712	1.54170001	0.564936996	1.342764974	0.54450268	0.50832617	4.36515904	0.123594701	2.376840115	0.60255963	0.37742439	2.3550489	0.251188606	1.419057012
846	2.13	2.23	15.3099999	2.65800003	0.73330002	sp|Q61739|IT	Integrin alpha-6 OS=Mus musculus GN=Itga6 PE=1 SV=3	MOUSE	1	0.74473202	0.67668718	1.90546095	0.405508488	1.419057012	0.80909592	0.4709641	1.85353196	0.428548515	1.499685049	1.85353196	0.1596868	1.38038397	1.342764974	2.679167986
847	2.12	2.14	10.5300002	4.60500009	3.28899995	sp|Q91VI7|RI	Ribonuclease inhibitor OS=Mus musculus GN=Rnh1 PE=1 SV=1	MOUSE	1	0.96382898	0.97585708	1.18032098	0.816582382	1.137627006	0.94623709	0.98613417	1.247383	0.758577585	1.180320978	1.05681801	0.68797362	1.18032098	0.895364821	1.247382998
848	2.12	2.14	18.5100004	18.5100004	3.91499996	sp|Q8R1U2|C	Cell growth regulator with EF hand domain protein 1 OS=Mus musculus GN=Cgref1 PE=2 SV=1	MOUSE	2															
849	2.11	2.24	16.6999996	2.21999995	1.05699999	sp|P70268|PI	Serine/threonine-protein kinase N1 OS=Mus musculus GN=Pkn1 PE=1 SV=3	MOUSE	1	1.39315701	0.02251082	2.14783001	0.648634374	3.019952059	1.247383	0.05329488	1.64437199	0.758577585	2.070141077	1	0.04588789	2.14783001	0.465586096	2.147830009
850	2.1	2.12	43.3299989	28.8899998	22.2200006	sp|P09602|HI	Non-histone chromosomal protein HMG-17 OS=Mus musculus GN=Hmgn2 PE=1 SV=2	MOUSE	2	1.13762701	0.6563887	1.247383	0.912010789	1.419057012	1.09647799	0.78226262	1.23594701	0.88715601	1.367728949	1.158777	0.70290083	1.247383	0.928966403	1.445440054
851	2.1	2.11	17.7100003	2.82700006	1.48799997	sp|Q9D2R0|A	Acetoacetyl-CoA synthetase OS=Mus musculus GN=Aacs PE=1 SV=1	MOUSE	1	0.70469308	0.42174721	1.41905701	0.465586096	1	0.54450268	0.44936961	1.69044101	0.319153786	0.920449615	0.88715601	0.99983943	1.70608199	0.524807513	1.51356101
852	2.1	2.11	35.1999998	15.1999995	7.19999969	sp|P62900|RI	60S ribosomal protein L31 OS=Mus musculus GN=Rpl31 PE=2 SV=1	MOUSE	1	1.18032098	0.66073918	1.247383	0.946237087	1.458814025	1	0.94224101	1.25892496	0.794328213	1.258924961	0.99083197	0.97840762	1.247383	0.794328213	1.235947013
853	2.1	2.1	21.9699994	12.4300003	8.95999968	sp|Q920Q6|N	RNA-binding protein Musashi homolog 2 OS=Mus musculus GN=Msi2 PE=1 SV=1	MOUSE	2	0.29922649	0.04026292	27.2897797	0.02249055	8.165823936	1.07646501	0.84061432	2.0892961	0.515228629	2.249054909	0.64268768	0.38848341	2.14783001	0.293765008	1.380383968
854	2.09	2.48	19.5800006	3.36700007	1.99500006	sp|Q6A068|C	Cell division cycle 5-related protein OS=Mus musculus GN=Cdc5l PE=1 SV=2	MOUSE	1	1.36772895	0.56447041	2.18776202	0.625172675	2.964831114	1.48593605	0.57563031	20.7014103	0.07177943	22.28434944	1.599558	0.4967255	3.04789496	0.524807513	4.920394897
855	2.09	2.42	16.0699993	1.12899998	0.47590002	sp|Q9QXZ0|N	Microtubule-actin cross-linking factor 1 OS=Mus musculus GN=Macf1 PE=1 SV=2	MOUSE	2	1.11686301	0.8137359	2.0892961	0.534564376	2.333457947	2.12813902	0.30258891	2.10862804	1.009253025	4.446312904	2.91071701	0.22259191	2.10862804	1.380383968	6.08134985
856	2.09	2.14	15.5900002	3.93700004	2.36200001	sp|Q8BQP8|F	Rab11 family-interacting protein 4 OS=Mus musculus GN=Rab11fip4 PE=1 SV=1	MOUSE	1	0.09289664	0.00369321	5.54625702	0.01958845	0.515228629	0.76559663	0.50314713	3.37287307	0.216770396	2.582259893	0.1584893	0.07043582	4.83058786	0.030478951	0.765596628
857	2.09	2.11	32.5800002	21.3499993	8.98900032	sp|Q9WV98|T	Mitochondrial import inner membrane translocase subunit Tim9 OS=Mus musculus GN=Timm9 PE=1 SV=1	MOUSE	1	0.99083197	0.99912149	2.0892961	0.474242002	2.070141077	0.95499259	0.9433828	2.0892961	0.457088202	1.995262027	0.87902248	0.82001108	4.61317587	0.190546095	4.055085182
858	2.09	2.1	30.2899987	10.2200002	5.10899983	sp|Q9QZ06|T	Toll-interacting protein OS=Mus musculus GN=Tollip PE=1 SV=1	MOUSE	1	0.97274721	0.92260188	6.79203701	0.144544005	6.606935024	1.40604806	0.58879852	11.8032103	0.119124196	16.14359093	0.70469308	0.70401812	12.3594704	0.060813501	8.709635735
859	2.09	2.09	19.4900006	15.8999994	4.10300009	sp|O55022|P	Membrane-associated progesterone receptor component 1 OS=Mus musculus GN=Pgrmc1 PE=1 SV=4	MOUSE	1	1.10662401	0.94428301	3.07609701	0.359749287	3.435580015	1.58489299	0.4436622	1.38038397	1.14815402	2.187762022	1.23594701	0.9833591	4.4463129	0.277971298	5.495409012
860	2.08	4.04	33.4300011	5.71400002	5.71400002	sp|Q3UNZ8|C	Quinone oxidoreductase-like protein 2 OS=Mus musculus PE=2 SV=1	MOUSE	2	0.75857759	0.63415349	1.70608199	0.444631308	1.29419601	0.75162292	0.6784668	3.49945211	0.214782998	2.630268097	0.76559663	0.77729231	4.4463129	0.172186896	3.40408206
861	2.08	2.6	17.7100003	7.99399987	2.19400004	sp|P08003|P	Protein disulfide-isomerase A4 OS=Mus musculus GN=Pdia4 PE=1 SV=3	MOUSE	1	0.4168694	0.39761281	1.95884502	0.175388098	0.816582382	0.28054339	0.33957639	2.72897792	0.069823243	0.765596628	0.4325138	0.43801439	2.22843504	0.169044107	0.963828981
862	2.08	2.25	14.5899996	1.37599995	0.91739995	sp|P34152|F	Focal adhesion kinase 1 OS=Mus musculus GN=Ptk2 PE=1 SV=3	MOUSE	1	1.80301797	0.39965671	2.10862804	0.855066717	3.80189395	1.52756596	0.49082249	8.95364761	0.170608193	13.55189037	1.27057397	0.64464653	10.8642597	0.116949901	13.80383968
863	2.08	2.19	24.8500004	7.1010001	3.156	sp|Q3TW96|L	UDP-N-acetylhexosamine pyrophosphorylase-like protein 1 OS=Mus musculus GN=Uap1l1 PE=2 SV=1	MOUSE	4	0.86297852	0.75891089	1.247383	0.698232412	1.076465011	1.247383	0.4639945	2.07014108	0.602559626	2.606153011	1.06659603	0.65216512	2.24905491	0.474242002	2.398833036
864	2.08	2.12	10.7799999	2.46799998	1.55800004	sp|P12023|A	Amyloid beta A4 protein OS=Mus musculus GN=App PE=1 SV=3	MOUSE	1	1.47231197	0.4805305	1.61435902	0.912010789	2.376840115	1.30617094	0.58467829	1.38038397	0.946237087	1.803017974	1.75388098	0.35262409	1.38038397	1.270573974	2.421029091
865	2.08	2.08	19.3499997	4.69200015	3.66600007	sp|Q99NB1|A	Acetyl-coenzyme A synthetase 2-like, mitochondrial OS=Mus musculus GN=Acss1 PE=1 SV=1	MOUSE	1	0.60255963	0.00433877	1.72186899	0.340408206	1.037528038	0.61944109	0.00443107	2.39883304	0.248885706	1.485936046	0.564937	0.04024856	2.72897792	0.207014099	1.547000006
866	2.08	2.08	31.9200009	7.49199987	7.49199987	sp|Q8VIM4|B	Barttin OS=Mus musculus GN=Bsnd PE=1 SV=2	MOUSE	2	1.28233099	0.40389451	1.45881403	0.879022479	1.887990952	1.07646501	0.66009641	1.29419601	0.831763685	1.393157005	0.86297852	0.66543788	1.19124198	0.731139123	1.028015971
867	2.08	2.08	10.2799997	3.73799987	2.33600009	sp|Q9Z1N5|D	Spliceosome RNA helicase Ddx39b OS=Mus musculus GN=Ddx39b PE=1 SV=1	MOUSE	2	0.53456438	0.4052209	1.69044101	0.313328594	0.90364939	0.50582469	0.4078894	1.599558	0.316227794	0.809095919	0.60255963	0.44718379	1.14815402	0.519995987	0.691830993
867	0	2	15.6900004	2.34200005	2.34200005	sp|Q8VDW0|I	ATP-dependent RNA helicase DDX39A OS=Mus musculus GN=Ddx39a PE=2 SV=1	MOUSE	2															
868	2.07	2.54	20.7399994	3.5769999	2.575	sp|P41216|A	Long-chain-fatty-acid--CoA ligase 1 OS=Mus musculus GN=Acsl1 PE=1 SV=2	MOUSE	1															
869	2.07	2.19	23.8800004	2.10100003	1.62400007	sp|Q9Z0H8|C	CAP-Gly domain-containing linker protein 2 OS=Mus musculus GN=Clip2 PE=1 SV=2	MOUSE	1	1.16949904	0.67981643	2.0892961	0.55975759	2.443430901	1.22461605	0.65458143	2.0892961	0.586138189	2.558585882	1.39315701	0.54624718	7.17794323	0.194088593	10
870	2.07	2.15	10.74	1.00999996	0.68660001	sp|Q61687|A	Transcriptional regulator ATRX OS=Mus musculus GN=Atrx PE=1 SV=3	MOUSE	1	1.54170001	0.43999809	2.20800495	0.698232412	3.372873068	1.23594701	0.41103569	1.02226395	1.028015971	1.499685049	1.39315701	0.39675221	1.58489299	0.879022479	2.249054909
871	2.07	2.11	13.2100001	0.9943	0.63919998	sp|Q9ET54|P	Palladin OS=Mus musculus GN=Palld PE=1 SV=2	MOUSE	1	1.61435902	0.57761878	2.10862804	0.765596628	3.435580015	2.03235698	0.43639949	2.10862804	0.963828981	4.365159035	2.29086804	0.38648471	2.51188588	0.912010789	6.546361923
872	2.07	2.09	20.7499996	16.0400003	16.0400003	sp|Q99LY9|N	NADH dehydrogenase [ubiquinone] iron-sulfur protein 5 OS=Mus musculus GN=Ndufs5 PE=1 SV=3	MOUSE	2	0.45289761	0.2339084	1.78648806	0.233345807	0.809095919	0.8472274	0.52811492	1.49968505	0.55975759	1.270573974	0.79432821	0.51272529	1.23594701	0.636795521	0.981747925
873	2.07	2.08	14.5799995	6.94399998	5.09300008	sp|Q9DBL1|A	Short/branched chain specific acyl-CoA dehydrogenase, mitochondrial OS=Mus musculus GN=Acadsb PE=1 S	MOUSE	1	0.73790431	0.55462968	2.37684012	0.310455889	1.753880978	0.39445731	0.1453535	2.4434309	0.158489302	0.963828981	0.54450268	0.25657129	2.01372409	0.270395786	1.096477985
874	2.06	2.09	29.2600006	4.80300002	3.05700004	sp|Q9DBR0|A	A-kinase anchor protein 8 OS=Mus musculus GN=Akap8 PE=1 SV=1	MOUSE	1	2.07014108	0.34954241	5.97035313	0.346736789	12.35947037	1.43218803	0.57574242	2.18776202	0.654636085	3.133285999	1.599558	0.47012669	2.0892961	0.765596628	3.372873068
875	2.05	2.46	27.9100001	5.64800017	5.64800017	sp|P10711|T(	Transcription elongation factor A protein 1 OS=Mus musculus GN=Tcea1 PE=1 SV=2	MOUSE	2	1.20226395	0.7046231	2.0892961	0.57543987	2.511885881	1.04712904	0.89295918	2.0892961	0.501187205	2.187762022	1.07646501	0.85858208	2.10862804	0.510505021	2.249054909
876	2.05	2.07	67.4600005	35.710001	35.710001	sp|P70349|HI	Histidine triad nucleotide-binding protein 1 OS=Mus musculus GN=Hint1 PE=1 SV=3	MOUSE	4	1.18032098	0.20165309	1.18032098	1	1.406048059	1.23594701	0.1087067	1.29419601	0.954992592	1.614359021	0.80909592	0.78843361	1.34276497	0.55462569	1.08642602
877	2.05	2.06	15.0000006	5.41700013	2.91700009	sp|Q9D8Y0|E	EF-hand domain-containing protein D2 OS=Mus musculus GN=Efhd2 PE=1 SV=1	MOUSE	1	1.07646501	0.91517073	2.55858588	0.420726597	2.754229069	1.02801597	0.94250822	1.64437199	0.625172675	1.690441012	1.00925303	0.97671711	1.48593605	0.679203629	1.499685049
878	2.05	2.05	13.0899996	0.94109997	0.57279998	sp|Q9QX47|S	Protein SON OS=Mus musculus GN=Son PE=1 SV=2	MOUSE	1	1.08642602	0.85415357	2.0892961	0.519995987	2.269865036	1.51356101	0.47231469	2.0892961	0.724435985	3.191538095	0.98174793	0.97829169	2.0892961	0.469894111	2.051162004
879	2.05	2.05	16.8899998	2.53400002	2.53400002	sp|Q9CWJ9|F	Bifunctional purine biosynthesis protein PURH OS=Mus musculus GN=Atic PE=1 SV=2	MOUSE	1	1.54170001	0.4632884	2.0892961	0.73790431	3.221069098	1.03752804	0.92207438	2.0892961	0.496592313	2.167704105	1.05681801	0.89102912	2.0892961	0.505824685	2.208004951
880	2.05	2.05	23.9899993	6.41900003	4.05399986	sp|Q9D939|S	Sulfotransferase 1C2 OS=Mus musculus GN=Sult1c2 PE=1 SV=1	MOUSE	1	0.50582469	0.28507659	2.42120909	0.207014099	1.224616051	1.247383	0.30126709	1.870682	0.666806817	2.333457947	0.83946002	0.43325999	2.18776202	0.383707315	1.836537957
881	2.04	2.97	29.4200003	8.12299997	7.22000003	sp|P23953|ES	Liver carboxylesterase N OS=Mus musculus GN=Es1 PE=1 SV=4	MOUSE	5	1.61435902	0.56056541	2.0892961	0.772680581	3.40408206	1.14815402	0.87769079	13.3045397	0.086297847	14.85935974	1.54170001	0.6014089	2.10862804	0.731139123	3.221069098
882	2.04	2.11	17.6200002	5.96200004	5.96200004	sp|Q9Z2I0|LE	LETM1 and EF-hand domain-containing protein 1, mitochondrial OS=Mus musculus GN=Letm1 PE=2 SV=1	MOUSE	2	1.29419601	0.63043177	15.1356096	0.08550667	17.3780098	1.44544005	0.51732349	21.8776207	0.066069342	23.12064934	1.01859105	0.96072418	18.8799095	0.053951059	18.70681953
883	2.04	2.1	37.0200008	16.02	16.02	sp|Q3UHX2|F	28 kDa heat- and acid-stable phosphoprotein OS=Mus musculus GN=Pdap1 PE=1 SV=1	MOUSE	2	0.83946002	0.76964039	1.247383	0.672976673	1.047129035	0.75162292	0.60219711	1.64437199	0.457088202	1.235947013	0.73790431	0.59339702	1.47231197	0.496592313	1.08642602
884	2.04	2.09	16.3900003	3.27900015	3.27900015	sp|Q9WUZ9|E	Ectonucleoside triphosphate diphosphohydrolase 5 OS=Mus musculus GN=Entpd5 PE=1 SV=1	MOUSE	2	0.89536482	0.87574428	1.40604806	0.619441092	1.258924961	1.07646501	0.45804521	1.12719798	0.954992592	1.224616051	0.96382898	0.78388602	1.18032098	0.816582382	1.137627006
885	2.04	2.08	26.6799997	3.20799984	3.20799984	sp|Q8VH51|R	RNA-binding protein 39 OS=Mus musculus GN=Rbm39 PE=1 SV=2	MOUSE	2	0.76559663	0.63101709	2.0892961	0.366437614	1.599557996	1	0.98808599	2.0892961	0.478630096	2.089296103	0.79432821	0.66894889	2.0892961	0.380189389	1.659587026
886	2.04	2.08	50.6099999	19.5899993	6.93899989	sp|Q8R1G2|C	Carboxymethylenebutenolidase homolog OS=Mus musculus GN=Cmbl PE=2 SV=1	MOUSE	1	0.4875285	0.33245859	7.44731998	0.064268783	3.630779982	0.4168694	0.27586049	3.34194994	0.087902263	1.393157005	0.44463131	0.30241579	10.0925303	0.04613176	4.487453938
887	2.04	2.08	19.19	14.6500006	10.1000004	sp|Q921Z5|TI	Tumor necrosis factor alpha-induced protein 8 OS=Mus musculus GN=Tnfaip8 PE=2 SV=1	MOUSE	1															
888	2.04	2.07	7.00199977	2.17499994	1.63199995	sp|Q3TYD6|L	Serine/threonine-protein kinase LMTK2 OS=Mus musculus GN=Lmtk2 PE=1 SV=3	MOUSE	1	0.7798301	0.66194248	2.6546061	0.291071713	2.070141077	0.81658238	0.71556568	2.0892961	0.390840888	1.706081986	0.88715601	0.82318562	2.0892961	0.424619585	1.853531957
889	2.04	2.05	23.7200007	13.5000005	4.74499986	sp|Q9WU84|(	Copper chaperone for superoxide dismutase OS=Mus musculus GN=Ccs PE=1 SV=1	MOUSE	2	0.80167812	0.2482719	1.36772895	0.586138189	1.096477985	0.88715601	0.30292931	1.83653796	0.48305881	1.629295945	0.73790431	0.04471179	2.051162	0.359749287	1.51356101
890	2.04	2.04	51.1099994	32.5899988	32.5899988	sp|P63276|R	40S ribosomal protein S17 OS=Mus musculus GN=Rps17 PE=1 SV=2	MOUSE	6	3.94457293	0.54041201	2.3550489	1.67494297	9.036495209	3.49945211	0.56651127	2.4434309	1.432188034	8.394599915	3.66437602	0.54730278	2.33345795	1.570363045	8.709635735
891	2.04	2.04	71.4299977	11.2199999	11.2199999	sp|Q62426|C	Cystatin-B OS=Mus musculus GN=Cstb PE=1 SV=1	MOUSE	1	1.13762701	0.1227609	1.85353196	0.613762021	2.108628035	1.27057397	0.09136859	1.48593605	0.855066717	1.887990952	1.21338904	0.1107364	1.28233099	0.946237087	1.55596602
892	2.04	2.04	15.58	5.05300015	5.05300015	sp|P26369|U	Splicing factor U2AF 65 kDa subunit OS=Mus musculus GN=U2af2 PE=1 SV=3	MOUSE	2															
893	2.04	2.04	12.4600001	4.15199995	4.15199995	sp|O88958|G	Glucosamine-6-phosphate isomerase 1 OS=Mus musculus GN=Gnpda1 PE=2 SV=2	MOUSE	1	0.42854852	0.405018	1.47231197	0.233345807	0.630957425	0.3981072	0.36456731	1.34276497	0.258226007	0.534564376	0.32809529	0.3235065	1.83653796	0.134276494	0.602559626
894	2.04	2.04	17.7200005	10.1300001	10.1300001	sp|P61967|A	AP-1 complex subunit sigma-1A OS=Mus musculus GN=Ap1s1 PE=1 SV=1	MOUSE	1	0.61376202	0.47272259	2.10862804	0.293765008	1.29419601	0.57543987	0.38729689	6.42687702	0.089536473	3.698282003	1	0.9917143	2.0892961	0.478630096	2.089296103
895	2.03	2.3	13.6000007	3.54099982	2.83300001	sp|Q9QXS6|D	Drebrin OS=Mus musculus GN=Dbn1 PE=1 SV=4	MOUSE	1	1.36772895	0.37978491	1.58489299	0.862978518	2.22843504	1.45581403	0.33479819	1.58489299	0.920449615	2.355048895	1.75388098	0.249672	1.92309201	0.912010789	3.46736908
896	2.03	2.29	15.4400006	3.95000018	1.97500005	sp|Q9Z1M8|F	Protein Red OS=Mus musculus GN=Ik PE=2 SV=2	MOUSE	1	1.69044101	0.57868397	4.09260702	0.413047493	7.17794323	2.14783001	0.45299619	4.48745394	0.478630096	9.638290405	1.90546095	0.46765411	4.36515904	0.436515808	8.472273827
897	2.03	2.14	15.5599996	4.1960001	3.14700007	sp|P06801|M	NADP-dependent malic enzyme OS=Mus musculus GN=Me1 PE=1 SV=2	MOUSE	1	0.45289761	0.3105064	2.4434309	0.185353205	1.106624007	0.564937	0.40097889	4.78630114	0.118032098	2.703958035	0.53456438	0.37638569	2.10862804	0.2558586	1.127197981
898	2.03	2.11	37.8399998	8.10799971	8.10799971	sp|Q9DB34|C	Charged multivesicular body protein 2a OS=Mus musculus GN=Chmp2a PE=1 SV=1	MOUSE	1	0.91201079	0.6645937	2.22843504	0.40926069	2.032356977	0.9289664	0.68449581	1.38038397	0.672976673	1.28233099	0.95499259	0.7955448	1.38038397	0.691830993	1.318256974
899	2.03	2.1	18.9799994	8.52899998	2.34500002	sp|Q8BGH2|S	Sorting and assembly machinery component 50 homolog OS=Mus musculus GN=Samm50 PE=1 SV=1	MOUSE	1	0.60255963	0.616005	20.7014103	0.041686941	12.47383022	0.474242	0.4729532	2.46603894	0.185353205	1.16949904	0.2355049	0.33970991	26.0615292	0.020511162	6.137619972
900	2.03	2.09	21.9999999	2.40000002	2.40000002	sp|P47791|G	Glutathione reductase, mitochondrial OS=Mus musculus GN=Gsr PE=2 SV=3	MOUSE	1	0.82413811	0.68561888	2.18776202	0.373250186	1.803017974	0.53456438	0.30684149	21.2813892	0.039084092	11.37627029	1.38038397	0.52918869	2.10862804	0.652455285	2.884032011
901	2.03	2.08	29.3500006	4.94900011	4.94900011	sp|Q8R081|H	Heterogeneous nuclear ribonucleoprotein L OS=Mus musculus GN=Hnrnpl PE=1 SV=2	MOUSE	2															
902	2.03	2.07	28.8500011	3.46199982	3.46199982	sp|Q91VJ2|P	Protein kinase C delta-binding protein OS=Mus musculus GN=Prkcdbp PE=1 SV=1	MOUSE	1	1.00925303	0.97269863	10.7646503	0.093756199	10.86425972	0.98174793	0.98744279	16.9044094	0.058613818	16.59586906	1	0.99127603	18.8799095	0.052966349	18.70681953
903	2.03	2.06	17.5699994	1.26999999	1.26999999	sp|P16406|AI	Glutamyl aminopeptidase OS=Mus musculus GN=Enpep PE=1 SV=1	MOUSE	1	0.87902248	0.79676873	1.54170001	0.570164323	1.355188966	0.52966338	0.25606751	2.12813902	0.246603906	1.127197981	0.51522863	0.2445365	1.65958703	0.296483099	0.855066717

904	2.03	2.06	15.5300006	2.21800003	2.21800003	sp|Q9EQP2|E	EH domain-containing protein 4 OS=Mus musculus GN=Ehd4 PE=1 SV=1	MOUSE	2	0.81658238	0.71232152	2.0892961	0.390840888	1.706081986	0.80167812	0.68907422	2.10862804	0.383707315	1.690441012	1.21338904	0.69379997	2.0892961	0.580764413	2.53512907
905	2.03	2.05	15.7600001	0.60609998	0.60609998	sp|O08784|T(	Treacle protein OS=Mus musculus GN=Tcof1 PE=1 SV=1	MOUSE	1	3.133286	0.2389296	5.01187182	0.625172675	15.84893036	2.6546061	0.27429149	2.85758996	0.928966403	7.655965805	2.42102909	0.30842829	13.0617104	0.185353205	24.21029091
906	2.03	2.04	15.1600003	2.44500004	2.44500004	sp|O35657|NI	Sialidase-1 OS=Mus musculus GN=Neu1 PE=2 SV=1	MOUSE	1	0.62517268	0.72293741	4.4463129	0.140604705	2.779712915	0.75857759	0.84500402	2.80543399	0.267916799	2.128139019	0.60813499	0.64976919	3.94457293	0.151356101	2.398833036
907	2.03	2.04	22.8300005	7.30599985	7.30599985	sp|Q9D7S9|C	Charged multivesicular body protein 5 OS=Mus musculus GN=Chmp5 PE=2 SV=1	MOUSE	1															
908	2.03	2.03	19.4100007	3.84600014	3.84600014	sp|Q8R086|S	Sulfite oxidase, mitochondrial OS=Mus musculus GN=Suox PE=1 SV=2	MOUSE	1	0.87902248	0.96240801	2.80543399	0.313328594	2.466038942	1.00925303	0.92194611	2.6302681	0.383707315	2.654606104	0.80167812	0.78926003	2.03235698	0.390840888	1.629295945
909	2.03	2.03	18.0500001	1.884	1.884	sp|Q60825|NI	Sodium-dependent phosphate transport protein 2A OS=Mus musculus GN=Slc34a1 PE=1 SV=2	MOUSE	1	0.59156162	0.54710031	2.10862804	0.283139199	1.247382998	0.54954088	0.58174771	16.1435909	0.045289759	8.871560097	0.06729767	0.29501849	31.9153805	0.01406047	2.147830009
910	2.03	2.03	14.1800001	8.15600008	8.15600008	sp|Q9R0P3|E	S-formylglutathione hydrolase OS=Mus musculus GN=Esd PE=2 SV=1	MOUSE	4	0.50582469	0.30081421	8.8715601	0.057016429	4.487453938	0.34355801	0.2295679	2.0892961	0.162929595	0.717794299	0.60813499	0.35692829	22.6986504	0.039445732	13.80383968
911	2.03	2.03	25.2099991	16.8099999	16.8099999	sp|Q8R404|Q	Protein QIL1 OS=Mus musculus GN=Qil1 PE=2 SV=1	MOUSE	2	0.89536482	0.89355379	2.12813902	0.413047493	1.905460954	0.1235947	0.04037485	4.61317587	0.01106624	0.570164323	0.4487454	0.2851353	3.59749293	0.064863443	1.614359021
912	2.03	2.03	12.6100004	7.56300017	7.56300017	sp|P01887|B2	Beta-2-microglobulin OS=Mus musculus GN=B2m PE=1 SV=1	MOUSE	1	0.92044962	0.73455012	1.85353196	0.515228629	1.706081986	1.06659603	0.88209742	1.61435902	0.660693526	1.958845019	2.03235698	0.4181155	1.83653796	1.106624007	4.246195793
913	2.02	6.94	37.5499994	30.6100011	26.5300006	sp|P68254|14	14-3-3 protein theta OS=Mus musculus GN=Ywhaq PE=1 SV=1	MOUSE	11	0.51050502	0.74446392	21.6770401	0.033113111	11.06624031	0.80909592	0.84775603	2.72897792	0.283139199	2.208004951	1.30617094	0.75059629	2.6546061	0.492039502	3.597492933
914	2.02	6.52	28.459999	7.46700019	5.55000007	sp|Q80YR5|S	Scaffold attachment factor B2 OS=Mus musculus GN=Safb2 PE=1 SV=2	MOUSE	3	2.10862804	0.40397349	1.85353196	1.137627006	4.092607021	1.599558	0.54144383	1.44544005	1.106624007	2.355048895	1.38038397	0.74668819	1.80301797	0.765596628	2.53512907
915	2.02	3.15	10.1499997	2.14600004	1.46300001	sp|Q8C129|L	Leucyl-cystinyl aminopeptidase OS=Mus musculus GN=Lnpep PE=1 SV=1	MOUSE	2	1.18032098	0.70474398	6.91831017	0.170608193	8.241380692	0.2558586	0.43418989	28.0543404	0.020323571	7.17794323	1.34276497	0.58449328	8.47227383	0.158489302	11.48153973
916	2.02	2.7	15.5599996	6.91400021	4.69099991	sp|Q9WV69|D	Dematin OS=Mus musculus GN=Epb49 PE=1 SV=1	MOUSE	2	1.43218803	0.52674753	2.0892961	0.685488224	3.019952059	1.00925303	0.96140993	4.05508518	0.248885706	4.092607021	0.90364939	0.97779852	19.4088593	0.04965923	17.53881073
917	2.02	2.67	16.9599995	1.32200001	1.32200001	sp|Q8VDM4|F	26S proteasome non-ATPase regulatory subunit 2 OS=Mus musculus GN=Psmd2 PE=1 SV=1	MOUSE	2	1.25892496	0.73090142	19.0546093	0.066069342	19.95261955	1.90546095	0.3570984	2.6302681	0.724435985	5.011871815	1.72186899	0.41560301	4.7424202	0.363078088	8.241380692
918	2.02	2.22	16.9400007	9.96700004	6.31200001	sp|Q9DCN2|N	NADH-cytochrome b5 reductase 3 OS=Mus musculus GN=Cyb5r3 PE=1 SV=3	MOUSE	1	1.05681801	0.89374048	19.0546093	0.055462569	19.0546093	0.98174793	0.98961067	18.03018	0.05445027	17.70108986	1.28233099	0.61780918	16.7494297	0.076559663	18.03017998
919	2.02	2.21	15.4699996	1.28499996	0.7708	sp|A2AQ25|S	Sickle tail protein OS=Mus musculus GN=Skt PE=1 SV=1	MOUSE	1	1.33045399	0.589396	2.0892961	0.636795521	2.779712915	0.2964831	0.1995946	2.0892961	0.140604705	0.619441092	0.51522863	0.34554279	2.10862804	0.246603906	1.08642602
920	2.02	2.17	30.5900008	2.74299998	2.05799993	sp|Q3UZ39|L	Leucine-rich repeat flightless-interacting protein 1 OS=Mus musculus GN=Lrrfip1 PE=1 SV=2	MOUSE	1	1.40604806	0.59324259	1.39315701	1.009253025	1.958845019	1.65958703	0.45990121	1.61435902	1.028015971	2.779712915	1.88799095	0.3989256	2.55858588	0.73790431	4.920394897
921	2.02	2.14	9.00399983	0.40469999	0.40469999	sp|Q8BQM8|E	Echinoderm microtubule-associated protein-like 5 OS=Mus musculus GN=Eml5 PE=2 SV=2	MOUSE	1	1.09647799	0.83925778	2.0892961	0.524807513	2.290868044	1.52756596	0.4703629	2.10862804	0.724435985	3.191538095	3.43558002	0.1942344	2.10862804	1.629295945	7.17794323
922	2.02	2.13	32.3500007	1.05400002	1.05400002	sp|Q8CGK3|L	Lon protease homolog, mitochondrial OS=Mus musculus GN=Lonp1 PE=1 SV=2	MOUSE	1	0.64268768	0.4996585	9.20449543	0.069823243	5.915616989	0.78704578	0.7122196	4.0179081	0.197696999	3.162277937	0.73113912	0.63980532	6.60693502	0.110662401	4.830587864
923	2.02	2.12	23.2299998	5.23900017	4.10000011	sp|Q9CQU1|M	Microfibrillar-associated protein 1 OS=Mus musculus GN=Mfap1 PE=1 SV=1	MOUSE	1	4.61317587	0.1733039	5.19996023	0.88715601	23.55048943	4.83058786	0.15075091	5.24807501	0.920449615	26.54604912	2.6302681	0.7296834	5.24807501	0.501187205	14.06048012
924	2.02	2.11	19.4100007	1.95099991	0.97559998	sp|Q91W43|G	Glycine dehydrogenase [decarboxylating], mitochondrial OS=Mus musculus GN=Gldc PE=1 SV=1	MOUSE	1	0.89536482	0.83432198	2.0892961	0.428548515	1.870682001	1.05681801	0.89074868	2.0892961	0.505824685	2.208004951	1.22461605	0.68609768	2.0892961	0.586138189	2.558585882
925	2.02	2.11	21.9500005	2.11399999	2.11399999	sp|Q8BZR9|C	Uncharacterized protein C17orf85 homolog OS=Mus musculus PE=1 SV=1	MOUSE	1	1.31825697	0.61809838	11.6949902	0.1127197	14.99685001	0.98174793	0.90029591	7.37904215	0.133045405	7.244359016	1.34276497	0.71500909	2.0892961	0.642687678	2.83139205
926	2.02	2.1	14.9700001	1.06899999	1.06899999	sp|Q05D44|IF	Eukaryotic translation initiation factor 5B OS=Mus musculus GN=Eif5b PE=1 SV=2	MOUSE	1	1.33045399	0.62229413	1.47231197	0.90364939	1.976969957	1.09647799	0.85342783	1.70608199	0.642687678	1.887990952	1.04712904	0.84905082	1.39315701	0.751622915	1.458814025
927	2.02	2.08	16.8599993	1.85700003	1.85700003	sp|O08529|C	Calpain-2 catalytic subunit OS=Mus musculus GN=Capn2 PE=2 SV=4	MOUSE	1	0.4613176	0.30028909	2.0892961	0.218776196	0.963828981	0.67920363	0.50860781	2.0892961	0.322106898	1.419057012	0.73113912	0.57877111	2.0892961	0.349945188	1.527565956
928	2.02	2.08	49.0200013	8.82399976	8.82399976	sp|Q9DCS2|C	UPF0585 protein C16orf13 homolog OS=Mus musculus PE=1 SV=1	MOUSE	3	1.44544005	0.50807887	2.0892961	0.691830993	3.047894955	1.77010906	0.37892461	2.10862804	0.839460015	3.698282003	1.72186899	0.39255911	2.0892961	0.824138105	3.597492933
929	2.02	2.07	28.7200004	7.71299973	6.11700006	sp|Q9R0A0|P	Peroxisomal membrane protein PEX14 OS=Mus musculus GN=Pex14 PE=1 SV=1	MOUSE	1	19.4088593	0.2095827	32.5087318	0.597035289	75.85775757	17.2186909	0.1889059	32.5087318	0.529663384	73.11390686	16.2929592	0.217499	32.2106895	0.505824685	71.77942657
930	2.02	2.07	33.6600006	8.29299986	8.29299986	sp|Q61160|F	Protein FADD OS=Mus musculus GN=Fadd PE=1 SV=1	MOUSE	1															
931	2.02	2.06	29.9100012	3.5050001	3.5050001	sp|O08917|FI	Flotillin-1 OS=Mus musculus GN=Flot1 PE=1 SV=1	MOUSE	1	0.86297852	0.83657229	2.96483111	0.293765008	2.558585882	0.96382898	0.67574078	1.97696996	0.492039502	1.905460954	1.14815402	0.69770432	1.57036305	0.731139123	1.803017974
932	2.02	2.05	12.4499999	1.688	1.688	sp|P16675|PI	Lysosomal protective protein OS=Mus musculus GN=Ctsa PE=1 SV=1	MOUSE	1	0.65463609	0.68253022	1.39315701	0.457088202	0.912010789	0.58076441	0.77236408	1.94088602	0.293765008	1.127197981	0.96382898	0.97944802	1.28233099	0.744732022	1.235947013
933	2.02	2.03	38.440001	4.42200005	4.42200005	sp|O08600|NI	Endonuclease G, mitochondrial OS=Mus musculus GN=Endog PE=2 SV=1	MOUSE	1	0.75162292	0.60809898	2.10862804	0.359749287	1.584892988	0.62517268	0.44847539	3.94457293	0.158489302	2.466038942	0.55462569	0.3728829	18.03018	0.04325138	10
934	2.02	2.03	36.1099988	4.16699983	4.16699983	sp|Q9CXN7|F	Phenazine biosynthesis-like domain-containing protein 2 OS=Mus musculus GN=Pbld2 PE=2 SV=1	MOUSE	1	0.78704578	0.65739721	2.0892961	0.373250186	1.644371986	0.82413811	0.72372532	2.0892961	0.39445731	1.721868992	0.51522863	0.34520111	2.10862804	0.246603906	1.08642602
934	0	2.02	28.8199991	4.16699983	4.16699983	sp|Q9DCG6|F	Phenazine biosynthesis-like domain-containing protein 1 OS=Mus musculus GN=Pbld1 PE=2 SV=2	MOUSE	1															
935	2.02	2.03	20.4799995	6.50599971	6.50599971	sp|Q9D7N9|A	Adipocyte plasma membrane-associated protein OS=Mus musculus GN=Apmap PE=1 SV=1	MOUSE	1															
936	2.02	2.03	10.1599999	1.15499999	1.15499999	sp|Q4KMM3|(	Oxidation resistance protein 1 OS=Mus musculus GN=Oxr1 PE=1 SV=3	MOUSE	1	1.40604806	0.54396141	20.5116196	0.068548821	21.8776207	2.16770411	0.31955329	7.24435902	0.299226493	15.84893036	2.0892961	0.3194353	4.16869402	0.501187205	8.629785538
937	2.02	2.03	17.3199996	2.85100006	2.85100006	sp|Q9D8W5|F	26S proteasome non-ATPase regulatory subunit 12 OS=Mus musculus GN=Psmd12 PE=2 SV=4	MOUSE	1															
938	2.02	2.03	28.5699993	6.28599972	6.28599972	sp|P23927|CI	Alpha-crystallin B chain OS=Mus musculus GN=Cryab PE=1 SV=2	MOUSE	1	1.33045399	0.88414568	2.18776202	0.608134985	9.289664268	2.22843504	0.5846833	3.31131101	0.672976673	14.58813953	5.29663515	0.26949909	4.28548479	1.235947013	30.4789505
939	2.02	2.02	14.3600002	3.72299999	3.72299999	sp|Q62523|Z	Zyxin OS=Mus musculus GN=Zyx PE=1 SV=2	MOUSE	4	1.38038397	0.55507678	2.0892961	0.660693526	2.884032011	1.25892496	0.64886302	2.0892961	0.602559626	2.630268097	1.23594701	0.67644298	2.0892961	0.591561615	2.582259893
940	2.02	2.02	34.2999995	16.4299995	6.76300004	sp|P51150|R	Ras-related protein Rab-7a OS=Mus musculus GN=Rab7a PE=1 SV=2	MOUSE	3	1.41905701	0.50298202	2.58225989	0.549540877	3.630779982	1.247383	0.64045048	2.18776202	0.570164323	2.728977919	1.870682	0.32713789	2.0892961	0.895364821	3.908409119
941	2.02	2.02	23.7800002	7.92699978	7.92699978	sp|Q9D967|M	Magnesium-dependent phosphatase 1 OS=Mus musculus GN=Mdp1 PE=1 SV=1	MOUSE	1	0.42854852	0.27784881	2.0892961	0.203235701	0.895364821	1.10662401	0.82856208	2.0892961	0.529663384	2.312064886	0.55462569	0.37856171	2.0892961	0.26546061	1.158776999
942	2.02	2.02	8.59199986	2.14799997	2.14799997	sp|O09043|N	Napsin-A OS=Mus musculus GN=Napsa PE=1 SV=1	MOUSE	1	0.67297667	0.7518245	1.80301797	0.359749287	1.213389039	0.4786301	0.54462957	1.85353196	0.208929598	0.88715601	0.59156162	0.67191738	1.80301797	0.310455889	1.066596031
943	2.01	2.74	14.6899998	3.125	3.125	sp|Q8BH04|P	Phosphoenolpyruvate carboxykinase [GTP], mitochondrial OS=Mus musculus GN=Pck2 PE=2 SV=1	MOUSE	2	0.83176369	0.71140409	2.10862804	0.398107201	1.753880978	0.92044962	0.86027199	2.96483111	0.307609707	2.728977919	1.25892496	0.64447838	18.03018	0.069823243	18.87990952
944	2.01	2.27	4.96199988	0.333	0.19980001	sp|A2AWL7|M	MAX gene-associated protein OS=Mus musculus GN=Mga PE=2 SV=1	MOUSE	1															
944	0.01	2.02	5.51600009	0.84870001	0.84870001	RRRRRsp|Q(	REVERSED Caprin-1 OS=Mus musculus GN=Caprin1 PE=1 SV=2	MOUSE	1															
944	0.01	2.01	5.34900017	0.89149997	0.89149997	RRRRRsp|Q8	REVERSED NACHT, LRR and PYD domains-containing protein 10 OS=Mus musculus GN=Nlrp10 PE=1 SV=1	MOUSE	1															
945	2.01	2.17	21.4300007	3.71400006	2.28599999	sp|A2AHG0|F	ProSAP-interacting protein 1 OS=Mus musculus GN=Prosapip1 PE=2 SV=1	MOUSE	1															
946	2.01	2.14	18.9400002	11.0100001	8.81099999	sp|P58044|ID	Isopentenyl-diphosphate Delta-isomerase 1 OS=Mus musculus GN=Idi1 PE=2 SV=1	MOUSE	1															
947	2.01	2.14	29.8700005	18.1799993	14.2900005	sp|P68037|UI	Ubiquitin-conjugating enzyme E2 L3 OS=Mus musculus GN=Ube2l3 PE=2 SV=1	MOUSE	1															
948	2.01	2.12	26.7100006	4.76199985	4.76199985	sp|Q9DCD0|6	6-phosphogluconate dehydrogenase, decarboxylating OS=Mus musculus GN=Pgd PE=2 SV=3	MOUSE	1															
949	2.01	2.1	25.2499998	0.98679997	0.98679997	sp|Q148V7|K	LisH domain and HEAT repeat-containing protein KIAA1468 OS=Mus musculus GN=Kiaa1468 PE=1 SV=1	MOUSE	1	1.14815402	0.79864693	3.90840912	0.293765008	4.487453938	0.99083197	0.96681762	18.8799095	0.05248075	18.70681953	1.08642602	0.87115628	2.0892961	0.519995987	2.269865036
950	2.01	2.1	23.7200007	4.8080001	2.88500004	sp|Q64012|R	RNA-binding protein Raly OS=Mus musculus GN=Raly PE=1 SV=3	MOUSE	1	1.10662401	0.58324969	1.47231197	0.751622915	1.629295945	0.98174793	0.68389452	2.37684012	0.413047493	2.333457947	1.04712904	0.79563099	1.38038397	0.758577585	1.445440054
951	2.01	2.08	12.6000002	2.27300003	2.27300003	sp|P54822|PI	Adenylosuccinate lyase OS=Mus musculus GN=Adsl PE=2 SV=2	MOUSE	1	0.92044962	0.88803321	2.0892961	0.440554887	1.923092008	0.7798301	0.6509828	2.0892961	0.373250186	1.629295945	1.11686301	0.807064	2.0892961	0.534564376	2.333457947
952	2.01	2.07	23.9500001	5.90200014	5.90200014	sp|Q8BTW9|F	Serine/threonine-protein kinase PAK 4 OS=Mus musculus GN=Pak4 PE=1 SV=1	MOUSE	2	0.9289664	0.9013629	2.0892961	0.444631308	1.940886021	0.98174793	0.98947138	6.98232412	0.140604705	6.854881763	0.83946002	0.7474401	2.0892961	0.401790798	1.753880978
953	2.01	2.07	22.0200002	7.51300007	5.95900007	sp|P51661|DI	Corticosteroid 11-beta-dehydrogenase isozyme 2 OS=Mus musculus GN=Hsd11b2 PE=2 SV=2	MOUSE	1	0.43651581	0.28321371	2.0892961	0.207014099	0.912010789	0.4168694	0.26921469	2.0892961	0.197696999	0.870963573	0.54954088	0.3713437	2.0892961	0.260615289	1.14815402
954	2.01	2.07	11.5500003	2.30999999	2.30999999	sp|Q791V5|M	Mitochondrial carrier homolog 2 OS=Mus musculus GN=Mtch2 PE=1 SV=1	MOUSE	1	0.34355801	0.27247629	4.40554905	0.077268057	1.51356101	0.34040821	0.27102462	2.10862804	0.161435902	0.717794299	0.1148154	0.2127904	32.5087318	0.01614359	3.732501984
955	2.01	2.06	22.1699998	3.77399996	3.77399996	sp|Q99JB8|P	Protein kinase C and casein kinase II substrate protein 3 OS=Mus musculus GN=Pacsin3 PE=1 SV=1	MOUSE	1	1.03752804	0.97382611	1.47231197	0.704693079	1.527565956	0.99083197	0.99026603	1.38038397	0.717794299	1.367728949	1.25892496	0.60963279	1.52756596	0.824138105	1.923092008
956	2.01	2.05	24.6299997	1.15099996	1.15099996	sp|Q9DBT9|M	Dimethylglycine dehydrogenase, mitochondrial OS=Mus musculus GN=Dmgdh PE=1 SV=1	MOUSE	1	0.57543987	0.4621923	1.48593605	0.387257606	0.855066717	0.50118721	0.4468919	2.37684012	0.208929598	1.19124198	0.72443599	0.67541772	1.44544005	0.492039502	1.047129035
957	2.01	2.04	15.4799998	1.63000003	1.63000003	sp|Q8VDM6|H	Heterogeneous nuclear ribonucleoprotein U-like protein 1 OS=Mus musculus GN=Hnrnpul1 PE=1 SV=1	MOUSE	2	1.23594701	0.6607675	1.20226395	1.028015971	1.499685049	1.25892496	0.59641051	1.54170001	0.816582382	1.940886021	0.91201079	0.99888951	1.73780096	0.519995987	1.584892988
958	2.01	2.04	13.1099999	2.049	2.049	sp|Q8R146|A	Acylamino-acid-releasing enzyme OS=Mus musculus GN=Apeh PE=2 SV=2	MOUSE	1	0.75162292	0.60735989	2.10862804	0.359749287	1.584892988	0.65463609	0.48749021	2.0892961	0.313328594	1.367728949	0.71121353	0.5466069	2.0892961	0.340408206	1.485936046
959	2.01	2.04	31.979999	9.45900008	9.45900008	sp|Q9CR59|G	Growth arrest and DNA damage-inducible proteins-interacting protein 1 OS=Mus musculus GN=Gadd45gip1 P	MOUSE	1	1.158777	0.75581592	2.0892961	0.55462569	2.421029091	1.13762701	0.78243178	2.0892961	0.544502676	2.376840115	1.34276497	0.57808697	2.0892961	0.642687678	2.83139205
960	2.01	2.04	24.2799997	8.67099986	8.67099986	sp|P10107|AI	Annexin A1 OS=Mus musculus GN=Anxa1 PE=1 SV=2	MOUSE	2	1.69044101	0.40098581	2.0892961	0.809095919	3.564511061	0.90364939	0.84877157	2.0892961	0.428548515	1.887990952	2.6302681	0.2417423	2.0892961	1.258924961	5.546257019
961	2.01	2.03	32.5800002	3.96599993	3.96599993	sp|Q99JB2|S	Stomatin-like protein 2 OS=Mus musculus GN=Stoml2 PE=1 SV=1	MOUSE	1	1.19124198	0.86388892	16.4437199	0.072443597	17.06081963	1.22461605	0.77838588	5.59757614	0.218776196	6.854881763	1.21338904	0.79155719	5.39510584	0.224905506	6.546361923
962	2.01	2.03	18.6199993	1.67599991	1.67599991	sp|P13597|IC	Intercellular adhesion molecule 1 OS=Mus musculus GN=Icam1 PE=1 SV=1	MOUSE	1	1.48593605	0.40528831	2.0892961	0.711213529	3.104559898	1.44544005	0.46121791	3.63077998	0.398107201	5.199960232	1.88799095	0.29887101	5.64937019	0.334194988	10.66596031
963	2.01	2.03	17.1800003	4.90799993	4.90799993	sp|Q9CSU0|F	Regulation of nuclear pre-mRNA domain-containing protein 1B OS=Mus musculus GN=Rprd1b PE=2 SV=2	MOUSE	1	1.07646501	0.93510008	2.22843504	0.48305881	2.398833036	1.07646501	0.94121468	2.29086804	0.469894111	2.466038942	0.99083197	0.71004307	8.39459992	0.118032098	8.317637444
964	2.01	2.03	10.8999997	3.52599993	3.52599993	sp|Q8R3P0|A	Aspartoacylase OS=Mus musculus GN=Aspa PE=1 SV=2	MOUSE	1	0.39084089	0.41553691	21.2813892	0.032210689	8.317637444	0.3981072	0.40344599	15.1356096	0.035974931	6.025596142	0.54954088	0.53967631	11.2719698	0.053456441	6.194410801
965	2.01	2.03	20.4699993	8.66099969	8.66099969	sp|P11031|T(	Activated RNA polymerase II transcriptional coactivator p15 OS=Mus musculus GN=Sub1 PE=1 SV=3	MOUSE	1	0.88715601	0.88874137	3.25087309	0.275422901	2.884032011	0.93756199	0.91383332	2.0892961	0.4487454	1.958845019	0.7177943	0.61558199	4.4463129	0.161435902	3.191538095
966	2.01	2.02	23.2199997	2.25300007	2.25300007	sp|Q9JLV1|B	BAG family molecular chaperone regulator 3 OS=Mus musculus GN=Bag3 PE=1 SV=2	MOUSE	1	1.22461605	0.73755151	1.38038397	0.88715601	1.690441012	1.40604806	0.61408782	2.20800495	0.636795521	3.133285999	1.31825697	0.56591159	4.4463129	0.296483099	5.861382008
967	2.01	2.02	18.1099996	3.34299989	3.34299989	sp|P11247|PI	Myeloperoxidase OS=Mus musculus GN=Mpo PE=2 SV=2	MOUSE	1	1.54170001	0.46346599	2.10862804	0.731139123	3.221069098	1.599558	0.43822321	2.10862804	0.758577585	3.34194994	2.91071701	0.221816	2.0892961	1.393157005	6.08134985
968	2.01	2.02	14.4299999	5.57399988	5.57399988	sp|Q08093|CI	Calponin-2 OS=Mus musculus GN=Cnn2 PE=2 SV=1	MOUSE	1															
969	2.01	2.02	33.5299999	11.7600001	11.7600001	sp|Q9QY93|D	dCTP pyrophosphatase 1 OS=Mus musculus GN=Dctpp1 PE=1 SV=1	MOUSE	1															
970	2.01	2.02	10.65	2.66200006	2.66200006	sp|Q63850|NI	Nuclear pore glycoprotein p62 OS=Mus musculus GN=Nup62 PE=1 SV=2	MOUSE	1															
971	2.01	2.01	24.0799993	2.05700006	0.94259996	sp|Q9Z0U1|Z	Tight junction protein ZO-2 OS=Mus musculus GN=Tjp2 PE=1 SV=2	MOUSE	1	0.96382898	0.9450255	1.25892496	0.765596628	1.213389039	1.01859105	0.78186381	2.16770411	0.469894111	2.208004951	1.20226395	0.6612044	1.58489299	0.758577585	1.905460954
972	2.01	2.01	43.3299989	5.23799993	5.23799993	sp|Q91YN9|B	BAG family molecular chaperone regulator 2 OS=Mus musculus GN=Bag2 PE=1 SV=1	MOUSE	1	0.4570882	0.29710391	2.0892961	0.216770396	0.954992592	1.31825697	0.59620929	2.0892961	0.630957425	2.754229069	1.70608199	0.3964076	2.0892961	0.816582382	3.597492933
973	2.01	2.01	13.6500001	4.01600003	4.01600003	sp|Q8C4V1|R	Rho GTPase-activating protein 24 OS=Mus musculus GN=Arhgap24 PE=2 SV=2	MOUSE	2															
974	2.01	2.01	10.2499999	1.16699999	1.16699999	sp|O70570|PI	Polymeric immunoglobulin receptor OS=Mus musculus GN=Pigr PE=1 SV=1	MOUSE	1	0.53951061	0.36344361	2.0892961	0.258226007	1.127197981	1.03752804	0.92699403	2.0892961	0.496592313	2.167704105	0.83946002	0.74372071	2.0892961	0.401790798	1.753880978
975	2.01	2.01	27.1800011	14.5600006	7.76700005	sp|Q9Z2Q5|R	39S ribosomal protein L40, mitochondrial OS=Mus musculus GN=Mrpl40 PE=2 SV=2	MOUSE	1															
976	2.01	2.01	20.2399999	4.85799983	4.85799983	sp|Q9CR67|T	Transmembrane protein 33 OS=Mus musculus GN=Tmem33 PE=2 SV=1	MOUSE	1	0.37325019	0.242567	2.0892961	0.177010894	0.779830098	0.564937	0.3867653	2.0892961	0.270395786	1.180320978	0.57016432	0.39241901	2.0892961	0.27289781	1.19124198
977	2.01	2.01	54.3900013	8.77199993	8.77199993	sp|Q9JJU8|SI	SH3 domain-binding glutamic acid-rich-like protein OS=Mus musculus GN=Sh3bgrl PE=2 SV=1	MOUSE	1	1.247383	0.37891391	1.28233099	0.972747207	1.599557996	1.39315701	0.38682151	1.88799095	0.73790431	2.654606104	1.30617094	0.3789041	1.44544005	0.90364939	1.887990952
978	2.01	2.01	17.4199998	3.87100019	3.87100019	sp|P31230|AI	Aminoacyl tRNA synthase complex-interacting multifunctional protein 1 OS=Mus musculus GN=Aimp1 PE=1 S'	MOUSE	1	1.36772895	0.54597682	18.7068195	0.073113911	19.95261955	1.20226395	0.68034208	2.0892961	0.57543987	2.53512907	1	0.99168539	8.55066681	0.116949901	8.550666809
979	2.01	2.01	27.9199988	23.3799994	11.6899997	sp|Q9WU28|F	Prefoldin subunit 5 OS=Mus musculus GN=Pfdn5 PE=1 SV=1	MOUSE	1															
980	2.01	2.01	19.1599995	3.44800018	3.44800018	sp|P15947|KI	Kallikrein-1 OS=Mus musculus GN=Klk1 PE=1 SV=3	MOUSE	1	0.89536482	0.82193768	1.48593605	0.619441092	1.330453992	1.61435902	0.60895813	1.65958703	0.972747207	2.884032011	2.03235698	0.41253811	1.55596602	1.30617094	3.630779982

981	2.01	2.01	15.7100007	8.90100002	8.90100002	sp|P60766|C	Cell division control protein 42 homolog OS=Mus musculus GN=Cdc42 PE=1 SV=2	MOUSE	1																
982	2	32.19	84.6199989	63.8499975	56.9199979	sp|Q8CGP7|H	Histone H2A type 1-K OS=Mus musculus GN=Hist1h2ak PE=1 SV=3	MOUSE	27																
983	2	28.1	45.9500015	37.1600002	31.979999	sp|P99024|TE	Tubulin beta-5 chain OS=Mus musculus GN=Tubb5 PE=1 SV=1	MOUSE	36	1.158777	0.75567257	2.0892961	0.55462569	2.421029091	1.78648806	0.37240309	2.0892961	0.855066717	3.767038107	1.55596602	0.45204991	2.0892961	0.744732022	3.28095293	
984	2	18.15	77.7800024	62.6999974	46.0299999	sp|Q8CGP0|H	Histone H2B type 3-B OS=Mus musculus GN=Hist3h2bb PE=1 SV=3	MOUSE	24																
984	0	17.48	69.0500021	54.7599971	46.0299999	sp|Q9D2U9|H	Histone H2B type 3-A OS=Mus musculus GN=Hist3h2ba PE=1 SV=3	MOUSE	24																
985	2	6.1	54.339999	11.4100002	9.67700034	sp|P19001|K	Keratin, type I cytoskeletal 19 OS=Mus musculus GN=Krt19 PE=2 SV=1	MOUSE	3																
986	2	5.43	42.1900004	20.3099996	20.3099996	sp|Q3THW5|H	Histone H2A.V OS=Mus musculus GN=H2afv PE=1 SV=3	MOUSE	6	0.9289664	0.89613748	2.0892961	0.444631308	1.940886021	0.91201079	0.86338711	2.0892961	0.436515808	1.905460954	1.05681801	0.9006635	2.0892961	0.505824685	2.208004951	
986	0	5.43	42.1900004	20.3099996	20.3099996	sp|P0C0S6|H	Histone H2A.Z OS=Mus musculus GN=H2afz PE=1 SV=2	MOUSE	6																
987	2	5.34	53.1899989	15.2400002	12.19	sp|Q9JKB3|D	DNA-binding protein A OS=Mus musculus GN=Csda PE=1 SV=2	MOUSE	4	0.73790431	0.9162333	13.5518904	0.058613818	10	1.33045399	0.72276819	2.20800495	0.602559626	3.019952059	2.03235698	0.4437139	7.31139088	0.277971298	15.13560963	
988	2	4.04	28.9900005	11.1100003	11.1100003	sp|P07361|A	Alpha-1-acid glycoprotein 2 OS=Mus musculus GN=Orm2 PE=1 SV=1	MOUSE	2																
989	2	4.01	21.7700005	15.6499997	15.6499997	sp|P04443|H	Hemoglobin subunit beta-H0 OS=Mus musculus GN=Hbb-bh0 PE=2 SV=5	MOUSE	2																
990	2	4	19.8300004	19.8300004	19.8300004	sp|P63044|V	Vesicle-associated membrane protein 2 OS=Mus musculus GN=Vamp2 PE=1 SV=2	MOUSE	2	1.48593605	0.4920741	2.10862804	0.704693079	3.104559898	1.57036305	0.44815171	2.0892961	0.751622915	3.28095293	1.30617094	0.60818988	2.0892961	0.625172675	2.728977919	
991	2	3.58	50.2799988	17.8800002	17.8800002	sp|Q505D7|O	Optic atrophy 3 protein homolog OS=Mus musculus GN=Opa3 PE=1 SV=1	MOUSE	4																
992	2	3.21	18.6299995	5.79699986	5.79699986	sp|Q8BVE3|V	V-type proton ATPase subunit H OS=Mus musculus GN=Atp6v1h PE=1 SV=1	MOUSE	3	0.67920363	0.51364708	2.10862804	0.325087309	1.432188034	0.36643761	0.2395497	2.10862804	0.175388098	0.772680581	0.75857759	0.62087238	2.0892961	0.363078088	1.584892988	
993	2	3.05	18.5800001	5.57300001	5.57300001	sp|O35639|A	Annexin A3 OS=Mus musculus GN=Anxa3 PE=1 SV=4	MOUSE	2	1.97696996	0.90897721	3.04789496	0.648634374	11.48153973	1.35518897	0.88141233	2.29086804	0.591561615	5.248075008	9.63829041	0.20693959	3.46736908	2.779712915	36.9828186	
994	2	2.91	25.1800001	2.56699994	2.56699994	sp|Q9D3S3|S	Sorting nexin-29 OS=Mus musculus GN=Snx29 PE=1 SV=2	MOUSE	2	1.28233099	0.63861132	2.10862804	0.608134985	2.679167986	1.33045399	0.63157171	2.67916799	0.496592313	3.564511061	1	0.93991309	7.24435902	0.138038397	7.244359016	
995	2	2.82	10.0900002	5.84100001	4.95599993	sp|Q8VCC2|E	Liver carboxylesterase 1 OS=Mus musculus GN=Ces1 PE=2 SV=1	MOUSE	5	0.67920363	0.56209153	10.5681801	0.064268783	7.17794323	0.85506672	0.81872922	3.28095293	0.260615289	2.805433989	0.89536482	0.86237532	2.0892961	0.428548515	1.870682001	
996	2	2.77	22.0799997	4.56900001	4.56900001	sp|P61161|A	Actin-related protein 2 OS=Mus musculus GN=Actr2 PE=1 SV=1	MOUSE	2	0.80167812	0.74836558	4.09260702	0.195884496	3.28095293	0.91201079	0.91438359	2.0892961	0.436515808	1.905460954	0.97274721	0.98315418	2.0892961	0.465586096	2.032356977	
997	2	2.47	8.81400034	2.48600002	1.13000004	sp|Q60953|P	Protein PML OS=Mus musculus GN=Pml PE=1 SV=3	MOUSE	1	1.12719798	0.79192579	2.0892961	0.539510608	2.376840115	0.87902248	0.80826968	2.0892961	0.420726597	1.836537957	1.16949904	0.74469483	2.0892961	0.55975759	2.443430901	
998	2	2.18	24.6600002	8.0430001	6.43400028	sp|Q61767|3	3 beta-hydroxysteroid dehydrogenase type 4 OS=Mus musculus GN=Hsd3b4 PE=2 SV=3	MOUSE	1																
998	0	2.02	25.2000004	6.43400028	6.43400028	sp|Q61694|3	3 beta-hydroxysteroid dehydrogenase type 5 OS=Mus musculus GN=Hsd3b5 PE=1 SV=4	MOUSE	1																
999	2	2.15	43.2599992	9.30199996	6.51199967	sp|Q91V41|R	Ras-related protein Rab-14 OS=Mus musculus GN=Rab14 PE=1 SV=3	MOUSE	1	1.23594701	0.66870022	2.0892961	0.591561615	2.606153011	1.65958703	0.4123224	2.0892961	0.794328213	3.499452114	1.55596602	0.45646581	2.10862804	0.73790431	3.250873089	
1000	2	2.13	17.2099993	1.56399999	1.56399999	sp|Q9Z1Z0|U	General vesicular transport factor p115 OS=Mus musculus GN=Uso1 PE=1 SV=2	MOUSE	1																
1001	2	2.1	21.0999996	1.26599995	1.26599995	sp|Q0P678|Z	Zinc finger CCCH domain-containing protein 18 OS=Mus musculus GN=Zc3h18 PE=1 SV=1	MOUSE	1	0.89536482	0.72138691	1.83653796	0.487528503	1.644371986	1.11686301	0.89160299	2.3550489	0.474242002	2.654606104	0.80167812	0.6267758	1.43218803	0.55462569	1.14815402	
1002	2	2.1	8.26999992	1.33100003	0.85549997	sp|Q8C5W0|C	Calmin OS=Mus musculus GN=Clmn PE=1 SV=2	MOUSE	1	3.25087309	0.43500441	8.79022503	0.369828194	27.03957939	3.49945211	0.4104695	19.2309208	0.181970105	35.64511108	3.2210691	0.4123843	29.1071701	0.110662401	36.64376068	
1003	2	2.1	16.9699997	7.01100007	7.01100007	sp|P56402|A	Aquaporin-2 OS=Mus musculus GN=Aqp2 PE=2 SV=2	MOUSE	2	0.80909592	0.65792882	2.16770411	0.373250186	1.753880978	0.57016432	0.37374729	2.10862804	0.27289781	1.202263951	0.79432821	0.65542519	2.0892961	0.376703799	1.659587026	
1004	2	2.09	17.3999995	1.25000002	1.25000002	sp|Q7TSG2|C	RNA polymerase II subunit A C-terminal domain phosphatase OS=Mus musculus GN=Ctdp1 PE=1 SV=1	MOUSE	1	1.34276497	0.57870358	1.97696996	0.679203629	2.654606104	1.49968505	0.44454861	1.57036305	0.954992592	2.355048895	1.39315701	0.52979612	1.70608199	0.816582382	2.376840115	
1005	2	2.09	17.7900001	4.34800014	4.34800014	sp|Q8VCH8|U	UBX domain-containing protein 4 OS=Mus musculus GN=Ubxn4 PE=1 SV=1	MOUSE	1																
1006	2	2.09	23.9099994	3.91299985	2.82600001	sp|P33587|P	Vitamin K-dependent protein C OS=Mus musculus GN=Proc PE=1 SV=2	MOUSE	1	1	0.99214298	2.0892961	0.478630096	2.089296103	0.94623709	0.92406392	2.0892961	0.452897608	1.976969957	2.07014108	0.30944911	2.0892961	0.990831971	4.365159035	
1007	2	2.08	14.0100002	0.94560003	0.94560003	sp|Q6PB44|P	Tyrosine-protein phosphatase non-receptor type 23 OS=Mus musculus GN=Ptpn23 PE=1 SV=2	MOUSE	1	1.48593605	0.51848239	2.0892961	0.711213529	3.104559898	1.52756596	0.4932344	2.0892961	0.731139123	3.221069098	2.20800495	0.30545461	2.3550489	0.937561989	5.199960232	
1008	2	2.08	20.6200004	6.45800009	5.41700013	sp|Q9Z1Y4|T	Thyroid receptor-interacting protein 6 OS=Mus musculus GN=Trip6 PE=1 SV=1	MOUSE	1	0.87902248	0.80935383	2.0892961	0.420726597	1.836537957	0.98174793	0.98608297	2.0892961	0.469894111	2.051162004	0.7798301	0.65386802	2.0892961	0.373250186	1.629295945	
1009	2	2.08	13.4900004	2.79900003	2.79900003	sp|P31938|M	Dual specificity mitogen-activated protein kinase kinase 1 OS=Mus musculus GN=Map2k1 PE=1 SV=2	MOUSE	1	1.72186899	0.46082851	7.24435902	0.237683997	12.47383022	1.99526203	0.3757968	12.9419603	0.154170096	21.47830009	2.26986504	0.3231723	14.8593597	0.152756602	24.43431091	
1010	2	2.07	18.4100002	2.09599994	2.09599994	sp|O08582|G	GTP-binding protein 1 OS=Mus musculus GN=Gtpbp1 PE=1 SV=2	MOUSE	1																
1011	2	2.07	10.9999999	5.37899993	4.15600017	sp|Q9JLZ8|S	Single Ig IL-1-related receptor OS=Mus musculus GN=Sigirr PE=1 SV=2	MOUSE	1	0.78704578	0.66694468	4.6558609	0.169044107	3.66437602	0.98174793	0.99067181	2.0892961	0.469894111	2.051162004	1.11686301	0.80916673	2.0892961	0.534564376	2.333457947	
1012	2	2.07	51.3300002	30.9700012	26.5500009	sp|P48024|E	Eukaryotic translation initiation factor 1 OS=Mus musculus GN=Eif1 PE=2 SV=2	MOUSE	2	2.6302681	0.2434845	2.10862804	1.247382998	5.495409012	2.10862804	0.30572552	2.10862804	1	4.405549049	2.77971292	0.2312976	2.10862804	1.318256974	5.80764389	
1012	0	2	52.2099972	26.5500009	26.5500009	sp|Q9CXU9|E	Eukaryotic translation initiation factor 1b OS=Mus musculus GN=Eif1b PE=2 SV=2	MOUSE	2																
1013	2	2.06	14.3999994	2.19999999	2.19999999	sp|P61922|G	4-aminobutyrate aminotransferase, mitochondrial OS=Mus musculus GN=Abat PE=1 SV=1	MOUSE	1	0.86297852	0.78367078	2.0892961	0.413047493	1.803017974	0.69183099	0.53105837	2.10862804	0.331131101	1.458814025	0.80167812	0.6883893	2.0892961	0.383707315	1.67494297	
1014	2	2.06	22.9699999	7.06700012	7.06700012	sp|Q9EQI8|R	39S ribosomal protein L46, mitochondrial OS=Mus musculus GN=Mrpl46 PE=2 SV=1	MOUSE	1																
1015	2	2.05	21.6000006	2.81700008	2.81700008	sp|Q3TIV5|Z	Zinc finger CCCH domain-containing protein 15 OS=Mus musculus GN=Zc3h15 PE=1 SV=2	MOUSE	1	1.62929595	0.42498949	2.0892961	0.779830098	3.40408206	1.55596602	0.45802629	2.10862804	0.73790431	3.250873089	1.57036305	0.44599381	2.0892961	0.751622915	3.311311007	
1016	2	2.05	18.6000004	2.47900002	2.47900002	sp|P47740|A	Fatty aldehyde dehydrogenase OS=Mus musculus GN=Aldh3a2 PE=2 SV=2	MOUSE	1	0.75857759	0.61244351	1.34276497	0.55975759	1.018591046	0.69823241	0.58102369	1.57036305	0.444631308	1.096477985	0.87096357	0.78043401	1.247383	0.698232412	1.08642602	
1017	2	2.05	9.516	2.18400005	2.18400005	sp|Q923D5|W	WW domain-binding protein 11 OS=Mus musculus GN=Wbp11 PE=1 SV=2	MOUSE	1	1	0.98211378	2.0892961	0.478630096	2.089296103	0.87902248	0.81290329	2.0892961	0.420726597	1.836537957	0.64268768	0.47137049	2.10862804	0.307609707	1.355188966	
1018	2	2.05	18.2099998	1.68600008	1.68600008	sp|Q64324|S	Syntaxin-binding protein 2 OS=Mus musculus GN=Stxbp2 PE=2 SV=1	MOUSE	1	1.30617094	0.60532951	2.0892961	0.625172675	2.754229069	1.55596602	0.45513809	2.10862804	0.73790431	3.250873089	1.599558	0.43467301	2.0892961	0.765596628	3.372873068	
1019	2	2.05	8.08599964	1.64999999	1.64999999	sp|O88342|W	WD repeat-containing protein 1 OS=Mus musculus GN=Wdr1 PE=1 SV=3	MOUSE	1	0.64268768	0.53758389	4.16869402	0.094623707	2.679167986	0.1270574	0.45242721	36.6437607	0.01674943	4.655860901	0.69183099	0.88619179	18.03018	0.046989411	12.47383022	
1020	2	2.05	34.7000003	7.30599985	7.30599985	sp|Q9D1D4|T	Transmembrane emp24 domain-containing protein 10 OS=Mus musculus GN=Tmed10 PE=2 SV=1	MOUSE	2																
1021	2	2.05	14.26	4.4780001	4.4780001	sp|Q6P5E6|G	ADP-ribosylation factor-binding protein GGA2 OS=Mus musculus GN=Gga2 PE=1 SV=1	MOUSE	1																
1022	2	2.05	18.9300001	3.88300009	2.66999994	sp|O55028|B	[3-methyl-2-oxobutanoate dehydrogenase [lipoamide]] kinase, mitochondrial OS=Mus musculus GN=Bckdk PE	MOUSE	1	1.02801597	0.93837482	1.94088602	0.529663384	1.976969957	1.00925303	0.91495532	2.051162	0.492039502	2.070141077	1.05681801	0.81695801	1.65958703	0.636795521	1.753880978	
1023	2	2.05	21.2899998	6.43600002	6.43600002	sp|Q9JMG7|H	Hepatoma-derived growth factor-related protein 3 OS=Mus musculus GN=Hdgfrp3 PE=1 SV=2	MOUSE	1																
1024	2	2.05	11.8600003	3.39000002	3.39000002	sp|O88271|C	Craniofacial development protein 1 OS=Mus musculus GN=Cfdp1 PE=1 SV=1	MOUSE	1	1.48593605	0.4915731	2.10862804	0.704693079	3.104559898	1.55596602	0.45608521	2.0892961	0.744732022	3.250873089	1.52756596	0.47104079	2.10862804	0.724435985	3.191538095	
1025	2	2.05	4.43900004	3.39399986	2.08899993	sp|Q00420|G	GA-binding protein subunit beta-1 OS=Mus musculus GN=Gabpb1 PE=1 SV=2	MOUSE	1	1.51356101	0.47144651	2.0892961	0.724435985	3.191538095	1.158777	0.75359619	2.0892961	0.55462569	2.421029091	1.31825697	0.59480959	2.0892961	0.630957425	2.779712915	
1026	2	2.04	16.4000005	3.4740001	2.82300003	sp|A2ATU0|D	Probable 2-oxoglutarate dehydrogenase E1 component DHKTD1, mitochondrial OS=Mus musculus GN=Dhtkd	MOUSE	1																
1027	2	2.04	18.22	1.42099997	1.42099997	sp|Q6Y685|T	Transforming acidic coiled-coil-containing protein 1 OS=Mus musculus GN=Tacc1 PE=1 SV=1	MOUSE	1	0.70469308	0.81874323	16.4437199	0.050582461	11.58777046	1.02801597	0.99607301	8.8715601	0.115877703	9.120108604	0.83176369	0.8620922	2.60615301	0.316227794	2.167704105	
1028	2	2.04	15.6000003	2.30199993	2.30199993	sp|Q9R0N0|G	Galactokinase OS=Mus musculus GN=Galk1 PE=2 SV=1	MOUSE	1	0.86297852	0.86675012	1.599558	0.534564376	1.380383968	0.73113912	0.59165162	1.62929595	0.444631308	1.19124198	1	0.98591369	1.36772895	0.731139123	1.367728949	
1029	2	2.04	14.9900004	3.67799997	3.67799997	sp|Q8K3H0|D	DCC-interacting protein 13-alpha OS=Mus musculus GN=Appl1 PE=1 SV=1	MOUSE	1																
1030	2	2.04	11.5500003	1.89100001	1.89100001	sp|P21614|V	Vitamin D-binding protein OS=Mus musculus GN=Gc PE=1 SV=2	MOUSE	1	1.07646501	0.77940607	2.0892961	0.515228629	2.249054909	1.08642602	0.91352928	2.75422907	0.39445731	2.992264986	3.01995206	0.21243841	28.8403091	0.104712903	34.67367935	
1031	2	2.04	15.9799993	4.13199998	4.13199998	sp|Q8R1N4|N	NudC domain-containing protein 3 OS=Mus musculus GN=Nudcd3 PE=2 SV=3	MOUSE	1	0.8472274	0.77547568	6.2517271	0.135518894	5.296635151	0.73790431	0.58830541	2.12813902	0.346736789	1.570363045	0.89536482	0.83006811	2.0892961	0.424619585	1.870682001	
1032	2	2.04	9.78799984	2.91000009	2.91000009	sp|Q99PM3|T	Transcription initiation factor IIA subunit 1 OS=Mus musculus GN=Gtf2a1 PE=2 SV=2	MOUSE	1	0.29376501	0.32954669	2.10862804	0.130617097	0.619441092	0.57543987	0.53253752	2.0892961	0.27289781	1.202263951	0.08472274	0.261197	34.0408211	0.01499685	2.884032011	
1033	2	2.04	9.47000012	3.78799997	3.78799997	sp|P40936|I	Indolethylamine N-methyltransferase OS=Mus musculus GN=Inmt PE=2 SV=1	MOUSE	1	0.72443599	0.6730485	3.872576	0.187068194	2.805433989	0.66680682	0.57262307	2.0892961	0.316227794	1.393157005	0.55462569	0.46492341	2.58225989	0.212813899	1.432188034	
1034	2	2.04	10.1700001	7.90999979	7.90999979	sp|Q9CQ89|C	Protein CutA OS=Mus musculus GN=Cuta PE=2 SV=3	MOUSE	1	1.03752804	0.92096162	2.0892961	0.496592313	2.167704105	0.58076441	0.40389621	2.10862804	0.277971298	1.224616051	0.87902248	0.81445497	2.0892961	0.420726597	1.836537957	
1035	2	2.03	16.8599993	2.874	1.72400009	sp|Q9WV80|S	Sorting nexin-1 OS=Mus musculus GN=Snx1 PE=1 SV=1	MOUSE	1	0.87096357	0.86162132	7.65596581	0.113762699	6.668066978	1.04712904	0.9149015	2.6546061	0.39445731	2.754229069	0.87096357	0.80631828	2.0892961	0.416869402	1.819700956	
1036	2	2.03	12.7299994	2.17199996	2.17199996	sp|A2A7S8|K	Uncharacterized protein KIAA1522 OS=Mus musculus GN=Kiaa1522 PE=1 SV=1	MOUSE	1																
1037	2	2.03	38.1099999	6.14800006	6.14800006	sp|Q91X52|D	L-xylulose reductase OS=Mus musculus GN=Dcxr PE=2 SV=2	MOUSE	1	0.88715601	0.82131243	2.0892961	0.420726597	1.853531957	0.80167812	0.65730202	3.8370719	0.2108628	3.076097012	0.64863437	0.4771927	2.10862804	0.310455889	1.367728949	
1038	2	2.03	35.4099989	8.56000036	8.56000036	sp|Q9D1B9|R	39S ribosomal protein L28, mitochondrial OS=Mus musculus GN=Mrpl28 PE=2 SV=3	MOUSE	1	0.79432821	0.67037553	2.0892961	0.380189389	1.659587026	1.02801597	0.93912607	2.0892961	0.492039502	2.147830009	1.16949904	0.7486304	2.0892961	0.55975759	2.443430901	
1039	2	2.03	35.1700008	15.1700005	15.1700005	sp|P62843|R	40S ribosomal protein S15 OS=Mus musculus GN=Rps15 PE=2 SV=2	MOUSE	3	0.63679552	0.51484078	2.46603894	0.251188606	1.570363045	0.1923092	0.28615812	3.1045599	0.061376199	0.597035289	0.1541701	0.2400469	3.16227794	0.02805434	0.487528503	
1040	2	2.03	28.0600011	4.31700014	4.31700014	sp|Q8VEA4|M	Mitochondrial intermembrane space import and assembly protein 40 OS=Mus musculus GN=Chchd4 PE=1 SV	MOUSE	1	1.22461605	0.76119667	1.61435902	0.758577585	1.958845019	1.13762701	0.86495948	2.24905491	0.505824685	2.558585882	0.81658238	0.63474202	2.67916799	0.304789513	2.187762022	
1041	2	2.03	29.2899996	9.09100026	9.09100026	sp|P56376|A	Acylphosphatase-1 OS=Mus musculus GN=Acyp1 PE=2 SV=2	MOUSE	1	1.20226395	0.77319437	1.43218803	0.839460015	1.753880978	1	0.86250889	1.78648806	0.55975759	1.786488056	0.48305881	0.50082243	2.77971292	0.152756602	1.342764974	
1042	2	2.03	4.8560001	2.87800003	2.87800003	sp|O08966|S	Solute carrier family 22 member 1 OS=Mus musculus GN=Slc22a1 PE=2 SV=2	MOUSE	1	0.73113912	0.57925451	2.0892961	0.349945188	1.527565956	0.1737801	0.14052001	2.0892961	0.082413808	0.363078088	0.36643761	0.239676	2.10862804	0.175388098	0.772680581	
1043	2	2.02	17.6699996	1.53200002	1.53200002	sp|Q9EP71|R	Ankycorbin OS=Mus musculus GN=Rai14 PE=1 SV=1	MOUSE	1	1.23594701	0.67423028	2.0892961	0.591561615	2.582259893	2.31206489	0.27663499	2.10862804	1.096477985	4.830587864	1.47231197	0.49698141	2.0892961	0.704693079	3.076097012	
1044	2	2.02	15.9999996	2.31400002	2.31400002	sp|Q8R429|A	Sarcoplasmic/endoplasmic reticulum calcium ATPase 1 OS=Mus musculus GN=Atp2a1 PE=2 SV=1	MOUSE	1																
1044	0	2.01	15.23	2.20299996	2.20299996	sp|O55143|A	Sarcoplasmic/endoplasmic reticulum calcium ATPase 2 OS=Mus musculus GN=Atp2a2 PE=1 SV=2	MOUSE	1																
1045	2	2.02	18.6399996	2.99900007	1.30399996	sp|Q9D662|S	Protein transport protein Sec23B OS=Mus musculus GN=Sec23b PE=2 SV=1	MOUSE	1	0.53456438	0.56193417	4.40554905	0.121338896	2.355048895	0.83946002	0.77316022	1.75388098	0.478630096	1.472311974	0.75162292	0.68037659	1.39315701	0.539510608	1.047129035	
1045	0	2.01	25.7499993	3.00699994	1.30700003	sp|Q01405|S	Protein transport protein Sec23A OS=Mus musculus GN=Sec23a PE=1 SV=2	MOUSE	1																
1046	2	2.02	10.4099996	1.15700001	1.15700001	sp|Q8C0Q2|Z	Zinc fingers and homeoboxes protein 3 OS=Mus musculus GN=Zhx3 PE=2 SV=2	MOUSE	1	1.23594701	0.60813081	2.0892961	0.591561615	2.582259893	1.30617094	0.53114837	2.0892961	0.625172675	2.754229069	1.01859105	0.87673348	2.22843504	0.457088202	2.269865036	
1047	2	2.02	20.2999994	3.35600004	2.51700003	sp|Q5SWY8|S	Sodium/glucose cotransporter 5 OS=Mus musculus GN=Slc5a10 PE=2 SV=1	MOUSE	1	0.73790431	0.5654276	8.0909586	0.091201082	5.970353127	0.60255963	0.46060062	2.10862804	0.288403213	1.270573974	0.44463131	0.2986148	18.7068195	0.038018938	8.317637444	
1048	2	2.02	38.1700009	20.4300001	17.2000006	sp|Q9D6J5|N	NADH dehydrogenase [ubiquinone] 1 beta subcomplex subunit 8, mitochondrial OS=Mus musculus GN=Ndufb	MOUSE	2	1.19124198	0.79638273	2.0892961	0.570164323	2.511885881	1.18032098	0.77644682	2.10862804	0.55975759	2.488857031	1.23594701	0.75095248	2.10862804	0.586138189	2.582259893	
1049	2	2.02	16.2200004	3.2850001	3.2850001	sp|Q9JI11|ST	Serine/threonine-protein kinase 4 OS=Mus musculus GN=Stk4 PE=1 SV=1	MOUSE	1																
1050	2	2.02	40.2999997	8.45799968	8.45799968	sp|Q9D1G1|R	Ras-related protein Rab-1B OS=Mus musculus GN=Rab1b PE=1 SV=1	MOUSE	1	1.599558	0.43671399	2.0892961	0.765596628	3.34194994	1.81970096	0.36627141	2.10862804	0.862978518	3.80189395	1.54170001	0.46161401	2.0892961	0.73790431	3.221069098	
1051	2	2.02	11.5800001	4.07800004	4.07800004	sp|Q8BFW7|L	Lipoma-preferred partner homolog OS=Mus musculus GN=Lpp PE=1 SV=1	MOUSE	2	1.10662401	0.87247348	1.247383	0.88715601	1.380383968	1.07646501	0.964127	1.55596602	0.691830993	1.67494297	1.13762701	0.8441897	1.35518897	0.839460015	1.541700006	
1052	2	2.02	8.01699981	3.376	3.376	sp|Q9WUM4|	Coronin-1C OS=Mus musculus GN=Coro1c PE=1 SV=2	MOUSE	1	0.38370732	0.24922331	2.0892961	0.183653802	0.801678121	0.54450268	0.3701238	2.10862804	0.260615289	1.14815402	0.83946002	0.74117088	2.0892961	0.401790798	1.753880978	
1053	2	2.02	25.1500011	4.43800017	4.43800017	sp|Q9DAR7|D	Scavenger mRNA-decapping enzyme DcpS OS=Mus musculus GN=Dcps PE=1 SV=1	MOUSE	2	2.93764997	0.2195697	2.0892961	1.406048059	6.194410801	3.07609701	0.21217459	2.10862804	1.458814025	6.426877022	3.01995206	0.2150262	2.0892961	1.445440054	6.309574127	
1054	2	2.02	35.2299988	26.1400014	12.5	sp|P56135|A	ATP synthase subunit f, mitochondrial OS=Mus musculus GN=Atp5j2 PE=1 SV=3	MOUSE	2	0.95499259	0.97259837	1.19124198	0.801678121	1.137627006	0.97274721	0.97218293	1.21338904	0.801678121	1.180320978	1.05681801	0.8885895	1.14815402	0.920449615	1.213389039	

1055	2	2.02	15.9999996	9.20000002	5.99999987	sp|O88630|G	Golgi SNAP receptor complex member 1 OS=Mus musculus GN=Gosr1 PE=1 SV=1	MOUSE	1	1.30617094	0.6066159	2.0892961	0.625172675	2.728977919	1.20226395	0.71188778	2.0892961	0.57543987	2.511885881	1.05681801	0.88901132	2.0892961	0.505824685	2.22843504
1056	2	2.02	15.2500004	3.95499989	3.95499989	sp|O08691|AI	Arginase-2, mitochondrial OS=Mus musculus GN=Arg2 PE=1 SV=1	MOUSE	1															
1057	2	2.02	16.8500006	6.17999993	6.17999993	sp|Q9JM76|A	Actin-related protein 2/3 complex subunit 3 OS=Mus musculus GN=Arpc3 PE=1 SV=3	MOUSE	1	0.58613819	0.49950761	1.247383	0.469894111	0.731139123	0.51050502	0.4903501	1.78648806	0.280543387	0.912010789	0.77268058	0.69174022	1.31825697	0.580764413	1.018591046
1058	2	2.02	25	14.0599996	14.0599996	sp|Q3UIU2|N	NADH dehydrogenase [ubiquinone] 1 beta subcomplex subunit 6 OS=Mus musculus GN=Ndufb6 PE=2 SV=3	MOUSE	1	0.73790431	0.59114587	2.10862804	0.35318321	1.55596602	0.80167812	0.68835813	2.0892961	0.383707315	1.67494297	0.74473202	0.60240263	2.10862804	0.356451094	1.570363045
1059	2	2.02	5.28500006	2.43900009	2.43900009	sp|Q6PDL0|D	Cytoplasmic dynein 1 light intermediate chain 2 OS=Mus musculus GN=Dync1li2 PE=1 SV=2	MOUSE	2	1.16949904	0.76458168	2.0892961	0.55975759	2.466038942	1.29419601	0.63538009	2.10862804	0.613762021	2.703958035	1.52756596	0.48660529	3.98107195	0.383707315	6.08134985
1060	2	2.01	32.6099992	7.48799965	3.38199996	sp|Q9Z2E1|M	Methyl-CpG-binding domain protein 2 OS=Mus musculus GN=Mbd2 PE=2 SV=2	MOUSE	1	1.54170001	0.67530757	19.9526196	0.077268057	22.28434944	0.9289664	0.73979068	18.7068195	0.05152287	17.3780098	0.86297852	0.81193209	2.77971292	0.310455889	2.398833036
1061	2	2.01	12.3800002	1.95400007	1.95400007	sp|Q91ZR2|S	Sorting nexin-18 OS=Mus musculus GN=Snx18 PE=2 SV=1	MOUSE	1	1.97696996	0.4481163	1.75388098	1.127197981	3.767038107	1.31825697	0.66999549	2.0892961	0.630957425	2.85758996	0.60813499	0.6719324	1.62929595	0.319153786	0.990831971
1062	2	2.01	8.20700005	2.26799995	2.26799995	sp|Q3UW53|N	Protein Niban OS=Mus musculus GN=Fam129a PE=1 SV=2	MOUSE	1															
1063	2	2.01	24.1300002	3.19800004	3.19800004	sp|Q9CZA6|N	Nuclear distribution protein nudE homolog 1 OS=Mus musculus GN=Nde1 PE=1 SV=1	MOUSE	1	1.09647799	0.77890497	8.8715601	0.123594701	9.817479134	1.13762701	0.75265151	5.86138201	0.194088593	6.729765892	1.09647799	0.82191187	3.25087309	0.337287307	3.597492933
1063	0	2	22.9000002	3.18799987	3.18799987	sp|Q9ERR1|N	Nuclear distribution protein nudE-like 1 OS=Mus musculus GN=Ndel1 PE=1 SV=2	MOUSE	1															
1064	2	2.01	27.2700012	5.01599982	5.01599982	sp|P97429|AN	Annexin A4 OS=Mus musculus GN=Anxa4 PE=2 SV=4	MOUSE	1	0.86297852	0.78884947	2.10862804	0.413047493	1.819700956	0.81658238	0.71282423	2.10862804	0.390840888	1.721868992	2.37684012	0.26689339	2.0892961	1.137627006	5.011871815
1065	2	2.01	9.61199999	1.29399998	1.29399998	sp|O55201|SI	Transcription elongation factor SPT5 OS=Mus musculus GN=Supt5h PE=1 SV=1	MOUSE	1	1.10662401	0.89599443	15.2756596	0.072443597	15.70363045	1.47231197	0.41573101	21.8776207	0.067297667	23.12064934	1.04712904	0.90588212	19.2309208	0.05445027	19.23092079
1066	2	2.01	13.6099994	2.37600002	2.37600002	sp|Q9WTM5|I	RuvB-like 2 OS=Mus musculus GN=Ruvbl2 PE=2 SV=3	MOUSE	1	0.82413811	0.72482061	2.0892961	0.39445731	1.721868992	0.93756199	0.99010319	6.54636192	0.1432188	6.137619972	1.18032098	0.71250647	2.0892961	0.564936996	2.466038942
1067	2	2.01	44.9600011	4.65099998	4.65099998	sp|Q9QXV0|F	ProSAAS OS=Mus musculus GN=Pcsk1n PE=1 SV=2	MOUSE	1	1.41905701	0.53909749	3.1915381	0.444631308	4.487453938	1.30617094	0.61993498	2.10862804	0.619441092	2.728977919	0.97274721	0.97166133	2.0892961	0.465586096	2.032356977
1068	2	2.01	6.68400005	2.13900004	2.13900004	sp|Q9CW46|F	Ribonucleoprotein PTB-binding 1 OS=Mus musculus GN=Raver1 PE=1 SV=2	MOUSE	1															
1069	2	2.01	14.26	3.86999995	2.64800005	sp|Q8BGQ1|\	VPS33B-interacting protein OS=Mus musculus GN=Vipar PE=1 SV=1	MOUSE	1	2.85758996	0.58704788	27.2897797	0.104712903	37.67037964	2.75422907	0.4515332	16.5958691	0.165958703	37.32501984	2.75422907	0.56954938	26.7916794	0.102801599	38.01893997
1070	2	2.01	15.7499999	2.04700008	2.04700008	sp|Q61550|R	Double-strand-break repair protein rad21 homolog OS=Mus musculus GN=Rad21 PE=1 SV=3	MOUSE	1	1.07646501	0.96302372	2.58225989	0.416869402	2.779712915	0.92044962	0.74889332	9.20449543	0.100000001	8.472273827	1.13762701	0.67551768	2.0892961	0.544502676	2.376840115
1071	2	2.01	27.8299987	3.66999991	3.66999991	sp|Q6WVG3|I	BTB/POZ domain-containing protein KCTD12 OS=Mus musculus GN=Kctd12 PE=1 SV=1	MOUSE	1	0.7798301	0.65086353	2.0892961	0.373250186	1.629295945	1.02801597	0.93856162	2.0892961	0.492039502	2.147830009	1.03752804	0.92668909	2.0892961	0.496592313	2.167704105
1072	2	2.01	25.5699992	4.20700014	4.20700014	sp|P51949|M	CDK-activating kinase assembly factor MAT1 OS=Mus musculus GN=Mnat1 PE=2 SV=2	MOUSE	1															
1073	2	2.01	22.6099998	4.52300012	4.52300012	sp|Q3SXD3|H	HD domain-containing protein 2 OS=Mus musculus GN=Hddc2 PE=2 SV=1	MOUSE	1	1.51356101	0.50114697	2.10862804	0.717794299	3.162277937	0.77268058	0.58472413	20.5116196	0.04570882	15.84893036	0.73790431	0.55849862	21.0862808	0.044055492	15.55965996
1074	2	2.01	35.8700007	11.9599998	11.9599998	sp|P62305|RI	Small nuclear ribonucleoprotein E OS=Mus musculus GN=Snrpe PE=2 SV=1	MOUSE	1	0.4487454	0.36683661	2.72897792	0.165958703	1.224616051	0.564937	0.42908451	1.40604806	0.401790798	0.794328213	0.71121353	0.5473963	2.0892961	0.337287307	1.485936046
1075	2	2.01	52.579999	11.3399997	11.3399997	sp|Q9WVA2|T	Mitochondrial import inner membrane translocase subunit Tim8 A OS=Mus musculus GN=Timm8a1 PE=1 SV=	MOUSE	1	0.69823241	0.55595279	1.38038397	0.505824685	0.963828981	0.79432821	0.65073109	3.07609701	0.260615289	2.443430901	0.3981072	0.27596489	1.38038397	0.283139199	0.549540877
1075	0	2.01	35.0499988	11.3399997	11.3399997	sp|Q4FZG7|T	Putative mitochondrial import inner membrane translocase subunit Tim8 A-B OS=Mus musculus GN=Timm8a2	MOUSE	1															
1076	2	2.01	5.3679999	2.18700003	2.18700003	sp|Q9R0M4|F	Podocalyxin OS=Mus musculus GN=Podxl PE=1 SV=2	MOUSE	1	2.39883304	0.26861379	2.29086804	1.047129035	5.495409012	2.29086804	0.2891264	2.60615301	0.879022479	5.970353127	2.48885703	0.26058009	2.0892961	1.19124198	5.248075008
1077	2	2.01	15.3799996	3.49700004	3.49700004	sp|Q78JT3|3H	3-hydroxyanthranilate 3,4-dioxygenase OS=Mus musculus GN=Haao PE=1 SV=1	MOUSE	1	0.72443599	0.57399148	2.0892961	0.346736789	1.51356101	0.78704578	0.65997559	2.0892961	0.376703799	1.644371986	0.93756199	0.91479778	2.0892961	0.4487454	1.958845019
1078	2	2.01	20.9999993	5.49999997	5.49999997	sp|P61027|R	Ras-related protein Rab-10 OS=Mus musculus GN=Rab10 PE=1 SV=1	MOUSE	2															
1079	2	2.01	10.1599999	6.91099986	6.91099986	sp|Q9QUM9|I	Proteasome subunit alpha type-6 OS=Mus musculus GN=Psma6 PE=1 SV=1	MOUSE	1															
1080	2	2.01	20.0000003	16.55	16.55	sp|Q8K5B2|M	Multiple coagulation factor deficiency protein 2 homolog OS=Mus musculus GN=Mcfd2 PE=2 SV=1	MOUSE	1															
1081	2	2.01	22.0300004	9.3220003	9.3220003	sp|Q8BMC1|\	V-type proton ATPase subunit G 3 OS=Mus musculus GN=Atp6v1g3 PE=2 SV=1	MOUSE	3	1.31825697	0.5975554	2.0892961	0.630957425	2.754229069	1.33045399	0.59192359	2.0892961	0.636795521	2.805433989	1.21338904	0.67629242	2.10862804	0.57543987	2.53512907
1082	2	2.01	3.77399996	2.07499992	2.07499992	sp|Q8BG30|N	Negative elongation factor A OS=Mus musculus GN=Whsc2 PE=1 SV=1	MOUSE	1	1	0.97824699	2.0892961	0.478630096	2.108628035	0.69183099	0.53139699	2.10862804	0.331131101	1.458814025	0.96382898	0.95977628	2.0892961	0.461317599	2.013724089
1083	2	2	17.7399993	1.76299997	1.76299997	sp|Q9JIK5|DI	Nucleolar RNA helicase 2 OS=Mus musculus GN=Ddx21 PE=1 SV=3	MOUSE	1															
1084	2	2	17.4500004	1.61600001	1.61600001	sp|Q9JLZ6|HI	Hypermethylated in cancer 2 protein OS=Mus musculus GN=Hic2 PE=2 SV=4	MOUSE	1	1.41905701	0.53716552	3.90840912	0.363078088	5.597576141	1.45881403	0.54956043	5.64937019	0.258226007	8.241380692	2.07014108	0.3445906	4.83058786	0.428548515	10
1085	2	2	24.6700004	4.33299989	4.33299989	sp|Q9D7J9|E	Enoyl-CoA hydratase domain-containing protein 3, mitochondrial OS=Mus musculus GN=Echdc3 PE=2 SV=1	MOUSE	1	0.70469308	0.54234242	2.0892961	0.337287307	1.472311974	0.80167812	0.67921943	2.0892961	0.383707315	1.67494297	0.74473202	0.59520298	2.0892961	0.356451094	1.55596602
1086	2	2	45.9100008	12.8900006	6.91799969	sp|Q8R0Y8|S	Solute carrier family 25 member 42 OS=Mus musculus GN=Slc25a42 PE=2 SV=1	MOUSE	1	1.12719798	0.82156378	2.0892961	0.539510608	2.582259893	0.73113912	0.62073427	2.10862804	0.077983007	1.541700006	0.82413811	0.75537628	2.0892961	0.244343102	1.721868992
1087	2	2	17.4500004	3.38499993	3.38499993	sp|Q8BWM0|	Prostaglandin E synthase 2 OS=Mus musculus GN=Ptges2 PE=1 SV=3	MOUSE	1															
1088	2	2	41.8599993	7.55800009	7.55800009	sp|Q9R1K9|C	Centrin-2 OS=Mus musculus GN=Cetn2 PE=1 SV=1	MOUSE	1	1.73780096	0.367028	2.10862804	0.824138105	3.630779982	1.48593605	0.41649821	3.49945211	0.424619585	5.199960232	1.78648806	0.34146419	2.0892961	0.855066717	3.767038107
1089	2	2	19.8699996	4.10099998	4.10099998	sp|Q8BGV3|T	Tumor-associated calcium signal transducer 2 OS=Mus musculus GN=Tacstd2 PE=2 SV=1	MOUSE	1	0.7798301	0.65317887	2.10862804	0.373250186	1.644371986	1.29419601	0.62383032	2.10862804	0.613762021	2.703958035	1.38038397	0.54951459	2.0892961	0.660693526	2.91071701
1090	2	2	18.0299997	3.94399986	3.94399986	sp|Q60739|B	BAG family molecular chaperone regulator 1 OS=Mus musculus GN=Bag1 PE=1 SV=3	MOUSE	1	1.247383	0.63968182	2.0892961	0.597035289	2.630268097	1.04712904	0.92111868	2.0892961	0.501187205	2.187762022	0.94623709	0.90205258	2.22843504	0.424619585	2.108628035
1091	2	2	14.61	2.62199994	2.62199994	sp|P50429|AI	Arylsulfatase B OS=Mus musculus GN=Arsb PE=2 SV=3	MOUSE	1	1.158777	0.7361868	2.0892961	0.55462569	2.421029091	1.158777	0.78526288	3.8370719	0.301995188	4.446312904	1.39315701	0.53733432	2.18776202	0.636795521	3.047894955
1092	2	2	13.7099996	5.01700006	5.01700006	sp|Q9JHQ5|L	Leucine zipper transcription factor-like protein 1 OS=Mus musculus GN=Lztfl1 PE=2 SV=1	MOUSE	1	1.09647799	0.97356021	1.41905701	0.772680581	1.55596602	0.79432821	0.59492421	1.47231197	0.544502676	1.16949904	0.73790431	0.64242733	1.247383	0.597035289	0.920449615
1093	2	2	13.6800006	4.21099998	4.21099998	sp|Q9DCX8|I	Iodotyrosine dehalogenase 1 OS=Mus musculus GN=Iyd PE=1 SV=1	MOUSE	1	0.96382898	0.94766319	2.0892961	0.457088202	2.013724089	1.22461605	0.67811781	2.3550489	0.519995987	2.91071701	0.7798301	0.66929507	2.10862804	0.373250186	1.644371986
1094	2	2	19.8300004	2.91499998	2.91499998	sp|Q9CZ42|C	Carbohydrate kinase domain-containing protein OS=Mus musculus GN=Carkd PE=1 SV=1	MOUSE	1	0.2013724	0.83511472	32.2106895	0.021478301	6.486343861	1.28233099	0.65894008	3.80189395	0.337287307	4.920394897	1.31825697	0.69682747	2.20800495	0.597035289	2.937649965
1095	2	2	15.49	3.63299996	3.63299996	sp|Q8R1Q8|D	Cytoplasmic dynein 1 light intermediate chain 1 OS=Mus musculus GN=Dync1li1 PE=1 SV=1	MOUSE	1	2.93764997	0.5532043	2.10862804	1.393157005	18.36537933	3.66437602	0.53668243	2.60615301	1.406048059	20.32357025	2.85758996	0.68536258	2.51188588	1.137627006	12.47383022
1096	2	2	23.59	10.7699998	10.7699998	sp|Q63829|C	COMM domain-containing protein 3 OS=Mus musculus GN=Commd3 PE=2 SV=1	MOUSE	1	0.7798301	0.65368682	2.10862804	0.373250186	1.644371986	1.19124198	0.72146761	2.10862804	0.564936996	2.488857031	0.70469308	0.54514849	2.10862804	0.337287307	1.485936046
1097	2	2	40.2999997	7.95999989	7.95999989	sp|Q9R0Q3|T	Transmembrane emp24 domain-containing protein 2 OS=Mus musculus GN=Tmed2 PE=1 SV=1	MOUSE	1															
1098	2	2	10.7299998	1.73499994	1.73499994	sp|Q9D687|S	Sodium-dependent neutral amino acid transporter B(0)AT1 OS=Mus musculus GN=Slc6a19 PE=1 SV=1	MOUSE	1	0.89536482	0.83830309	2.0892961	0.428548515	1.870682001	0.69183099	0.52551079	2.0892961	0.331131101	1.445440054	0.74473202	0.59961009	2.0892961	0.356451094	1.55596602
1099	2	2	15.1600003	5.31900004	5.31900004	sp|Q91WU5|A	Arsenite methyltransferase OS=Mus musculus GN=As3mt PE=2 SV=2	MOUSE	1	0.72443599	0.57319492	2.0892961	0.346736789	1.51356101	1.20226395	0.71370208	2.10862804	0.570164323	2.511885881	0.86297852	0.78775758	2.0892961	0.413047493	1.803017974
1100	2	2	16.1300004	2.48099994	2.48099994	sp|Q8VE47|U	Ubiquitin-like modifier-activating enzyme 5 OS=Mus musculus GN=Uba5 PE=2 SV=2	MOUSE	1	1.61435902	0.4318867	2.10862804	0.765596628	3.372873068	1.90546095	0.3436648	2.0892961	0.912010789	3.981071949	0.89536482	0.83292908	2.0892961	0.428548515	1.870682001
1101	2	2	35.5800003	10.5800003	10.5800003	sp|Q8BTE0|C	UPF0369 protein C6orf57 homolog OS=Mus musculus PE=2 SV=2	MOUSE	1	14.7231302	0.2537849	31.9153805	0.461317599	69.18309784	14.1905804	0.27520731	31.9153805	0.444631308	68.5488205	11.5877705	0.41448569	31.6227799	0.366437614	63.67955017
1102	2	2	20.3600004	11.27	11.27	sp|Q8BFY6|P	Peflin OS=Mus musculus GN=Pef1 PE=2 SV=1	MOUSE	1															
1103	2	2	11.8799999	3.83099988	3.83099988	sp|P97765|W	WW domain-binding protein 2 OS=Mus musculus GN=Wbp2 PE=1 SV=1	MOUSE	1	0.79432821	0.67261362	2.0892961	0.380189389	1.659587026	0.78704578	0.6566031	2.0892961	0.373250186	1.644371986	0.61944109	0.44004771	2.0892961	0.293765008	1.29419601
1104	2	2	13.5299996	6.76300004	6.76300004	sp|P55258|R	Ras-related protein Rab-8A OS=Mus musculus GN=Rab8a PE=1 SV=2	MOUSE	1	0.38018939	0.2467404	2.0892961	0.1803018	0.794328213	0.69183099	0.52662498	2.0892961	0.331131101	1.445440054	0.73113912	0.57529211	2.0892961	0.346736789	1.527565956
1105	2	2	9.80399996	4.70600016	4.70600016	sp|Q9QZ23|N	NFU1 iron-sulfur cluster scaffold homolog, mitochondrial OS=Mus musculus GN=Nfu1 PE=1 SV=2	MOUSE	1	0.77268058	0.81008732	2.20800495	0.346736789	1.706081986	1.05681801	0.97093099	2.39883304	0.440554887	2.53512907	0.72443599	0.75357229	2.18776202	0.325087309	1.584892988
1106	2	2	21.0800007	5.38100004	5.38100004	sp|Q9DCH6|Z	AN1-type zinc finger protein 6 OS=Mus musculus GN=Zfand6 PE=1 SV=1	MOUSE	1	1.28233099	0.63425899	2.10862804	0.608134985	2.679167986	0.86297852	0.78856081	2.10862804	0.413047493	1.819700956	1.08642602	0.84722263	2.0892961	0.519995987	2.290868044
1107	2	2	18.1299999	7.69200027	7.69200027	sp|Q8C5P7|C	Uncharacterized protein C8orf42 homolog OS=Mus musculus PE=2 SV=1	MOUSE	1	1.05681801	0.96931732	2.22843504	0.474242002	2.376840115	0.81658238	0.80866212	2.10862804	0.390840888	1.721868992	0.71121353	0.69176841	2.80543399	0.246603906	1.995262027
1108	2	2	17.3600003	6.03800006	6.03800006	sp|Q78IK4|AI	Apolipoprotein O-like OS=Mus musculus GN=Apool PE=2 SV=1	MOUSE	1	0.27289781	0.54984713	29.1071701	0.02333458	7.943282127	0.49659231	0.42814231	2.33345795	0.2108628	1.158776999	0.29376501	0.39760661	21.4783001	0.02728978	6.309574127
1109	2	2	9.64699984	2.35300008	2.35300008	sp|Q60973|RI	Histone-binding protein RBBP7 OS=Mus musculus GN=Rbbp7 PE=1 SV=1	MOUSE	1	0.73790431	0.6376186	2.96483111	0.248885706	2.187762022	0.77268058	0.70421952	2.10862804	0.369828194	1.629295945	1.08642602	0.94540739	4.09260702	0.26546061	4.487453938
1110	2	2	35.710001	8.57099965	8.57099965	sp|P45878|FK	Peptidyl-prolyl cis-trans isomerase FKBP2 OS=Mus musculus GN=Fkbp2 PE=1 SV=1	MOUSE	1	0.67297667	0.76852977	10.7646503	0.063679554	7.244359016	0.77268058	0.75322199	2.10862804	0.369828194	1.629295945	0.61376202	0.54558653	2.55858588	0.239883304	1.570363045
1111	2	2	32.8500003	8.02899972	8.02899972	sp|Q9DBX1|F	Response gene to complement 32 protein OS=Mus musculus GN=Rgc32 PE=2 SV=1	MOUSE	1	1.12719798	0.82073468	19.7696991	0.057016429	20.13723946	0.73790431	0.55928588	17.8648796	0.050582461	13.18257046	1.13762701	0.82646912	19.0546093	0.059703529	19.40885925
1112	2	2	38.3899987	28.5699993	28.5699993	sp|Q9D1C3|F	Protein preY, mitochondrial OS=Mus musculus GN=Prey PE=2 SV=1	MOUSE	1															
1113	2	2	14.3800005	7.8429997	7.8429997	sp|Q8R2K1|F	Fucose mutarotase OS=Mus musculus GN=Le51 PE=1 SV=1	MOUSE	1	0.99083197	0.95138198	3.46736908	0.285759091	3.435580015	1.158777	0.82779717	6.2517271	0.185353205	7.244359016	1.07646501	0.94602132	6.66806698	0.161435902	7.112134933
1114	2	2	9.68699977	4.68799993	4.68799993	sp|Q8BP92|R	Reticulocalbin-2 OS=Mus musculus GN=Rcn2 PE=2 SV=1	MOUSE	2															
1115	2	2	36.3599986	32.2299987	9.91699994	sp|P61110|AI	Kidney androgen-regulated protein OS=Mus musculus GN=Kap PE=2 SV=1	MOUSE	1	0.67297667	0.5097068	2.46603894	0.27289781	1.659587026	0.86297852	0.78847867	2.0892961	0.413047493	1.803017974	0.68548822	0.52835947	2.0892961	0.325087309	1.432188034
1116	2	2	30.399999	18.4	18.4	sp|P60898|RI	DNA-directed RNA polymerase II subunit RPB9 OS=Mus musculus GN=Polr2i PE=2 SV=1	MOUSE	1															
1117	2	2	4.63399999	2.19500009	2.19500009	sp|P18242|C	Cathepsin D OS=Mus musculus GN=Ctsd PE=1 SV=1	MOUSE	1	0.58076441	0.3623859	7.04693079	0.081658237	4.092607021	0.85506672	0.81926572	5.44502592	0.157036304	4.655860901	0.91201079	0.88713598	2.96483111	0.307609707	2.703958035
1118	2	2	36.2599999	21.9799995	21.9799995	sp|Q9D1L9|H	Hepatitis B virus X-interacting protein homolog OS=Mus musculus GN=Hbxip PE=2 SV=1	MOUSE	1	1.01859105	0.95498401	2.16770411	0.469894111	2.208004951	1.21338904	0.69847029	2.0892961	0.580764413	2.53512907	1.07646501	0.82072258	6.91831017	0.155596599	7.447319984
1119	2	2	14.9399996	9.76999998	9.76999998	sp|Q9CWZ3|I	RNA-binding protein 8A OS=Mus musculus GN=Rbm8a PE=1 SV=3	MOUSE	1	0.83176369	0.73979092	2.0892961	0.398107201	1.737800956	0.75857759	0.62609261	2.0892961	0.363078088	1.584892988	0.73790431	0.58689922	2.0892961	0.35318321	1.541700006
1120	2	2	13.5100007	13.5100007	13.5100007	sp|Q8R034|A	Anaphase-promoting complex subunit 13 OS=Mus musculus GN=Anapc13 PE=2 SV=1	MOUSE	1	2.53512907	0.251176	2.0892961	1.213389039	5.345643997	1.10662401	0.82655227	2.0892961	0.529663384	2.312064886	1.04712904	0.91196519	2.0892961	0.501187205	2.187762022
1121	2	2	5.57399988	3.93400006	3.93400006	sp|Q5QNS5|H	Hepatitis A virus cellular receptor 1 homolog OS=Mus musculus GN=Havcr1 PE=1 SV=2	MOUSE	1	1.52756596	0.46519521	2.0892961	0.731139123	3.221069098	1.51356101	0.4755871	2.0892961	0.724435985	3.162277937	3.8370719	0.1788329	2.0892961	1.836537957	8.090958595
1122	2	2	17.7100003	10.4199998	10.4199998	sp|Q06318|U	Uteroglobin OS=Mus musculus GN=Scgb1a1 PE=1 SV=1	MOUSE	1	1.158777	0.82393843	2.22843504	0.519995987	2.582259893	1.19124198	0.78336501	1.80301797	0.660693526	2.128139019	1.34276497	0.6162824	1.75388098	0.765596628	2.376840115
1123	2	2	13.0700007	13.0700007	13.0700007	sp|P19258|M	Protein Mpv17 OS=Mus musculus GN=Mpv17 PE=2 SV=1	MOUSE	1															
1124	2	2	5.10899983	2.92000007	2.92000007	sp|O09172|G	Glutamate--cysteine ligase regulatory subunit OS=Mus musculus GN=Gclm PE=2 SV=1	MOUSE	1	0.79432821	0.6873219	2.0892961	0.380189389	1.659587026	0.95499259	0.94069737	2.0892961	0.457088202	1.995262027	0.93756199	0.91462028	2.0892961	0.4487454	1.958845019
1125	2	2	10.5300002	10.5300002	10.5300002	sp|Q9R257|H	Heme-binding protein 1 OS=Mus musculus GN=Hebp1 PE=1 SV=1	MOUSE	1	1.40604806	0.51145172	18.3653793	0.076559663	19.95261955	1.00925303	0.96930373	2.0892961	0.48305881	2.128139019	1.21338904	0.72123212	2.60615301	0.465586096	3.133285999
1126	2	2	13.0999997	13.0999997	13.0999997	sp|Q9CQ91|N	NADH dehydrogenase [ubiquinone] 1 alpha subcomplex subunit 3 OS=Mus musculus GN=Ndufa3 PE=1 SV=1	MOUSE	1	1.07646501	0.87178582	2.0892961	0.515228629	2.249054909	1.10662401	0.82310551	2.0892961	0.529663384	2.312064886	1.35518897	0.56753641	2.0892961	0.648634374	2.83139205
1127	1.92	2.04	13.5399997	1.48799997	1.48799997	sp|Q8K2C7|C	Protein OS-9 OS=Mus musculus GN=Os9 PE=1 SV=2	MOUSE	1	2.01372409	0.64844191	24.6603909	0.081658237	27.79713058	0.77268058	0.85811788	20.7014103	0.044463132	15.99557972	2.07014108	0.50084651	2.10862804	0.981747925	4.405549049
1128	1.89	2.43	15.4400006	8.08800012	6.86300024	sp|Q8BLF1|A	Neutral cholesterol ester hydrolase 1 OS=Mus musculus GN=Nceh1 PE=1 SV=1	MOUSE	1	0.78704578	0.7303912	2.70395804	0.288403213	2.128139019	0.88715601	0.87189949	2.0892961	0.420726597	1.853531957	0.93756199	0.93041772	2.0892961	0.4487454	1.958845019
1129	1.89	1.89	16.7300001	9.79600027	9.79600027	sp|O55135|IF	Eukaryotic translation initiation factor 6 OS=Mus musculus GN=Eif6 PE=1 SV=2	MOUSE	1	1.04712904	0.9068678	2.0892961	0.501187205	2.187762022	0.93756199	0.90551871	2.0892961	0.4487454	1.958845019	1.67494297	0.4058187	2.0892961	0.801678121	3.53183198
1130	1.86	4.93	33.5500002	4.56000008	4.56000008	sp|P35922|FI	Fragile X mental retardation protein 1 homolog OS=Mus musculus GN=Fmr1 PE=1 SV=1	MOUSE	3	0.52480751	0.35244349	2.10862804	0.251188606	1.106624007	0.83946002	0.74586028	2.0892961	0.401790798	1.753880978	0.64268768	0.46892691	2.0892961	0.307609707	1.342764974
1131	1.85	2.51	8.76900032	1.48700001	0.56409999	sp|A2ASQ1|A	Agrin OS=Mus musculus GN=Agrn PE=1 SV=1	MOUSE	1	1.158777	0.5895251	4.83058786	0.239883304	5.105050087	0.30760971	0.64364821	5.59757614	0.068548821	1.721868992	1.00925303	0.53874642	4.57088184	0.220800504	4.570881844
1132	1.85	2.05	6.29400015	4.1960001	4.1960001	sp|P59325|IF	Eukaryotic translation initiation factor 5 OS=Mus musculus GN=Eif5 PE=1 SV=1	MOUSE	2	0.88715601	0.85641128	1.69044101	0.524807513	1.499685049	0.88715601	0.91442567	1.97696996	0.4487454	1.753880978	1.02801597	0.60433477	2.14783001	0.478630096	2.208004951

1133	1.85	1.85	10.9999999	6.13799989	6.13799989	sp|Q9WUR2|I	Enoyl-CoA delta isomerase 2, mitochondrial OS=Mus musculus GN=Eci2 PE=1 SV=2	MOUSE	1																
1134	1.83	1.83	21.3799998	9.65500027	9.65500027	sp|Q9D6K5|S	Synaptojanin-2-binding protein OS=Mus musculus GN=Synj2bp PE=1 SV=1	MOUSE	1	1.08642602	0.88510323	8.79022503	0.123594701	9.549925804	0.90364939	0.99770641	9.37561989	0.096382901	8.472273827	0.58076441	0.40444371	10.4712896	0.0564937	6.08134985	
1135	1.82	2.12	36.9599998	8.2510002	6.9310002	sp|Q91WT4|D	DnaJ homolog subfamily C member 17 OS=Mus musculus GN=Dnajc17 PE=2 SV=1	MOUSE	1																
1136	1.8	1.87	12.8000006	2.68300008	2.073	sp|Q61165|SI	Sodium/hydrogen exchanger 1 OS=Mus musculus GN=Slc9a1 PE=1 SV=1	MOUSE	1	2.0892961	0.3068203	2.0892961	1	4.405549049	1.67494297	0.4068425	2.0892961	0.801678121	3.499452114	1.83653796	0.3580499	2.0892961	0.879022479	3.872575998	
1137	1.8	1.82	11.5099996	3.7659999	3.7659999	sp|P50431|GI	Serine hydroxymethyltransferase, cytosolic OS=Mus musculus GN=Shmt1 PE=1 SV=3	MOUSE	1	0.63095743	0.4541204	2.0892961	0.301995188	1.318256974	0.59703529	0.42000929	2.0892961	0.285759091	1.247382998	0.44463131	0.28787059	2.0892961	0.2108628	0.928966403	
1138	1.8	1.81	23.0800003	7.69200027	7.69200027	sp|Q8K3A0|H	Iron-sulfur cluster co-chaperone protein HscB, mitochondrial OS=Mus musculus GN=Hscb PE=2 SV=2	MOUSE	1	0.73113912	0.71204382	2.42102909	0.296483099	1.770109057	0.61376202	0.4733924	7.11213493	0.08550667	4.365159035	0.30478951	0.34701371	16.9044094	0.02805434	5.152287006	
1139	1.8	1.8	30.0000012	11.1500002	6.53799996	sp|Q3U0B3|D	Dehydrogenase/reductase SDR family member 11 OS=Mus musculus GN=Dhrs11 PE=2 SV=1	MOUSE	1																
1140	1.77	1.78	15.7800004	2.75199991	2.75199991	sp|Q8K1N4|S	Spermatogenesis-associated serine-rich protein 2 OS=Mus musculus GN=Spats2 PE=1 SV=1	MOUSE	1	1.44544005	0.51233751	2.10862804	0.685488224	3.019952059	2.53512907	0.25179389	2.10862804	1.202263951	5.296635151	1.85353196	0.3566311	2.10862804	0.879022479	3.872575998	
1141	1.77	1.77	9.27200019	8.60899985	8.60899985	sp|P62264|R	40S ribosomal protein S14 OS=Mus musculus GN=Rps14 PE=2 SV=3	MOUSE	1	1.61435902	0.92401189	19.9526196	0.080909587	27.03957939	1.67494297	0.85788119	19.0546093	0.087902263	27.28977966	2.75422907	0.48329032	24.4343109	0.1127197	36.9828186	
1142	1.76	1.76	72.7299988	47.7299988	47.7299988	sp|Q6ZWY8|T	Thymosin beta-10 OS=Mus musculus GN=Tmsb10 PE=2 SV=3	MOUSE	2	1.81970096	0.40834469	1.95884502	0.928966403	4.698941231	2.0892961	0.35553601	1.78648806	1.16949904	5.152287006	2.051162	0.34462231	1.51356101	1.355188966	4.168694019	
1143	1.75	1.78	32.5500011	2.93300003	2.93300003	sp|Q8VCN9|T	Tubulin-specific chaperone C OS=Mus musculus GN=Tbcc PE=2 SV=1	MOUSE	1	0.80167812	0.75257838	2.10862804	0.383707315	1.690441012	1.61435902	0.4819831	4.16869402	0.387257606	6.79203701	1.75388098	0.43214601	2.80543399	0.625172675	5.058247089	
1144	1.73	2.17	30.6800008	2.77299993	1.84799992	sp|Q99MN9|F	Propionyl-CoA carboxylase beta chain, mitochondrial OS=Mus musculus GN=Pccb PE=1 SV=2	MOUSE	1	0.76559663	0.63370788	2.10862804	0.366437614	1.614359021	0.53456438	0.35868561	2.0892961	0.253512889	1.116863012	1.10662401	0.82025421	2.0892961	0.529663384	2.312064886	
1145	1.73	1.82	16.0600007	6.73599988	4.14500013	sp|Q9QUI0|R	Transforming protein RhoA OS=Mus musculus GN=Rhoa PE=1 SV=1	MOUSE	1	1.01859105	0.90960997	1.18032098	0.862978518	1.202263951	0.9289664	0.73324877	1.34276497	0.691830993	1.247382998	1.10662401	0.94586569	1.28233099	0.862978518	1.419057012	
1145	0	1.82	15.0299996	6.73599988	4.14500013	sp|Q62159|RI	Rho-related GTP-binding protein RhoC OS=Mus musculus GN=Rhoc PE=1 SV=2	MOUSE	1																
1146	1.71	1.77	8.94500017	3.44000012	2.06400007	sp|Q7TNE1|C	CaiB/baiF CoA-transferase family protein C7orf10 homolog OS=Mus musculus PE=2 SV=1	MOUSE	1	0.1158777	0.77544677	31.6227799	0.01721869	3.66437602	0.78704578	0.83041322	2.10862804	0.376703799	1.659587026	0.91201079	0.89606923	2.16770411	0.420726597	1.976969957	
1147	1.7	1.76	13.8500005	2.88500004	2.88500004	sp|Q921T2|T	Torsin-1A-interacting protein 1 OS=Mus musculus GN=Tor1aip1 PE=1 SV=2	MOUSE	1	1.00925303	0.9786858	2.0892961	0.48305881	2.108628035	1.03752804	0.93579882	2.0892961	0.496592313	2.167704105	0.8472274	0.78972149	11.9124203	0.07112135	10.09253025	
1148	1.7	1.7	13.3900002	5.41299991	5.41299991	sp|P51863|V	V-type proton ATPase subunit d 1 OS=Mus musculus GN=Atp6v0d1 PE=1 SV=2	MOUSE	2	0.63095743	0.45343861	2.0892961	0.301995188	1.318256974	0.61944109	0.44189721	2.0892961	0.296483099	1.29419601	0.68548822	0.51608342	2.0892961	0.328095287	1.432188034	
1149	1.7	1.7	16.4100006	11.7200002	11.7200002	sp|Q80WK2|C	Organic solute transporter subunit beta OS=Mus musculus GN=Ostb PE=1 SV=1	MOUSE	1	0.7177943	0.56009978	2.0892961	0.343558013	1.499685049	1.08642602	0.85031241	2.0892961	0.519995987	2.269865036	0.90364939	0.84630758	2.0892961	0.432513803	1.887990952	
1150	1.69	1.72	6.13799989	2.81300005	2.81300005	sp|P28656|N	Nucleosome assembly protein 1-like 1 OS=Mus musculus GN=Nap1l1 PE=1 SV=2	MOUSE	1																
1151	1.69	1.7	57.6900005	25.9600014	10.5800003	sp|Q8BK30|N	NADH dehydrogenase [ubiquinone] flavoprotein 3, mitochondrial OS=Mus musculus GN=Ndufv3 PE=2 SV=1	MOUSE	1	1.54170001	0.27663589	1.55596602	0.990831971	2.779712915	1.39315701	0.41758511	1.55596602	0.895364821	2.421029091	0.61944109	0.33988839	1.80301797	0.144544005	1.116863012	
1152	1.66	4.35	29.0300012	2.87100002	2.87100002	sp|Q9JIG7|C	Coiled-coil domain-containing protein 22 OS=Mus musculus GN=Ccdc22 PE=1 SV=1	MOUSE	2	0.89536482	0.94905633	1.78648806	0.492039502	1.599557996	0.88715601	0.9522562	3.8370719	0.224905506	3.40408206	1.13762701	0.82965678	2.07014108	0.549540877	2.355048895	
1153	1.65	1.71	14.3099994	1.33199999	0.90239998	sp|Q8VHY0|C	Chondroitin sulfate proteoglycan 4 OS=Mus musculus GN=Cspg4 PE=1 SV=2	MOUSE	1	1	0.98754948	2.0892961	0.478630096	2.089296103	1	0.99068469	2.0892961	0.478630096	2.089296103	0.85506672	0.7684772	2.0892961	0.40926069	1.786488056	
1154	1.64	1.64	5.86699992	1.06699998	1.06699998	sp|Q61391|N	Neprilysin OS=Mus musculus GN=Mme PE=1 SV=3	MOUSE	1	0.64863437	0.47387359	2.0892961	0.310455889	1.355188966	0.51999599	0.3465949	2.0892961	0.248885706	1.08642602	0.58613819	0.4093807	2.0892961	0.280543387	1.224616051	
1155	1.64	1.64	27.5599986	18.5900003	18.5900003	sp|Q9CR21|A	Acyl carrier protein, mitochondrial OS=Mus musculus GN=Ndufab1 PE=1 SV=1	MOUSE	6	0.90364939	0.80745113	1.23594701	0.724435985	1.116863012	0.69823241	0.50878531	1.247383	0.564936996	0.870963573	0.57543987	0.4085913	1.52756596	0.369828194	0.879022479	
1156	1.62	1.62	30.3799987	6.48500025	4.0959999	sp|P25444|R	40S ribosomal protein S2 OS=Mus musculus GN=Rps2 PE=1 SV=3	MOUSE	1	1.07646501	0.66012847	1.30617094	0.824138105	1.406048059	1.28233099	0.25551099	1.49968505	0.855066717	1.923092008	1.22461605	0.33166	1.28233099	0.954992592	1.570363045	
1157	1.6	1.66	16.5900007	3.92999984	3.92999984	sp|Q61206|P	Platelet-activating factor acetylhydrolase IB subunit beta OS=Mus musculus GN=Pafah1b2 PE=1 SV=2	MOUSE	1	0.93756199	0.94014663	1.45881403	0.642687678	1.367728949	0.75162292	0.40448001	1.36772895	0.534564376	1.028015971	0.83176369	0.56485182	1.49968505	0.544502676	1.247382998	
1158	1.57	1.67	10.3299998	3.73600014	2.63700001	sp|Q71RI9|K	Kynurenine--oxoglutarate transaminase 3 OS=Mus musculus GN=Ccbl2 PE=1 SV=1	MOUSE	1	0.4613176	0.3523581	24.8885708	0.034355789	11.48153973	0.38018939	0.26723921	2.0892961	0.1803018	0.794328213	0.2355049	0.2066112	31.0456009	0.023768401	7.311390877	
1159	1.57	1.58	18.0600002	1.13599999	1.13599999	sp|Q6NZF1|Z	Zinc finger CCCH domain-containing protein 11A OS=Mus musculus GN=Zc3h11a PE=1 SV=1	MOUSE	1	2.39883304	0.26545861	2.10862804	1.137627006	5.011871815	1.95884502	0.33105439	2.0892961	0.937561989	4.130475044	2.60615301	0.24454729	2.0892961	1.247382998	5.495409012	
1160	1.57	1.57	19.1799998	3.28800008	3.28800008	sp|P01900|H	H-2 class I histocompatibility antigen, D-D alpha chain OS=Mus musculus GN=H2-D1 PE=1 SV=1	MOUSE	1	0.63095743	0.7719928	22.2843494	0.036982819	14.06048012	1.01859105	0.79365522	18.19701	0.055975761	18.03017998	2.53512907	0.3653788	2.10862804	1.202263951	5.395105839	
1160	0	1.57	18.7900007	4.02700007	4.02700007	sp|P01895|H	H-2 class I histocompatibility antigen, alpha chain (Fragment) OS=Mus musculus GN=H2-D1 PE=2 SV=1	MOUSE	1																
1160	0	1.55	13.4499997	3.36100012	3.36100012	sp|P06339|H	H-2 class I histocompatibility antigen, D-37 alpha chain OS=Mus musculus GN=H2-T23 PE=1 SV=1	MOUSE	1																
1161	1.57	1.57	50.4899979	17.4799994	17.4799994	sp|Q9CQQ8|I	U6 snRNA-associated Sm-like protein LSm7 OS=Mus musculus GN=Lsm7 PE=2 SV=1	MOUSE	1	1.62929595	0.43852839	2.0892961	0.779830098	3.435580015	2.42102909	0.27334431	2.10862804	1.14815402	5.058247089	1.158777	0.76994282	2.10862804	0.549540877	2.421029091	
1162	1.55	7.78	25.1399994	14.6400005	12.9800007	sp|Q61990|P	Poly(rC)-binding protein 2 OS=Mus musculus GN=Pcbp2 PE=1 SV=1	MOUSE	4	1.08642602	0.92304349	2.12813902	0.510505021	2.312064886	1.14815402	0.81665558	3.04789496	0.376703799	3.499452114	1.01859105	0.93475449	2.0892961	0.487528503	2.128139019	
1163	1.55	2.24	25.4599989	5.77399992	4.19900008	sp|Q04447|K	Creatine kinase B-type OS=Mus musculus GN=Ckb PE=1 SV=1	MOUSE	1	1.04712904	0.62011111	1.99526203	0.524807513	2.089296103	1.20226395	0.2075758	1.247383	0.963828981	1.499685049	1.33045399	0.116122	1.62929595	0.816582382	2.167704105	
1164	1.55	1.55	6.9310002	6.9310002	6.9310002	sp|P63166|S	Small ubiquitin-related modifier 1 OS=Mus musculus GN=Sumo1 PE=1 SV=1	MOUSE	1	0.70469308	0.61353022	8.01678085	0.088715598	5.649370193	0.69823241	0.59303772	8.16582394	0.084722742	5.70164299	0.73113912	0.56918728	2.77971292	0.263026804	2.032356977	
1165	1.54	1.58	8.85199979	1.79399997	1.79399997	sp|Q8C0C0|Z	Zinc fingers and homeoboxes protein 2 OS=Mus musculus GN=Zhx2 PE=1 SV=1	MOUSE	1	1.247383	0.50419271	19.7696991	0.063095734	20.32357025	0.89536482	0.81536132	3.49945211	0.2558586	3.133285999	0.83946002	0.7530306	18.5353203	0.05152287	15.55965996	
1166	1.54	1.57	14.35	4.54499982	2.87100002	sp|Q99JY9|A	Actin-related protein 3 OS=Mus musculus GN=Actr3 PE=1 SV=3	MOUSE	1																
1167	1.54	1.54	30.250001	8.40300024	8.40300024	sp|Q60692|P	Proteasome subunit beta type-6 OS=Mus musculus GN=Psmb6 PE=1 SV=3	MOUSE	2	0.69823241	0.58358997	1.51356101	0.465586096	1.056818008	0.7798301	0.86293483	2.55858588	0.304789513	1.995262027	0.85506672	0.89939791	1.77010906	0.48305881	1.51356101	
1168	1.54	1.54	12.3599999	2.47200001	2.47200001	sp|Q61598|G	Rab GDP dissociation inhibitor beta OS=Mus musculus GN=Gdi2 PE=1 SV=1	MOUSE	1	0.4875285	0.39772481	2.29086804	0.194088593	1.116863012	0.57016432	0.4135403	1.34276497	0.383707315	0.765596628	0.66680682	0.51510358	1.20226395	0.544502676	0.801678121	
1169	1.53	1.67	12.8299996	3.04700006	2.12399997	sp|Q9WVL3|S	Solute carrier family 12 member 7 OS=Mus musculus GN=Slc12a7 PE=1 SV=1	MOUSE	2	1.23594701	0.66758579	2.0892961	0.591561615	2.582259893	1.23594701	0.67022288	2.0892961	0.591561615	2.582259893	1.54170001	0.46266109	2.0892961	0.73790431	3.221069098	
1170	1.53	1.55	15.8099994	2.75699999	2.75699999	sp|Q8BFR4|G	N-acetylglucosamine-6-sulfatase OS=Mus musculus GN=Gns PE=2 SV=1	MOUSE	2	0.75857759	0.77093422	3.80189395	0.197696999	2.884032011	1.18032098	0.6148383	2.77971292	0.424619585	3.28095293	1.12719798	0.63922417	3.2210691	0.349945188	3.630779982	
1171	1.53	1.53	22.9300007	2.83799991	2.83799991	sp|Q9JIY5|H	Serine protease HTRA2, mitochondrial OS=Mus musculus GN=Htra2 PE=1 SV=2	MOUSE	1	0.91201079	0.86010939	4.57088184	0.197696999	4.168694019	0.79432821	0.66487002	4.78630114	0.162929595	3.80189395	1.22461605	0.67549127	4.69894123	0.260615289	5.861382008	
1172	1.52	1.52	35.5300009	30.2599996	14.4700006	sp|Q9D115|Z	Zinc finger protein 706 OS=Mus musculus GN=Znf706 PE=2 SV=1	MOUSE	1	1.27057397	0.5459221	1.23594701	1.028015971	1.584892988	1.10662401	0.66720402	1.95884502	0.564936996	2.167704105	0.95499259	0.9082368	2.26986504	0.420726597	2.167704105	
1173	1.49	1.58	26.9800007	6.54800013	3.57099995	sp|Q91YW3|D	DnaJ homolog subfamily C member 3 OS=Mus musculus GN=Dnajc3 PE=1 SV=1	MOUSE	1	0.69183099	0.5243665	2.0892961	0.328095287	1.445440054	0.53951061	0.3662113	2.10862804	0.258226007	1.137627006	1.16949904	0.74494529	2.0892961	0.55975759	2.443430901	
1174	1.47	1.6	17.1700001	1.58699993	1.58699993	sp|Q3THK7|G	GMP synthase [glutamine-hydrolyzing] OS=Mus musculus GN=Gmps PE=1 SV=2	MOUSE	1	0.52480751	0.3524597	2.0892961	0.251188606	1.096477985	0.50118721	0.33171111	2.0892961	0.239883304	1.047129035	0.68548822	0.52309942	2.10862804	0.328095287	1.445440054	
1175	1.46	1.53	43.4500009	10.7100003	4.16699983	sp|Q9D7P6|I	Iron-sulfur cluster assembly enzyme ISCU, mitochondrial OS=Mus musculus GN=Iscu PE=1 SV=1	MOUSE	1	0.85506672	0.76525509	2.0892961	0.40926069	1.786488056	1.06659603	0.88875622	2.10862804	0.505824685	2.22843504	1.02801597	0.93318182	2.0892961	0.492039502	2.147830009	
1176	1.45	1.6	7.90800005	1.23600001	0.82369996	sp|Q6GQT9|N	Nodal modulator 1 OS=Mus musculus GN=Nomo1 PE=1 SV=1	MOUSE	1	1.57036305	0.4455086	2.0892961	0.751622915	3.311311007	1.30617094	0.60939312	2.0892961	0.625172675	2.728977919	2.51188588	0.25257751	2.0892961	1.202263951	5.296635151	
1177	1.44	5.89	55.9199989	36.1799985	23.68	sp|P15532|N	Nucleoside diphosphate kinase A OS=Mus musculus GN=Nme1 PE=1 SV=1	MOUSE	6	1.08642602	0.83120352	7.24435902	0.149968505	7.870458126	0.85506672	0.80730993	3.1045599	0.275422901	2.654606104	0.7798301	0.68108553	2.10862804	0.373250186	1.644371986	
1178	1.44	1.53	13.1600007	1.99100003	1.63599998	sp|Q3UJB9|E	Enhancer of mRNA-decapping protein 4 OS=Mus musculus GN=Edc4 PE=1 SV=2	MOUSE	1	1.00925303	0.97129351	2.0892961	0.48305881	2.108628035	1.20226395	0.7102471	2.0892961	0.57543987	2.511885881	1.16949904	0.74507308	2.0892961	0.55975759	2.443430901	
1179	1.44	1.44	39.1799986	5.26299998	5.26299998	sp|Q9CQ45|N	Neudesin OS=Mus musculus GN=Nenf PE=1 SV=1	MOUSE	1	1.06659603	0.90500093	2.0892961	0.510505021	2.22843504	1.20226395	0.62862808	2.70395804	0.444631308	3.250873089	1.35518897	0.60302109	2.22843504	0.608134985	3.019952059	
1180	1.41	1.41	16.2900001	12.3599999	6.17999993	sp|Q6P8I4|P	PEST proteolytic signal-containing nuclear protein OS=Mus musculus GN=Pcnp PE=1 SV=1	MOUSE	1																
1181	1.4	2.12	11.9000003	2.84400005	0.85979998	sp|Q8CGC7|S	Bifunctional aminoacyl-tRNA synthetase OS=Mus musculus GN=Eprs PE=2 SV=4	MOUSE	2	0.83176369	0.71233273	1.247383	0.666806817	1.037528038	0.9289664	0.99088353	1.20226395	0.772680581	1.116863012	0.90364939	0.9925763	1.34276497	0.672976673	1.213389039	
1182	1.38	1.38	30.0599992	7.05500022	3.98800001	sp|Q8BVI5|S	Syntaxin-16 OS=Mus musculus GN=Stx16 PE=1 SV=3	MOUSE	1																
1183	1.37	1.43	29.4099987	12.2500002	12.2500002	sp|P97461|R	40S ribosomal protein S5 OS=Mus musculus GN=Rps5 PE=2 SV=3	MOUSE	2	2.37684012	0.2675499	2.0892961	1.137627006	5.011871815	2.01372409	0.32099769	2.0892961	0.963828981	4.207265854	2.29086804	0.27886429	2.10862804	1.08642602	4.786301136	
1184	1.36	1.37	18.2500005	11.6800003	11.6800003	sp|Q9CR61|N	NADH dehydrogenase [ubiquinone] 1 beta subcomplex subunit 7 OS=Mus musculus GN=Ndufb7 PE=1 SV=3	MOUSE	1	0.7798301	0.68348402	1.34276497	0.57543987	1.047129035	0.7177943	0.68565083	1.52756596	0.461317599	1.096477985	0.98174793	0.89285392	1.48593605	0.660693526	1.458814025	
1185	1.34	1.35	10.8400002	2.13200003	2.13200003	sp|O88343|S	Electrogenic sodium bicarbonate cotransporter 1 OS=Mus musculus GN=Slc4a4 PE=1 SV=2	MOUSE	3	0.99083197	0.93893671	1.52756596	0.654636085	1.51356101	0.83946002	0.77197832	1.40604806	0.267916799	1.180320978	1.67494297	0.20934001	2.75422907	0.608134985	5.199960232	
1186	1.33	1.4	20.2399999	1.26499999	0.94879996	sp|Q9R0B6|L	Laminin subunit gamma-3 OS=Mus musculus GN=Lamc3 PE=2 SV=2	MOUSE	1																
1187	1.32	1.32	16.2000001	4.93000001	4.93000001	sp|P62743|A	AP-2 complex subunit sigma OS=Mus musculus GN=Ap2s1 PE=1 SV=1	MOUSE	1	0.67920363	0.51426578	2.10862804	0.325087309	1.432188034	0.42461959	0.27458361	2.0892961	0.2013724	0.88715601	0.79432821	0.67643368	2.0892961	0.380189389	1.659587026	
1188	1.31	1.34	27.4500012	4.31400016	4.31400016	sp|Q9JKK1|S	Syntaxin-6 OS=Mus musculus GN=Stx6 PE=2 SV=1	MOUSE	1	1.06659603	0.88695163	2.10862804	0.505824685	2.22843504	0.87096357	0.8036176	2.10862804	0.416869402	1.836537957	0.99083197	0.9987877	2.0892961	0.474242002	2.070141077	
